# π-Aggregation-free, fused perylene pentamers: synthesis, narrowband far-red to near-infrared emission, and chiroptical properties

**DOI:** 10.1039/d6sc00676k

**Published:** 2026-04-30

**Authors:** Qifeng Zhou, Lin-Tao Bao, Rui Liu, Zhitao Sun, Zhuolin Ye, Liuying Jiao, Wei Fan, Ya Zou, Hai-Bo Yang, Jishan Wu

**Affiliations:** a Department of Chemistry, National University of Singapore 3 Science Drive 3 117543 Singapore zouya@nus.edu.sg chmwuj@nus.edu.sg; b Shanghai Key Laboratory of Green Chemistry and Chemical Processes, School of Chemistry and Molecular Engineering, East China Normal University Shanghai 200062 China hbyang@chem.ecnu.edu.cn; c School of Chemistry and Chemical Engineering, Beijing Institute of Technology Beijing 100081 China

## Abstract

Chiral nanographenes have emerged as promising materials for chiral optoelectronics owing to their intrinsic chiroptical properties. However, their development remains constrained by synthetic challenges, strong π-aggregation, and low fluorescence quantum yields, while emission extending to the near-infrared (NIR) region is still rare. Here, we present a molecular design strategy that combines structural multiplicity with π-extension in a butterfly-shaped fused perylene pentamer scaffold to achieve active circularly polarized luminescence (CPL) emitters. By tuning the Scholl reaction conditions, we selectively obtained either racemic 1a (1a-*rac*) together with its *meso*-isomer (1a-*meso*) or an extended series of nanographenes (1a–1d). X-ray crystallography revealed contorted architectures featuring helicene subunits, while bulky aryl substituents improved solubility, enhanced stability, and enabled enantiomer separation. Owing to their extended π-conjugation, perylene-like frontier orbital distribution, and increased molecular symmetry and rigidity, these nanographenes exhibit highly tunable and remarkable optical and chiroptical properties. Notably, 1a demonstrates outstanding chiroptical performance (*Φ*_F_ = 65%; *B*_CPL_ = 66.7 M^−1^ cm^−1^), whereas 1d exhibits narrowband emission (FWHM = 37 nm) spanning the far-red to NIR region.

## Abstract 2

π-Aggregation-free, fused perylene pentamers were synthesized in one pot, showing high solubility and fluorescence. Isomeric and π-extension tuning enables visible to pure far-red to NIR emission with narrow bandwidths and enhanced CPL brightness.

## Graphical abstract

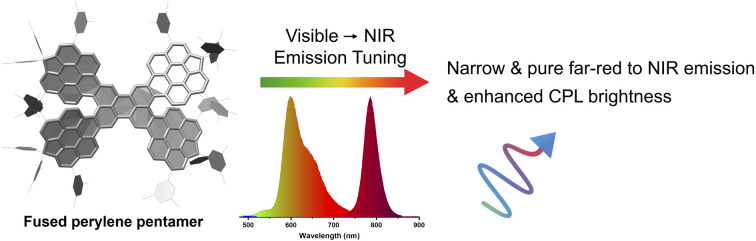

## Introduction

Chiral nanographenes are an unconventional class of π-conjugated materials that deviate from planar graphene fragments and have attracted increasing interest due to their unique electronic structures, pronounced molecular chirality, and intriguing optical properties.^1^ Unlike planar graphene cutouts, chiral nanographenes incorporate helicenes or contorted polycyclic aromatic hydrocarbons, resulting in three-dimensional architectures.^2^ The combination of extended π-electron delocalization and structural chirality gives rise to strong electronic circular dichroism (ECD) and circularly polarized luminescence (CPL),^3^ enabling applications in circularly polarized organic light-emitting diodes (CP-OLEDs),^4^ spin filters,^5^ and chiral photodetectors.^6^ This integration of π-extension and chirality has thus emerged as a powerful design paradigm for functional organic materials, with numerous studies reporting diverse chiral nanographenes featuring novel topologies and pronounced chiroptical responses.^7^

Despite these advances, achieving chiral nanographenes that combine high fluorescence quantum yields (*Φ*_F_), strong CPL, and emission extending into the near-infrared (NIR) region (>800 nm) remains challenging.^8^ Extended π-systems are prone to strong intermolecular π–π interactions, which induce aggregation-caused quenching (ACQ) and reduce luminescence efficiency.^9^ For CPL-active materials, suppressed emission directly limits CPL brightness (*B*_CPL_),^10^ defined as the product of *Φ*_F_ and the luminescence dissymmetry factor (*g*_lum_).^11^ Designing nanographenes that resist π-aggregation while retaining rigidity and extended conjugation therefore remains a key bottleneck.^12^ Furthermore, while most studies focus on visible-light chiral emitters,^13^ efficient far-red to NIR chiral emitters are rare yet highly desirable for bioimaging,^14^ biosensing,^15^ and optoelectronic applications.^16^ Achieving far-red to NIR emission is challenging because the energy gap law predicts lower quantum yields at longer wavelengths.^17^

**Fig. 1:**
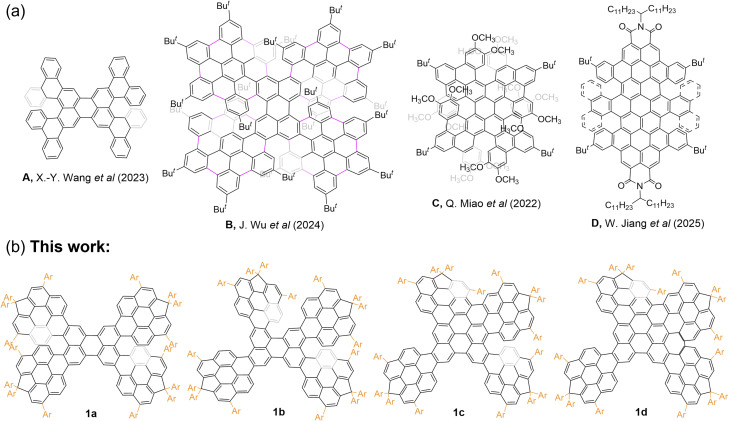
(a) Representative examples of the reported perylene-embedded chiral nanographene. (b) Novel fused perylene pentamers 1a–d reported in this work.

Recent efforts illustrate both progress and limitations. Wang and co-workers^18^ reported chiral nanographenes with high *Φ*_F_*via* helical π-extension of perylene, but emission remained in the visible range (A, Fig. 1a). Chaolumen's^19^ “double-twist” pyrene-based nanographenes achieved red-shifted luminescence with moderate *Φ*_F_ (∼58%), though CPL brightness was modest. Our^20^ previous perylene-based helicenes extended emission to 1010 nm, but with enhanced π–π interactions that reduced *Φ*_F_ (B, Fig. 1a). Similarly, Miao^21^ and Jiang^22^ expanded perylene diimides into chiral nanographenes with emission spanning the far-red to NIR region, yet efficiency losses persisted (C–D, Fig. 1a). Incorporation of bulky *bay* substituents has been shown to suppress π-aggregation and improve solubility, highlighting a strategy to mitigate ACQ.^23^

These precedents suggest key design principles: π-extension and structural multiplicity enable far-red to NIR absorption and emission, molecular rigidity and symmetry reduce nonradiative decay and improve *Φ*_F_, and bulky substituents prevent π-aggregation. Perylene scaffolds are particularly attractive due to high intrinsic fluorescence, favourable frontier molecular orbital (FMO) distributions, and versatile *bay* positions for steric protection.^24^ Yet, embedding perylene into extended, rigid architectures while retaining strong emission and robust CPL remains a major challenge.

Here, we report the synthesis of a series of π-aggregation-free, fused perylene pentamers 1a–d with butterfly-like architectures that integrate π-extension, structural multiplicity, and steric protection (Fig. 1b). Suzuki coupling and Scholl reactions yield racemic and *meso* isomers and a family of contorted nanographenes confirmed by X-ray crystallography. Bulky aryl (Ar) substituents and *bay*-fused cyclopentadiene (CP) rings prevent π-aggregation, stabilize enantiomers, and enable efficient chiral HPLC separation. Extended π-conjugation preserves perylene-like FMO distributions, producing emission spanning the far-red to NIR region. Notably, compound 1a-*rac* exhibits high *Φ*_F_ (65%) and *B*_CPL_ (66.7 M^−1^ cm^−1^), while 1d exhibits narrowband emission extending from the far-red into the NIR region. This work demonstrates a generalizable strategy for designing chiral nanographenes that are π-aggregation-free, enantiomerically stable, and tunable in optical and chiroptical properties, achieving simultaneously high fluorescence efficiency and active CPL within a single molecular family.

## Results and discussion

### Design and synthesis

**Scheme 1:**
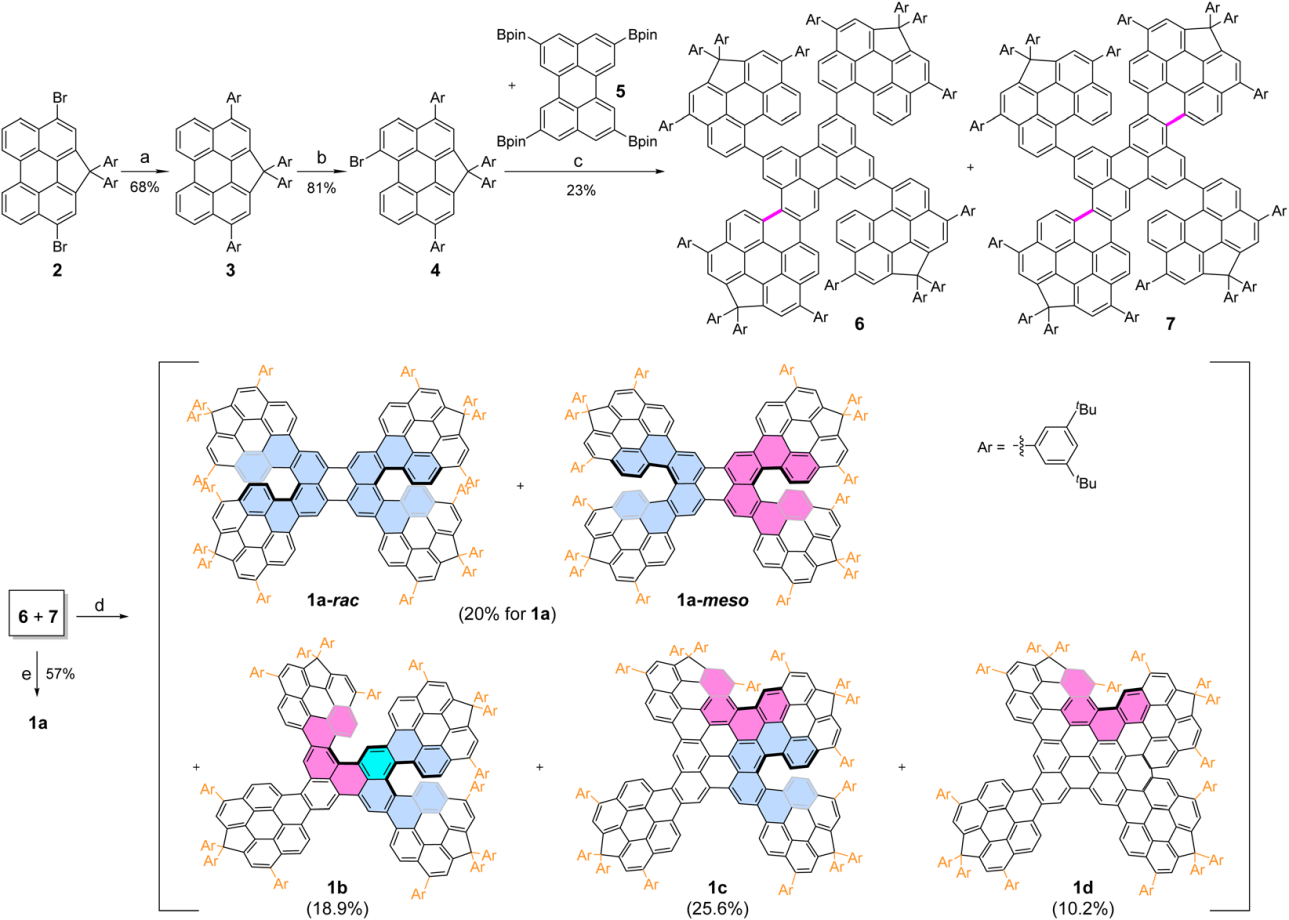
Synthetic route of compounds 1a–d. Reaction conditions: (a) ArB(OH)_2_ (4.0 eq.), Pd(dppf)Cl_2_·CH_2_Cl_2_, K_2_CO_3_, THF/H_2_O, 80 °C, overnight; (b) Br_2_, DCM, r.t., 1 h; (c) Pd_2_(dba)_3_·CHCl_3_, Sphos, Cs_2_CO_3_, toluene/H_2_O, 90 °C; 32 h; (d) DDQ, TfOH, DCM, r.t., 1.5 h; (e) FeCl_3_, MeNO_2_, DCM, r.t., 1 h.

To access the desired π-aggregation-free, fused perylene pentamers, two modified perylene building blocks, 4 and 5, were designed and synthesized (Scheme 1). Building block 5,^25^ bearing four pinacol boronate (Bpin) groups, provides a perylene-embedded core for the pentamers and acts as a strongly luminescent chromophore. Building block 4, a CP-fused perylene featuring sp^3^-carbons at one *bay* position, was prepared from compound 2^26^ using a modified protocol. Specifically, compound 2 was coupled with (3,5-di-*tert*-butylphenyl) boronic acid under Suzuki conditions to afford compound 3 in 68% yield. Subsequent bromination of 3 with Br_2_ gave building block 4 in 81% yield. The Ar substituents (3,5-di-*tert*-butylphenyl), introduced both at the sp^3^-carbons of the fused CP and the *peri* positions, enhance the solubility of 4, its intermediates, and the final pentamers.

Suzuki coupling between building blocks 4 and 5 yielded a mixture of products 6 and 7 in 23% yield. Compound 6 features a perylene core with a single Ar-substituted perylene unit fused at one *peri*-position. By contrast, compound 7 contains two such units, fused diagonally at the *peri*-positions of the core. Surprisingly, the expected pentamer 8, in which five perylene units are connected by four C–C single bonds, was not observed (see details in the SI). Instead, a domino process occurred, generating new C–C bonds *via* an unusual C–H transformation.^27^ This unusual reactivity may arise from a palladium-catalyzed C–H activation/C–C cross-coupling pathway, possibly facilitated by the electron-rich, multi-perylene precursor.^28^ A possible reaction mechanism is proposed (Fig. S1). Although the expected product 8 was not obtained under the typical Suzuki coupling conditions, the products 6 and 7 did not affect the subsequent Scholl reaction toward the target fused pentamers. Therefore, no further optimization to obtain 8 was pursued, and compounds 6 and 7 were directly used in the next step.

The final perylene-fused pentamers were obtained *via* the Scholl cyclodehydrogenation reaction,^29^ which displayed condition-dependent selectivity. Using DDQ/TfOH, both *peri* and *bay* positions underwent cyclodehydrogenation, yielding a family of tailored pentamers (1a–d) in 10.2–25.6% yields (Scheme 1). Importantly, this method avoided producing a complex mixture of random fusion products, instead affording structurally defined pentamers (Fig. S34 and S35). In contrast, the FeCl_3_-mediated Scholl reaction induced cyclodehydrogenation exclusively at the *peri* positions, affording pentamer 1a in 57% yield. Both racemic (1a-*rac*) and *meso* (1a-*meso*) isomers were obtained in a nearly 1 : 1 ratio as determined by ^1^H NMR (Fig. S10 and S66–S71), whereas diastereomers for 1b–d were not observed. These findings highlight the distinct regioselectivity imparted by different Scholl reaction conditions^30^ and provide new insights into the construction of molecular nanocarbons.

### X-ray crystallographic analysis

**Fig. 2:**
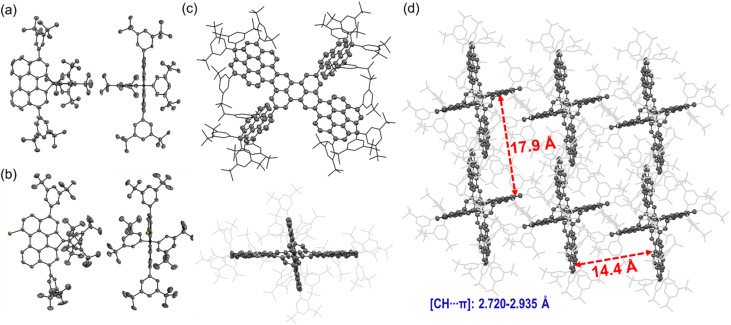
Top view and side view of X-ray crystallographic structures of (a) 3, (b) 4 and (c) 7; (d) molecular packing of X-ray crystallographic structures of 7.

The solid-state structures of building block 4, its precursor 3, and the intermediate 7 were determined by single-crystal X-ray diffraction (XRD),^31^ providing insights into their conformations and packing arrangements (Fig. 2). Single crystals suitable for XRD analysis were obtained through slow solvent diffusion or evaporation. Specifically, crystals of 3 and 7 were grown by slow diffusion of methanol into their chloroform and dichloromethane (DCM) solutions, respectively, while crystals of 4 were obtained by slow vapor diffusion of acetonitrile into its DCM solution. From the side view of 3, the backbone adopts a nearly planar conformation (Fig. 2a). In contrast, the backbone of 4 exhibits a slightly bent conformation due to the combined effects of the CP ring fused at the *bay* positions and steric hindrance from the bromine substituent at another *bay* position (Fig. 2b). The Br atom at the *bay*-position is oriented outward because of steric repulsion. In both 3 and 4, the Ar groups attached to the *peri* positions and sp^3^-carbons orient perpendicularly to the perylene backbone. This orthogonal arrangement of bulky Ar substituents effectively suppresses π–π stacking by occupying the interstitial space between adjacent perylene backbones.

The intermediate 7, obtained after Suzuki coupling, adopts a butterfly-like conformation (Fig. 2c and d). The three fused perylene units form a slightly twisted plane, while the two singly linked perylene units lie in another plane, resulting in a cross-shaped arrangement. The Ar groups are distributed around this butterfly-like skeleton and oriented perpendicularly to each connected perylene backbone, thereby creating an environment in which the π-skeleton is effectively encapsulated within the molecule. The distances between the two perylene-based cross planes were measured to be approximately 17.9 Å and 14.4 Å, respectively—significantly larger than the 3.5 Å separation observed between parent perylene molecules in the solid state.^32^ This expanded spacing demonstrates π-aggregation-free crystal packing and highlights the effectiveness of bulky Ar substituents in suppressing π–π aggregation between perylene backbones.

**Fig. 3:**
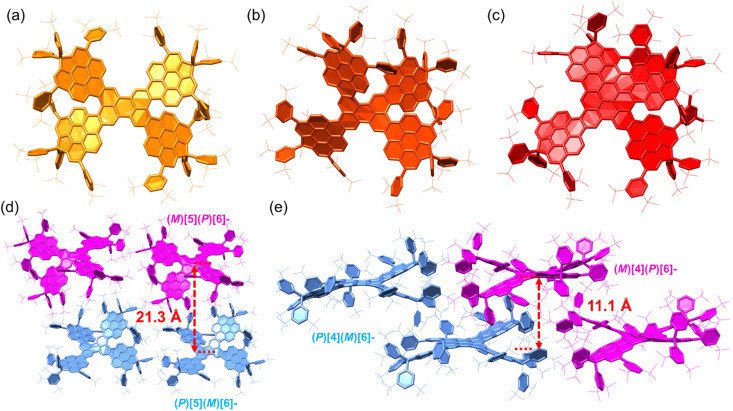
X-ray crystallographic structures of (a) 1a-*meso*, (b) 1b, and (c) 1c, and the packing of (d) 1b and (e) 1c.

**Fig. 4:**
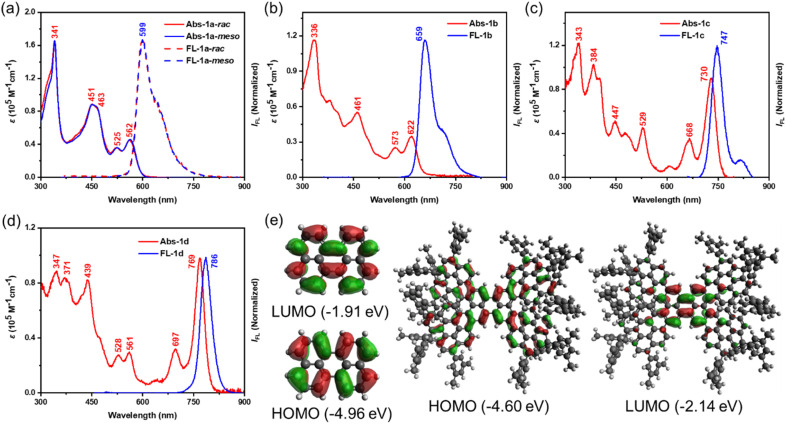
UV-vis absorption (Abs) and fluorescence (FL) spectra of (a) 1a-*rac* and 1a-*meso*, (b) 1b, (c) 1c, and (d) 1d; (e) calculated frontier molecular orbital profiles of perylene and 1a-*rac*. The *tert*-butyl substituents were replaced by methyl groups for the calculations.

The structures of the final fused perylene pentamers 1a-*meso*, 1b, and 1c were unambiguously confirmed by single-crystal X-ray diffraction (Fig. 3).^31^ Single crystals of 1a-*meso* and 1b were obtained by slow diffusion of methanol into their solutions in *o*-DCB/CS_2_ (4 : 1) and chlorobenzene/CS_2_ (3 : 1), respectively, while crystals of 1c were grown by slow diffusion of acetonitrile into its toluene/DCM (2 : 3) solution. Crystals of 1a-*rac* and 1d were also obtained but proved too unstable for accurate structure determination, as weak diffraction signals were observed due to solvent loss upon removal from the mother liquor. This instability likely originates from weak intermolecular interactions in the packing, consistent with their pronounced π-aggregation-free behaviour. The possible structures of 1d are illustrated in Fig. S36. Considering the fusion trend from 1a to 1c, together with the *C*_1_ structure confirmed by NMR analysis (Fig. S17, S18, S75 and S76), structure A in Fig. S36, corresponding to 1d in Scheme 1, is assigned as the most plausible structure. Furthermore, the electronic properties are in good agreement with the calculated results (Fig. 4d, S28, S48, S49 and Table S5), which further supports this assignment.

XRD analysis confirmed the fused perylene pentamer skeletons and butterfly-like geometries of 1a-*meso*, 1b, and 1c (Fig. 3). In 1a-*meso*, all Ar-substituted perylene units (Ar–Per) are fused at the *peri*-positions of the perylene (Per) core, generating two extended [6]helicene motifs along opposite sides of the backbone, with bulky substituents distributed around the pentamer framework (Fig. 3a). In 1b, one Ar–Per unit is fused at a *bay* position rather than a *peri*-position. This structural change gives rise to an extended [6]helicene on the *peri* side and an extended [5]helicene on the *bay* side, while the remaining Ar–Per units retain the perpendicular orientation relative to the Per backbone, preserving π-aggregation suppression (Fig. 3b and d). 1c builds on the structure of 1b, incorporating two additional fusions of the Ar–Per unit that forms the [5]helicene in 1b (Fig. 3c and e). Specifically, the *ortho*-position of this Ar–Per unit couples with the *bay* position of the Per core, while its *peri*-position couples with the *ortho*-position of an adjacent Ar–Per unit. These additional fusions enlarge the π-skeleton, forming an extended [6]helicene on the *peri* side and an extended [4]helicene on the *bay* side. The two opposite *peri*-fused Ar–Per units together with the Per core generate a wave-like extended heptacene motif, while on each side, the *bay*-fused Ar–Per unit cooperates with the adjacent *peri*-fused Ar–Per units and the Per core to form stable helical termini, giving rise to *P*[4]*M*[6] or *M*[4]*P*[6] enantiomers. In all three pentamers, the Ar substituents are oriented perpendicular to the Per backbones, consistent with the structures of 3 and 4. This orthogonal arrangement effectively prevents π–π stacking, thereby minimizing π-aggregation. The crystal packing structures (Fig. 3d, e and S51–S61) further highlight this π-aggregation-free behaviour: the Ar groups occupy the intermolecular spaces around the pentamer backbones, eliminating π–π interactions among the π-conjugated cores.

Enantiomers are equally present in the packing structures of 1b and 1c—*P*[5]*M*[6]/*M*[5]*P*[6] for 1b and *P*[4]*M*[6]/*M*[4]*P*[6] for 1c (Fig. 3d, e, S58 and S60). The distances between paired enantiomers are large (21.3 Å for 1b and 11.1 Å for 1c), reflecting pronounced non-π-aggregation. Each enantiomeric pair assembles into heterochiral stacked chains in an alternating pattern, predominantly mediated by [CH⋯π] interactions and van der Waals forces (Fig. S58 and S60). Collectively, these weak intermolecular interactions stabilize the crystals and support the formation of a three-dimensional supramolecular architecture (Fig. S59 and S61).

### Photophysical properties

To elucidate the photophysical properties of 1a–d, their UV-vis-NIR absorption and fluorescence spectra were recorded in dilute DCM solutions (*c* = 1.0 × 10^−5^ M) at room temperature (Fig. 4). Compared with perylene,^33^ all compounds display pronounced red-shifts owing to their extended π-conjugation. For the perylene pentamers 1a-*rac* and 1a-*meso*, the absorption spectra are nearly identical, with the lowest-energy absorption maxima at 562 nm and 564 nm, respectively, along with a strong absorption band at ∼341 nm (Fig. 4a). TD-DFT calculations (Tables S1 and S2) assign the lowest-energy bands to HOMO–LUMO electronic transitions. The calculated FMOs of both isomers closely resemble those of perylene (Fig. 4e), and they also display similar absorption maxima (Fig. S38a and S41).

DFT-calculated energy levels show identical LUMO energies (−2.14 eV) and comparable HOMO energies (−4.60 eV for 1a-*rac* and −4.56 eV for 1a-*meso*) (Fig. 4e, S37 and S40). Compound 1b exhibits a markedly red-shifted absorption profile, with a lowest-energy maximum at 622 nm extending into the far-red region (Fig. 4b). TD-DFT analysis attributes this band to the HOMO–LUMO transition (Table S3). The shift arises from the fusion of one Ar–Per unit with the perylene core at the *bay* position rather than the *peri* position, leading to altered electronic structures: a lowered LUMO (−2.28 eV) and a raised HOMO (−4.49 eV) (Fig. S42). This highlights the critical role of subtle structural changes in tuning electronic properties. Compound 1c shows an even stronger bathochromic shift, with the lowest-energy maximum at 730 nm and six well-resolved absorption bands at 730, 668, 529, 447, 384 and 343 nm (Fig. 4c). The additional red shift is attributed to further π-extension from two additional fusions, again assigned to the HOMO–LUMO transition using TD-DFT calculations (Table S4). Compound 1d exhibits a similar absorption profile to 1c but with the lowest-energy maximum shifted further to 769 nm, also attributed to a HOMO–LUMO transition (Fig. 4d and Table S5).

The emission spectra of 1a–d show trends consistent with the absorption spectra (Fig. 4a–d). Compounds 1a-*rac* and 1a-*meso* display nearly identical fluorescence spectra with emission maxima at ∼599 nm and a broad shoulder extending into the far-red region (Fig. 4a), in line with their similar absorption features and frontier orbital distributions. With the structural modification from *peri*- to *bay*-fusion, 1b exhibits a red-shifted emission maximum at 659 nm, extending broadly across the visible-NIR range (Fig. 4b). Further π-extension in 1c leads to a more pronounced shift, with the emission maximum at 747 nm, again spanning a broad spectral window (Fig. 4c). These gradual bathochromic shifts mirror the absorption red-shifts and highlight the sensitivity of the excited-state properties to subtle structural changes. For 1d, an even larger red shift is observed, with the emission maximum at 786 nm (Fig. 4d). Unlike 1a–c, the spectrum of 1d exhibits narrowband emission extending from the far-red into the NIR region, with negligible visible-region contributions or shoulder peaks, reflecting its more extended π-conjugation and distinct helical framework. It is worth noting that these π-extended systems exhibit a certain sensitivity to light and thermal conditions (Fig. S21). Weak additional signals may appear on the high-energy side of the emission spectra upon exposure to ambient light or thermal treatment. These features are fully suppressed under strict exclusion of light and thermal input. This observation highlights the importance of careful control of light and temperature during spectroscopic measurements of large π-conjugated systems.

The absolute *Φ*_F_ of 1a–d, determined using an integrating sphere, are 65% (1a-*rac*), 59% (1a-*meso*), 24% (1b), and 7% (1c, 1d). These values reveal a general decrease in *Φ*_F_ as emission moves into the far-red to NIR region, consistent with the energy gap law,^34^ where smaller energy gaps enhance non-radiative decay through vibronic coupling. Nevertheless, compounds 1a–c retain relatively high *Φ*_F_ compared to other nanographenes with far-red to NIR emission,^35^ while 1d is noteworthy as a rare helical nanographene exhibiting narrowband emission spanning the far-red to NIR region with a full width at half maximum (FWHM) of 37 nm (compared to 70 nm for 1a-*rac*, 43 nm for 1b and 42 nm for 1c) and a respectable *Φ*_F_ of 7%, surpassing those of some molecular nanocarbon-based far-red to NIR fluorophores.^36^

Time-resolved fluorescence measurements reveal a clear trend of faster decay with increasing π-extension: the lifetimes decrease from 6.3 ns (1a-*rac*) and 6.2 ns (1a-*meso*) to 4.3 ns (1b) and 3.3 ns (1c), with 1d showing a slightly longer lifetime of 3.9 ns (Fig. S19). In parallel, the FWHM narrows progressively from 70 nm (1a) to 37 nm (1d), indicating increasingly well-defined emission profiles. This trend can be attributed to the enhanced structural rigidity and reduced conformational freedom associated with extended π-conjugation and fused frameworks. The increased rigidity suppresses vibronic coupling and limits excited-state geometric relaxation, thereby diminishing vibronic sidebands and resulting in narrower emission bands with improved spectral purity.^37^ These observations demonstrate how subtle variations in fusion topology and π-extension govern excited-state relaxation dynamics, thereby modulating both the efficiency and spectral purity of the emission.

Taken together with the absorption analysis, these results demonstrate that while the perylene pentamers share a common backbone, subtle isomeric variations and π-extension induce profound changes in their photophysical behaviour. The lowest-energy absorption and emission bands shift from 562/599 nm in 1a to 769/786 nm in 1d, corresponding to red shifts of 207 nm and 187 nm, respectively, accompanied by significant narrowing of emission bandwidth.

### Chiral properties

**Fig. 5:**
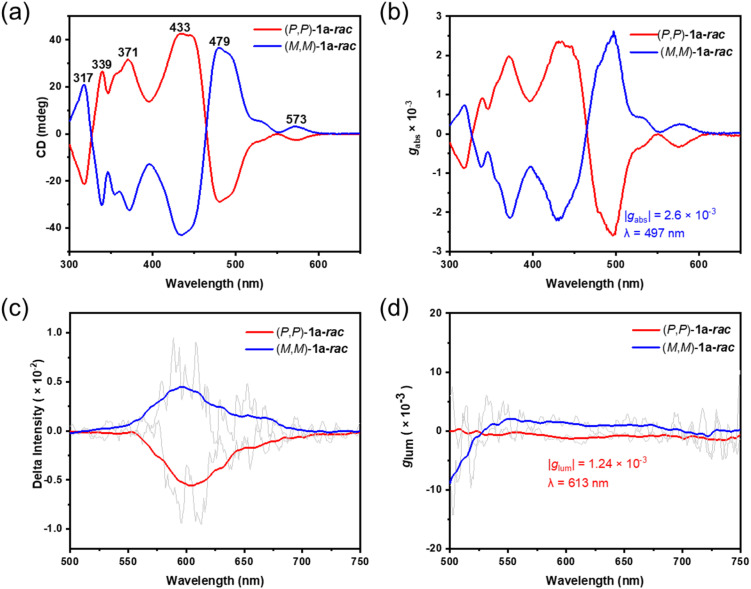
(a and b) Circular dichroism spectra of the enantiomers of 1a-*rac* measured in DCM. (c and d) Circularly polarized luminescence (CPL) spectra of enantiomers of 1a-*rac* in DCM (*c*: 1.2 × 10^−5^ M); the light gray line shows the original, unsmoothed data.

Successful chiral resolution of the perylene pentamers was achieved by chiral HPLC using an acetone/THF mixture as the eluent, affording enantiomerically pure 1a-*rac* and 1b–d (Fig. S22–S28). Owing to the rigid π-conjugated frameworks and the steric shielding from peripheral Ar groups, no racemization was observed even after 30 days at room temperature, confirming their exceptional configurational stability and high racemization barriers. The absolute configurations of the *P*- and *M*-enantiomers were assigned by comparing the experimental and simulated circular dichroism (CD) spectra. The CD spectra recorded in DCM (Fig. 5 and S26–S28) exhibit perfectly mirror-symmetric Cotton effects (CEs) over 300–800 nm for each enantiomeric pair. For instance, (*P*,*P*)-1a-*rac* displays a first positive CE at 339 nm and a first negative CE at 317 nm (Fig. 5a), followed by additional positive bands at 371 nm and 433 nm and negative bands at 479 nm and 573 nm, while its (*M*,*M*)-counterpart shows exact mirror symmetry. The simulated CD profiles reproduce both the sign and intensity of the experimental features, validating the configuration assignments and confirming the high chiroptical purity of all enantiomers. Across the series 1a–d, the CD spectra exhibit a systematic bathochromic shift of the lowest-energy CE, consistent with progressive π-extension and fusion topology modification, which mirrors the red-shift observed in the UV-vis absorption spectra. In particular, the chiroptical response gradually extends from the UV region (1a) toward the far-red region (1d), indicating effective modulation of electronic transitions through structural engineering (Fig. 5a and S26–S28). The absorption dissymmetry factors (|*g*_abs_|) were derived from the CD spectra, revealing that both the magnitude and wavelength of the maxima can be tuned systematically by subtle isomeric variation and π-extension. The maximum |*g*_abs_| values were determined to be 0.0026 at 497 nm (1a-*rac*), 0.0023 at 498 nm (1b), 0.0024 at 444 nm (1c), and 0.0042 at 594 nm (1d) (Fig. 5b and S26–28), indicating that progressive π-extension and topology modification effectively shift the chiroptical response from the UV-vis to the far-red region while enhancing |*g*_abs_| in the extended system. The CPL spectra of the enantiopure compound 1a-*rac* recorded in DCM (Fig. 5c and d) display characteristic mirror-image profiles corresponding to its fluorescence spectra. The (*P*,*P*)- and (*M*,*M*)-1a-*rac* pairs show distinct CPL signals with a maximum |*g*_lum_| of 1.24 × 10^−3^ at 613 nm. The CPL spectra of 1b–d were also measured in DCM, but exhibit very weaker CPL responses due to their emission extending into the far-red to NIR region, where reduced energy gaps lead to decreased radiative efficiency and consequently attenuated CPL intensity, in accordance with the energy-gap law.

To comprehensively assess CPL performance, the brightness of CPL (*B*_CPL_) was estimated using *B*_CPL_ = *ε* × *Φ* × |*g*_lum_|/2,^10^ where *ε* is the molar extinction coefficient at the excitation wavelength. For 1a-*rac*, the calculated *B*_CPL_ value is 66.7 M^−1^ cm^−1^, representing a significant improvement over compound 9^18^ (32 M^−1^ cm^−1^) and our previously reported perylene-based chiral nanographene^20^ (B, Fig. 1a), whose CPL signal was too weak to yield a reliable *g*_lum_ value. This enhancement is primarily attributed to the high molar absorptivity (*ε* = 165 420 M^−1^ cm^−1^ at 341 nm) resulting from the larger π-extension, highlighting the crucial role of π-expansion in strengthening chiroptical properties. In addition, the improved rigidity and symmetry of the unique fused perylene pentamer skeleton further contribute to the boosted CPL performance. The *B*_CPL_ value of 1a-*rac* therefore reflects an efficient balance between absorption strength, emission efficiency, and chiroptical activity, underscoring the importance of π-extension and structural rigidity in enhancing CPL performance.

### Theoretical analysis

**Fig. 6:**
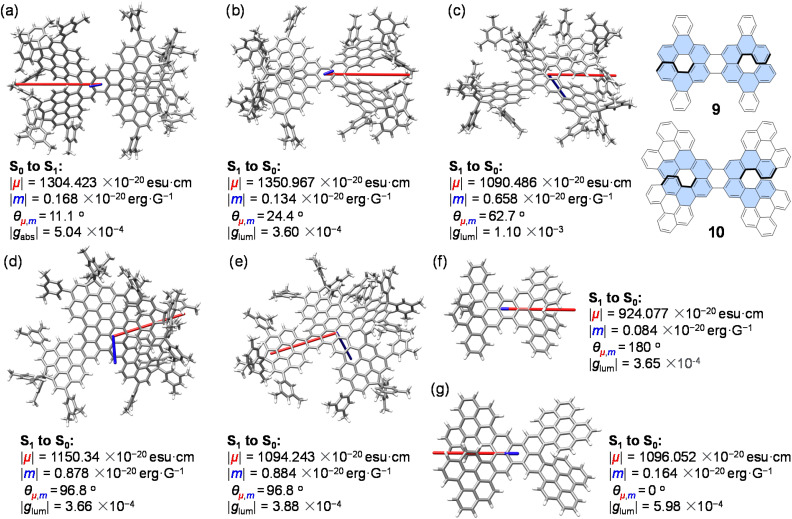
Calculated transition dipole moments for (a) S_0_ to S_1_ of 1a-*rac* and S_1_ to S_0_ electronic transitions of 1a-*rac* (b), 1b (c), 1c (d), 1d (e), 9 (f) and 10 (g). The *µ* vector is shown in red, and the *m* vector is shown in blue. The length of the *µ* vector is reduced 100 times when the length of the *m* vector is amplified 10 times for clarity. Calculated using TD-DFT at the B3LYP-D3-6-31G(d,p) level.

To elucidate the origins of the experimental |*g*_abs_| and |*g*_lum_| values, transition dipole moment (TDM) calculations were performed for the key absorption and emission transitions (Fig. 6). The dissymmetry factor can be expressed as *g* = 4 cos *θ*|*m*||*µ*|/(|*m*|^2^ + |*µ*|^2^), where *µ* and *m* are the electric and magnetic transition dipole moments and *θ* is the angle between them.^38^ For 1a-*rac*, the S_0_ → S_1_ transition shows *θ* = 11.1°, corresponding to nearly parallel alignment of *µ* and *m*, an outcome of its *D*_2_-symmetric, X-shaped π-skeleton (Fig. 6a), while the higher-energy S_0_ → S_7_ transition responsible for the maximum |*g*_abs_| exhibits *θ* = 175°, indicating an almost antiparallel orientation of *µ* and *m* (Fig. S50). Compared with the S_0_ → S_1_ transition of 1a-*rac*, the emissive S_1_ → S_0_ transition shows *θ* = 24.4°, suggesting a partial loss of ideal symmetry in the excited state (Fig. 6b). For 1b–d, the ground-state symmetry is reduced to *C*_1_, and the calculated *µ*–*m* angles for S_1_ → S_0_ transitions increase to 62.7°, 96.8°, and 96.8°, respectively (Fig. 6c–e). Despite these larger angles, the calculated |*g*_lum_| values are higher than that of 1a-*rac*. This apparent paradox arises from their smaller |*µ*| and larger |*m*|, yielding higher |*m*|/|*µ*| ratios, which dominate *g* according to the simplified expression *g* = 4 cos *θ*|*m*|/|*µ*|.^20^ Thus, fine-tuning the relative magnitudes of *m* and *µ* through isomeric topology control emerges as a critical strategy for optimizing chiroptical performance.

Comparative calculations for reference systems 9 and 10 further reinforce this design principle (Fig. 6f–g). Upon π-extension of the conjugated framework, both |*µ*| and |*m*| increase, but the enhancement in |*m*| is more pronounced, leading to a larger |*g*_lum_| for 10, despite its retention of *D*_2_ symmetry in both ground and excited states. Conversely, the Ar substituents in 1a-*rac* act as electron-donating groups, increasing |*µ*| and thus reducing the |*m*|/|*µ*| ratio, accounting for its smaller |*g*_lum_|.^39^ These insights highlight that while molecular symmetry is a key determinant of the *µ*–*m* alignment,^40^ electronic substituent effects and π-extension can modulate the balance between electric and magnetic contributions more effectively.

The chiroptical study reveals a clear structure–property relationship: (1) π-extension progressively red-shifts CD into the far-red region and emission into the far-red to NIR region, (2) |*g*_abs_| and |*g*_lum_| are governed by the |*m*|/|*µ*| ratio rather than symmetry alone, and (3) retaining high *Φ*_F_ and radiative efficiency remains essential for maximizing observable CPL brightness. These results offer molecular-level understanding of how topological variation, conjugation length, and symmetry interplay to dictate the chiroptical responses of helical nanographenes.

## Conclusions

In summary, a series of fully π-conjugated butterfly-like fused perylene pentamers (1a–d) with well-defined topological variations were successfully synthesized through a C–H transformation followed by a selective Scholl reaction, enabling systematic modulation of their photophysical and chiroptical properties. The rigid, helically twisted nanographene skeletons endow these compounds with exceptional configurational stability and strong absorption spanning from the UV-vis to the far-red to NIR region. Subtle isomeric variations and π-extension effectively tune their fluorescence color, quantum yield, and emission bandwidth, leading to a high quantum yield of up to 65% and narrowband emission spanning the far-red to NIR region with a FWHM of 37 nm. These results reveal a clear structure–property correlation governed by conjugation length and backbone symmetry.

Comprehensive studies of chiroptical properties further demonstrate that both the magnitude and spectral position of |*g*_abs_| and |*g*_lum_| can be precisely controlled by molecular topology. Transition dipole moment analyses reveal that the dissymmetry factors are predominantly governed by the balance between electric and magnetic transition dipole moments (|*m*|/|*µ*| ratio) rather than symmetry alone. Notably, 1a-*rac* achieves outstanding CPL brightness (66.7 M^−1^ cm^−1^) among nanographene emitters, highlighting the strong interplay between π-extension, electronic substitution, and radiative efficiency.

Overall, this integrated investigation of photophysical and chiroptical behaviors establishes a unified molecular design strategy for next-generation CPL emitters. By jointly optimizing conjugation topology, substituent electronics, and molecular symmetry, these findings pave the way toward high-brightness, CPL-active chiral nanographenes for advanced optoelectronic, sensing, and bioimaging applications.

## Author contributions

J. W., H.-B. Y. and Y. Z. supervised the project. Q. Z. synthesized and characterized the compounds. Z. S. and Z. Y. synthesized the starting materials. R. L., L. J. and W. F. performed crystallographic analysis. Q. Z. performed HPLC analysis. L.-T. B. performed circular dichroism and circularly polarized luminescence measurements. Q. Z. and R. L. carried out the calculations. Q. Z. wrote the manuscript. All authors discussed the results and contributed to the manuscript.

## Conflicts of interest

There are no conflicts to declare.

## Supplementary Material

SC-017-D6SC00676K-s001

SC-017-D6SC00676K-s002

## Data Availability

CCDC 2491985 (for 3), 2491986 (for 4), 2491987 (for 7), 2491988 (for 1a-*meso*), 2491989 (for 1b) and 2491990 (for 1c) contain the supplementary crystallographic data for this paper.^41*a*–*f*^ All data supporting this study are provided in the supplementary information (SI). Supplementary information: experimental procedures, compound characterization data, NMR and HRMS data, additional spectroscopic data (CD, absorption and emission spectra), HPLC traces, crystallographic data, and computational details. See DOI: https://doi.org/10.1039/d6sc00676k.
