# Supplementary material for: π-Aggregation-free, fused perylene pentamers: synthesis, narrowband far-red to near-infrared emission, and chiroptical properties
Source: Chem Sci. 2026 Apr 30;17(24):11892–902. doi: 10.1039/d6sc00676k (PMC13154877; doi:10.1039/d6sc00676k)
Supplement: SC-017-D6SC00676K-s001 [file SC-017-D6SC00676K-s001.pdf]

Supplementary Information (SI) for Chemical Science.
This journal is © The Royal Society of Chemistry 2026

Supporting Information

Table of Contents

1. Experimental section……………………………………………………….…......S2

1.1 General…………………………………………………………..……......S2

1.2 Synthetic procedures and characterization data………………………...…....S3

2. Additional spectra and data……...…………………...……...…….….……S11

3. Chiral HPLC analysis and chiroptical spectra……….………………………S21

4. DFT calculations……………………………………………………….....S24

5. X-ray crystallographic analysis………………………………….................S39

6. References..………………………………………………………………....S48

7. Appendix I: NMR and HR mass spectra of all new compounds…………….......S50

8. Appendix II: Cartesian Coordinates of optimized structures……….…………..S70

1. Experimental Section

1.1 General

The starting materials and all reagents were obtained from commercial suppliers and used without further purification unless otherwise noted. Compounds **2**[1] and **5**[2] were synthesized according to the procedures reported in the literature. Unless otherwise noted, all reactions were performed under an argon atmosphere in oven-dried glassware with standard vacuum-line techniques. All work-up and purification procedures were carried out with reagent-grade solvents in the air.

NMR spectra were recorded in deuterated solvents on a Bruker AVANCE 400 NMR Spectrometer (^{1}H 400 MHz, ^{13}C 100 MHz), a Bruker AVIII 500WB NMR Spectrometer (^{1}H 500 MHz, ^{13}C 125 MHz) or a Bruker AVNEO 600 NMR Spectrometer (^{1}H 600 MHz, ^{13}C 150 MHz). All chemical shifts are quoted in ppm, relative to the signals corresponding to the residual non-deuterated solvents CH_2Cl_2 (δ_H = 5.32 ppm, δ_C = 53.8 ppm) or C_6H_6 (δ_H = 7.16 ppm, δ_C = 128.1 ppm). Coupling constants (*J*) are given in Hz and the following abbreviations have been used to describe the signals: singlet (s); doublet (d); doublet of doublets (dd); triplet (t); multiplet (m). High-resolution mass (HR-MS) spectra were recorded on an Agilent 6546 Q-TOF LCMS instrument (for APCI) or a JEOL JMS-S3000 Sprial-TOF MS instrument (for MALDI).

Absorption spectra were recorded on a Shimadzu UV-3600 plus UV-Vis-NIR spectrophotometer and time-resolved fluorescence spectroscopic measurements were conducted on a Shimadzu RF-5301PC spectro fluorophotometer. The absolute quantum yields were recorded on a JASCO model FP-8550 spectrofluorometer, equipped with an integrating sphere while fluorescence lifetime measurements were conducted on a Hamamatsu model compact fluorescence lifetime spectrometer C11367 (Quantaurus-Tau).

Chiral HPLC isolations were conducted on a Shimadzu HPLC System (LC-20AP) equipped with a COSMOSIL Cholester column (2(i.d.) × 25 cm) at room temperature. Circular Dichroism (CD) and Circularly Polarized Luminescence (CPL) spectra were measured on a Chirascan Series Spectrometer (Applied Photophysics Ltd, UK) at room temperature with 10 × 10 mm quartz cells. The following are parameter settings: PMT/1000 V, scan speed 1 nm/s, slit width 20 nm, error range: ±5%.

1.2. Synthetic procedures and characterization data

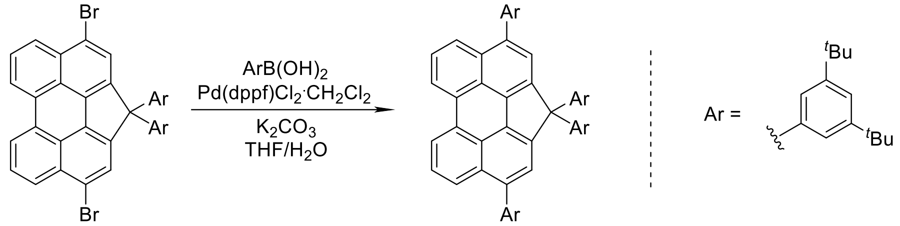

2 **3**

Synthesis of 3

To a mixture of compound **2**[1] (483 mg, 0.6 mmol), (3,5-di-*tert*-butylphenyl)boronic acid (568 mg, 2.4 mmol), Pd(dppf)Cl_2·CH_2Cl_2 (49 mg, 0.06 mmol), and K_2CO_3 (497 mg, 3.6 mmol) under an argon atmosphere were added THF (20 mL) and H_2O (12 mL), both pre-degassed by bubbling with argon for 20 minutes. The reaction flask was then sealed, and the mixture was heated to 80 °C and stirred overnight. After completion, the reaction mixture was concentrated under reduced pressure. The residue was diluted with DCM and washed with brine. The organic layer was dried over anhydrous Na_2SO_4, filtered, and concentrated under reduced pressure. The crude product was purified by column chromatography on silica gel using DCM/hexane (1:20) as eluent, affording the desired compound **3** (414.8 mg, 68%) as a yellow solid.

Compound 3: ^{1}H NMR (400 MHz, CD_2Cl_2, 298 K): δ ppm 8.22 (d, *J* = 7.0 Hz, 2H), 7.79 (d, *J* = 8.1 Hz, 2H), 7.58 (s, 2H), 7.56-7.52 (m, 2H), 7.50 (s, 2H), 7.40 (d, *J* = 1.4 Hz, 4H), 7.30 (t, *J* = 1.6 Hz, 2H), 7.24 (d, *J* = 1.7 Hz, 4H), 1.38 (s, 36H), 1.20 (s, 36H). ^{13}C NMR (100 MHz, CD_2Cl_2, 298 K): 151.2, 150.8, 142.8, 135.3, 133.2, 127.9, 126.6, 126.0, 124.7, 122.8, 121.4, 121.1, 35.3, 35.2, 31.7, 31.6. HRMS analysis (APCI, *m*/*z*) [$(M+H)^+$] calcd for $C_{77}H_{93}$: 1017.7272, found 1017.7278 (error: 0.59 ppm).

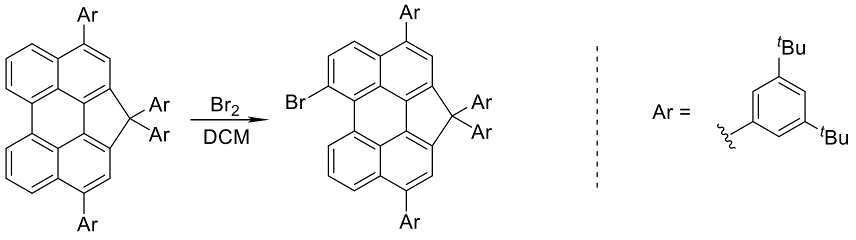

3 **4**

Synthesis of 4

To a solution of **3** (341.4 mg, 0.34 mmol) in analytical grade DCM (50 mL), bromine (0.02 mL, 0.4 mmol) was added. The reaction mixture turned deep green without obvious fluorescence upon addition. The mixture was stirred at room temperature for one hour, during

which it exhibited slight fluorescence. Subsequently, the reaction mixture was poured into aq. $Na_2S_2O_3$ (20 ml), resulting in a yellow solution with bright fluorescence. The organic phase was separated and washed with brine (50 ml), then dried over Na_2SO_4. After solvent evaporation under vacuum, the residue was purified by column chromatography on silica gel using hexane as the eluent, yielding the desired product **4** (297.7 mg, 81%) as a yellow solid.

Compound 4: ^{1}H NMR (400 MHz, CD_2Cl_2, 298 K): δ ppm 9.64 (d, *J* = 7.8 Hz, 1H), 7.89 (d, *J* = 8.3 Hz, 1H), 7.75 (d, *J* = 9.1 Hz, 1H), 7.61 (t, *J* = 7.4 Hz, 4H), 7.51 (d, *J* = 1.5 Hz, 2H), 7.38 (dd, *J* = 13.5, 1.7 Hz, 4H), 7.30 (t, *J* = 1.7 Hz, 2H), 7.21 (d, *J* = 1.7 Hz, 4H), 1.38 (d, *J* = 3.0 Hz, 36H), 1.20 (s, 36H). ^{13}C NMR (100 MHz, CD_2Cl_2, 298 K): 151.3, 151.2, 150.9, 147.0, 145.9, 142.6, 140.8, 140.5, 140.3, 135.8, 134.5, 134.3, 132.5, 132.4, 131.5, 131.1, 128.9, 127.3, 127.2, 126.4, 126.0, 125.7, 124.9, 124.8, 122.9, 121.6, 121.5, 121.2, 119.3 35.3, 35.2, 31.7, 31.7, 31.6. HRMS analysis (APCI, *m/z*) [$(M+H)^+$] calcd for $C_{77}H_{92}Br$: 1095.6377, found 1095.6374 (error: 0.27 ppm).

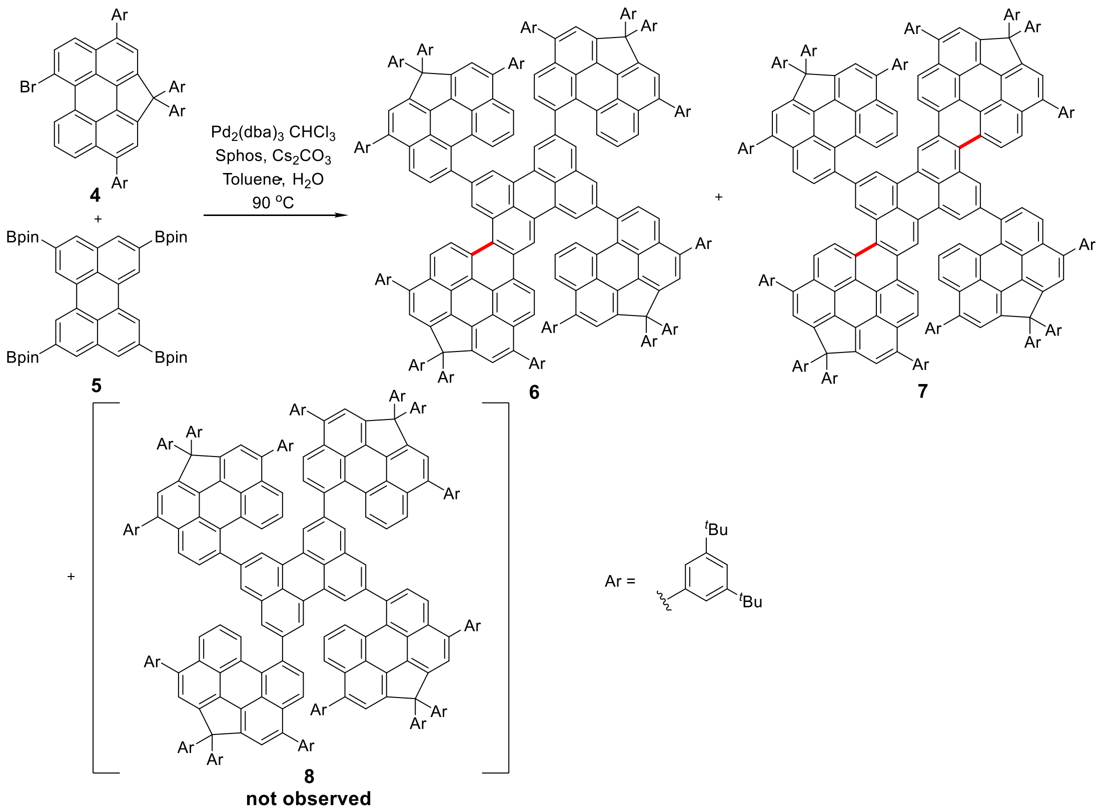

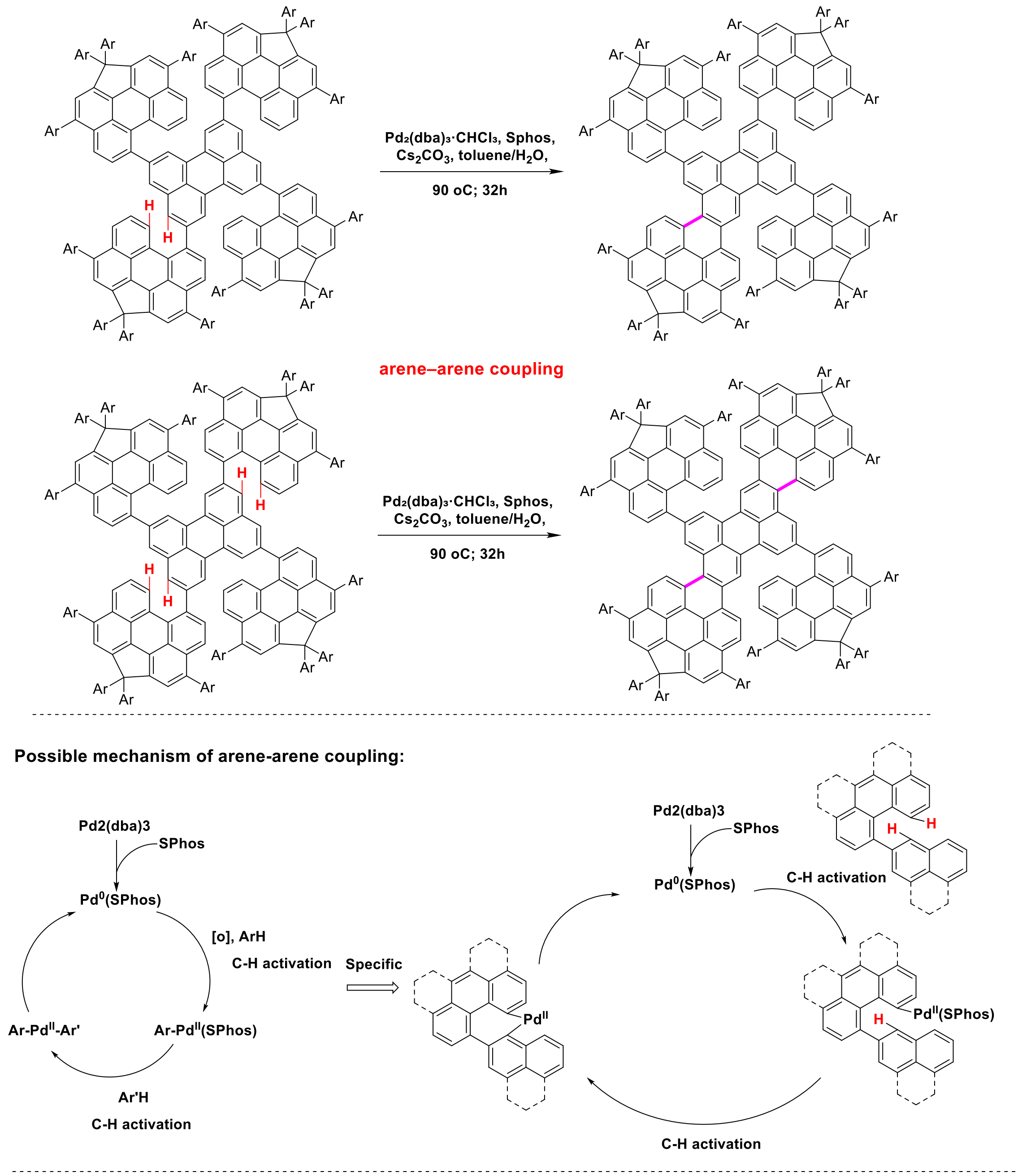

Fig. S1. Possible C-H activation procedure for **6** and **7**. **Possible mechanism:** the catalytic cycle is initiated by ligand exchange of $Pd_2(dba)_3$ with SPhos, generating an active low-coordinate Pd(0)(SPhos) species. Subsequently, C–H activation occurs via a concerted metalation–deprotonation pathway, in which Cs_2CO_3 assists deprotonation to form a Pd(II)–aryl intermediate. A second C–H activation at a proximal aryl position generates a diaryl–Pd(II) species, which then undergoes reductive elimination to form the new C–C bond, leading to intramolecular cyclization and formation of the observed pentameric frameworks **6** and **7**. Finally, Pd(0)(SPhos) is regenerated, completing the catalytic cycle.

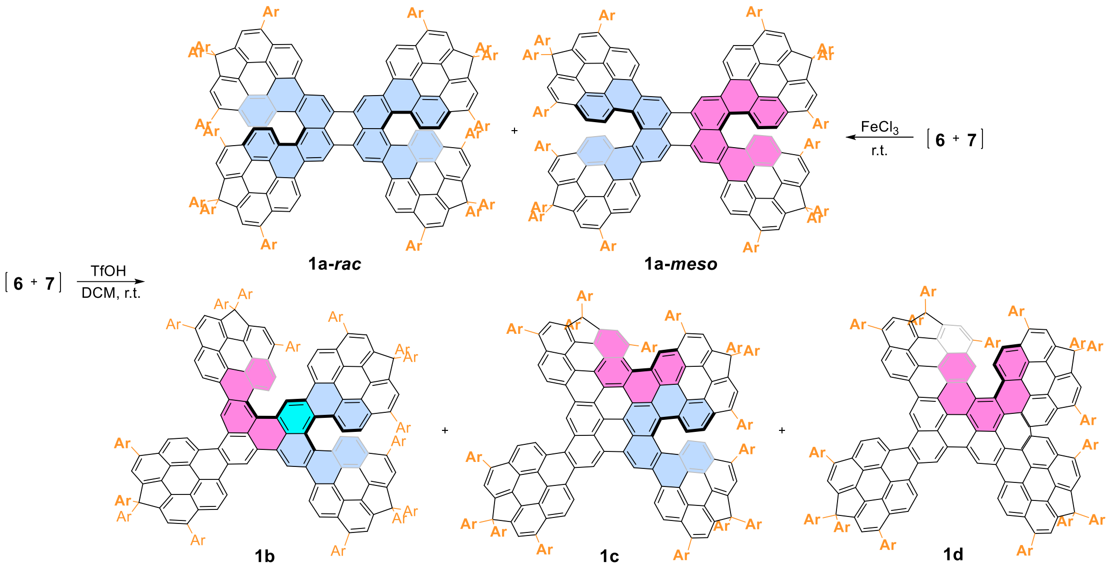

Synthesis of perylene pentamers

To a Schlenk flask under an argon atmosphere were added compound **4** (1.09 g, 1.0 mmol), compound **5** (75.6 mg, 0.1 mmol), $Pd_2(dba)_3$ (41.4 mg, 0.04 mmol), SPhos (32.8 mg, 0.08 mmol), and Cs_2CO_3 (521.3 mg, 1.6 mmol). The flask was evacuated and backfilled with argon three times, after which degassed toluene/H_2O (20 mL/10 mL) was injected. The reaction mixture was heated to 90 °C and stirred for 32 h. After cooling to room temperature, water was added, and the mixture was extracted with DCM (3×). The combined organic layers were washed with brine, dried over anhydrous Na_2SO_4, and the solvent was removed under reduced pressure. The crude product was first purified by short column chromatography (silica gel, DCM as eluent) to remove catalysts, followed by preparative GPC ($CHCl_3$, 14 mL/min) to afford a mixture of compounds **6** and **7** as an orange-yellow solid (99.1 mg, 23%). Compound **8** could not be isolated, while the unexpected formation of **6** and **7** did not affect the subsequent Scholl reaction toward the target compounds; thus, optimization of **8** was not pursued. Due to their similar polarity and the fact that both intermediates can lead to the desired pentamers, the mixture was used directly in the next step without further purification. A small portion of the mixture was subjected to preparative TLC (DCM/Hexane = 1/15), allowing characterization of compound **6** by HRMS and compound **7** by both HRMS and X-ray analysis. **Compound 6:** HRMS (MALDI, 100%, *m*/z) [M^+] calcd for $C_{328}H_{370}$: 4311.9049; found: 4311.5843. **Compound 7:** HRMS (MALDI, *m*/*z*) [M^+] calcd for $C_{328}H_{368}$: 4306.8791; found: 4306.8715 (error: -1.76 ppm).

Method I for Scholl reaction: To a Schlenk flask under an argon atmosphere was added the mixture of compounds **6** and **7** (88 mg, 0.02 mmol), DDQ (23 mg, 0.1 mmol), and DCM (200 mL). The solution was degassed by bubbling argon for 20 min, after which TfOH (0.26

mL, 2.9 mmol) was added dropwise at room temperature. The reaction mixture was stirred for 1.5 h at room temperature, then quenched with Et_3N (3.0 mL) and H_2O (50 mL) and stirred for an additional 15 min. The resulting mixture was extracted with DCM/H_2O, and the combined organic layers were washed sequentially with saturated aqueous $NaHCO_3$, H_2O, and brine, dried over Na_2SO_4. The solvent was removed under reduced pressure. The crude product was first purified by preparative GPC (THF, 18 mL/min), which showed only a single peak due to the structural similarity of the final products (Fig. S2). The collected fraction was concentrated and further purified by preparative TLC (hexane/DCM = 15:1), affording four main bands corresponding to crude **1a–1d**. These fractions were not completely separated, with neighboring bands overlapping owing to their similar polarity (Fig. S3). The crude **1a–1d** fractions were subsequently subjected to preparative HPLC (THF/acetone = 20:80, 8 mL/min) using a COSMOSIL Cholester column (2 i.d. × 25 cm) to afford relatively pure **1a–1d**. Each was further purified by preparative TLC (hexane/DCM = 15:1), yielding: **1a** (17.6 mg, 20%) as a red solid, **1b** (16.6 mg, 18.9%) as a black-green solid, **1c** (22.5 mg, 25.6%) as a dark solid, and **1d** (9.0 mg, 10.2%) as a brown-red solid. Compound **1a** was found to consist of a mixture of the **1a-*rac*** and **1a-*meso*** isomers. Recrystallization from THF/acetone (1:9) afforded **1a-*meso*** as the solid, while **1a-*rac*** remained in the mother liquor and was further purified by HPLC on a COSMOSIL Cholester column to give pure **1a-*rac***.

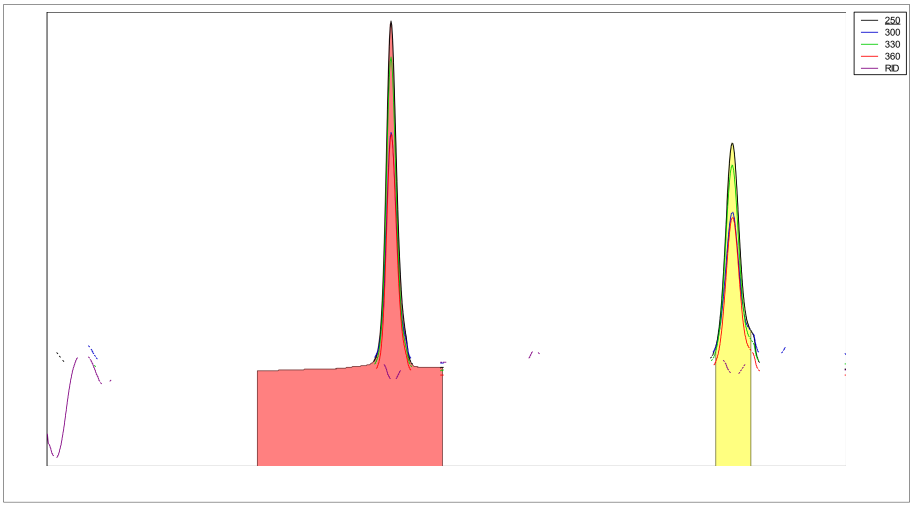

Fig. S2. GPC curve of the final products **1a-1d**. Shimazu GPC column K802 (8.0mm id x 30cm), SPD-20AV UV-Vis spectrophotometric detector, and chloroform as eluent.

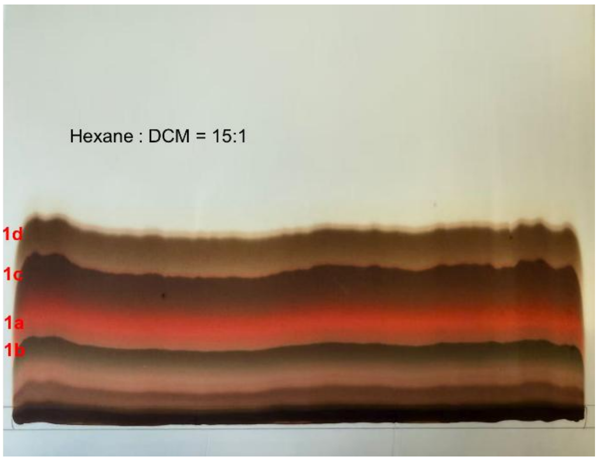

Fig. S3. PTLC results of the final products **1a-1d**.

Method II for Scholl reaction: To a Schlenk flask under an argon atmosphere was added the mixture of compounds **6** and **7** (20 mg, 0.0046 mmol) in DCM (40 mL). The solution was purged with argon for 15 min, after which a solution of $FeCl_3$ in $MeNO_2$ (103 mg, 0.4 mL) was added dropwise at room temperature. The resulting black solution was stirred at room temperature for 1 h under a continuous argon flow. The reaction progress was monitored by TLC. The mixture was then quenched with methanol (1 mL), extracted with DCM/H_2O, and the combined organic layers were dried over Na_2SO_4. The solvent was removed under reduced pressure. The crude product was purified by preparative GPC (THF, 18 mL/min) followed by a short silica gel column (hexane/DCM = 20:1), affording pure **1a** (11.4 mg, 57%) as a red solid. The **1a-*rac*** and **1a-*meso*** isomers were separated using the same procedure described in Method I.

Compound 1a: ^{1}H NMR (400 MHz, CD_2Cl_2, 298 K): δ ppm 10.86 (s, 2H), 10.74 (s, 2H), 9.75 (d, *J* =, 9.8 Hz, 2H), 9.68 (d, *J* = 9.7 Hz, 2H), 8.73 (d, *J* = 9.2 Hz, 2H), 8.67 (d, *J* = 9.2 Hz, 2H), 8.19 – 8.15 (m, 8H), 8.09 (dd, *J* = 9.6 Hz, 5.2 Hz, 4H), 7.78 – 7.73 (m, 16H), 7.70 – 7.67 (m, 4H), 7.44 (s, 4H), 7.33 (d, *J*= 9.6 Hz, 4H), 7.19 – 7.17 (m, 8H), 7.13 (d, *J* = 1.3 Hz, 8H), 7.07 (dd, *J* = 6.2 Hz, 1.7 Hz, 8H), 1.55 (s, 36H), 1.53 (s, 36H), 1.33 (s, 144H), 1.08 (s, 36H), 1.06 (s, 36H). ^{13}C NMR (150 MHz, CD_2Cl_2, 298 K): 151.5, 151.4, 151.0, 150.8, 150.7, 147.3, 146.9, 146.8, 144.6, 142.9, 141.2, 140.6, 140.0, 139.8, 135.5, 135.2, 129.5, 129.4, 129.2, 129.0, 128.9, 128.8, 128.3, 128.2, 128.1, 128.0, 127.7, 127.6, 127.2, 127.1, 126.8, 126.7, 126.6, 126.5, 125.4, 124.6, 124.3, 124.2, 123.9, 123.8, 122.7, 121.7, 121.6, 121.4, 120.8, 120.6, 120.1, 119.2, 35.5, 35.4, 35.1, 32.4, 31.9, 31.8, 31.5, 30.5, 30.4, 30.1, 29.8. HRMS (MALDI, m/z) [M^+] calcd for $C_{328}H_{364}$: 4302.8478; found: 4302.8394 (error: -1.95 ppm).

Compound 1a-*rac*: ^{1}H NMR (500 MHz, CD_2Cl_2, 298 K) δ ppm 10.86 (s, 4H), 9.74 (d, *J* = 9.7 Hz, 4H), 8.72 (d, *J* = 9.2 Hz, 4H), 8.19 (s, 4H), 8.15 (s, 4H), 8.09 (d, *J* = 9.6 Hz, 4H), 7.76 (d, *J* = 4.8 Hz, 16H), 7.69 (s, 4H), 7.44 (s, 4H), 7.33 (d, *J* = 9.6 Hz, 4H), 7.11 (dd, *J* = 41.1 Hz,

17.4 Hz, 24H), 1.55 (s, 124H), 1.32 (s, 72H), 1.07 (s, 72H). ^{1}H NMR (500 MHz, C_6D_6, 298 K) δ ppm 10.52 (s, 4H), 9.37 (d, *J* = 9.9 Hz, 4H), 8.79 – 8.75 (m, 12H), 8.54 (d, *J* = 9.6 Hz, 4H), 8.25 (s, 8H), 7.87 (s, 8H), 7.76 (d, *J* = 9.7 Hz, 8H), 7.70 (d, *J* = 1.6 Hz, 12H), 7.61 (s, 8H), 7.39 (d, *J* = 11.4 Hz, 8H), 1.57 (s, 72H), 1.35 (s, 124H), 1.12 (s, 72H). HRMS (MALDI, *m*/*z*) [M^+] calcd for $C_{328}H_{364}$: 4302.8478; found: 4302.8376 (error: -2.37 ppm).

Compound 1a-*meso*: ^{1}H NMR (500 MHz, CD_2Cl_2, 298 K) δ ppm 10.73 (s, 4H), 9.68 (d, *J* = 9.5 Hz, 4H), 8.66 (d, *J* = 9.1 Hz, 4H), 8.16 (d, *J* = 13,6 Hz, 8H), 8.08 (d, *J* = 9.5 Hz, 4H), 7.80 – 7.64 (m, 20H), 7.44 (s, 4H), 7.33 (d, *J* = 9.5 Hz, 4H), 7.27 – 7.01 (m, 24H), 1.53 (d, *J* = 4.7 Hz, 124H), 1.32 (s, 72H), 1.06 (s, 72H). ^{13}C NMR (150 MHz, CD_2Cl_2, 298K): 151.4, 150.9, 150.8, 150.6, 147.3, 146.8, 144.6, 142.8, 141.1, 140.5, 140.0, 139.8, 135.4, 135.1, 129.5, 129.3, 129.1, 128.7, 128.1, 128.0, 127.5, 127.2, 126.9, 126.6, 126.5, 125.3, 124.4, 124.3, 123.9, 123.7, 122.6, 121.6, 121.4, 120.7, 120.5, 120.1, 35.4, 35.3, 35.0, 31.9, 31.7, 31.4, 31.2, 31.0. HRMS (MALDI, *m*/*z*) [M^+] calcd for $C_{328}H_{364}$: 4302.8478; found: 4302.8255 (error: -5.18 ppm).

Compound 1b: ^{1}H NMR (600 MHz, C_6D_6, 298 K) δ ppm 11.05 (s, 1H), 10.81 (s, 1H), 10.25 (s, 1H), 9.99 (s, 1H), 9.49 (d, *J* = 9.3 Hz, 1H), 9.36 (d, *J* = 9.9 Hz, 1H), 9.22 (d, *J* = 9.9 Hz, 1H), 9.02 (dd, *J* = 21.9, 9.7 Hz, 2H), 8.95 (s, 1H), 8.88 (s, 1H), 8.85 – 8.77 (m, 6H), 8.73 (s, 1H), 8.69 (d, *J* = 8.8 Hz, 1H), 8.67 – 8.59 (m, 4H), 8.52 – 8.47 (m, 1H), 8.46 (s, 1H), 8.31 (d, *J* = 1.5 Hz, 2H), 8.26 (d, *J* = 9.6 Hz, 1H), 8.22 (d, *J* = 9.1 Hz, 1H), 8.19 (dd, *J* =6.8, 1.7 Hz, 4H), 8.17 (d, *J* = 1.6 Hz, 2H), 8.06 (d, *J* = 11.1 Hz, 3H), 7.94 (d, *J* = 1.7 Hz, 2H), 7.91 (s, 2H), 7.89 (d, *J* = 1.7 Hz, 4H), 7.83 (d, *J* = 1.6 Hz, 4H), 7.82 – 7.81 (m, 2H), 7.80 (s, 1H), 7.77 – 7.74 (m, 4H), 7.68 (d, *J* = 1.6 Hz, 2H), 7.66 (d, *J* = 2.8 Hz, 1H), 7.65 – 7.61 (m, 5H), 7.59 – 7.57 (m, 3H), 7.51 – 7.50 (m, 1H), 7.50 – 7.48 (m, 1H), 7.48 – 7.46 (m, 1H), 7.46 – 7.43 (m, 2H), 7.42 – 7.40 (m, 1H), 7.36 – 7.34 (m, 1H), 7.22 – 7.20 (m, 1H), 1.53 (d, *J* = 4.2 Hz, 42H), 1.47 (s, 18H), 1.40 (d, *J* = 2.7 Hz, 36H), 1.36 (s, 24H), 1.32 (d, *J* = 8.2 Hz, 72H), 1.27 (s, 24H), 1.23 (s, 24H), 1.18 (s, 24H), 1.10 (s, 24H). HRMS (MALDI, *m*/*z*) [M^+] calcd for $C_{328}H_{364}$: 4302.8478; found: 4302.8296 (error: -4.23 ppm).

Compound 1c: ^{1}H NMR (600 MHz, C_6D_6, 297 K) δ ppm 11.52 (s, 1H), 11.17 (s, 1H), 10.90 (s, 1H), 10.20 (s, 1H), 9.84 (d, *J* = 9.3 Hz, 1H), 9.63 (d, *J* = 10.0 Hz, 1H), 9.51 (d, *J* = 9.7 Hz, 1H), 9.45 (d, *J* = 9.7 Hz, 1H), 9.08 (s, 1H), 9.03 (d, *J* = 9.3 Hz, 1H), 8.92 – 8.84 (m, 6H), 8.81 – 8.78 (m, 3H), 8.74 (d, *J* = 9.2 Hz, 1H), 8.70 (s, 1H), 8.59 (d, *J* = 8.7 Hz, 1H), 8.52 (s, 2H), 8.40 (s, 2H), 8.29 (s, 2H), 8.06 – 8.02 (m, 8H), 7.96 – 7.92 (m, 6H), 7.88 (d, *J* = 7.4 Hz, 3H), 7.81 – 7.79 (m, 8H), 7.71 (s, 1H), 7.63 (d, *J* = 11.2 Hz, 2H), 7.58 (d, *J* = 7.3 Hz, 2H), 7.54 – 7.50 (m, 3H), 7.43 (dd, *J* = 16.9 Hz, 7.9 Hz, 4H), 7.29 (s, 1H), 7.20 (d, *J* = 14.2 Hz, 5H), 7.03 (s, 1H), 1.67 – 1.48 (m, 126H), 1.39 (s, 18H), 1.24 (d, *J* = 8.4 Hz, 54H), 1.15 (s, 18H), 1.01 (s, 18H), 0.85 (s, 18H), 0.59 (s, 9H), 0.43 (s, 27H). ^{13}C NMR (150 MHz, C_6D_6, 298 K): 151.8, 151.4, 151.3, 151.1, 150.9, 150.7, 150.4, 149.5, 148.7, 147.9, 147.8, 147.7, 147.0, 146.6, 145.7, 145.6, 145.0, 144.7, 144.3, 144.1, 143.8, 143.6, 143.4, 142.9, 142.1,

142.0, 141.9, 141.7, 141.6, 141.3, 141.1, 140.8, 140.6, 140.5, 139.1, 137.0, 136.3, 136.1, 136.0, 135.6, 135.1, 132.5, 131.0, 130.7, 130.4, 130.3, 130.2, 129.9, 129.8, 129.7, 129.5, 129.2, 129.0, 128.8, 128.7, 128.5, 127.7, 127.6, 127.5, 127.2, 127.0, 126.7, 126.5, 126.4, 126.1, 125.9, 125.6, 125.2, 125.0, 124.9, 124.7, 124.5, 124.2, 124.1, 123.8, 123.6, 123.5, 123.3, 123.2, 123.0, 122.9, 122.8, 121.5, 121.3, 121.0, 120.8, 120.6, 120.5, 119.3, 118.2, 35.6, 35.5, 35.4, 35.3, 35.2, 35.1, 34.9, 34.8, 34.4, 32.8, 32.4, 32.2, 32.1, 32.0, 31.8, 31.7, 31.6, 31.5, 31.4, 31.2, 31.0, 30.3, 30.2, 30.1, 30.0, 29.9, 29.8, 29.7, 27.8. HRMS (MALDI, *m/z*) [M^+] calcd for $C_{328}H_{360}$: 4298.8165; found: 4298.8234 (error: 1.61 ppm).

Compound 1d: ^{1}H NMR (600 MHz, C_6D_6, 298 K) δ ppm 12.32 (s, 1H), 11.93 (s, 1H), 10.66 (d, J = 9.1 Hz, 1H), 10.56 (s, 1H), 10.53 (s, 1H), 9.90 (d, J = 8.8 Hz, 1H), 9.68 (dd, J = 21.9 Hz, 9.4 Hz, 2H), 9.17 (s, 1H), 9.05 (d, J = 9.2 Hz, 1H), 8.98 – 8.85 (m, 5H), 8.76 (d, J = 11.0 Hz, 4H), 8.71 (d, J = 8.7 Hz, 1H), 8.66 (s, 1H), 8.46 (d, J = 16.6 Hz, 4H), 8.36 (s, 2H), 8.29 (s, 2H), 8.22 (s, 4H), 8.13 (s, 2H), 8.08 (d, J = 9.4 Hz, 1H), 7.98 (d, J = 44.5 Hz, 4H), 7.90 – 7.85 (m, 4H), 7.85 – 7.77 (m, 4H), 7.74 (s, 3H), 7.69 (s, 1H), 7.64 (d, J = 12.0 Hz, 4H), 7.58 (s, 1H), 7.55 (d, J = 8.8 Hz, 5H), 7.40 (s, 2H), 7.34 (s, 1H), 7.31 (d, J = 7.2 Hz, 2H), 7.23 (s, 1H), 7.07 (s, 1H), 6.84 (s, 1H), 1.65 (d, J = 4.9 Hz, 36H), 1.55 (d, J = 23.9 Hz, 90H), 1.48 (s, 18H), 1.34 (s, 54H), 1.22 (s, 18H), 1.03 (s, 18H), 1.01 (s, 18H), 0.98 (s, 18H), 0.95 (s, 18H). ^{13}C NMR (150 MHz, C_6D_6, 298 K): 151.5, 151.4, 151.3, 151.2, 151.1, 151.0, 150.0, 149.7, 149.6, 149.4, 149.2, 149.1, 149.0, 148.9, 148.7, 148.6, 148.5, 147.9, 147.7, 146.4, 146.1, 146.0, 145.3, 145.2, 145.1, 144.6, 144.4, 144.3, 144.2, 144.1, 143.8, 143.7, 143.0, 142.9, 142.5, 142.2, 142.1, 142.0, 141.9, 141.8, 141.6, 141.0, 140.7, 140.5, 140.2, 139.8, 139.6, 139.4, 139.1, 136.5, 136.3, 136.1, 135.6, 135.5, 135.4, 135.0, 134.7, 133.5, 132.7, 132.1, 131.2, 131.1, 131.0, 130.8, 130.7, 130.5, 130.3, 130.0, 129.5, 129.3, 129.2, 129.0, 127.2, 126.8, 126.6, 126.5, 126.1, 126.0, 125.8, 125.7, 125.6, 125.4, 125.3,125.2, 125.0,124.8, 124.7, 124.6, 124.5, 124.4,124.3, 124.2, 123.5, 123.4, 123.2, 122.8, 122.3, 121.7, 121.6, 121.5, 121.4, 121.2, 121.1, 121.0, 35.6, 35.5, 35.4, 35.3, 35.2, 35.1, 35.0, 34.9, 34.5, 32.4, 32.1, 32.0, 31.9, 31.8, 31.6, 31.5, 31.4, 31.3, 30.8, 30.3, 30.2, 30.0, 29.9, 29.8, 27.8. HRMS (MALDI, *m/z*) [M^+] calcd for $C_{328}H_{358}$: 4296.8008; found: 4296.8653 (error: 15.01 ppm).

2. Additional spectra and data

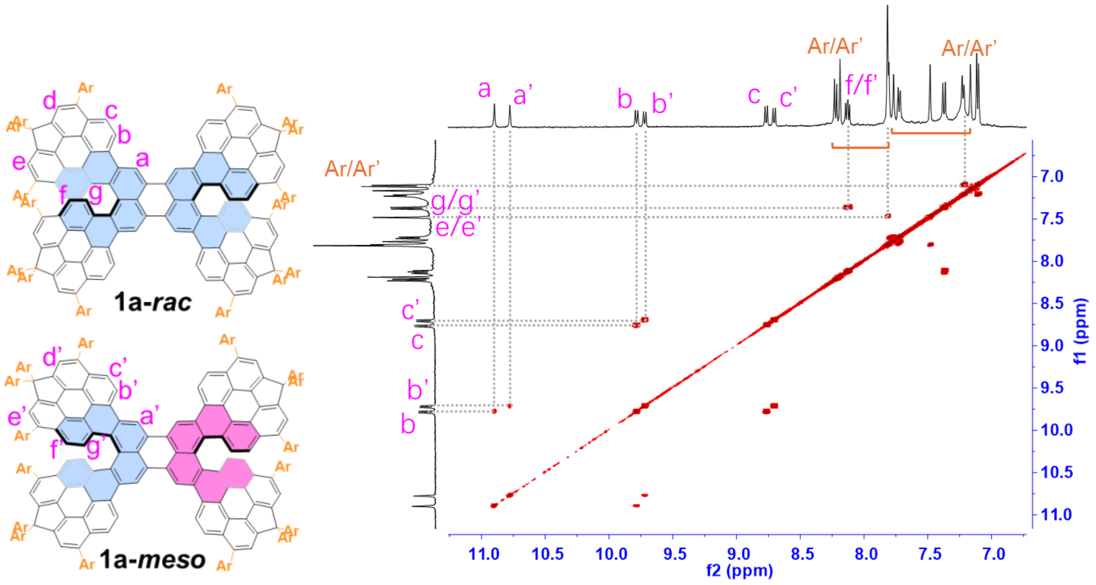

Fig. S4. Partial 2D COSY NMR spectrum of **1a** in CD_2Cl_2 (500 MHz) with assignment.

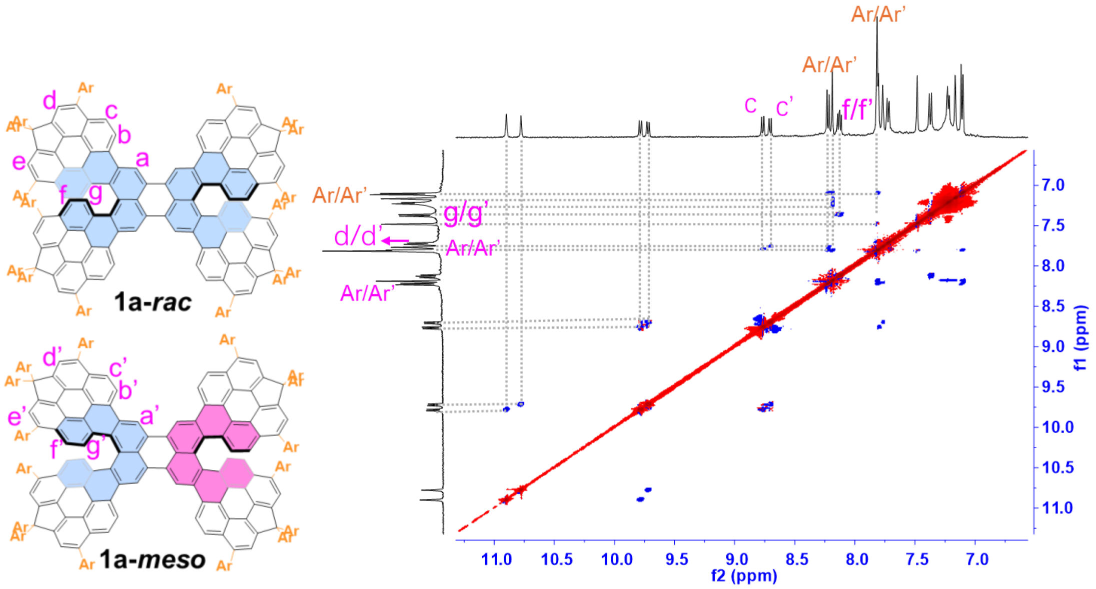

Fig. S5. Partial 2D ROESY NMR spectrum of **1a** in CD_2Cl_2 (500 MHz) with assignment.

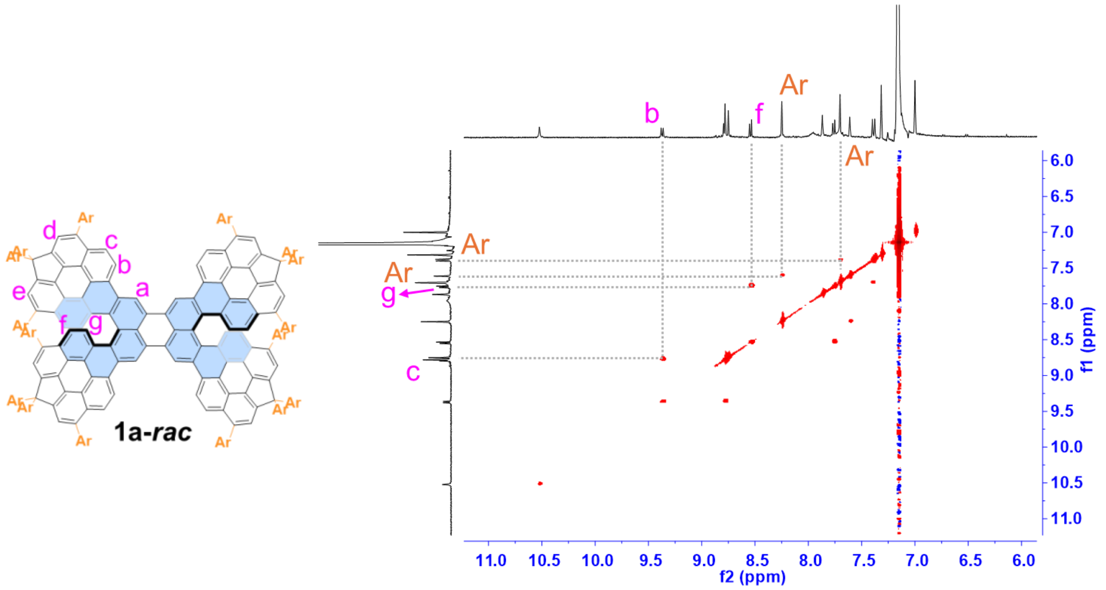

Fig. S6. Partial 2D COSY NMR spectrum of **1a-*rac*** in C_6D_6 (500 MHz) with assignment.

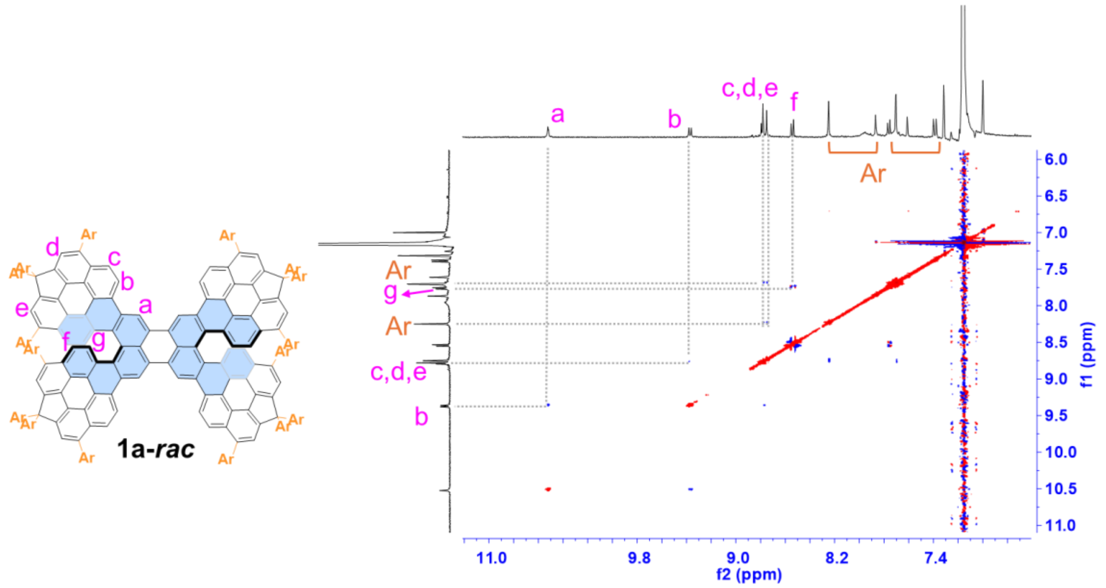

Fig. S7. Partial 2D ROESY NMR spectrum of **1a-*rac*** in C_6D_6 (500 MHz) with assignment.

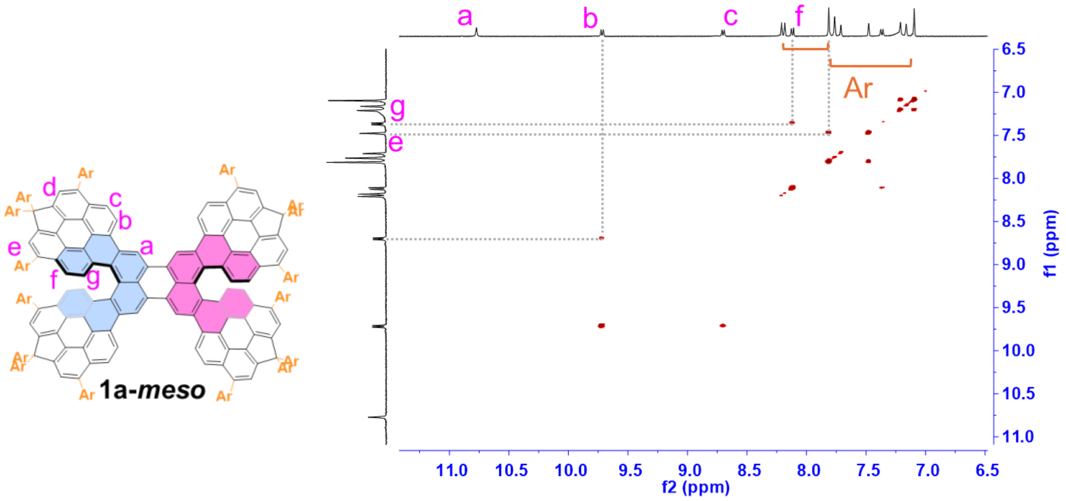

Fig. S8. Partial 2D COSY NMR spectrum of **1a-*meso*** in CD_2Cl_2 (600 MHz) with assignment.

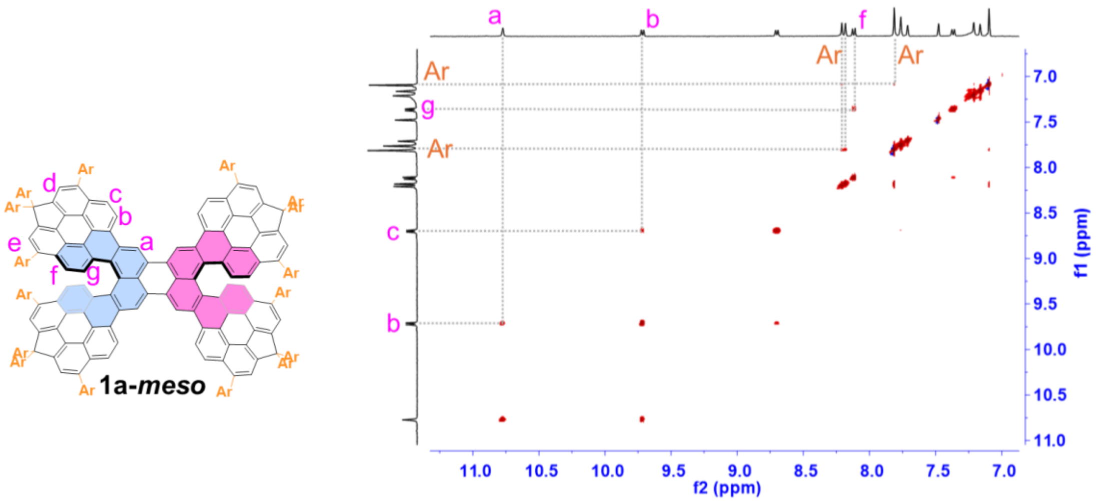

Fig. S9. Partial 2D NOESY NMR spectrum of **1a-*meso*** in CD_2Cl_2 (600 MHz) with assignment.

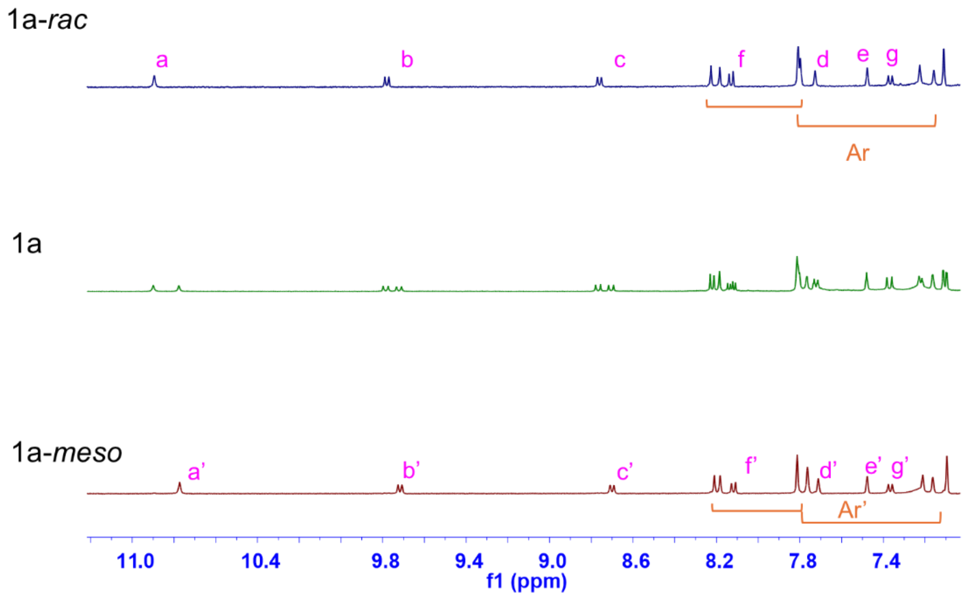

Fig. S10. Partial ^{1}H NMR spectra of **1a**, **1a-*rac*** and **1a-*meso*** in CD_2Cl_2 with assignment.

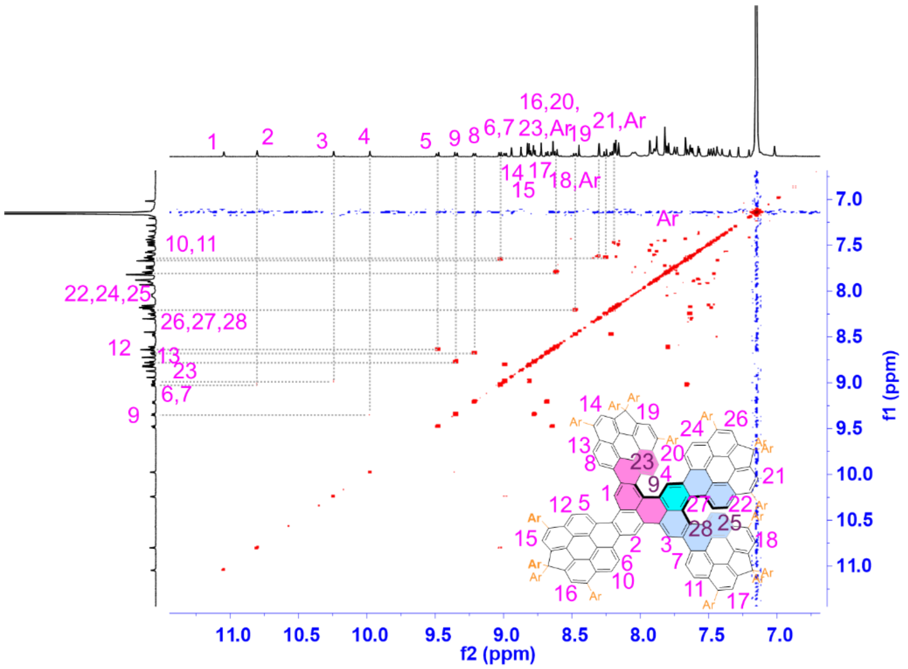

Fig. S11. Partial 2D COSY NMR spectrum of **1b** in C_6D_6 (500 MHz) with assignment.

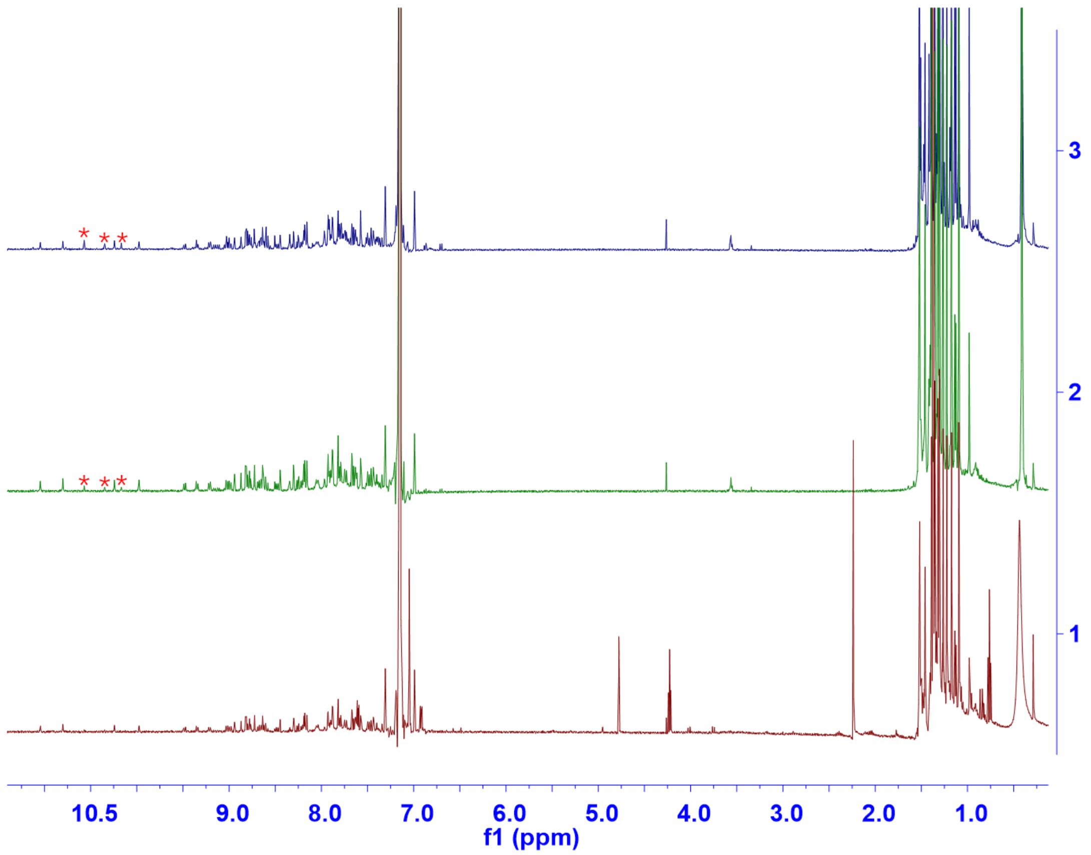

Fig. S12. The chemical transformation process of **1b** in solution monitored by ^{1}H NMR during a long time 2D-NMR experiments (about 4 h). Spectrum 1 corresponds to the initial ^{1}H NMR measurement. Spectrum 2 was recorded at the start of the 2D-NMR experiments, after washing the sample from Spectrum 1 with methanol to remove solvent impurities. Spectrum 3 was obtained after completing the 2D-NMR measurements. The peaks labelled with "*" indicate the formation of a new species.

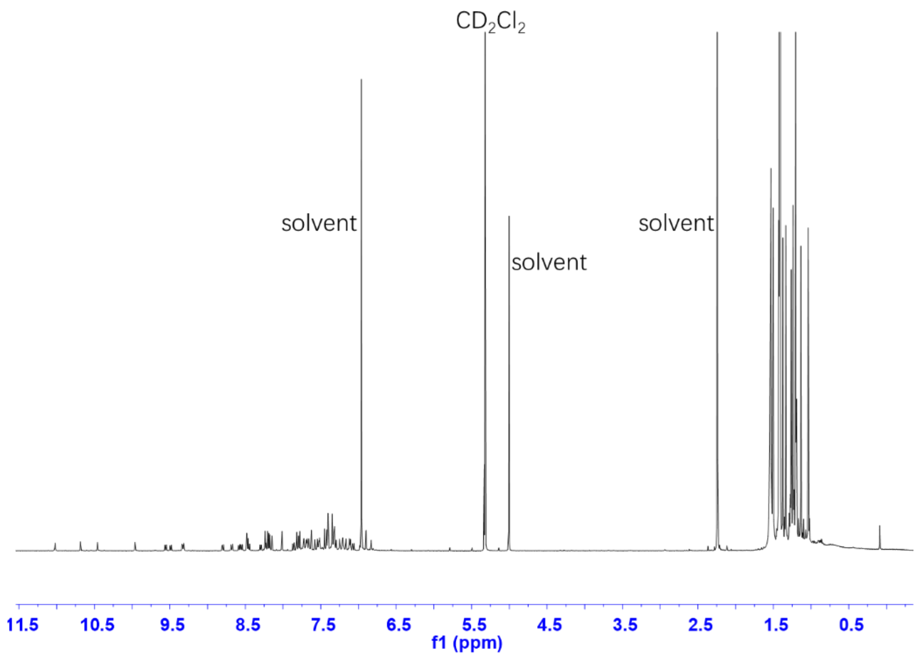

Fig. S13. ^{1}H NMR spectrum (500 MHz, CD_2Cl_2) of **1b** after purification by preparative HPLC. The clean spectrum with sharp peaks confirms the high purity of freshly prepared **1b**. The presence of multiple solvent signals is attributed to residual solvents from the preparative HPLC column.

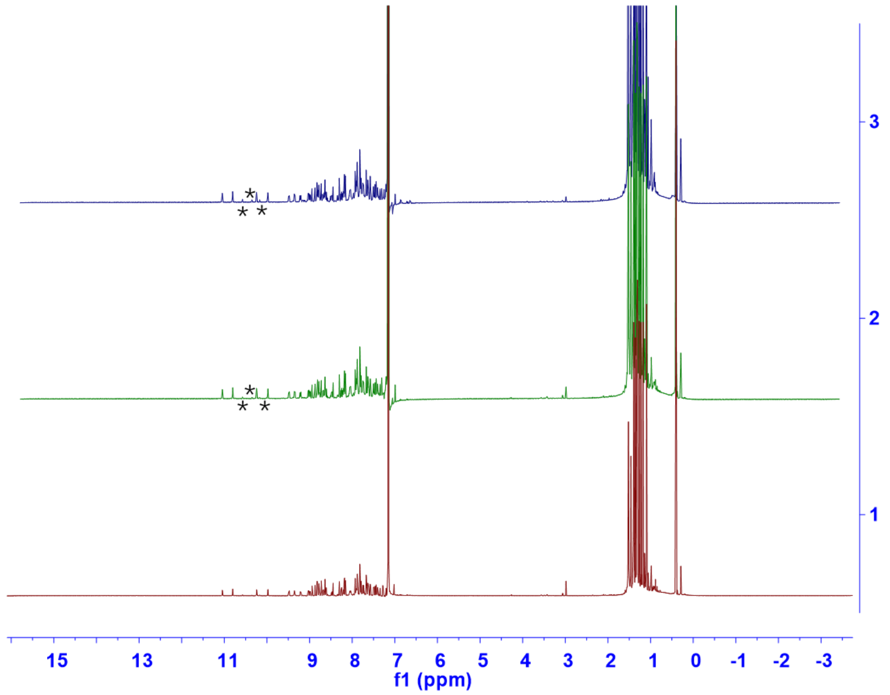

Fig. S14. Chemical transformation of **1b** in solution monitored by ^{1}H NMR over a short time period (~30 min) during 2D NMR experiments. Spectrum 1 corresponds to the initial ^{1}H NMR measurement after further purification by PTLC of the sample obtained from preparative HPLC (Fig. S10). Spectrum 2 was recorded at the beginning of the 2D NMR experiments. Spectrum

3 was acquired after completion of the 2D NMR measurements. Peaks marked with "*" indicate the formation of a new species.

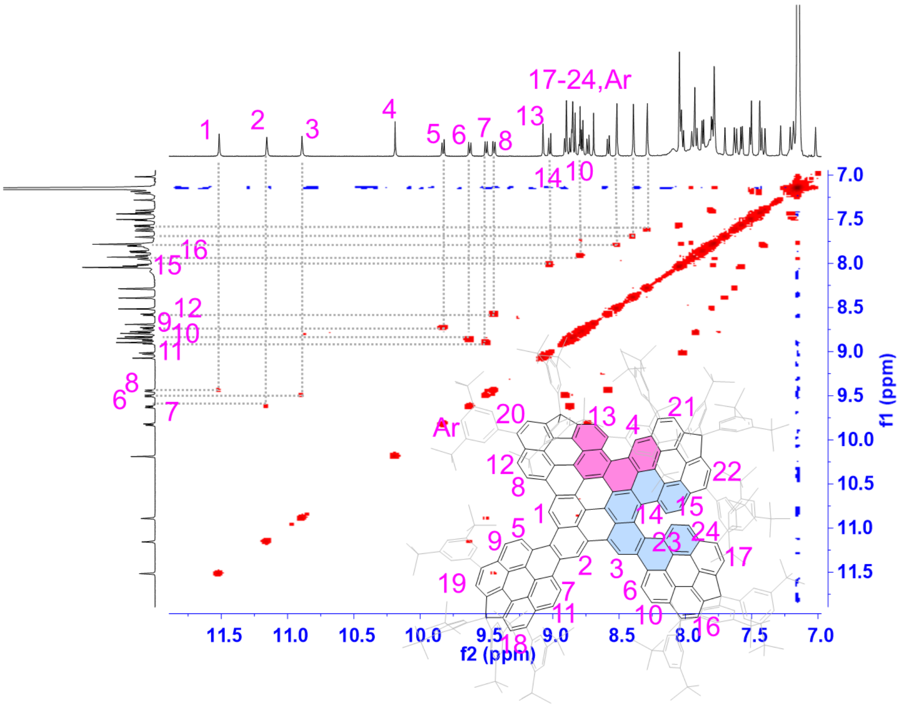

Fig. S15. Partial 2D COSY NMR spectrum of **1c** in C_6D_6 (500 MHz) with assignment.

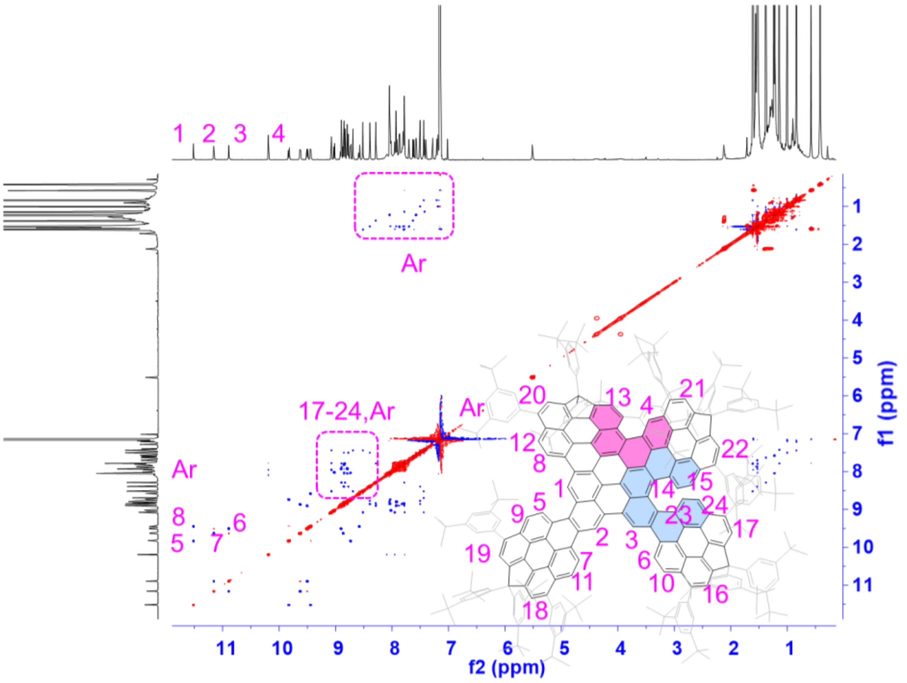

Fig. S16. 2D ROESY NMR spectrum of **1c** in C_6D_6 (500 MHz) with assignment.

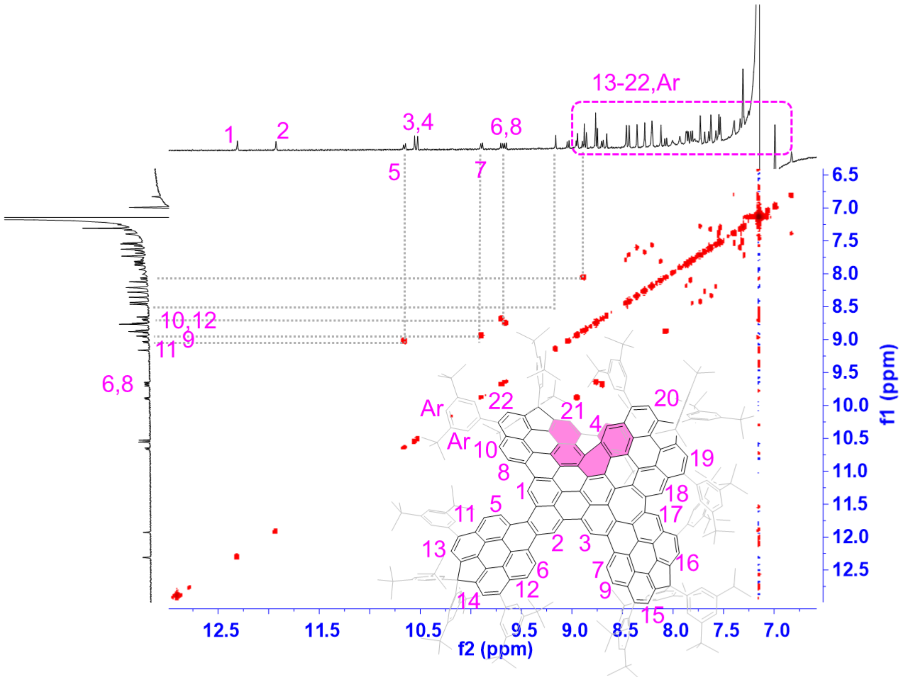

Fig. S17. Partial 2D COSY NMR spectrum of **1d** in C_6D_6 (500 MHz) with assignment.

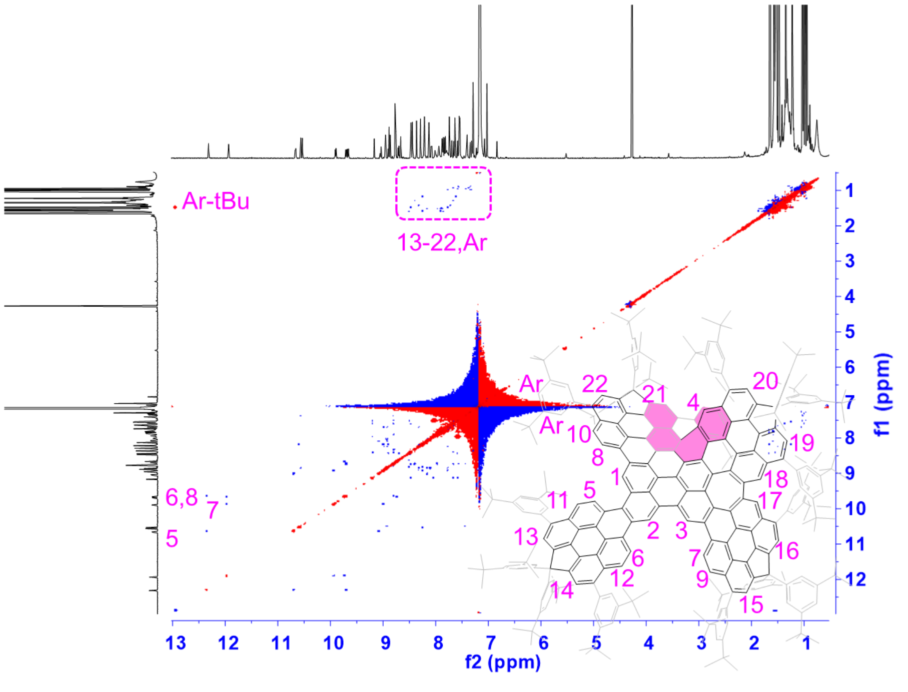

Fig. S18. 2D ROESY NMR spectrum of **1d** in C_6D_6 (500 MHz) with assignment.

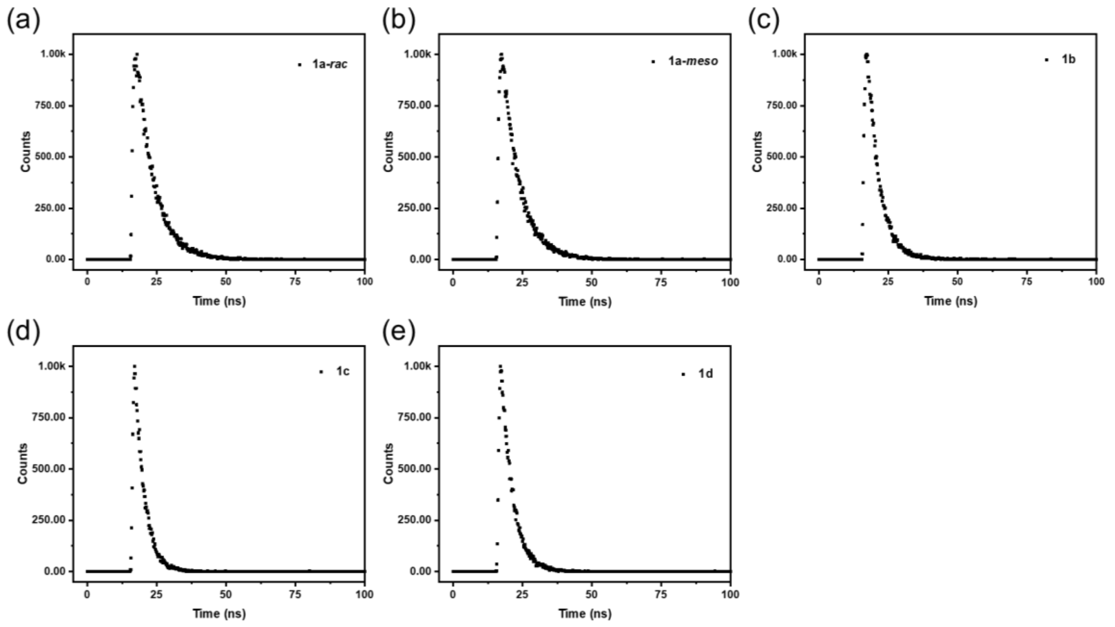

Fig. S19. Time-resolved fluorescence decay profile of **1a-*rac***, **1a-*meso***, **1b-d**. (a) **1a-*rac*** (λ_{exc} = 280 nm, λ_{probe} = 599 nm); (b) **1a-*meso*** (λ_{exc} = 280 nm, λ_{probe} = 599 nm); (c) **1b** (λ_{exc} = 280 nm, λ_{probe} = 661 nm); (d) **1c** (λ_{exc} = 365 nm, λ_{probe} = 760 nm); (e) **1d** (λ_{exc} = 365 nm, λ_{probe} = 786 nm).

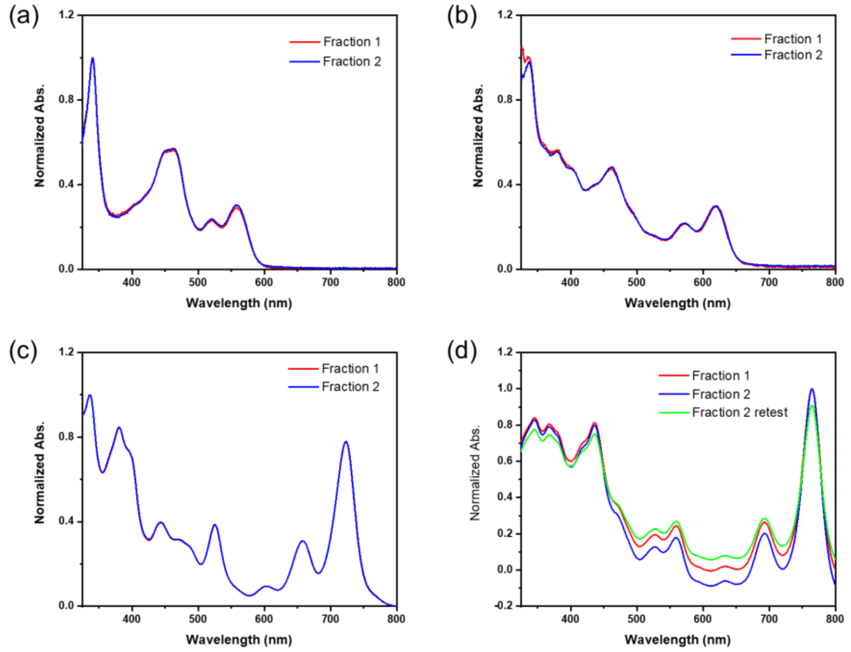

Fig. S20. Absorption spectra of the two fractions collected during the separation of (a) **1a-*rac***; (b) **1b**; (c) **1c**; and (d) **1d** by preparative HPLC. The separated fractions were recorded in THF/acetone (20:80). The nearly identical spectra of the two fractions confirm the purity of the collected samples. Minor differences are attributed to slight variations in solvent composition and baseline fluctuations, as evidenced by repeated measurements of Fraction 2 of **1d**, which show small inconsistencies.

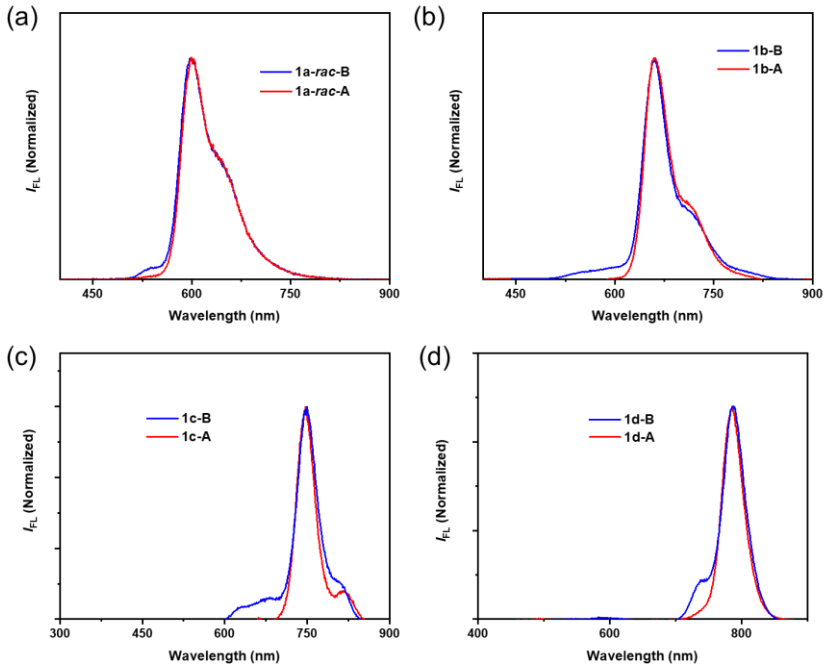

Fig. S21. Fluorescence spectra of freshly prepared (a) **1a-*rac***; (b) **1b**; (c) **1c**; and (d) **1d** under different solvent removal conditions. Condition A: solvent removed under a nitrogen stream with strict protection from light and without heating. Condition B: solvent removed using a rotary evaporator with heating and without strict light protection.

3. Chiral HPLC analysis and chiroptical spectra

Isolation of enantiomers of **1a-*rac*** by chiral HPLC

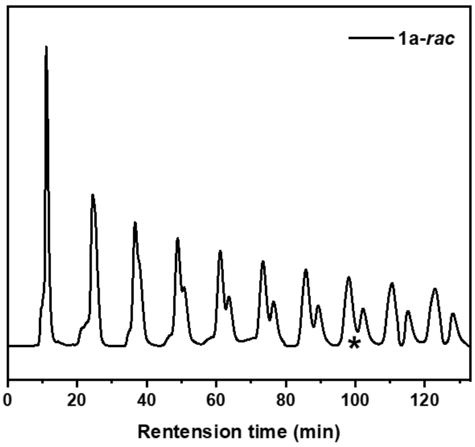

Fig. S22. HPLC chart for the separation of enantiomers of **1a-*rac*** using UV (350 nm) detectors in THF/acetone (20:80) at the flow rate of 8.0 ml/min. Optical resolution was carried out with a COSMOSIL Cholester column (2(i.d.) × 25 cm) at 25 °C. The portion labeled with "*" was collected first to ensure complete separation. The first and second fractions were determined as (*P*,*P*)-**1a-*rac*** and (*M*,*M*)-**1a-*rac*** based on experimental and theoretical circular dichroism (CD) spectra, respectively.

Isolation of enantiomers of **1b** by chiral HPLC

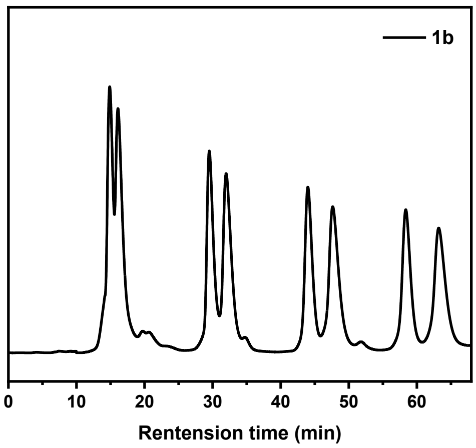

Fig. S23. HPLC chart for the separation of enantiomers of **1b** using UV (350 nm) detectors in THF/acetone (20:80) at the flow rate of 8.0 ml/min. Optical resolution was carried out with a COSMOSIL Cholester column (2(i.d.) × 25 cm) at 25 °C. The first and second fractions were determined as (*P*)[5](*M*)[6]-**1b** and (*M*)[5](*P*)[6]-**1b** based on experimental and theoretical circular dichroism (CD) spectra, respectively.

Isolation of enantiomers of **1c** by chiral HPLC

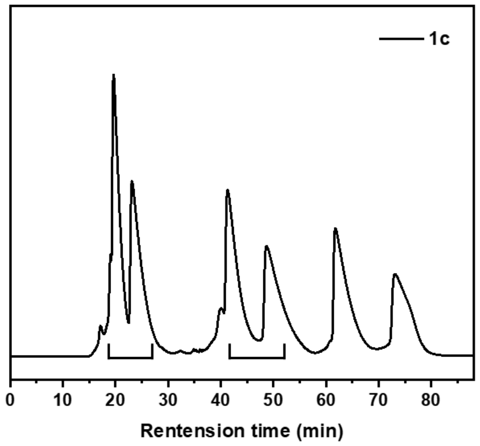

Fig. S24. HPLC chart for the separation of enantiomers of **1c** using UV (350 nm) detectors in THF/acetone (20:80) at the flow rate of 8.0 ml/min. Optical resolution was carried out with a COSMOSIL Cholester column (2(i.d.) × 25 cm) at 25 °C. The labels in the Fig. denote the ranges covered during the first and second HPLC separation cycles. The first and second fractions were determined as (*M*)[4](*P*)[6]-**1c** and (*P*)[4](*M*)[6]-**1c** based on experimental and theoretical circular dichroism (CD) spectra, respectively.

Isolation of enantiomers of **1d** by chiral HPLC

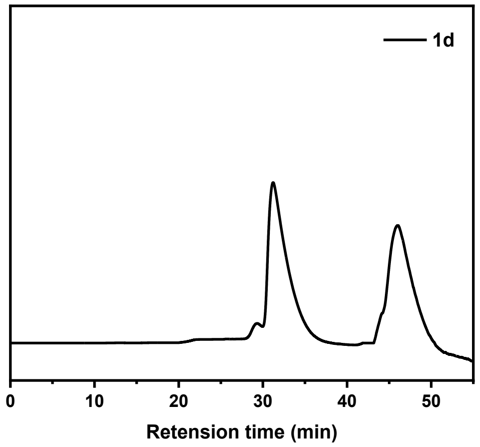

Fig. S25. HPLC chart for the separation of enantiomers of **1d** using UV (320 nm) detectors in THF/acetone (20:80) at the flow rate of 8.0 ml/min. Optical resolution was carried out with a COSMOSIL Cholester column (2(i.d.) × 25 cm) at 25 °C. The labels in the Fig. denote the ranges covered during the first and second HPLC separation cycles. The first and second fractions were determined as (*M*)-**1d** and (*P*)-**1d** based on experimental and theoretical circular dichroism (CD) spectra, respectively.

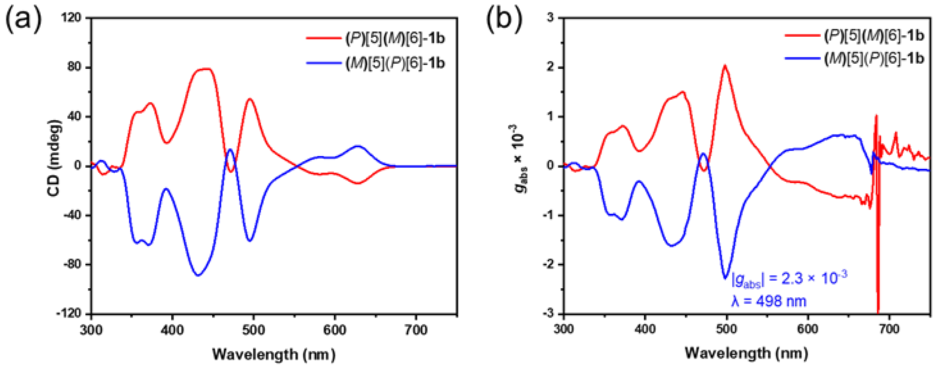

Fig. S26. (a), (b) CD spectra of the enantiomers of **1b** measured in DCM. ($c \sim 6.8 \times 10^{-6}$ M)

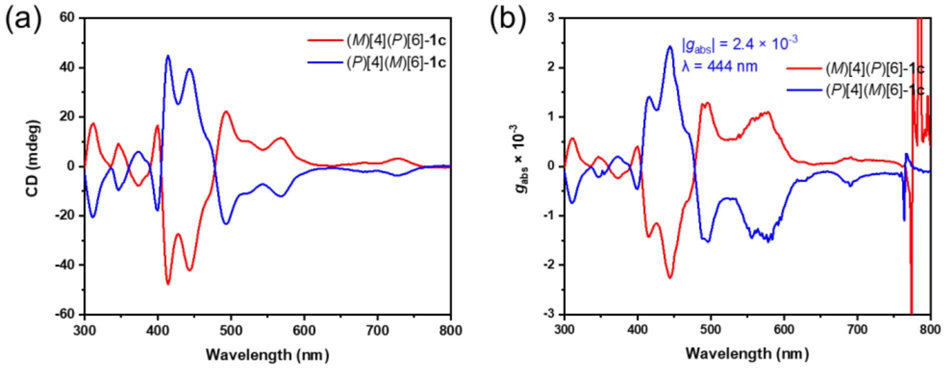

Fig. S27. (a), (b) CD spectra of the enantiomers of **1c** measured in DCM. ($c \sim 3.3 \times 10^{-6}$ M)

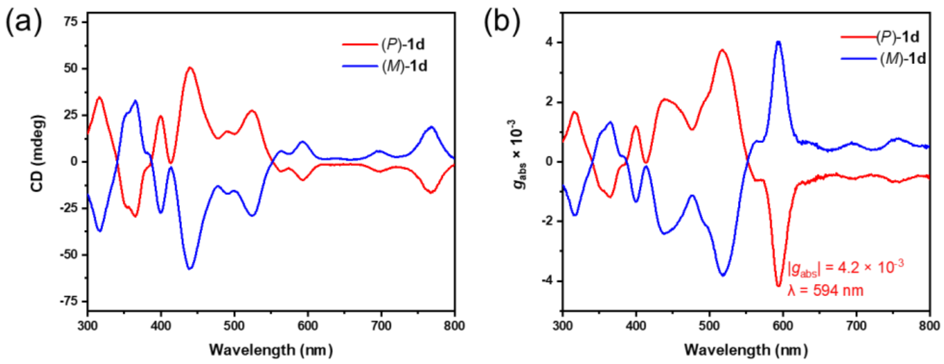

Fig. S28. (a), (b) CD spectra of the enantiomers of **1d** measured in DCM. ($c \sim 8.8 \times 10^{-6}$ M)

4. DFT calculations

Density functional theory (DFT) calculations were carried out using the Gaussian09 program package.[3] The Becke three-parameter hybrid exchange functional combined with the Lee–Yang–Parr correlation functional (B3LYP) was employed with the 6-31G(d,p) basis set for all atoms.[4] Geometry optimizations and time-dependent DFT (TD-DFT) calculations were performed at the B3LYP-D3/6-31G(d,p) level of theory, where the DFT-D3 dispersion correction was included to account for van der Waals interactions.[5] Solvent effects were considered using the polarizable continuum model (PCM).[6] All optimized structures were confirmed as true minima through vibrational frequency analysis, showing no imaginary frequencies. Transition dipole moments were visualized using UCSF Chimera.[7]

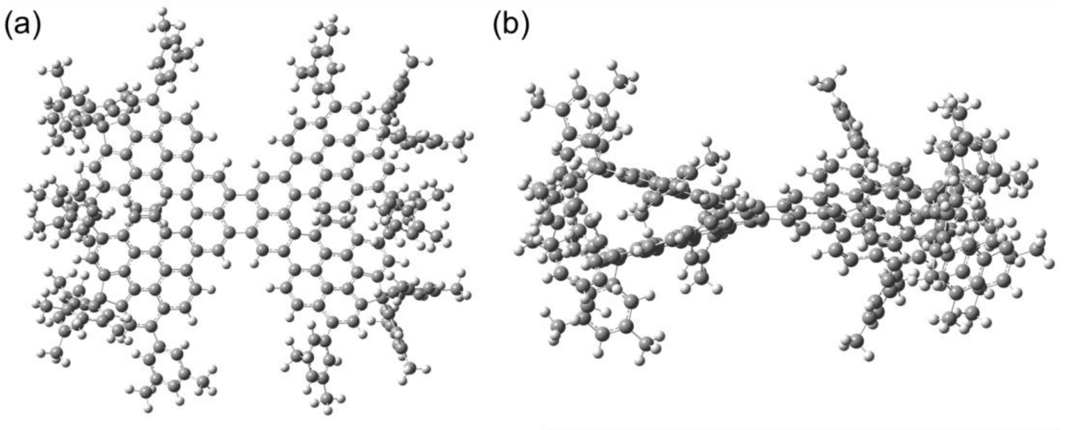

Fig. S29. The optimized geometry of **1a-*rac*** (a) top-view and (b) side-view with B3LYP-D3/6-31G (d,p) level of theory showing a butterfly-like geometry. The *tert*-butyl substituents are replaced by methyl groups during the calculations.

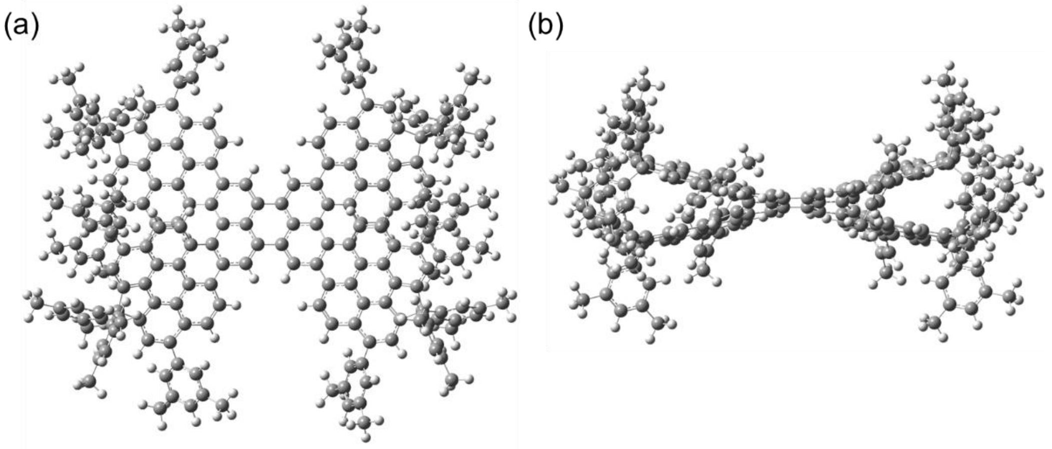

Fig. S30. The optimized geometry of **1a-*meso*** (a) top-view and (b) side-view with B3LYP-D3/6-31G (d,p) level of theory showing a butterfly-like geometry. The *tert*-butyl substituents are replaced by methyl groups during the calculations.

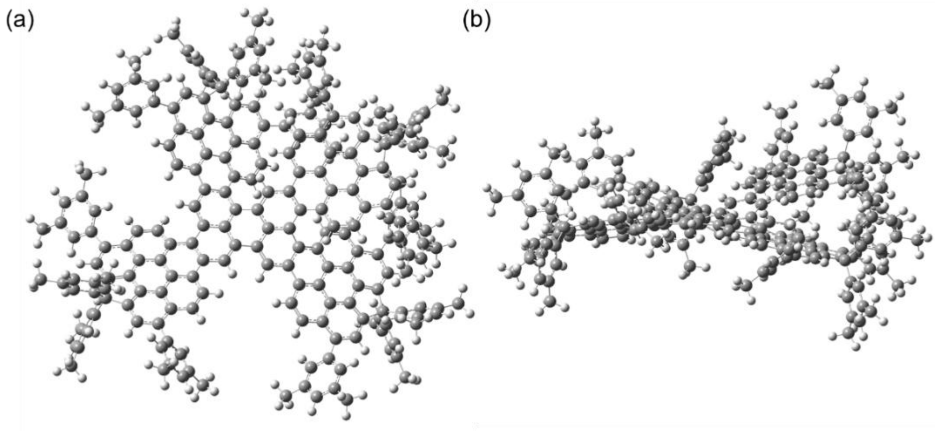

Fig. S31. The optimized geometry of **1b** (a) top-view and (b) side-view with B3LYP-D3/6-31G (d,p) level of theory showing a butterfly-like geometry. The *tert*-butyl substituents are replaced by methyl groups during the calculations.

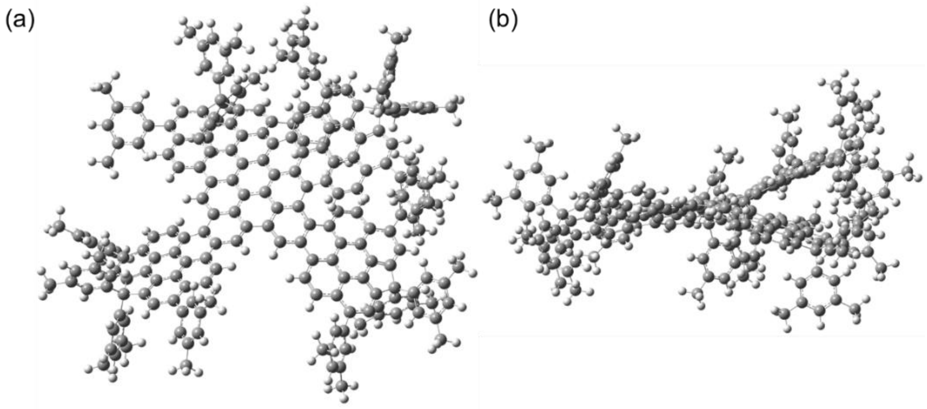

Fig. S32. The optimized geometry of **1c** (a) top-view and (b) side-view with B3LYP-D3/6-31G (d,p) level of theory showing a butterfly-like geometry. The *tert*-butyl substituents are replaced by methyl groups during the calculations.

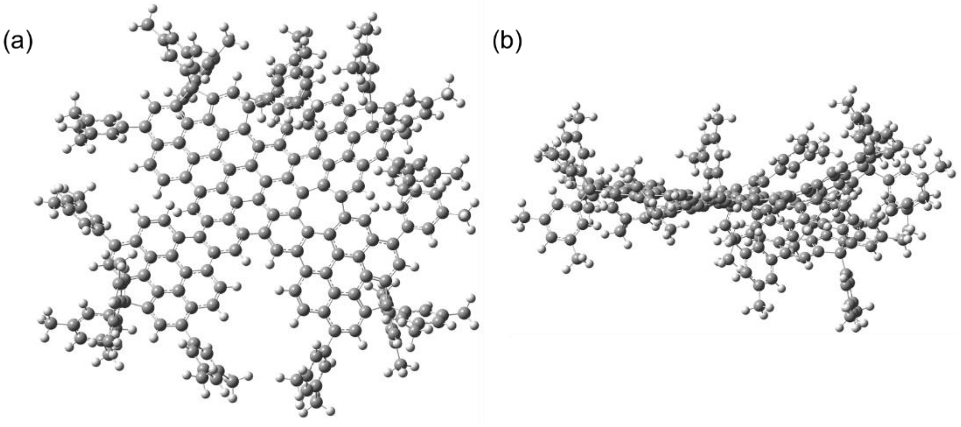

Fig. S33. The optimized geometry of **1d** (a) top-view and (b) side-view with B3LYP-D3/6-31G (d,p) level of theory showing a butterfly-like geometry. The *tert*-butyl substituents are replaced by methyl groups during the calculations.

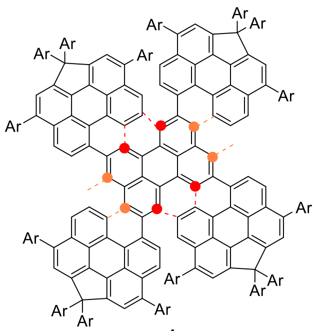

1

Possible isomers:

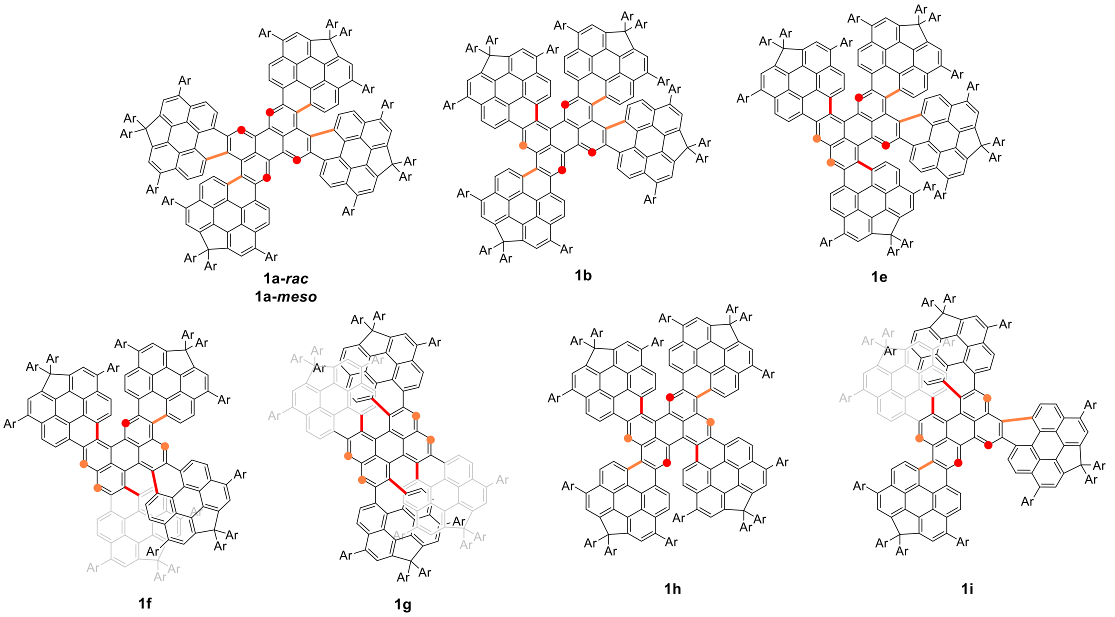

Fig. S34. The reaction sites for the final Scholl reaction and the possible final products.

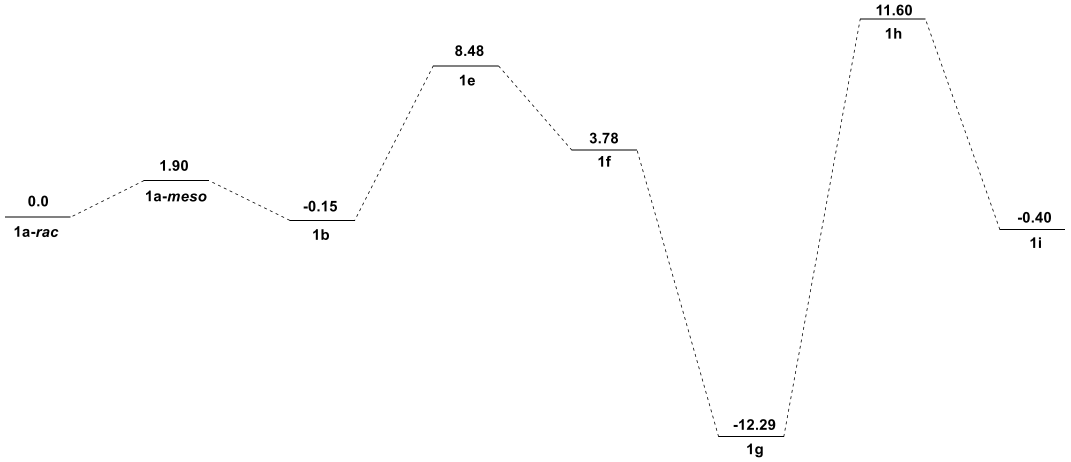

Fig. S35. The energy difference (in kcal/mol) of the possible products compared to compound **1a-*rac***.

Possible isomer of **1d**:

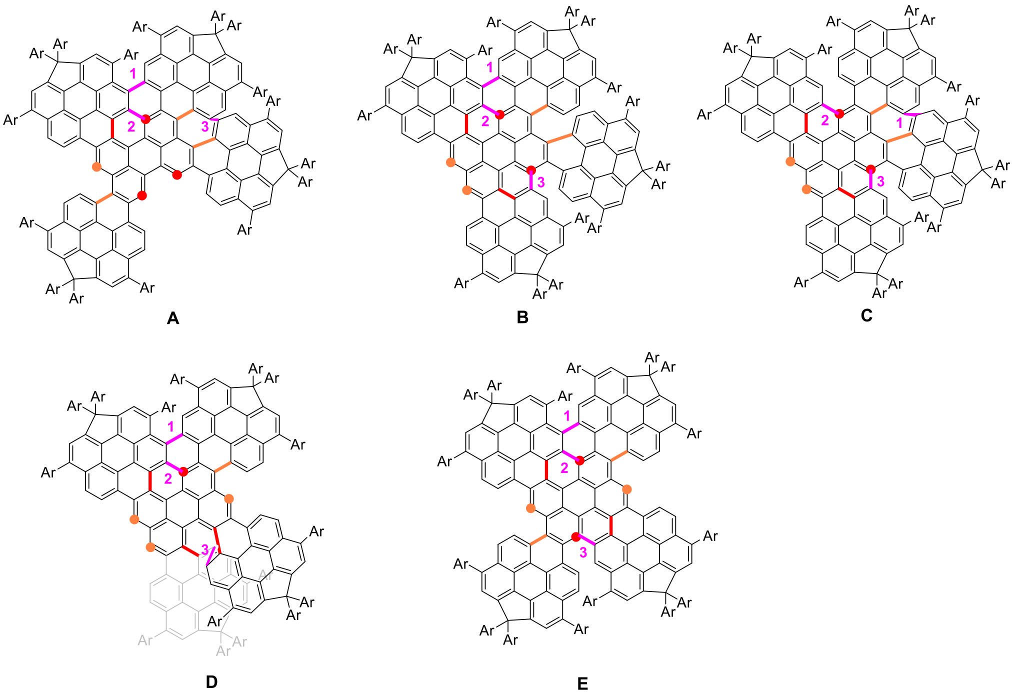

Fig. S36. The possible structure of **1d** is derived from the possible isomers of compound **1**.

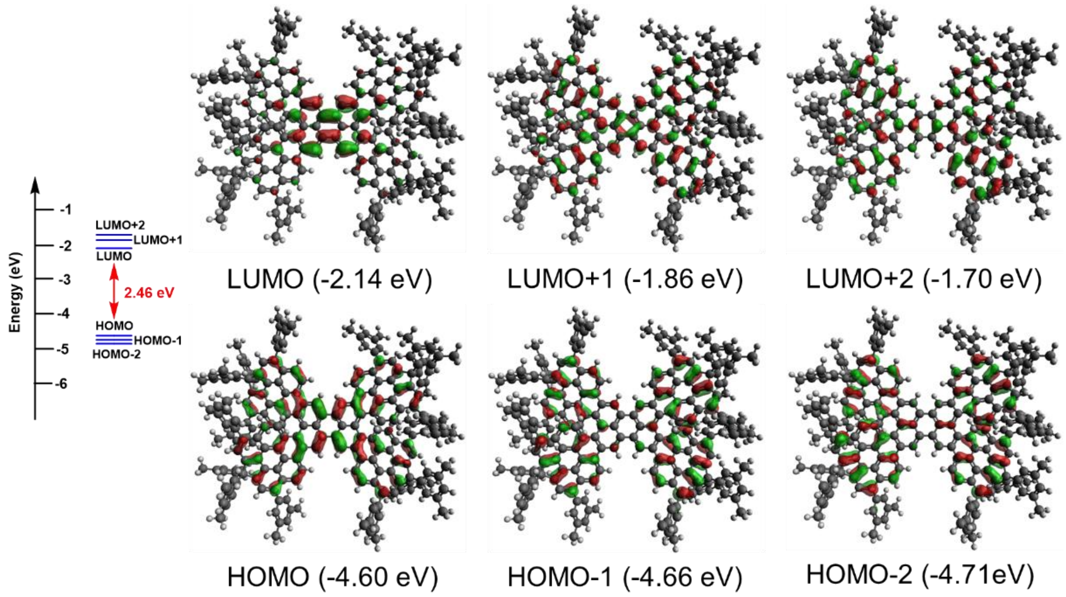

Fig. S37. Energy diagram and frontier molecular orbital profiles of **1a-*rac*** obtained by B3LYP-D3/6-31g(d,p) level calculation (isovalue = 0.02). The substituents are replaced by methyl groups during the calculations.

Table S1. Selected TD-DFT (B3LYP-D3/6-31G(d,p)) calculated wavelength, oscillator strength and compositions of major electronic transitions of **1a-*rac***.

Wavelength (nm)	Osc. Strength (*f*)	Major contribs
**599.1022**	1.3354	HOMO->LUMO (97%)
**583.8397**	0.0001	H-1->LUMO (95%)
**568.7087**	0.3422	H-2->LUMO (95%)
**537.4494**	0.0088	H-3->LUMO (94%)
**524.0466**	0.0094	HOMO->L+1 (93%)
**511.5493**	0.0371	H-4->LUMO (60%), H-1->L+1 (28%)
**500.5215**	0.3441	H-4->LUMO (24%), H-1->L+1 (60%)
**496.7713**	0.0017	H-2->L+1 (79%), H-1->L+2 (16%)
**489.4564**	0.994	H-3->L+1 (15%), HOMO->L+2 (80%)
**479.5552**	0.0273	HOMO->L+3 (80%)
**478.6111**	0.2025	H-3->L+1 (35%), H-1->L+3 (32%)
**477.3029**	0.0542	H-3->L+1 (10%), H-2->L+3 (25%), H-1->L+3 (22%), H-1->L+4 (28%)
**475.5089**	0.2098	HOMO->L+4 (78%)
**472.771**	0.217	H-3->L+1 (25%), H-2->L+4 (26%), H-1->L+3 (11%), H-1->L+4 (29%)
**463.423**	0.0215	H-4->L+1 (11%), H-2->L+1 (15%), H-1->L+2 (66%)
**463.3364**	0.1064	H-4->L+1 (53%), H-3->L+2 (20%), H-1->L+2 (13%)
**454.5375**	0.324	H-2->L+2 (90%)
**443.6404**	0.0014	H-2->L+3 (60%), H-1->L+3 (28%)
**440.9268**	0.0025	H-2->L+4 (56%), H-1->L+4 (33%)
**440.1597**	0.2033	H-4->L+4 (14%), H-3->L+3 (49%), H-3->L+4 (29%)
**437.5038**	0.0033	H-4->L+3 (17%), H-3->L+3 (23%), H-3->L+4 (47%)
**436.2568**	0.0048	H-4->L+1 (21%), H-3->L+2 (63%)
**426.1064**	0.0153	H-4->L+2 (80%)
**416.6976**	0.0173	H-4->L+3 (69%), H-3->L+3 (13%)
**409.8651**	0.0297	H-4->L+4 (75%)
**405.6676**	0.0208	H-5->LUMO (90%)
**399.215**	0.0005	H-6->LUMO (37%), HOMO->L+5 (44%)
**394.741**	0.0153	H-7->LUMO (22%), HOMO->L+6 (70%)
**391.8219**	0.3489	H-8->LUMO (10%), H-1->L+5 (60%)
**390.01**	0.0288	H-7->LUMO (23%), H-2->L+5 (17%), HOMO->L+6 (10%), HOMO->L+7 (35%)
**385.4031**	0.0001	H-6->LUMO (46%), HOMO->L+5 (35%)
**383.567**	0.0377	H-1->L+6 (79%)
**381.4194**	0.0084	H-5->L+1 (21%), H-3->L+5 (15%), H-1->L+7 (34%)
**379.7605**	0.0007	H-8->LUMO (22%), H-1->L+5 (21%), HOMO->L+8 (21%)
**379.1566**	0.0037	H-7->LUMO (15%), H-2->L+5 (57%)
**377.9084**	0.0001	H-2->L+6 (67%), H-1->L+8 (14%)

**372.6158**	0.0384	H-9->LUMO (24%), H-8->LUMO (18%), H-7->LUMO (12%), HOMO->L+7 (22%)
**371.7667**	0.0538	H-9->LUMO (22%), H-7->LUMO (16%), HOMO->L+7 (23%)
**370.8216**	0.0013	H-5->L+2 (11%), H-2->L+7 (39%), H-1->L+8 (10%)
**368.7043**	0.001	H-9->LUMO (15%), H-3->L+6 (49%)

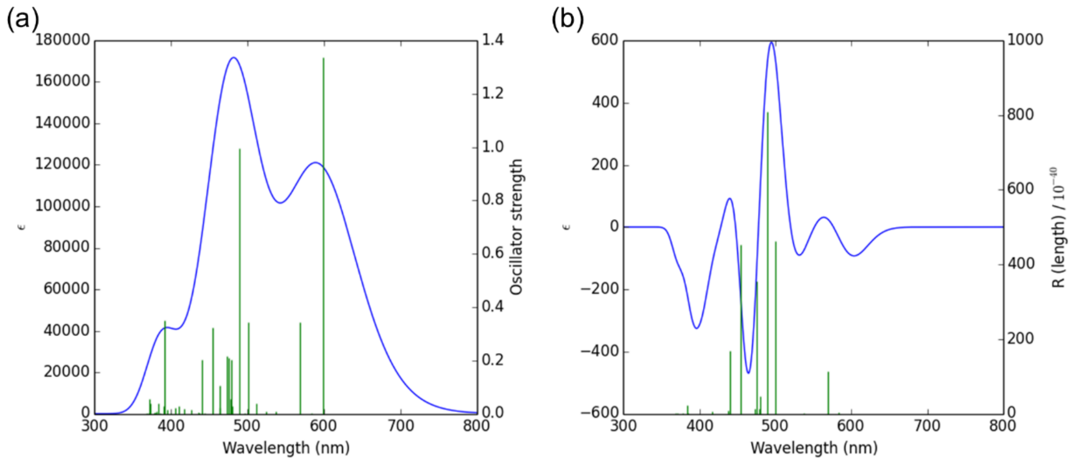

Fig. S38. Calculated (B3LYP-D3/6-31G(d,p)) (a) absorption spectrum of **1a-*rac***, (b) CD spectrum of (*M*,*M*)**-1a-*rac*** in DCM.

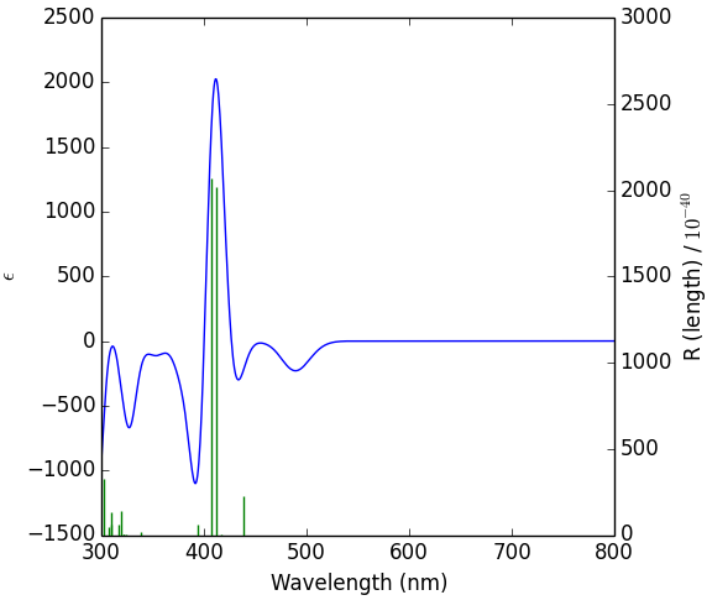

Fig. S39. Calculated (M06-2X/6-31G(d,p)) CD spectrum of (*M*,*M*)**-1a-*rac*** in DCM.

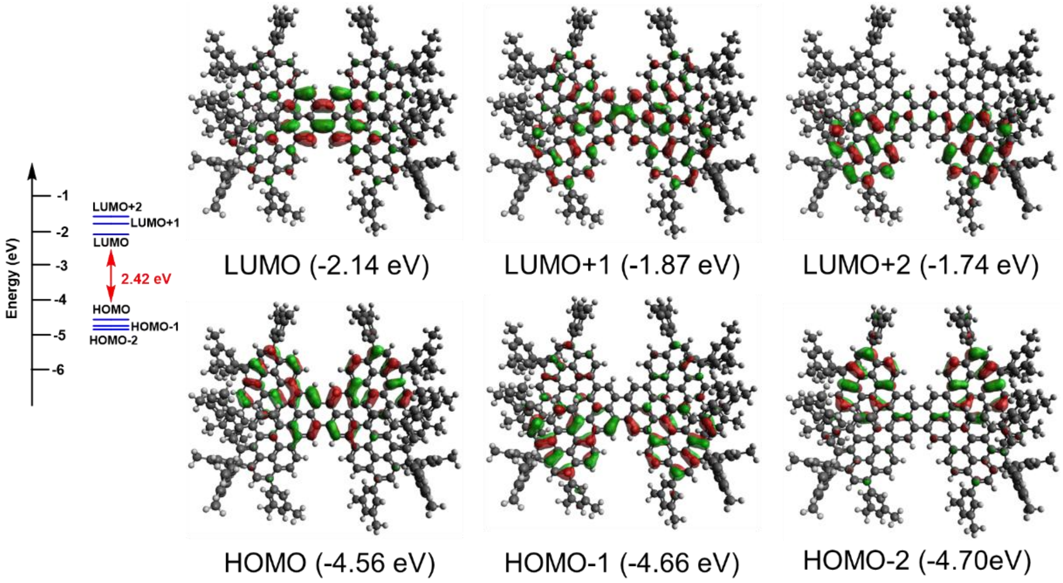

Fig. S40. Energy diagram and frontier molecular orbital profiles of **1a-*meso*** obtained by B3LYP-D3/6-31g(d,p) level calculation (isovalue = 0.02). The substituents are replaced by methyl groups during the calculations.

Table S2. Selected TD-DFT (B3LYP-D3/6-31G(d,p)) calculated wavelength, oscillator strength and compositions of major electronic transitions of **1a-*meso***.

Wavelength (nm)	Osc. Strength (*f*)	Major contribs
**602.948**	1.3464	HOMO->LUMO (97%)
**585.6599**	0.0241	H-1->LUMO (93%)
**567.5633**	0.3176	H-2->LUMO (94%)
**540.4716**	0.0007	H-3->LUMO (94%)
**532.9673**	0.0002	HOMO->L+1 (91%)
**518.3719**	0.035	H-4->LUMO (62%), H-1->L+1 (28%)
**506.6991**	0.3558	H-4->LUMO (24%), H-1->L+1 (60%), HOMO->L+4 (10%)
**502.1433**	0.0327	H-2->L+1 (74%), H-1->L+2 (13%)
**495.4017**	0.8551	H-3->L+1 (14%), HOMO->L+2 (69%)
**484.1052**	0.0837	H-3->L+1 (62%)
**483.4257**	0.1728	HOMO->L+3 (84%)
**481.8475**	0.1644	H-1->L+4 (15%), HOMO->L+4 (51%)
**478.9438**	0.086	H-2->L+3 (14%), H-1->L+4 (40%), HOMO->L+4 (27%)
**477.5603**	0.435	H-3->L+1 (11%), H-2->L+4 (25%), H-1->L+2 (22%), H-1->L+3 (32%)
**473.1138**	0.0017	H-4->L+1 (63%), H-3->L+2 (18%)
**466.0534**	0.0059	H-2->L+1 (16%), H-1->L+2 (53%), H-1->L+3 (26%)
**456.546**	0.2623	H-2->L+2 (78%), H-2->L+3 (11%)
**445.9703**	0.2258	H-4->L+4 (14%), H-3->L+2 (32%), H-3->L+3 (34%)

**444.1808**	0.0024	H-3->L+4 (12%), H-2->L+4 (51%), H-1->L+3 (20%)
**443.9105**	0.001	H-3->L+2 (15%), H-2->L+3 (45%), H-1->L+4 (26%)
**441.2563**	0.0032	H-4->L+3 (14%), H-3->L+4 (57%), H-2->L+4 (11%)
**438.9132**	0.0187	H-4->L+1 (13%), H-3->L+2 (25%), H-3->L+3 (42%)
**432.3622**	0.0379	H-4->L+2 (71%)
**419.1629**	0.0015	H-4->L+3 (62%), H-3->L+4 (21%)
**415.6638**	0.0379	H-4->L+4 (75%), H-3->L+3 (17%)
**405.005**	0.0016	H-5->LUMO (89%)
**400.3494**	0.0021	H-6->LUMO (30%), HOMO->L+5 (53%)
**398.7271**	0.0002	H-7->LUMO (24%), HOMO->L+6 (67%)
**392.6657**	0.0036	H-7->LUMO (15%), HOMO->L+6 (11%), HOMO->L+7 (51%)
**392.4668**	0.3203	H-1->L+5 (59%)
**386.1233**	0.0128	H-2->L+8 (10%), H-1->L+6 (77%)
**385.955**	0.0044	H-6->LUMO (44%), HOMO->L+5 (26%)
**383.7332**	0.0317	H-5->L+1 (23%), H-3->L+5 (11%), H-1->L+7 (41%)
**380.3546**	0.0056	H-9->LUMO (15%), H-1->L+5 (17%), HOMO->L+8 (30%)
**379.679**	0.0002	H-2->L+5 (65%)
**379.4002**	0.0002	H-2->L+6 (55%), H-1->L+8 (13%)
**376.3369**	0.0005	H-7->LUMO (44%), HOMO->L+6 (14%), HOMO->L+7 (30%)
**373.1317**	0.1081	H-9->LUMO (42%), H-8->LUMO (23%)
**372.0344**	0.0331	H-2->L+7 (34%)
**371.0324**	0.0256	H-9->LUMO (15%), H-3->L+6 (50%)

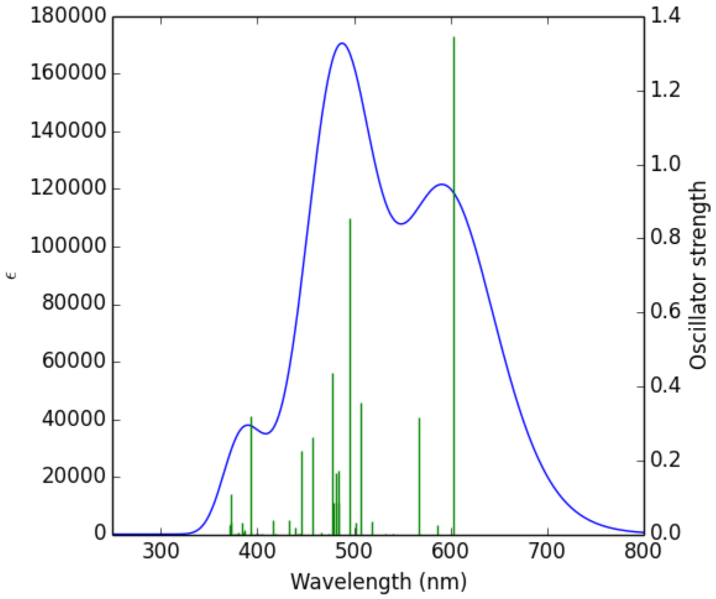

Fig. S41. Calculated (B3LYP-D3/6-31G(d,p)) (a) absorption spectrum of **1a-*meso*** in DCM.

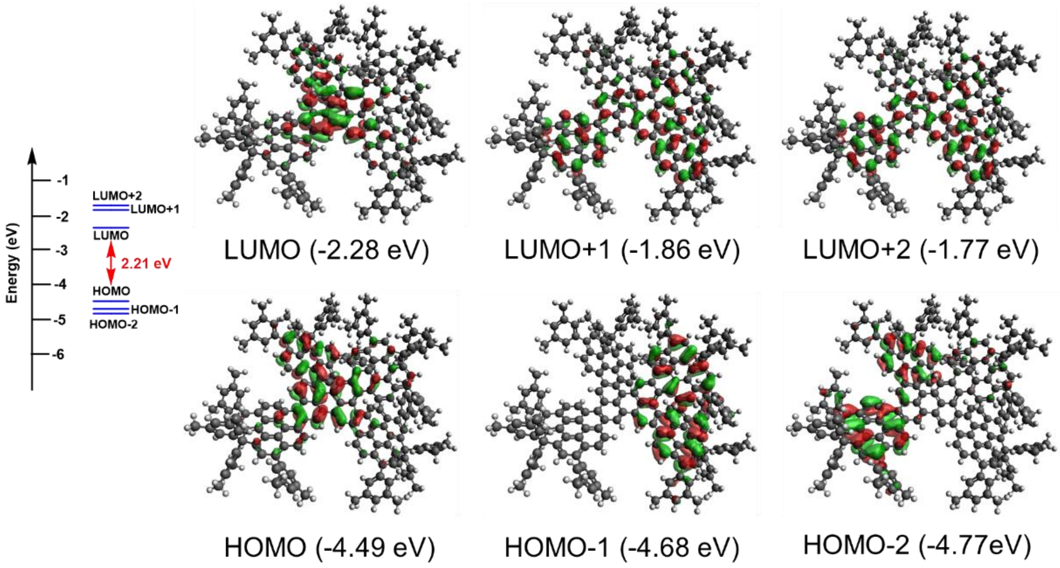

Fig. S42. Energy diagram and frontier molecular orbital profiles of **1b** obtained by B3LYP-D3/6-31g(d,p) level calculation (isovalue = 0.02). The substituents are replaced by methyl groups during the calculations.

Table S3. Selected TD-DFT (B3LYP-D3/6-31G(d,p)) calculated wavelength, oscillator strength and compositions of major electronic transitions of **1b**.

Wavelength (nm)	Osc. Strength (*f*)	Major contribs
**672.3655**	0.9252	HOMO->LUMO (99%)
**612.6004**	0.0907	H-1->LUMO (94%)
**592.4038**	0.1831	H-2->LUMO (96%)
**566.0862**	0.04	H-3->LUMO (84%)
**550.4537**	0.0598	HOMO->L+1 (81%)
**533.8853**	0.0524	H-4->LUMO (75%), HOMO->L+2 (11%)
**524.5788**	0.3502	HOMO->L+2 (72%), HOMO->L+4 (10%)
**508.5071**	0.3629	H-1->L+1 (35%), HOMO->L+4 (44%)
**507.3626**	0.079	H-1->L+1 (10%), HOMO->L+3 (74%)
**499.5535**	0.0557	H-1->L+1 (40%), HOMO->L+3 (13%), HOMO->L+4 (34%)
**483.5766**	0.0232	H-2->L+1 (56%), H-1->L+3 (22%)
**483.2373**	0.157	H-2->L+1 (21%), H-1->L+3 (61%)
**476.6056**	0.3192	H-3->L+1 (65%), H-2->L+2 (14%)
**469.8685**	0.0814	H-1->L+2 (65%)
**462.455**	0.1344	H-4->L+1 (13%), H-2->L+2 (62%)
**454.6375**	0.0756	H-1->L+2 (12%), H-1->L+4 (54%)
**451.6728**	0.0091	H-3->L+3 (39%), H-2->L+3 (44%)
**449.6254**	0.4373	H-4->L+1 (24%), H-3->L+2 (20%), H-2->L+2 (11%), H-1->L+4 (22%)
**446.3395**	0.3212	H-4->L+1 (13%), H-2->L+4 (61%)
**441.1778**	0.0091	H-4->L+1 (20%), H-3->L+2 (48%), H-2->L+4 (12%)

**439.0064**	0.0716	H-4->L+3 (11%), H-3->L+3 (35%), H-2->L+3 (38%)
**429.7397**	0.1398	H-5->LUMO (20%), H-4->L+2 (13%), HOMO->L+5 (32%)
**427.5907**	0.0219	H-3->L+4 (77%)
**426.5609**	0.1389	H-5->LUMO (11%), H-4->L+2 (53%), HOMO->L+5 (12%)
**419.0921**	0.0038	H-4->L+3 (37%), HOMO->L+6 (29%)
**414.9821**	0.0226	H-6->LUMO (26%), H-4->L+3 (36%), HOMO->L+6 (19%)
**411.361**	0.0034	H-7->LUMO (39%), H-6->LUMO (30%)
**411.0882**	0.014	H-4->L+4 (75%)
**407.588**	0.3341	H-5->LUMO (31%), H-4->L+4 (10%), HOMO->L+5 (21%), HOMO->L+6 (14%)
**402.4546**	0.1022	H-8->LUMO (24%), H-7->LUMO (17%), HOMO->L+7 (27%)
**400.0393**	0.0425	H-8->LUMO (15%), H-7->LUMO (18%), H-6->LUMO (19%), H-5->LUMO (12%), HOMO->L+6 (13%)
**392.5041**	0.2224	H-1->L+5 (65%)
**389.6791**	0.1603	H-1->L+5 (14%), HOMO->L+7 (10%), HOMO->L+8 (52%)
**384.185**	0.2193	H-2->L+5 (66%)
**383.5314**	0.0425	H-1->L+6 (64%)
**379.4118**	0.1388	H-8->LUMO (11%), H-3->L+5 (14%), H-1->L+8 (23%), HOMO->L+7 (11%)
**378.6239**	0.2071	H-8->LUMO (23%), H-1->L+8 (10%), HOMO->L+7 (17%)
**376.6914**	0.0664	H-2->L+6 (69%)
**373.0194**	0.0121	H-9->LUMO (83%)
**371.1546**	0.1144	H-10->LUMO (14%), H-3->L+5 (43%)

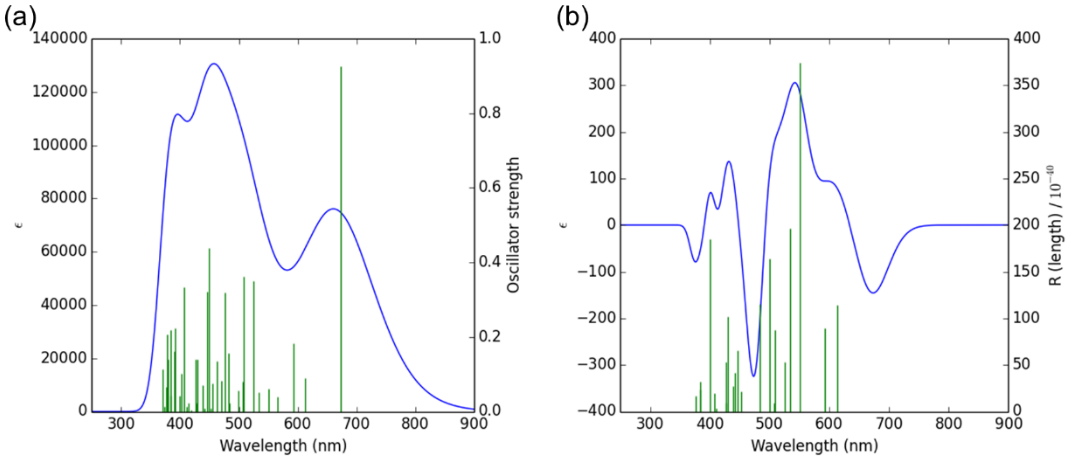

Fig. S43. Calculated (B3LYP-D3/6-31G(d,p)) (a) absorption spectrum of **1b**, (b) CD spectrum of (*P*)[5](*M*)[6]-**1b** in DCM.

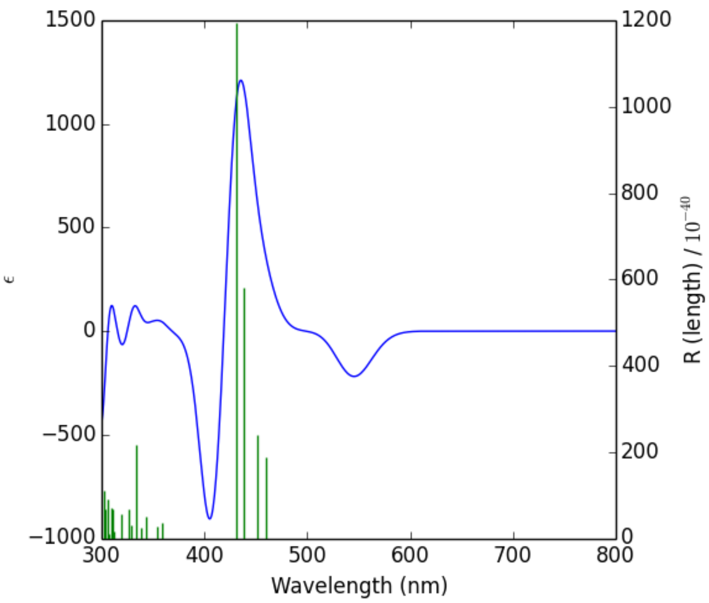

Fig. S44. Calculated (M06-2X/6-31G(d,p)) CD spectrum of (*P*)[5](*M*)[6]-**1b** in DCM.

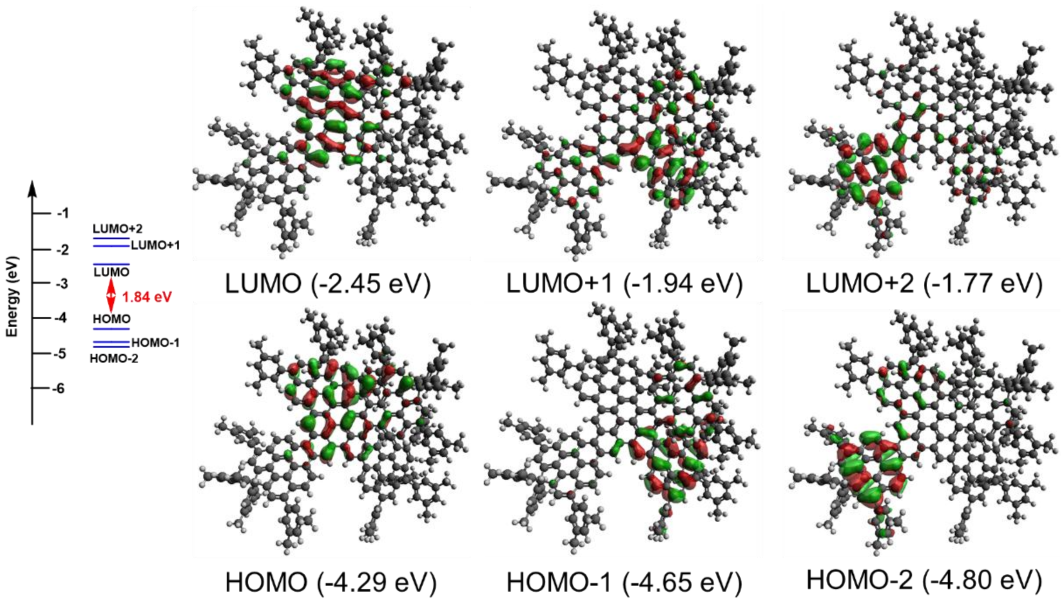

Fig. S45. Energy diagram and frontier molecular orbital profiles of **1c** obtained by B3LYP-D3/6-31g(d,p) level calculation (isovalue = 0.02). The substituents are replaced by methyl groups during the calculations.

Table S4. Selected TD-DFT (B3LYP-D3/6-31G(d,p)) calculated wavelength, oscillator strength and compositions of major electronic transitions of **1c**.

Wavelength (nm)	Osc. Strength (*f*)	Major contribs
**783.1735**	0.9718	HOMO->LUMO (99%)

**668.1623**	0.1217	H-1->LUMO (88%)
**629.2975**	0.1004	H-3->LUMO (13%), H-2->LUMO (76%)
**618.19**	0.1979	HOMO->L+1 (81%)
**608.6607**	0.0213	H-3->LUMO (77%), H-2->LUMO (10%)
**564.2827**	0.42	HOMO->L+2 (84%)
**550.4537**	0.0069	HOMO->L+3 (88%)
**539.6013**	0.0686	H-4->LUMO (77%), HOMO->L+4 (10%)
**530.4364**	0.1193	H-1->L+1 (70%), HOMO->L+4 (22%)
**519.7191**	0.0502	H-4->LUMO (12%), H-1->L+1 (19%), HOMO->L+4 (63%)
**494.4731**	0.0675	H-3->L+1 (80%), H-2->L+1 (13%)
**490.4244**	0.0474	H-2->L+1 (72%), H-1->L+3 (10%)
**485.0712**	0.3339	H-1->L+2 (20%), H-1->L+3 (61%)
**474.6533**	0.0133	H-2->L+2 (10%), H-1->L+2 (61%), H-1->L+3 (20%)
**464.7781**	0.0225	H-6->LUMO (17%), H-5->LUMO (14%), HOMO->L+5 (26%), HOMO->L+6 (20%)
**462.3343**	0.3419	H-2->L+2 (71%)
**453.6228**	0.0195	H-6->LUMO (15%), H-5->LUMO (18%), H-4->L+1 (11%), H-1->L+4 (11%), HOMO->L+5 (20%), HOMO->L+6 (11%)
**448.2599**	0.1626	H-3->L+3 (77%)
**443.1489**	0.0112	H-6->LUMO (11%), H-1->L+4 (44%), HOMO->L+6 (11%)
**442.0271**	0.0795	H-6->LUMO (14%), H-5->LUMO (19%), HOMO->L+5 (21%), HOMO->L+6 (14%)
**440.0035**	0.0501	H-3->L+2 (65%)
**436.8102**	0.0685	H-4->L+1 (44%), H-2->L+3 (11%), H-1->L+4 (10%)
**435.2155**	0.0406	H-2->L+3 (73%)
**430.7699**	0.5217	H-6->LUMO (22%), H-4->L+1 (12%), HOMO->L+6 (11%), HOMO->L+7 (31%)
**428.4625**	0.3831	H-8->LUMO (11%), H-5->LUMO (20%), HOMO->L+7 (31%)
**421.7149**	0.2798	H-3->L+4 (11%), H-2->L+4 (76%)
**418.6534**	0.1201	H-7->LUMO (54%), HOMO->L+8 (30%)
**414.3164**	0.0749	H-7->LUMO (22%), HOMO->L+8 (44%)
**413.7496**	0.1535	H-3->L+4 (63%), H-2->L+4 (10%)
**408.1515**	0.1059	H-9->LUMO (24%), H-8->LUMO (22%), H-4->L+2 (26%)
**407.1864**	0.1269	H-9->LUMO (20%), H-4->L+2 (45%)
**403.9626**	0.2805	H-9->LUMO (23%), H-8->LUMO (32%)
**400.634**	0.2184	H-4->L+3 (33%), H-1->L+5 (35%)
**398.8169**	0.0611	H-1->L+5 (10%), HOMO->L+9 (58%)
**397.6274**	0.025	H-4->L+3 (40%), H-1->L+5 (26%)
**395.8248**	0.0157	H-16->LUMO (28%), H-12->LUMO (27%), H-11->LUMO (10%), H-10->LUMO (11%)
**391.031**	0.0444	H-16->LUMO (29%), H-10->LUMO (23%)
**388.1177**	0.2063	H-2->L+5 (32%), H-1->L+7 (10%)
**386.7255**	0.0985	H-14->LUMO (47%), H-12->LUMO (16%)
**384.471**	0.0113	H-18->LUMO (17%), H-13->LUMO (30%), H-11->LUMO (22%)

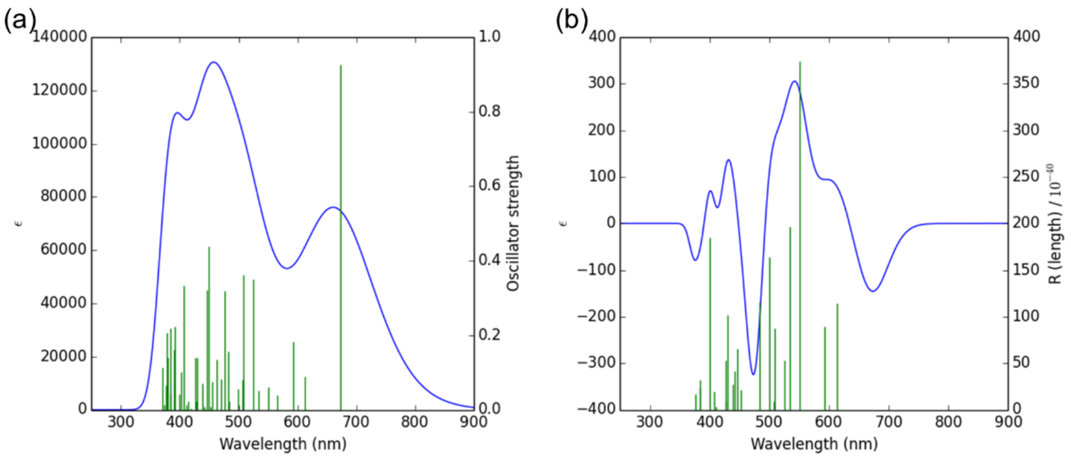

Fig. S46. Calculated (B3LYP-D3/6-31G(d,p)) (a) absorption spectrum of **1c**, (b) CD spectrum of (*P*)[4](*M*)[6]-**1c** in DCM.

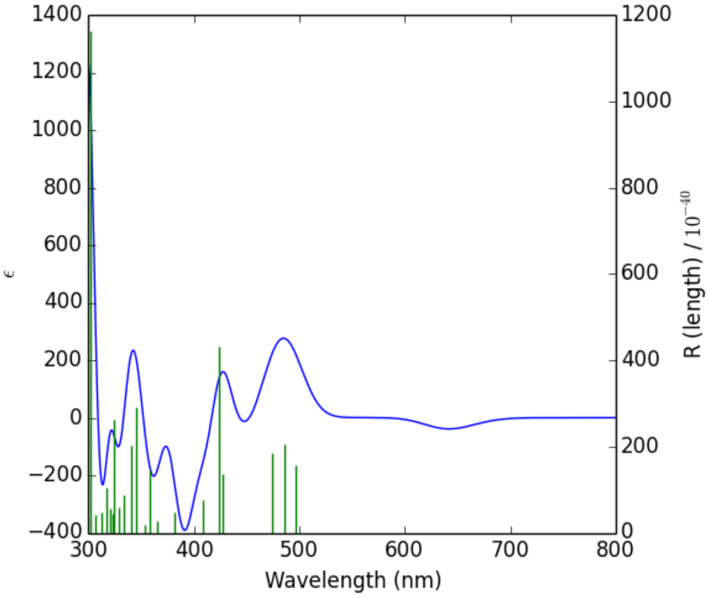

Fig. S47. Calculated (M06-2X/6-31G(d,p)) CD spectrum of (*P*)[4](*M*)[6]-**1c** in DCM.

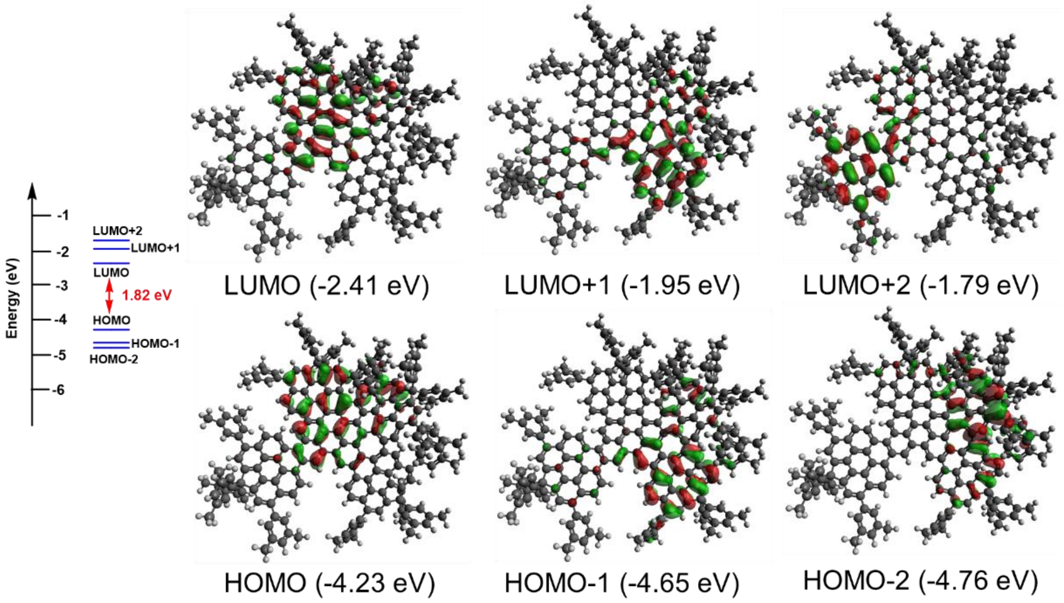

Fig. S48. Energy diagram and frontier molecular orbital profiles of **1d** obtained by B3LYP-D3/6-31g(d,p) level calculation (isovalue = 0.02). The substituents are replaced by methyl groups during the calculations.

Table S5. Selected TD-DFT (B3LYP-D3/6-31G(d,p)) calculated wavelength, oscillator strength and compositions of major electronic transitions of **1d**.

Wavelength (nm)	Osc. Strength (*f*)	Major contribs
**792.1806**	0.9797	HOMO->LUMO (99%)
**661.3196**	0.0484	H-1->LUMO (68%), HOMO->L+1 (19%)
**630.8986**	0.1764	H-2->LUMO (14%), H-1->LUMO (30%), HOMO->L+1 (46%)
**628.0861**	0.1692	H-3->LUMO (24%), H-2->LUMO (39%), HOMO->L+1 (32%)
**616.9596**	0.0468	H-3->LUMO (59%), H-2->LUMO (33%)
**582.6872**	0.4005	HOMO->L+2 (87%)
**553.2539**	0.0545	H-4->LUMO (76%)
**539.6483**	0.0558	H-4->LUMO (10%), HOMO->L+3 (62%), HOMO->L+4 (11%)
**527.9967**	0.1168	H-1->L+1 (41%), HOMO->L+3 (21%), HOMO->L+4 (31%)
**523.5598**	0.0953	H-1->L+1 (43%), HOMO->L+4 (43%)
**509.8663**	0.0239	H-3->L+1 (94%)
**491.8446**	0.0828	H-2->L+1 (86%)
**489.2053**	0.0124	HOMO->L+5 (90%)
**481.7539**	0.0882	H-1->L+2 (77%)
**472.6629**	0.0033	H-6->LUMO (18%), HOMO->L+6 (57%)
**468.8379**	0.3076	H-2->L+2 (73%), H-1->L+2 (11%)
**457.2532**	0.1267	H-1->L+3 (47%), H-1->L+4 (21%)
**455.2719**	0.0638	H-3->L+2 (60%), HOMO->L+6 (11%)

**452.365**	0.0496	H-5->LUMO (11%), H-4->L+1 (22%), H-3->L+2 (21%), H-1->L+3 (20%)
**447.2896**	0.0306	H-6->LUMO (17%), H-1->L+4 (13%), HOMO->L+8 (43%)
**445.3615**	0.0241	H-6->LUMO (10%), H-5->LUMO (30%), H-4->L+1 (17%)
**440.77**	0.0788	H-7->LUMO (17%), H-4->L+1 (12%), H-1->L+4 (18%), HOMO->L+7 (20%), HOMO->L+8 (16%)
**439.3175**	0.1542	H-3->L+3 (45%)
**437.0565**	0.2135	H-4->L+1 (15%), H-1->L+4 (19%), HOMO->L+7 (34%)
**430.1571**	0.2392	H-7->LUMO (19%), H-5->LUMO (28%), HOMO->L+7 (11%)
**426.6637**	0.2716	H-3->L+4 (12%), H-2->L+3 (28%), H-2->L+4 (45%)
**425.2879**	0.6895	H-7->LUMO (29%), H-6->LUMO (21%), H-3->L+4 (14%), HOMO->L+8 (12%)
**423.5156**	0.0803	H-3->L+4 (27%), H-2->L+3 (15%), H-1->L+5 (38%)
**422.2607**	0.1264	H-3->L+3 (14%), H-3->L+4 (13%), H-3->L+5 (13%), H-1->L+5 (30%)
**418.2156**	0.4	H-4->L+2 (63%)
**416.4037**	0.0447	H-3->L+5 (42%), H-2->L+3 (22%), H-2->L+4 (13%)
**414.996**	0.2229	H-4->L+2 (19%), H-3->L+5 (18%), H-2->L+3 (10%), H-2->L+4 (28%)
**408.2053**	0.2039	H-8->LUMO (35%), HOMO->L+9 (19%)
**406.9459**	0.0201	H-11->LUMO (11%), HOMO->L+9 (59%)
**404.5953**	0.0207	H-10->LUMO (10%), H-9->LUMO (37%), H-8->LUMO (22%)
**400.8671**	0.1067	H-11->LUMO (13%), H-10->LUMO (14%), H-9->LUMO (42%)
**399.9619**	0.0331	H-4->L+3 (10%), H-1->L+6 (46%)
**396.7621**	0.1695	H-2->L+5 (58%)
**392.6533**	0.0696	H-14->LUMO (10%), H-13->LUMO (12%), H-12->LUMO (22%), H-10->LUMO (29%)
**389.202**	0.1793	H-4->L+3 (51%), H-4->L+4 (14%)

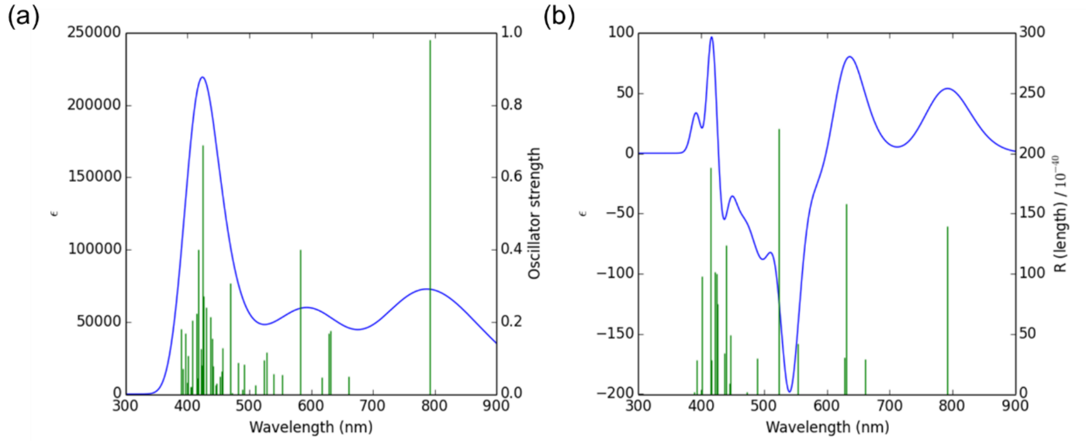

Fig. S49. Calculated (B3LYP-D3/6-31G(d,p)) (a) absorption spectrum of **1d**, (b) CD spectrum of (*M*)**-1d** in DCM.

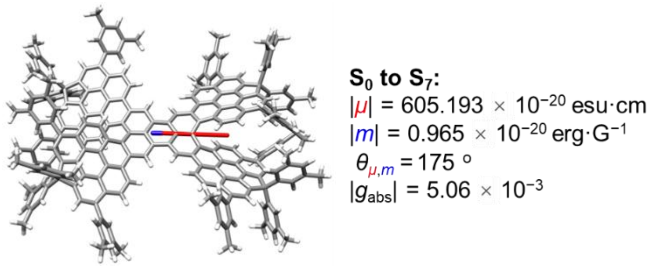

Fig. S50. Calculated transition dipole moments for S_0 to S_7 of **1a-*rac***. The μ vector is shown in red, and the *m* vector is shown in blue. The length of the μ vector is reduced 100 times when the length of the *m* vector is amplified 10 times for clarity. Calculated by TD-DFT at the B3LYP-D3-6-31G(d,p) level.

5. X-ray crystallographic analysis

Crystallographic data have been deposited with the Cambridge Crystallographic Data Centre as supplementary publication no. CCDC 2491985 for **3**, no. CCDC 2491986 for **4,** no. CCDC 2491987 for **7**, no. CCDC 2491988 for **1a-*meso***, no. CCDC 2491989 for **1b** and no. CCDC 2491990 for **1c**. The single crystal X-ray diffraction studies were performed at low temperature (T=100 K or 106 K) by using a four circles goniometer Kappa geometry, Bruker AXS D8 Venture, equipped with a Photon 100 CMOS active pixel sensor detector. Frames were integrated with the Bruker SAINT8 software package. Data were corrected for absorption effects using the multi-scan method (SADABS).[8] The structures were solved with the software SHELXT,[9] using a Dual Space method. Refinement of the structures were performed by least squares procedures on weighted F2 values using the SHELXLversion 2014/6[10] included in the WinGx system programs for Windows.[11] For more detailed information about diffraction data collection and refinement parameters, see Table S7 for **3**, Table S8 for **4**, Table S9 for **7**, Table S10 for **1a-*meso***, Table S11 for **1b**, Table S12 for **1c**.

Table S6. Crystal data and structure refinement for **3**.

Identification code	CCDC2491985
Empirical formula	$C_{77}H_{92}$
Formula weight	1017.50
Temperature/K	100.00
Crystal system	triclinic
Space group	P-1
a/Å	10.7993(4)
b/Å	17.2568(6)

c/Å	17.2568(6)
α/°	98.46
β/°	93.24
γ/°	93.24
Volume/$Å^3$	3169.0(2)
Z	2
$\rho_{calc}g/cm^3$	1.066
μ/mm^{-1}	0.441
F(000)	1108.0
Crystal size/mm^3	0.647 × 0.126 × 0.09
Radiation	CuKα (λ = 1.54178)
2Θ range for data collection/°	5.188 to 133.998
Index ranges	$-11 \leq h \leq 12$, $-20 \leq k \leq 20$, $-20 \leq l \leq 20$
Reflections collected	70008
Independent reflections	11042 [R_{int} = 0.0711, R_{sigma} = 0.0532]
Data/restraints/parameters	11042/1216/1093
Goodness-of-fit on F^2	1.030
Final R indexes [I>=2σ (I)]	R_1 = 0.0703, wR_2 = 0.1748
Final R indexes [all data]	R_1 = 0.0962, wR_2 = 0.1946
Largest diff. peak/hole / e $Å^{-3}$	0.60/-0.50

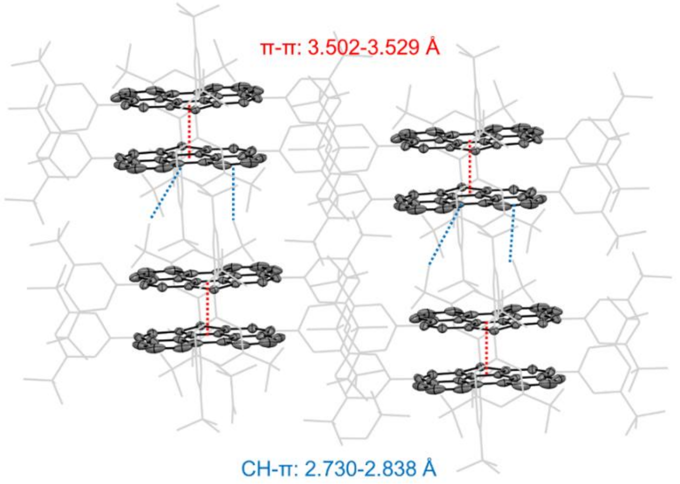

Fig. S51. 2D crystal-packing structure of **3** showing close π-π contacts and [CH···π] interactions.

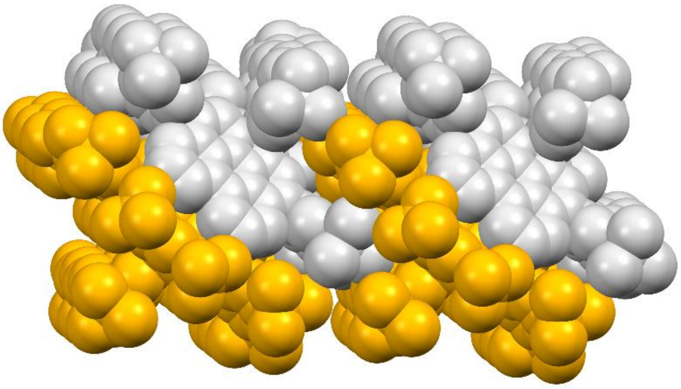

Fig. S52. 3D packing structure of **3** with space-filling model.

Table S7. Crystal data and structure refinement for **4**.

Identification code	CCDC2491986
Empirical formula	$C_{77.5}H_{92.5}BrCl_{0.5}O_{0.25}$
Formula weight	1134.26
Temperature/K	106.00
Crystal system	triclinic
Space group	P-1
a/Å	17.0845(5)
b/Å	18.9701(5)
c/Å	23.5086(7)
α/°	91.828(2)
β/°	92.765(2)
γ/°	111.670(2)
Volume/Å^3	7062.1(4)
Z	4
ρ_{calc}g/cm^3	1.067
μ/mm^{-1}	1.336
F(000)	2428.0
Crystal size/mm^3	0.133 × 0.127 × 0.109
Radiation	CuKα (λ = 1.54178)
2Θ range for data collection/°	3.768 to 133.628
Index ranges	$-20 \leq h \leq 19$, $-22 \leq k \leq 22$, $-28 \leq l \leq 27$
Reflections collected	179106
Independent reflections	24751 [R_{int} = 0.0522, R_{sigma} = 0.0302]
Data/restraints/parameters	24751/406/1635

Goodness-of-fit on F^2	1.026
Final R indexes [I>=2σ (I)]	R_1 = 0.0544, wR_2 = 0.1396
Final R indexes [all data]	R_1 = 0.0639, wR_2 = 0.1454
Largest diff. peak/hole / e Å^{-3}	0.74/-0.77

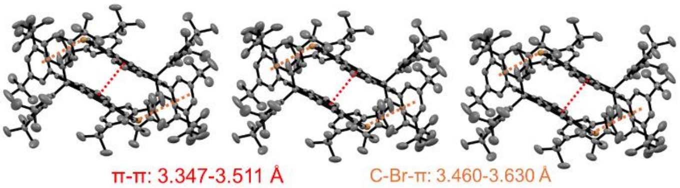

Fig. S53. 2D crystal-packing structure of **4** showing close π-π contacts and [C-Br···π] interactions.

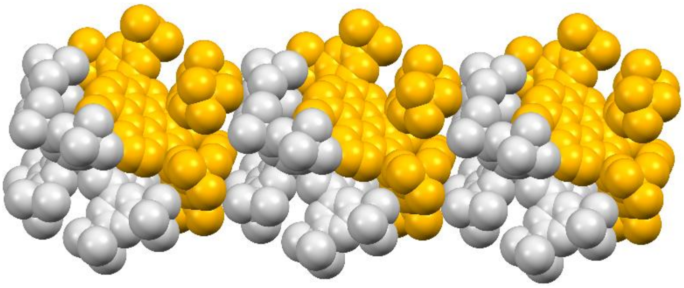

Fig. S54. 3D packing structure of **4** with space-filling model.

Table S8. Crystal data and structure refinement for **7**.

Identification code	CCDC2491987
Empirical formula	$C_{346}H_{404}Cl_{36}$
Formula weight	5838.87
Temperature/K	100.00
Crystal system	triclinic
Space group	P-1
a/Å	15.7143(11)
b/Å	20.9969(12)
c/Å	25.8490(17)
α/°	99.950(4)
β/°	93.203(5)
γ/°	90.623(4)
Volume/Å^3	8385.7(9)
Z	1
ρ_{calc}g/cm^3	1.156

μ/mm^{-1}	3.053
F(000)	3092.0
Crystal size/mm^3	0.96 × 0.108 × 0.071
Radiation	CuKα (λ = 1.54178)
2Θ range for data collection/°	5.02 to 134.34
Index ranges	-16 ≤ h ≤ 18, -24 ≤ k ≤ 24, -29 ≤ l ≤ 30
Reflections collected	137494
Independent reflections	29009 [R_{int} = 0.1518, R_{sigma} = 0.1682]
Data/restraints/parameters	29009/328/1712
Goodness-of-fit on F^2	1.194
Final R indexes [I>=2σ (I)]	R_1 = 0.1320, wR_2 = 0.3579
Final R indexes [all data]	R_1 = 0.1893, wR_2 = 0.3962
Largest diff. peak/hole / e Å^{-3}	0.73/-0.64

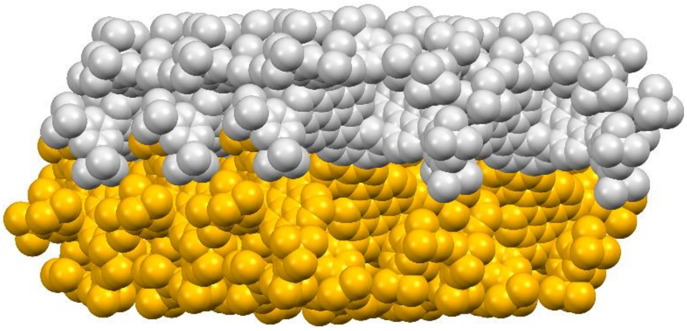

Fig. S55. 3D packing structure of **7** with space-filling model.

Table S9. Crystal data and structure refinement for **1a-*meso***.

Identification code	CCDC2491988
Empirical formula	$C_{400}H_{412}Cl_{24}$
Formula weight	6070.07
Temperature/K	100.00
Crystal system	triclinic
Space group	P-1
a/Å	13.7074(14)
b/Å	25.091(3)
c/Å	26.998(3)
α/°	68.915(9)
β/°	75.476(8)

γ/°	80.514(9)
Volume/Å^3	8358.2(18)
Z	1
ρ_{calc}g/cm^3	1.206
μ/mm^{-1}	2.225
F(000)	3220.0
Crystal size/mm^3	0.151 × 0.092 × 0.06
Radiation	CuKα (λ = 1.54178)
2Θ range for data collection/°	6.024 to 133.18
Index ranges	$-16 \le h \le 15$, $-29 \le k \le 27$, $-32 \le l \le 32$
Reflections collected	115713
Independent reflections	28666 [R_{int} = 0.2511, R_{sigma} = 0.2120]
Data/restraints/parameters	28666/3304/1525
Goodness-of-fit on F^2	0.892
Final R indexes [I>=2σ (I)]	R_1 = 0.1669, wR_2 = 0.3830
Final R indexes [all data]	R_1 = 0.4133, wR_2 = 0.5735
Largest diff. peak/hole / e Å^{-3}	0.36/-0.27

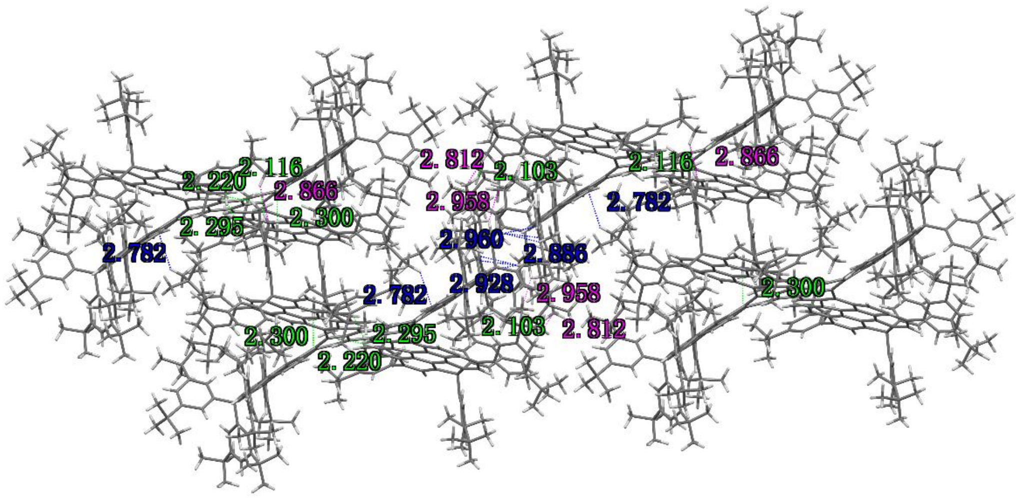

Fig. S56. 2D crystal-packing structure of **1a-*meso*** showing close [C-H···π], [C-H···C-H] and [C-H···C] interactions, labeled in blue, green and magenta, respectively.

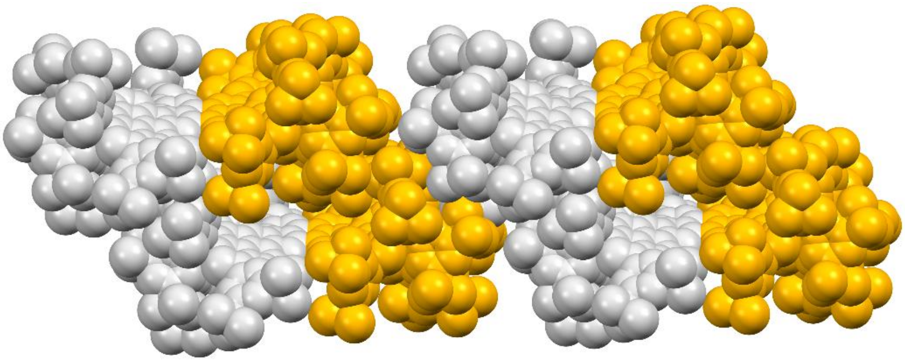

Fig. S57. 3D packing structure of **1a-*meso*** with space-filling model.

Table S10. Crystal data and structure refinement for **1b**.

Identification code	CCDC2491989
Empirical formula	$C_{344.89}H_{378.68}Cl_{3.74}$
Formula weight	4656.30
Temperature/K	100.00
Crystal system	triclinic
Space group	P-1
a/Å	14.4516(13)
b/Å	28.598(2)
c/Å	39.034(3)
α/°	101.304(6)
β/°	91.702(6)
γ/°	101.051(6)
Volume/Å^3	15488(2)
Z	2
ρ_{calc}g/cm^3	0.998
μ/mm^{-1}	0.705
F(000)	5023.0
Crystal size/mm^3	0.403 × 0.078 × 0.059
Radiation	CuKα (λ = 1.54178)
2Θ range for data collection/°	4.332 to 134.072
Index ranges	$-17 \leq h \leq 17$, $-33 \leq k \leq 30$, $-46 \leq l \leq 46$
Reflections collected	283312
Independent reflections	54159 [R_{int} = 0.1473, R_{sigma} = 0.1060]
Data/restraints/parameters	54159/4718/4685
Goodness-of-fit on F^2	1.186

Final R indexes [I>=2σ (I)]	R_1 = 0.1179, wR_2 = 0.3192
Final R indexes [all data]	R_1 = 0.1814, wR_2 = 0.3797
Largest diff. peak/hole / e Å^{-3}	1.17/-0.68

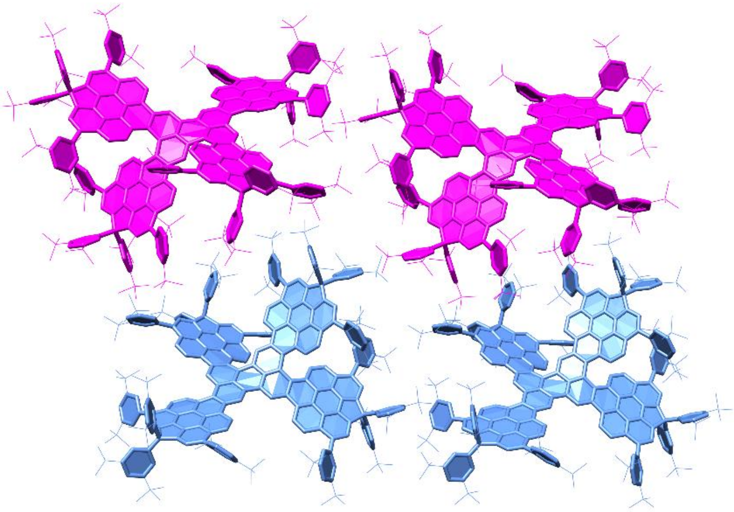

Fig. S58. 2D crystal-packing structure of **1b**.

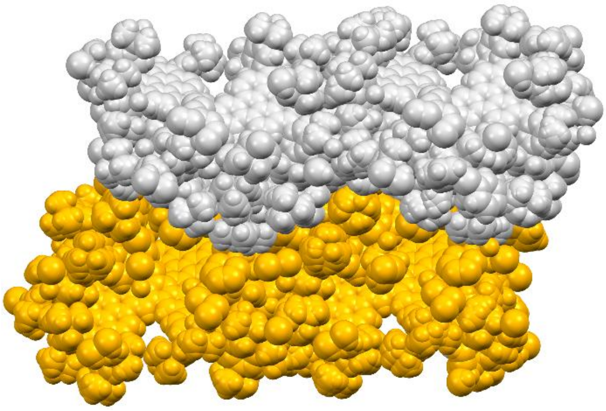

Fig. S59. 3D packing structure of **1b** with space-filling model.

Table S11. Crystal data and structure refinement for **1c**.

Identification code	CCDC2491990
Empirical formula	$C_{335.5}H_{372.5}Cl_5N_{2.5}$
Formula weight	4617.09
Temperature/K	100.00
Crystal system	triclinic
Space group	P-1

a/Å	19.5386(8)
b/Å	27.0718(13)
c/Å	31.4143(16)
α/°	67.712(3)
β/°	75.728(3)
γ/°	75.768(3)
Volume/Å^3	14683.9(13)
Z	2
ρ_{calc}g/cm^3	1.044
μ/mm^{-1}	0.845
F(000)	4976.0
Crystal size/mm^3	0.236 × 0.098 × 0.052
Radiation	CuKα (λ = 1.54178)
2Θ range for data collection/°	5.15 to 124.996
Index ranges	-22 ≤ h ≤ 22, -31 ≤ k ≤ 31, -34 ≤ l ≤ 34
Reflections collected	235034
Independent reflections	44305 [R_{int} = 0.2474, R_{sigma} = 0.1773]
Data/restraints/parameters	44305/3326/4000
Goodness-of-fit on F^2	1.003
Final R indexes [I>=2σ (I)]	R_1 = 0.1234, wR_2 = 0.3090
Final R indexes [all data]	R_1 = 0.3024, wR_2 = 0.4457
Largest diff. peak/hole / e Å^{-3}	0.54/-0.43

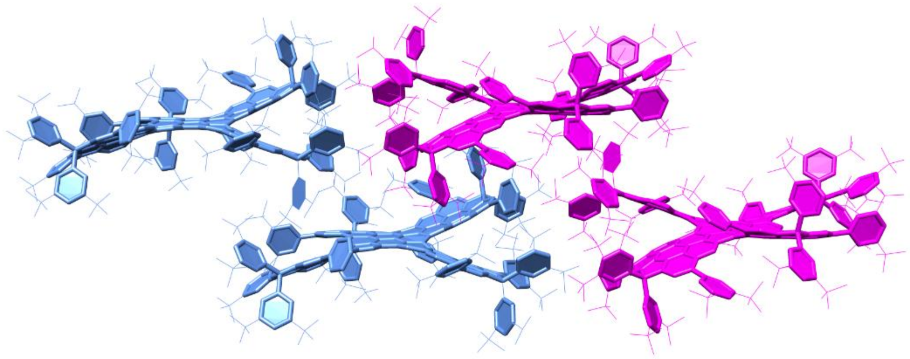

Fig. S60. 2D crystal-packing structure of **1c**.

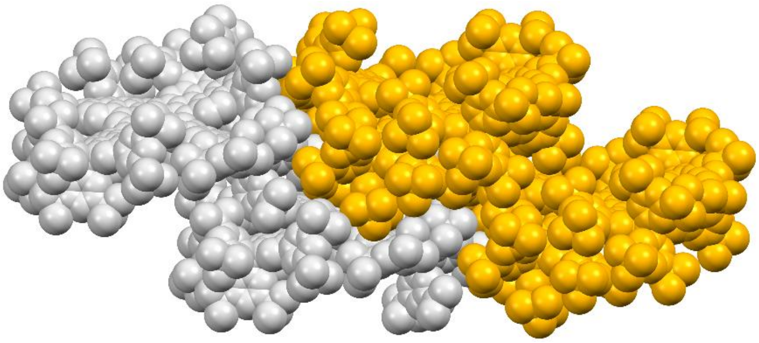

Fig. S61. 3D packing structure of **1c** with space-filling model.

6. References

[1] (a) W. Zeng, Q. Qi, J. Wu, *Sci. Bull.* **2015**, *60*, 1266–1271. (b) T. Shen, Y. Zou, X. Hou, H. Wei, L. Ren, L. Jiao, J. Wu, *Angew. Chem. Int. Ed.* **2023**, *62*, e202311928.

[2] J. M. Fisher, M. L. Williams, J. R. Palmer, N. E. Powers-Riggs, R. M. Young, M. R. Wasielewski, *J. Am. Chem. Soc.* **2024**, *146*, 9911−9919.

[3] *Gaussian 09; Revision A.2*; M. J. Frisch, G. W. Trucks, H. B. Schlegel, G. E. Scuseria, M. A. Robb, J. R. Cheeseman, G. Scalmani, V. Barone, B. Mennucci, G. A. Petersson, H. Nakatsuji, M. Caricato, X. Li, H. P. Hratchian, A. F. Izmaylov, J. Bloino, G. Zheng, J. L. Sonnenberg, M. Hada, M. Ehara, K. Toyota, R. Fukuda, J. Hasegawa, M. Ishida, T. Nakajima, Y. Honda, O. Kitao, H. Nakai, T. Vreven, J. J. A. Montgomery, J. E. Peralta, F. Ogliaro, M. Bearpark, J. J. Heyd, E. Brothers, K. N. Kudin, V. N. Staroverov, R. Kobayashi, J. Normand, K. Raghavachari, A. Rendell, J. C. Burant, S. S. Iyengar, J. Tomasi, M. Cossi, N. Rega, N. J. Millam, M. Klene, J. E. Knox, J. B. Cross, V. Bakken, C. Adamo, J. Jaramillo, R. Gomperts, R. E. Stratmann, O. Yazyev, A. J. Austin, R. Cammi, C. Pomelli, J. W. Ochterski, R. L. Martin, K. Morokuma, V. G. Zakrzewski, G. A. Voth, P. Salvador, J. J. Dannenberg, S. Dapprich, A. D. Daniels, Ö. Farkas, J. B. Foresman, J. V. Ortiz, J. Cioslowski, D. J. Fox, Gaussian, Inc., Wallingford CT, **2009**.

[4] (a) A. D. Becke, *J. Chem. Phys.* **1993**, *98*, 5648. (b) C. Lee, W. Yang, R. G. Parr, *Phys. Rev. B: Condens. Matter* **1988**, *37*, 785. (c) R. Ditchfield, W. J. Hehre, J. A. Pople, *J. Chem. Phys.* **1971**, *54*, 724. (d) W. J. Hehre, R. Ditchfield, J. A. Pople, *J. Chem. Phys.* **1972**, *56*, 2257. (e) P. C. Hariharan, J. A. Pople, *Theor. Chim. Acta* **1973**, *28*, 213.

[5] M. Caricato, A. Frisch, J. Hiscocks, M. J. Frisch. Gaussian 09, Revision d. 01, Gaussian. *Inc, Wallingford CT*, **2009,** *201.*

[6] T. Yanai, D. Tew, N. Handy, *Chem. Phys. Lett.* **2004**, *393*, 51.

[7] E. F. Pettersen, T. D. Goddard, C. C. Huang, G. S. Couch, D. M. Greenblatt, E. C. Meng, T. E. Ferrin, *J. Comput. Chem.* **2004**, *25*, 1605.

[8] SADABS. Ver. 2014/5. L. Krause, R. Herbs-Irmer, G. M. Sheldrick, D. Stalke, *J. Appl. Crystallogr.* **2015**, *48*.

[9] G. M. Sheldrick, *Sect. A*, *Acta Crystallogr.* **2015**, A71, 3.

[10] G. M. Sheldrick, Ver. 2014/7. *Acta Crystallographica. Sect C Structural Chemistry* 71, 3.

[11] L. J. Farrugia, *J. Appl. Cryst.* **1999**, *32*, 837.

7. Appendix I: NMR and HR-mass spectra of all new compounds

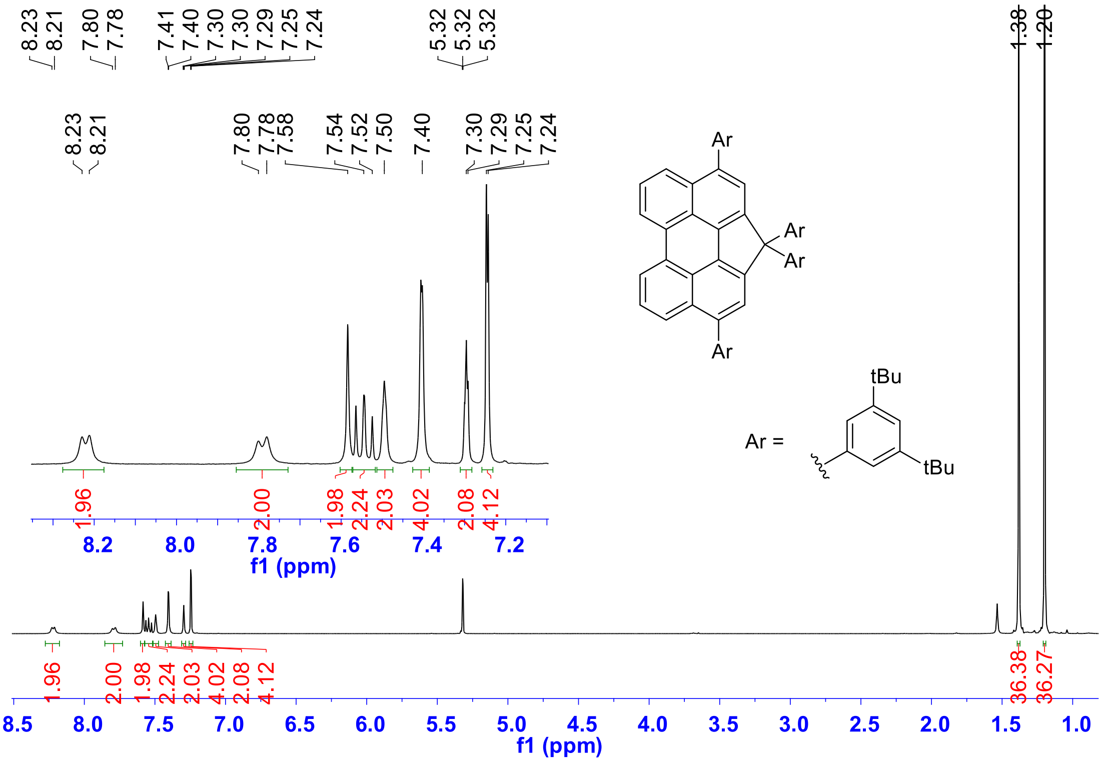

Fig. S62. ^{1}H NMR spectrum of **3** (400 MHz, CD_2Cl_2, 298 K).

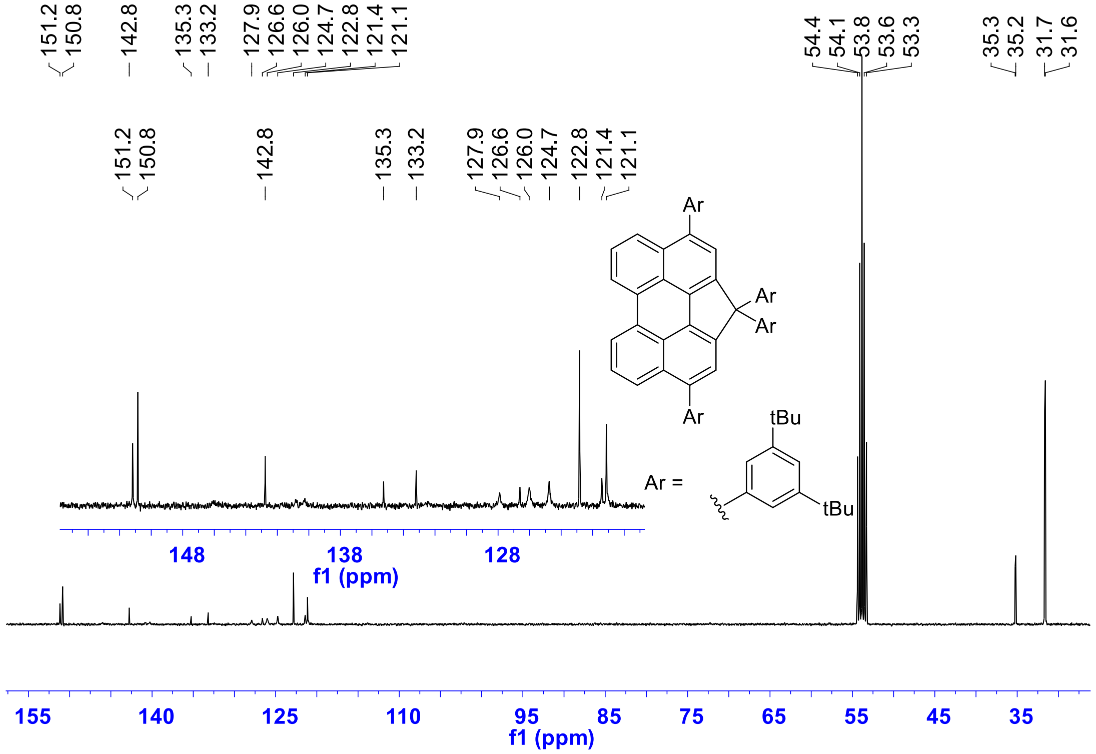

Fig. S63. ^{13}C NMR spectrum of **3** (100 MHz, CD_2Cl_2, 298 K).

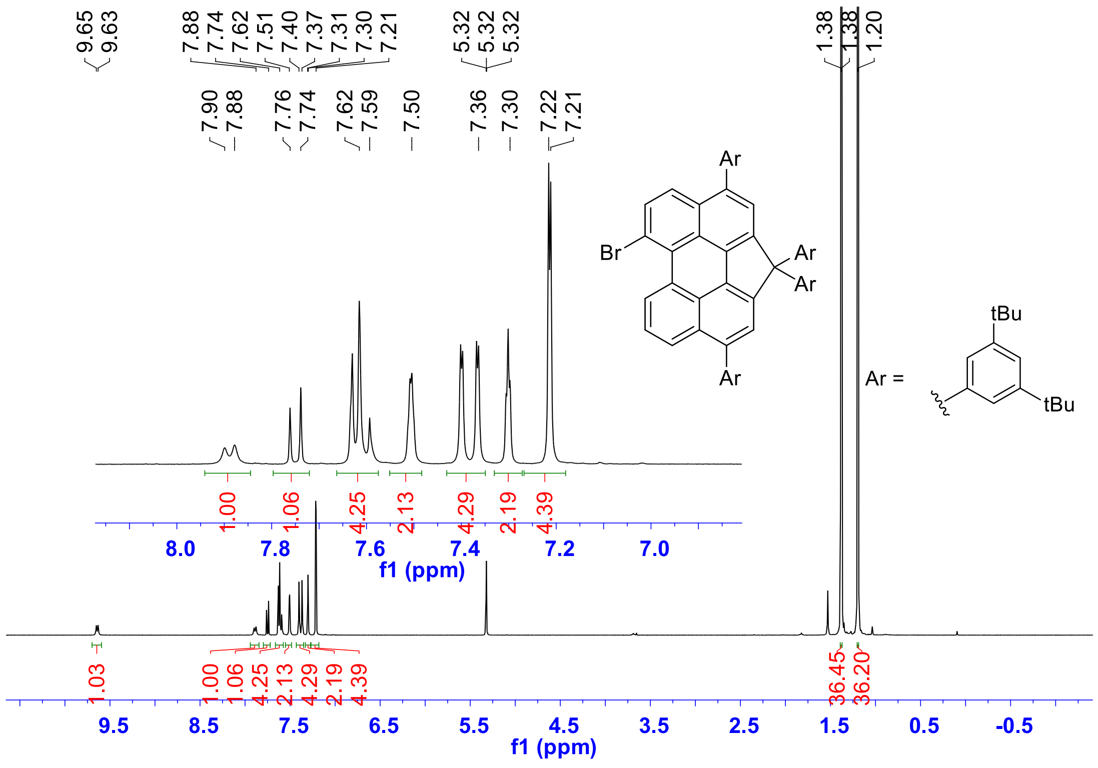

Fig. S64. ^{1}H NMR spectrum of **4** (400 MHz, CD_2Cl_2, 298 K).

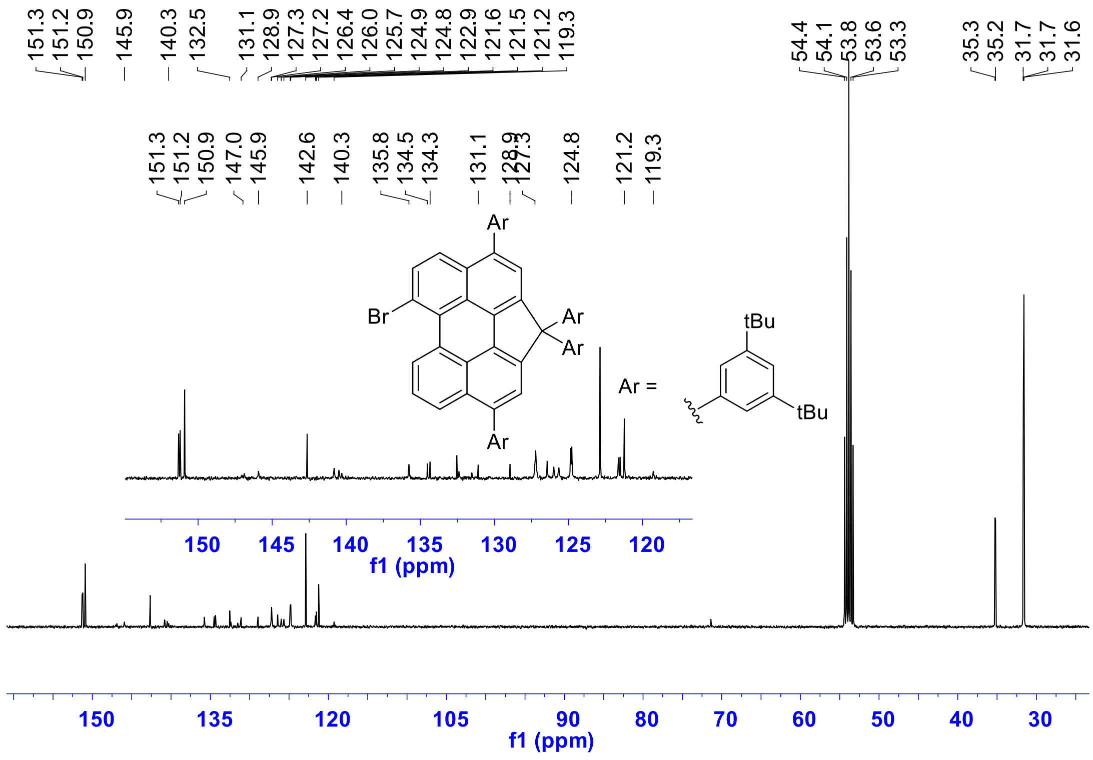

Fig. S65. ^{13}C NMR spectrum of **4** (100 MHz, CD_2Cl_2, 298 K).

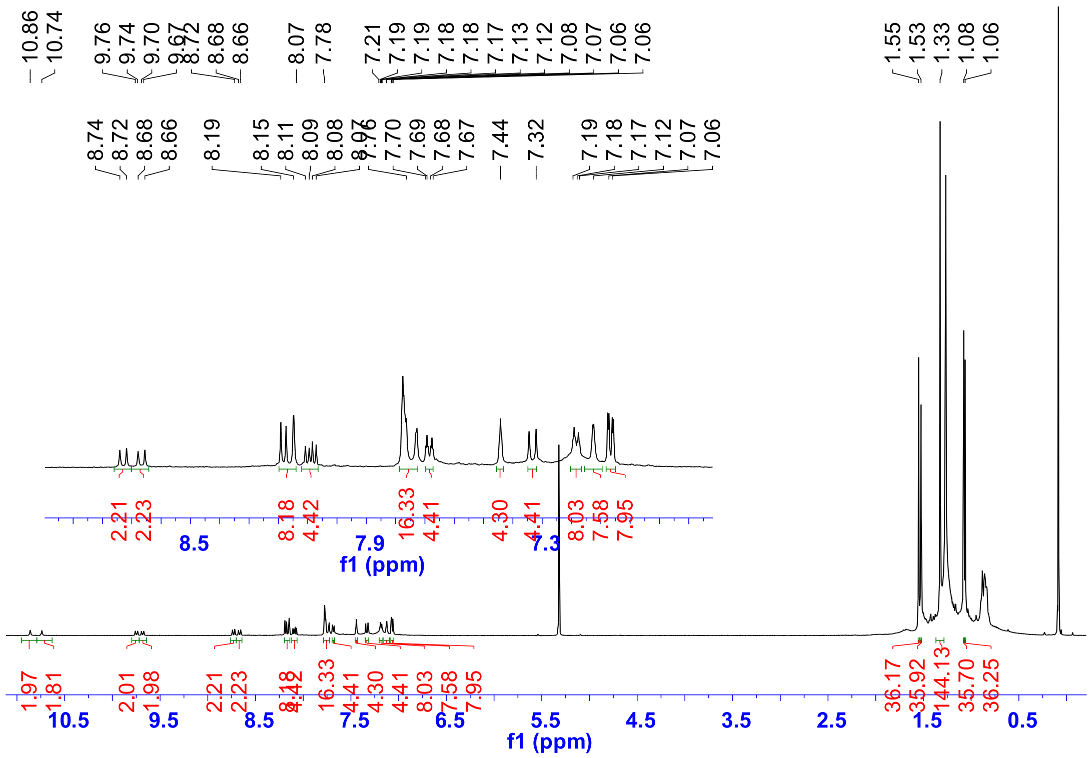

Fig. S66. ^{1}H NMR spectrum of **1a** (400 MHz, CD_2Cl_2, 298 K).

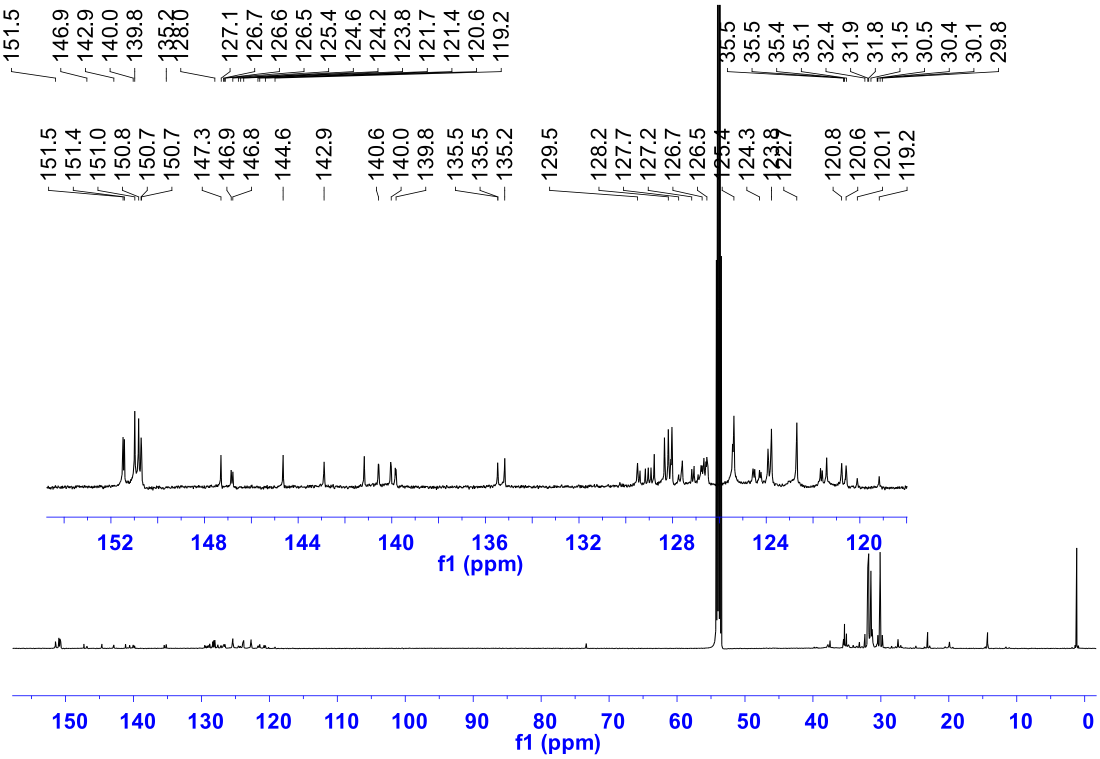

Fig. S67. ^{13}C NMR spectrum of **1a** (150 MHz, CD_2Cl_2, 298 K).

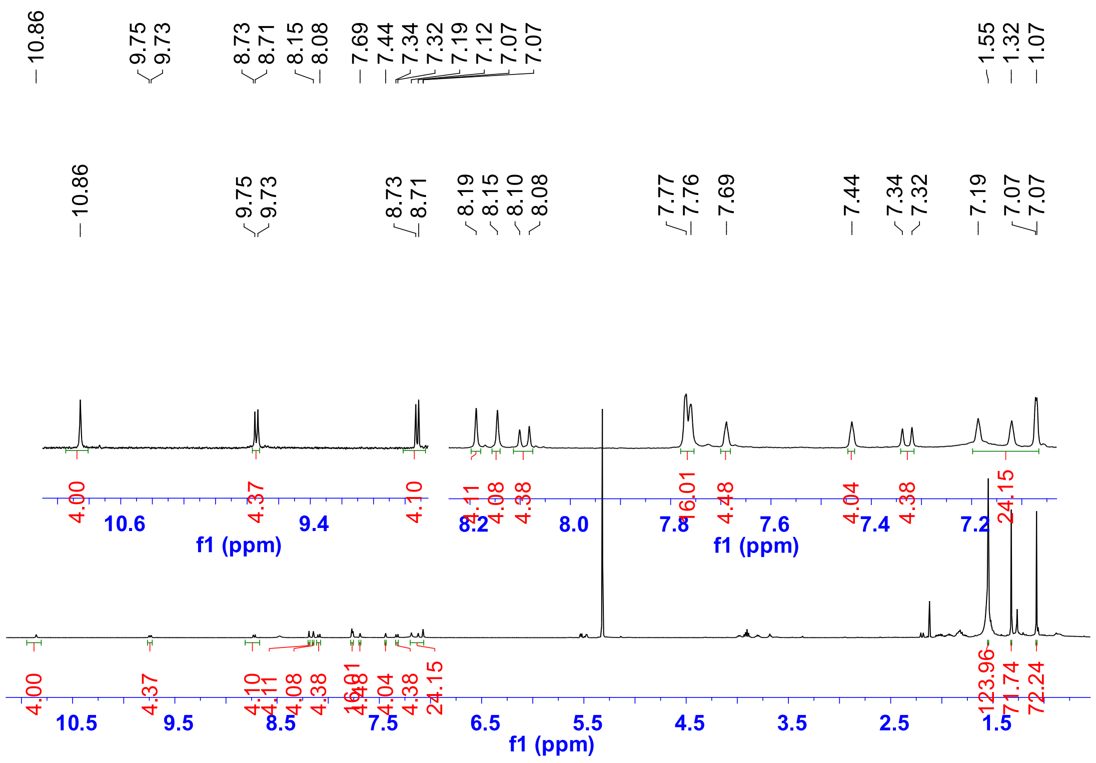

Fig. S68. ^{1}H NMR spectrum of **1a-*rac*** (500 MHz, CD_2Cl_2, 298 K).

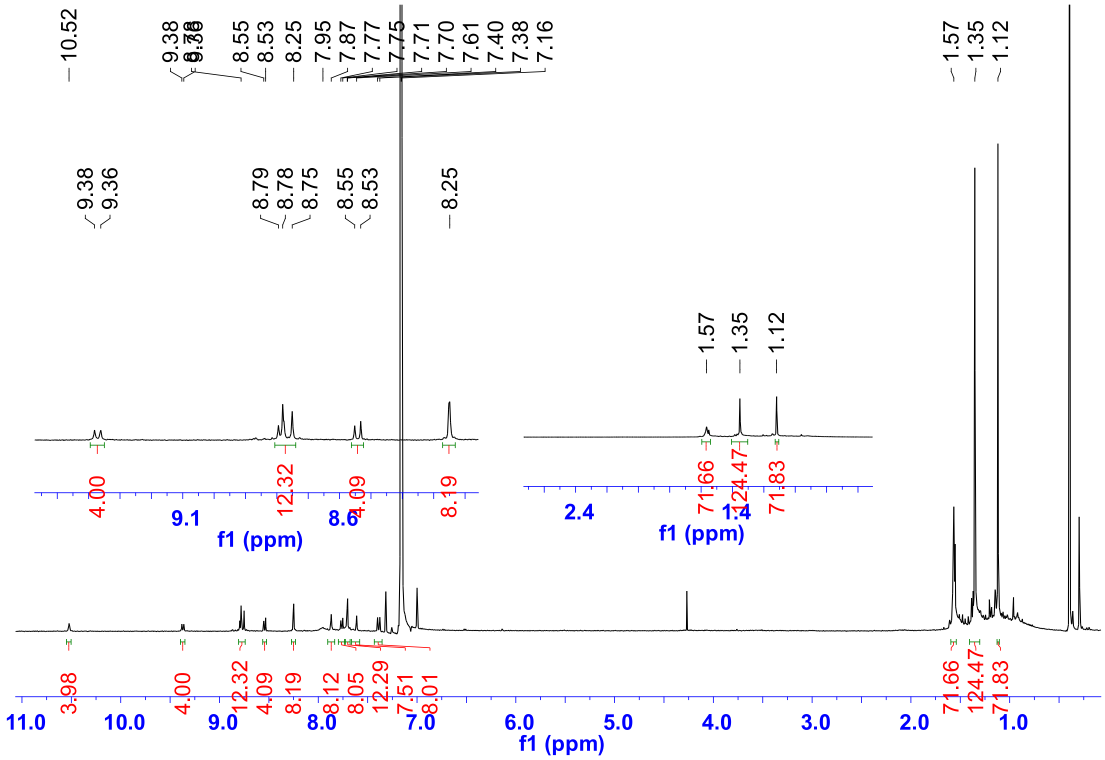

Fig. S69. ^{1}H NMR spectrum of **1a-*rac*** (500 MHz, C_6D_6, 298 K).

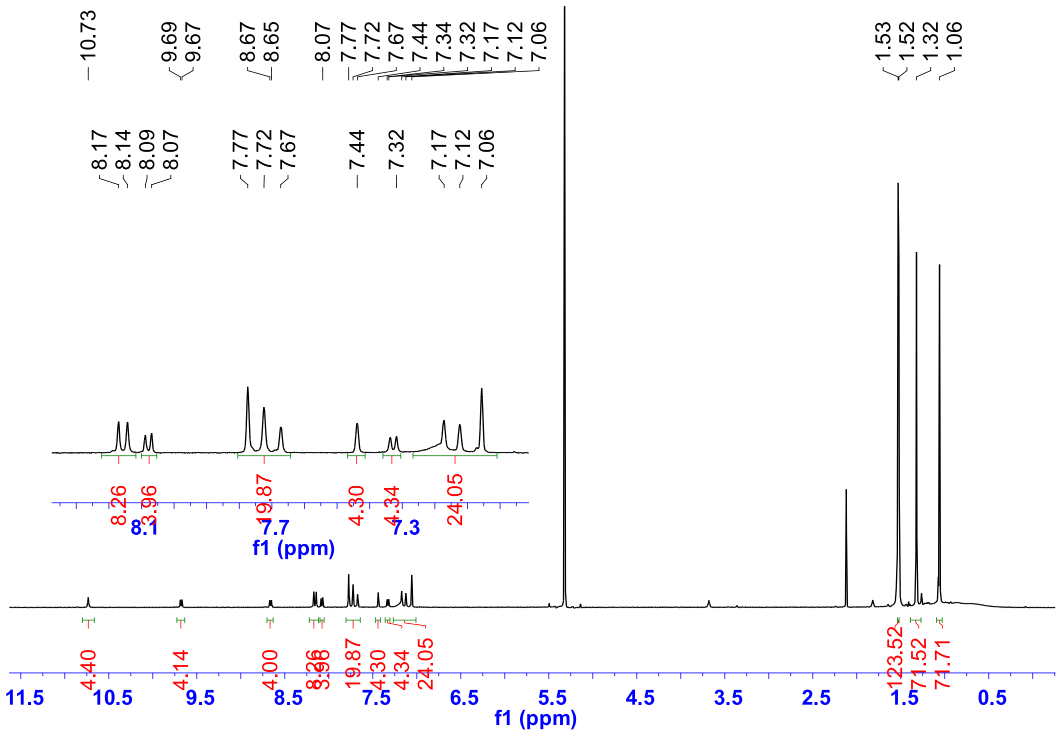

Fig. S70. ^{1}H NMR spectrum of **1a-*meso*** (500 MHz, CD_2Cl_2, 298 K).

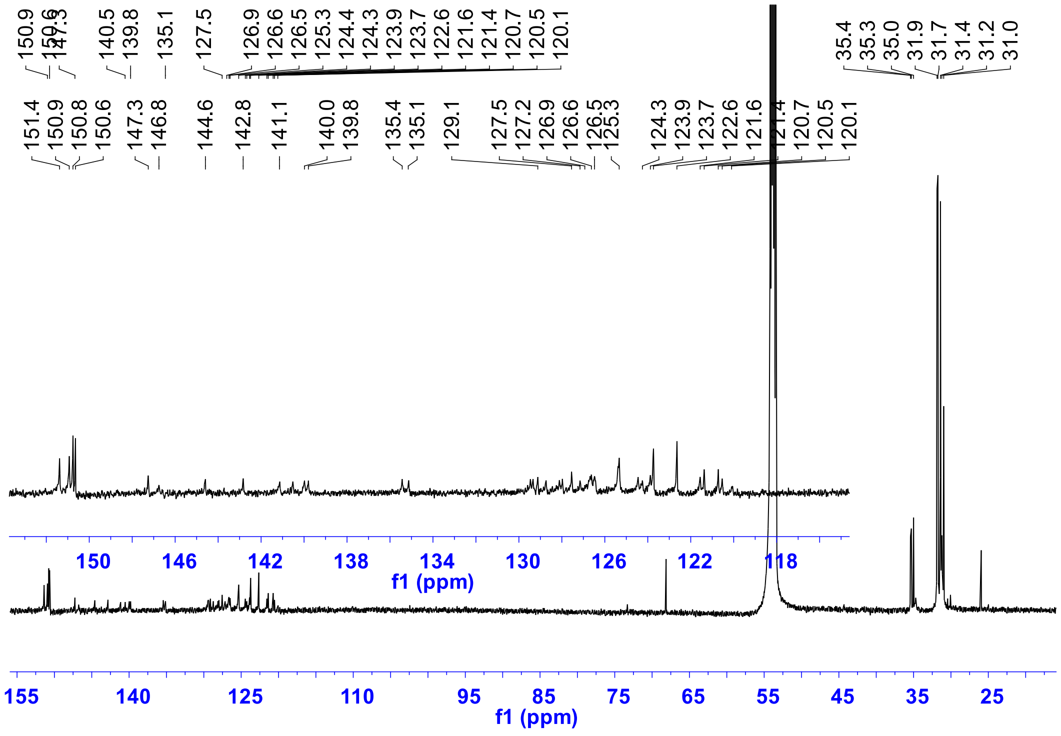

Fig. S71. ^{13}C NMR spectrum of **1a-*meso*** (150 MHz, CD_2Cl_2, 298 K).

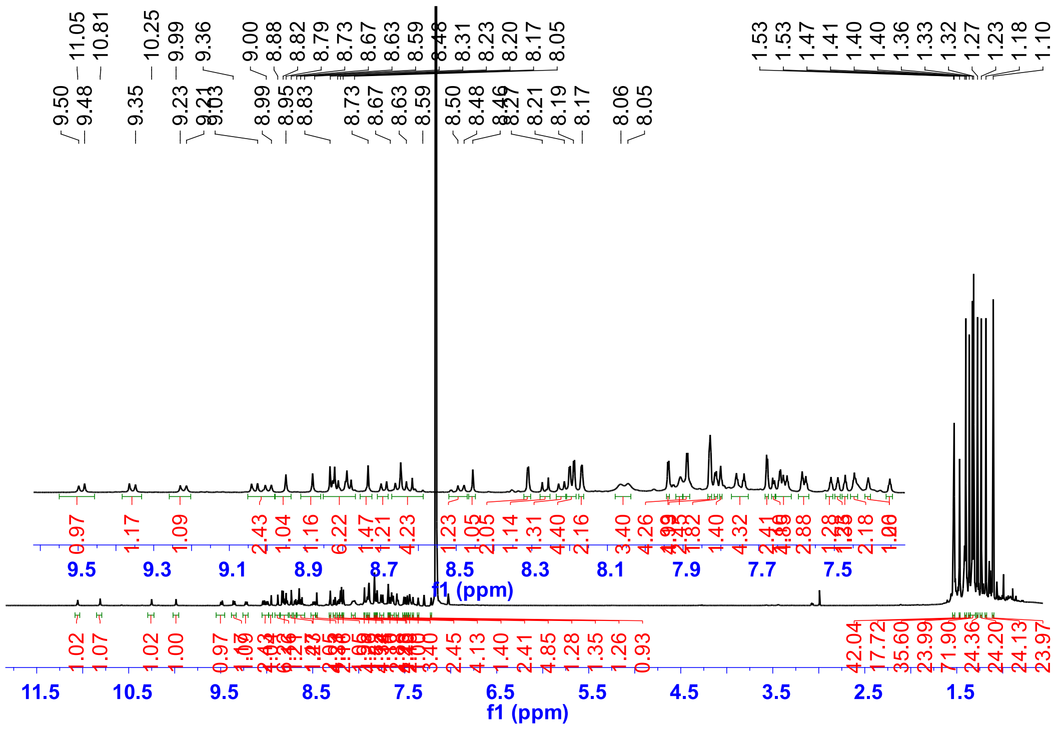

Fig. S72. ^{1}H NMR spectrum of **1b** (600 MHz, C_6D_6, 298 K).

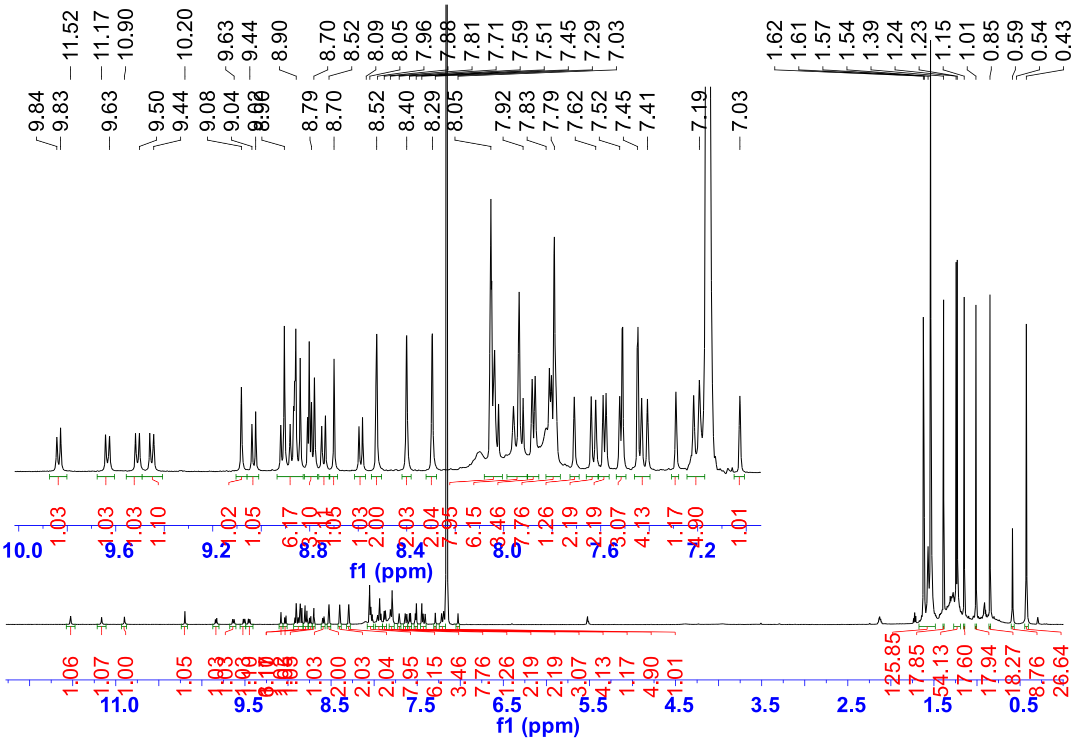

Fig. S73. ^{1}H NMR spectrum of **1c** (600 MHz, C_6D_6, 298 K).

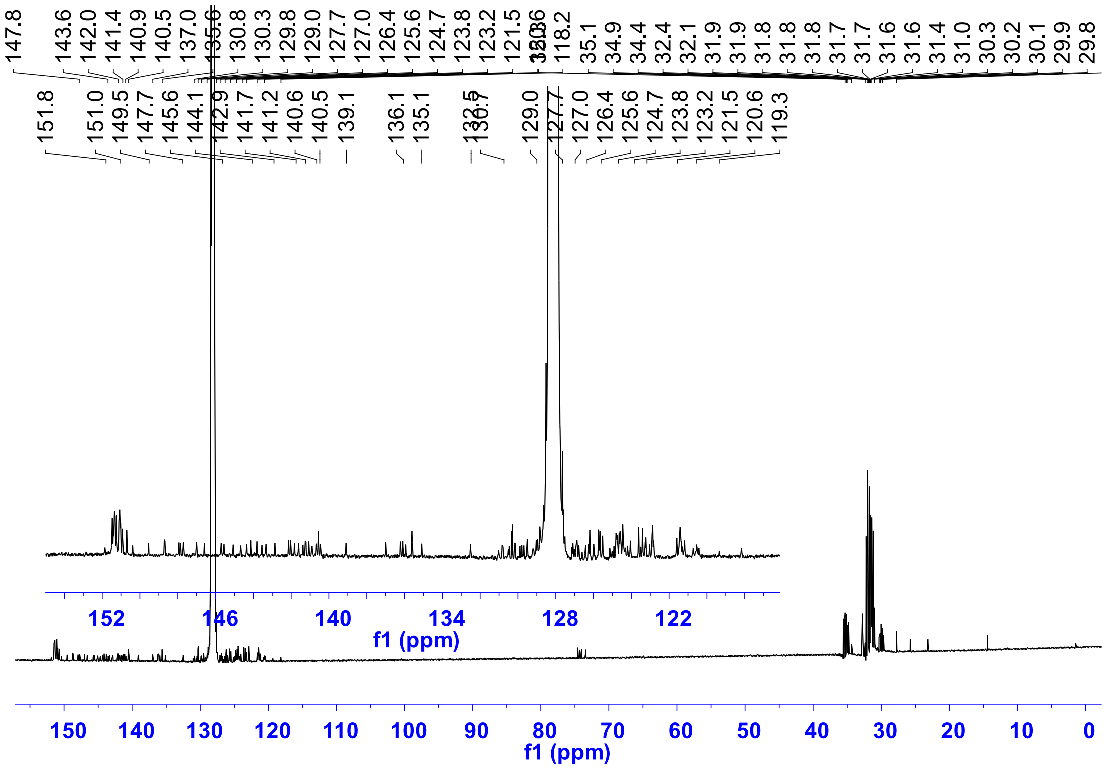

Fig. S74. ^{13}C NMR spectrum of **1c** (150 MHz, C_6D_6, 298 K).

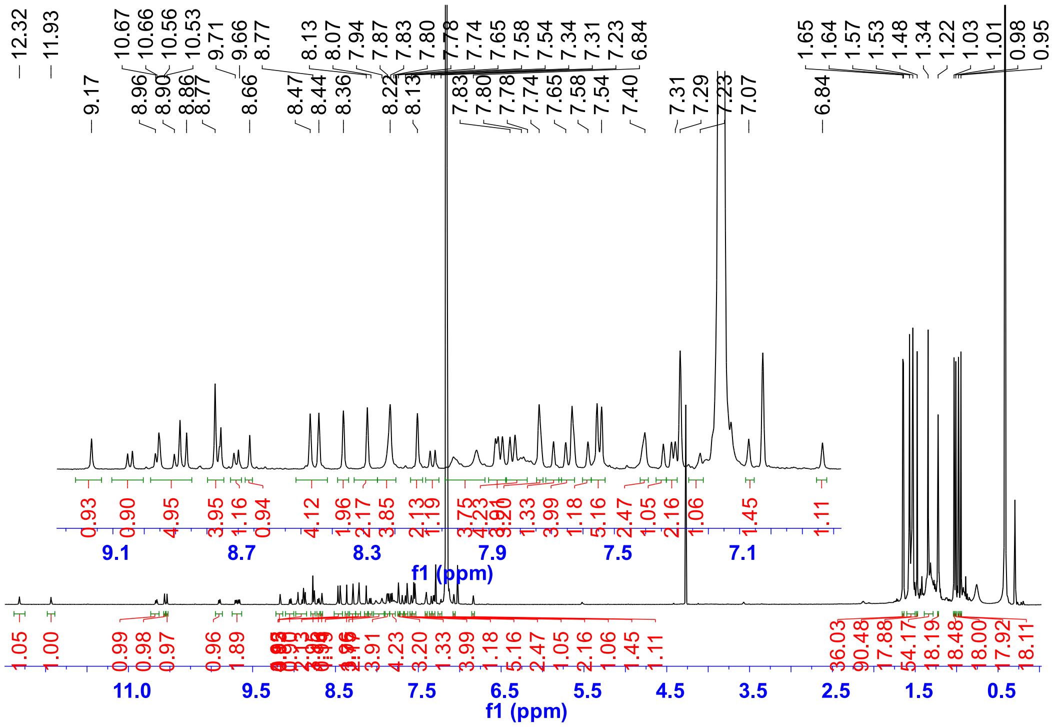

Fig. S75. ^{1}H NMR spectrum of **1d** (600 MHz, C_6D_6, 298 K).

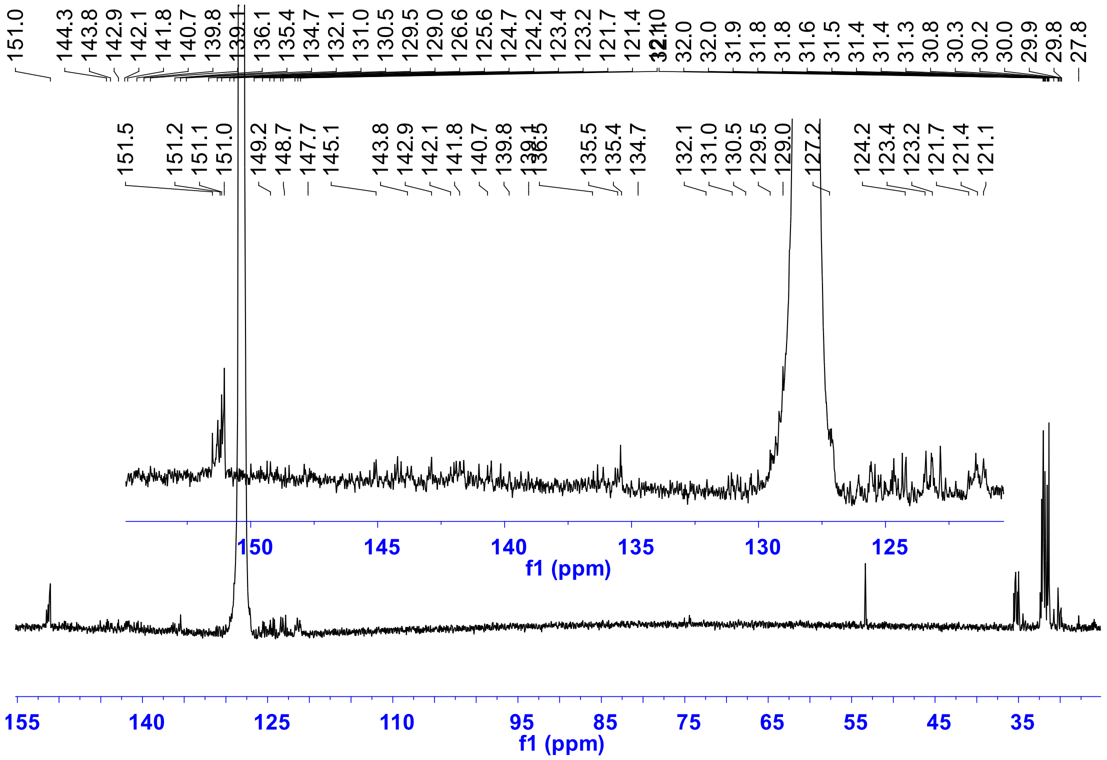

Fig. S76. ^{13}C NMR spectrum of **1d** (150 MHz, C_6D_6, 298 K). The low S/N ratio was due to the low concentration and existence of many *sp*^{2}-carbons.

Mass Spectrum SmartFormula Report

**Sample Name**	zqf-per	**Data File**	D:\MassHunter\Data\Chemistry\2025\202503\20250320-apci\zqf-per.d
**Instrument Name**	Agilent 6546 LC-QTOF	**IRM Calibration Status**	Success
**Acq Method**	MS Scan_union_APCI-3.m	**Acquired Time**	20/3/2025 11:42:43 AM (UTC+08:00)
**Comment**	Prof Wu Jishan	**Operator**	WLK

Meas. m/z	#	Formula	Calc. Mass	Err [ppm]
1017.7278	1	C77 H93	1017.7272	0.59

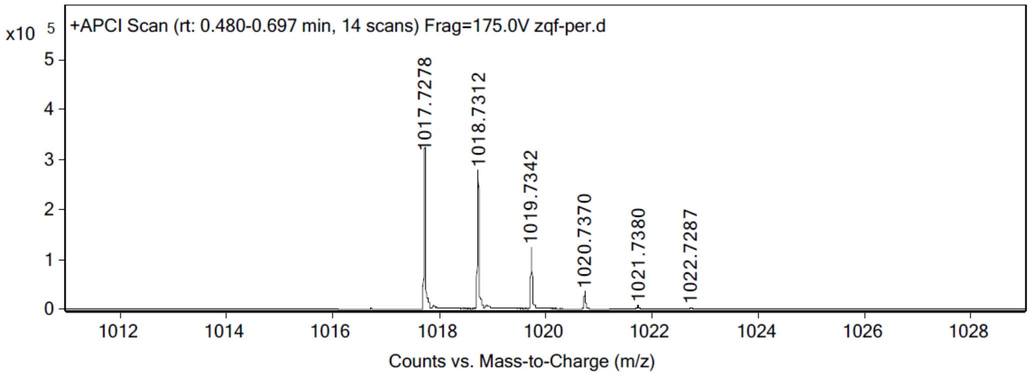

Page 1 of 1 Printed at 11:54 AM on 20-Mar-2025

Fig. S77. HR mass spectrum (APCI) of compound **3**.

Mass Spectrum SmartFormula Report

**Sample Name**	zqf-per-Br	**Data File**	D:\MassHunter\Data\Chemistry\2025\202503\20250320-apci\zqf-per-Br.d
**Instrument Name**	Agilent 6546 LC-QTOF	**IRM Calibration Status**	Success
**Acq Method**	MS Scan_union_APCI-3.m	**Acquired Time**	20/3/2025 11:56:03 AM (UTC+08:00)
**Comment**	Prof Wu Jishan	**Operator**	WLK

Meas. m/z	#	Formula	Calc. Mass	Err [ppm]
1095.6374	1	C77 H92 Br	1095.6377	0.27

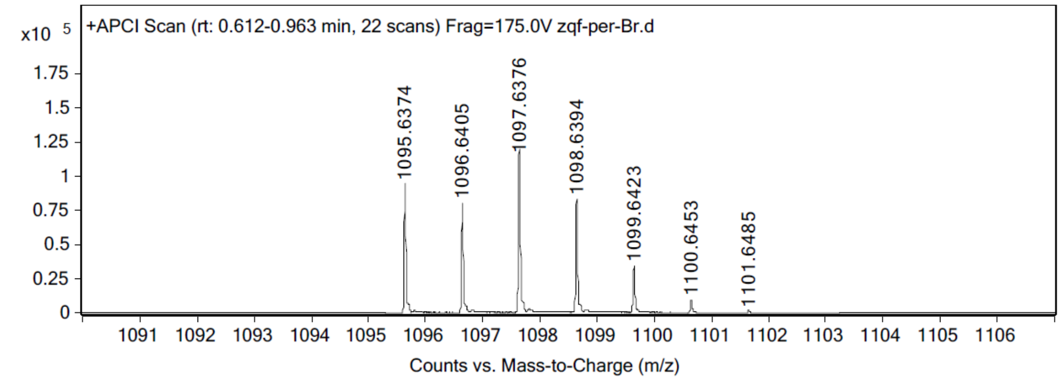

Printed at 12:03 PM on 20-Mar-2025

Fig. S78. HR mass spectrum (APCI) of compound **4**.

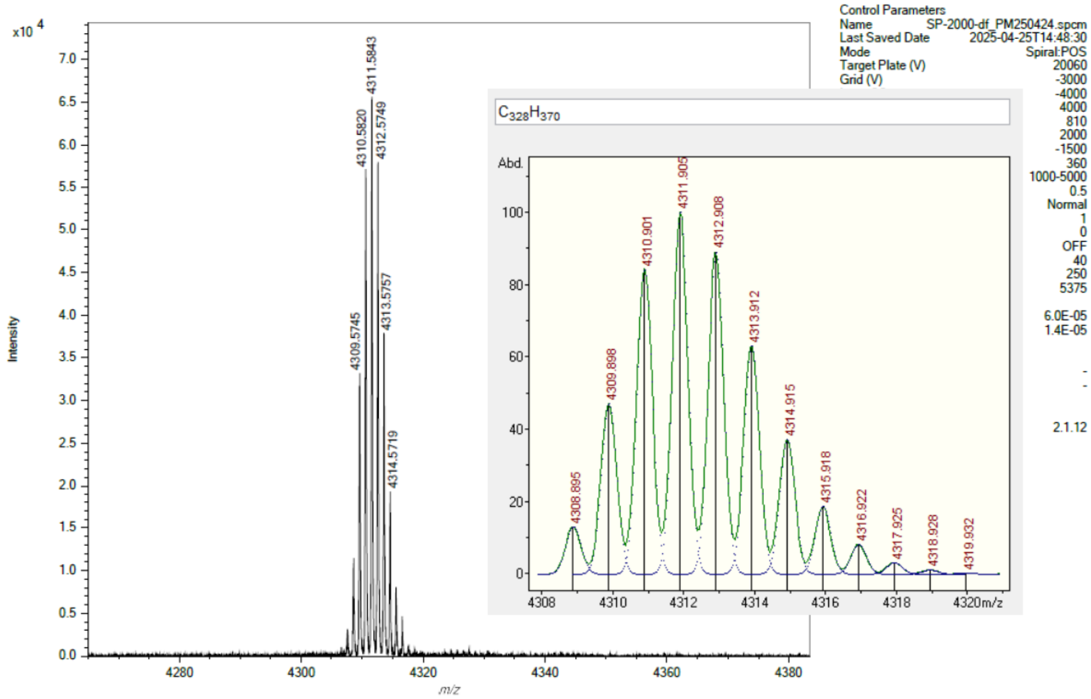

Acquired Date	2025-06-03T16:19:31
DataSet	Z:\PI_Wu Jishan\zqf\2025060301
Target Plate ID	001-0000000
Spot ID	O01
Spot Title	
Name	SP-O01-00-001
Description	5-per-4312

Fig. S79. HR mass spectrum (MALDI) of compound **6**.

Mass Spectrum Report

Sample Name	Compound **7**	Instrument Name	JEOL JMS-S3000 Sprial-TOF

**Meas. *m/z***	**Formula**	**Calc. Mass**	**Err [ppm]**
4306.8715	$C_{328}H_{368}$	4306.8791	-1.76

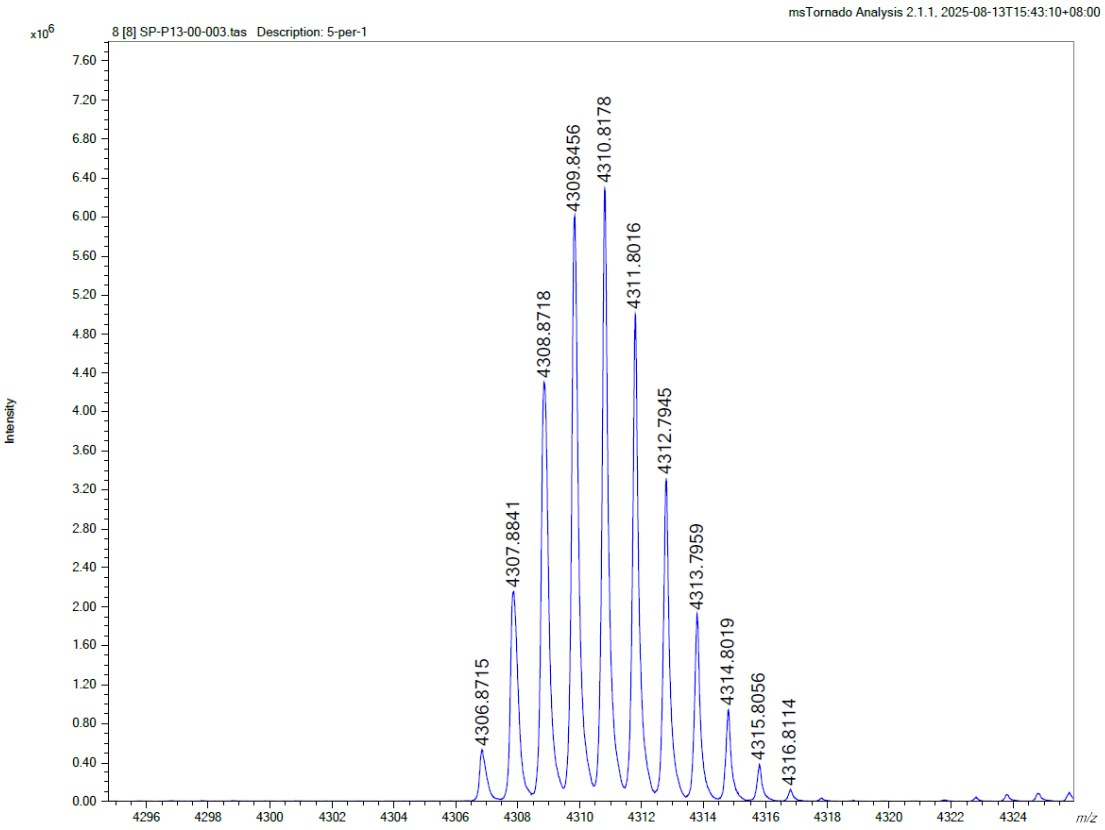

1/1

Fig. S80. HR mass spectrum (MALDI) of compound **7**.

Mass Spectrum Report

Sample Name	Compound **1a**	Instrument Name	JEOL JMS-S3000 Sprial-TOF
**Meas.** ***m*/*z***	**Formula**	**Calc. Mass**	**Err [ppm]**
4302.8394	$C_{328}H_{364}$	4302.8478	-1.95

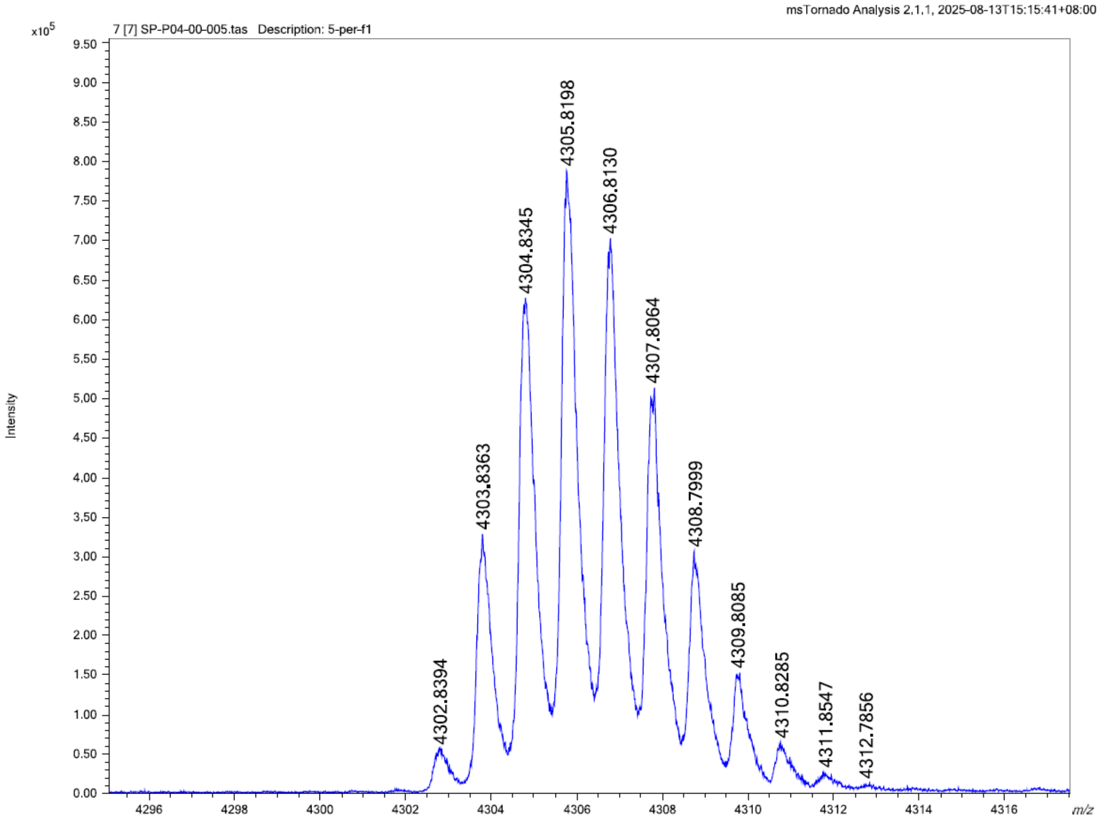

1/1

Fig. S81. HR mass spectrum (MALDI) of compound **1a**.

Mass Spectrum Report

Sample Name	Compound **1a**-*rac*	Instrument Name	JEOL JMS-S3000 Sprial-TOF

**Meas.** ***m/z***	**Formula**	**Calc. Mass**	**Err [ppm]**
4302.8376	$C_{328}H_{364}$	4302.8478	-2.37

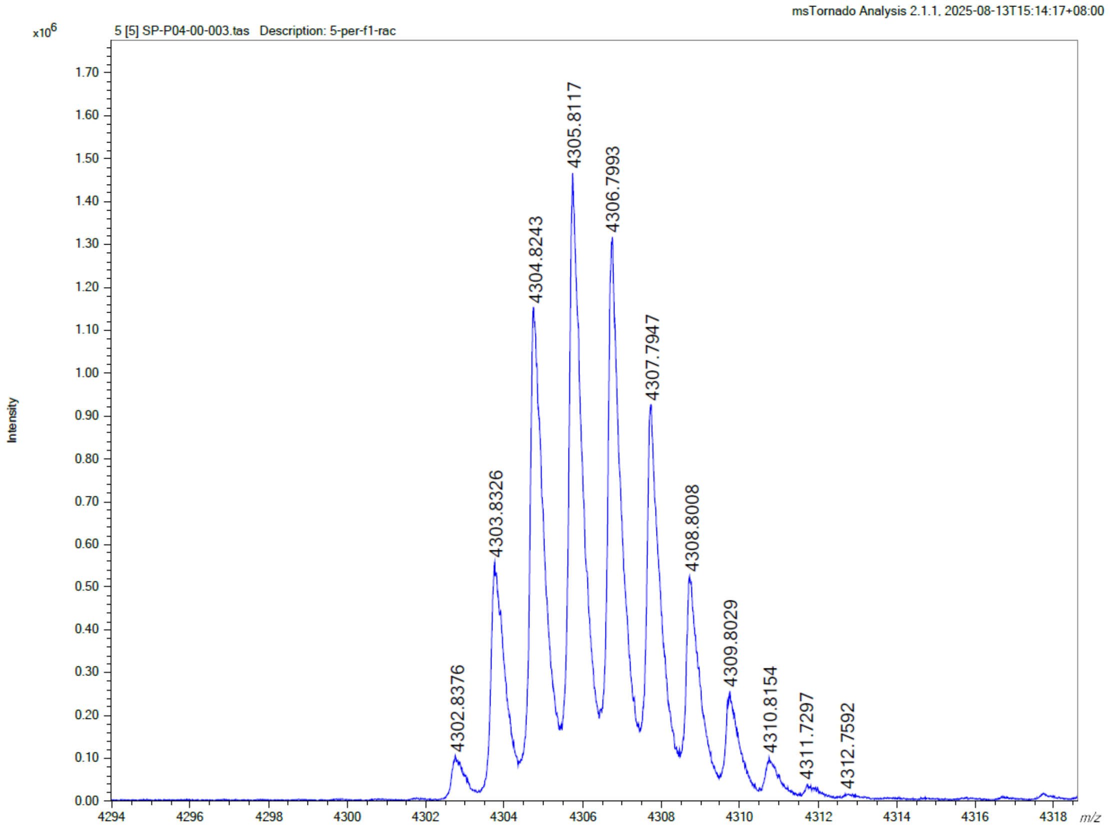

1/1

Fig. S82. HR mass spectrum (MALDI) of compound **1a**-***rac***.

Mass Spectrum Report

**Sample Name**	Compound **1a-*meso***	**Instrument Name**	JEOL JMS-S3000 Sprial-TOF
**Meas. *m*/*z***	**Formula**	**Calc. Mass**	**Err [ppm]**
4302.8255	$C_{328}H_{364}$	4302.8478	-5.18

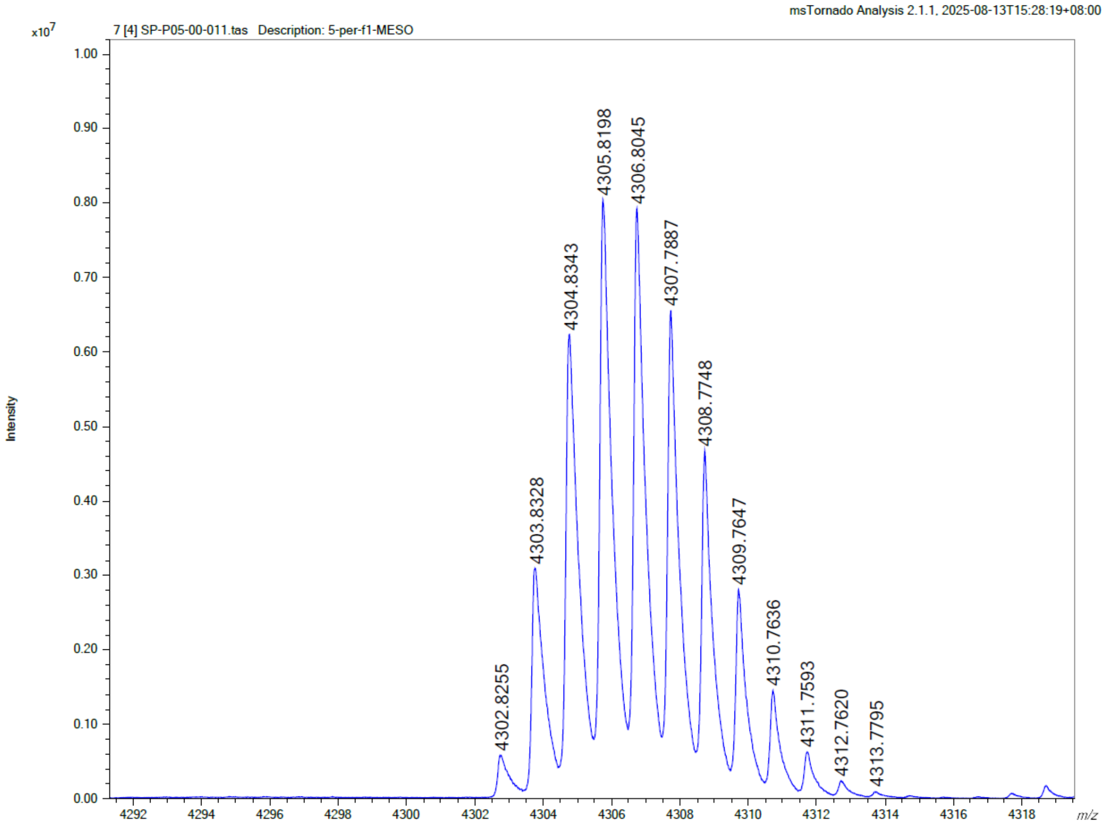

1/1

Fig. S83. HR mass spectrum (MALDI) of compound **1a-*meso***.

Mass Spectrum Report

Sample Name	Compound **1b**	Instrument Name	JEOL JMS-S3000 Sprial-TOF

**Meas. *m*/*z***	**Formula**	**Calc. Mass**	**Err [ppm]**
4302.8296	$C_{328}H_{364}$	4302.8478	-4.23

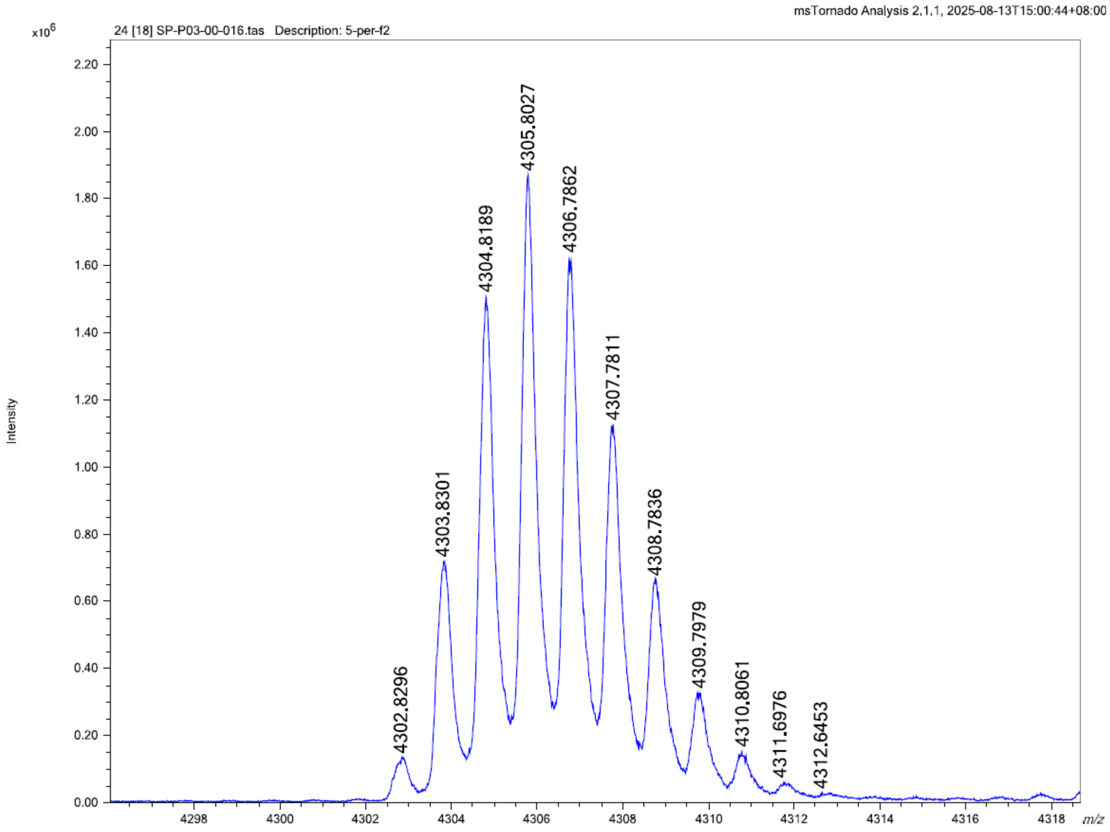

1/1

Fig. S84. HR mass spectrum (MALDI) of compound **1b**.

Mass Spectrum Report

Sample Name	Compound **1c**	Instrument Name	JEOL JMS-S3000 Sprial-TOF
**Meas. *m*/*z***	**Formula**	**Calc. Mass**	**Err [ppm]**
4298.8234	$C_{328}H_{360}$	4298.8165	1.61

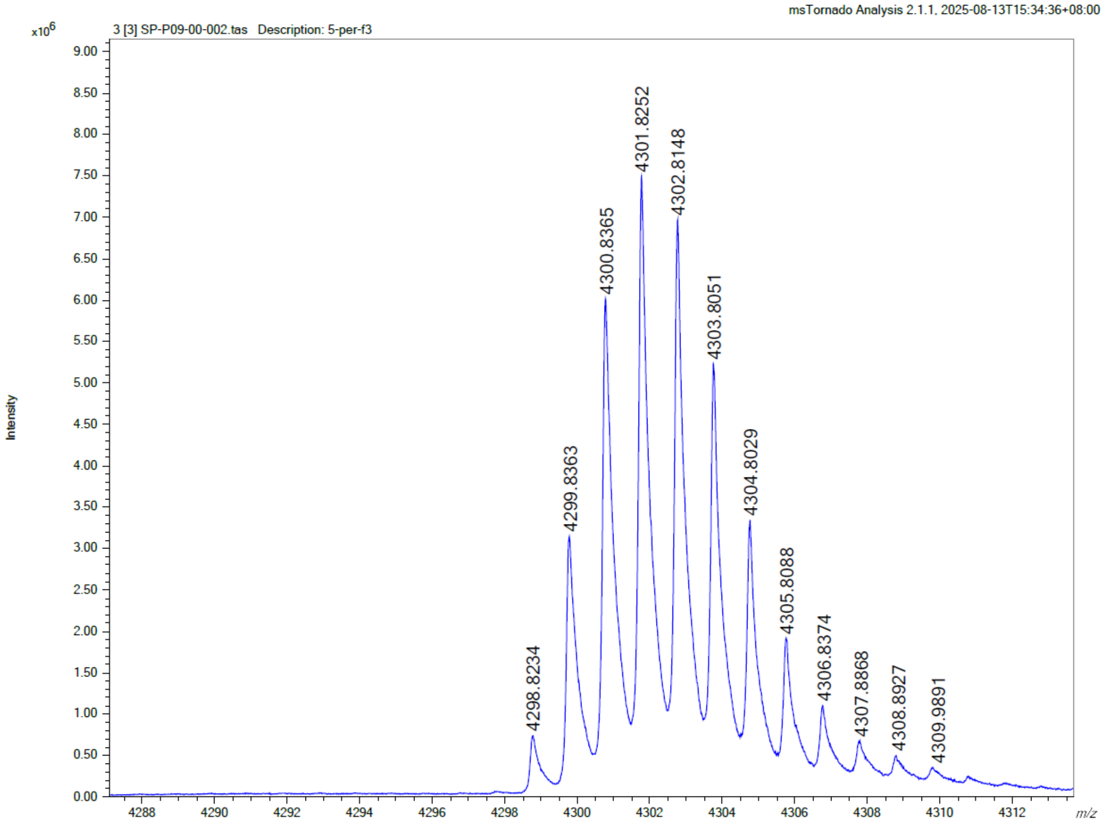

1/1

Fig. S85. HR mass spectrum (MALDI) of compound **1c**.

Mass Spectrum Report

**Sample Name**	Compound **1d**	**Instrument Name**	JEOL JMS-S3000 Sprial-TOF
**Meas. *m*/*z***	**Formula**	**Calc. Mass**	**Err [ppm]**
4296.8653	$C_{328}H_{358}$	4296.8008	15.01

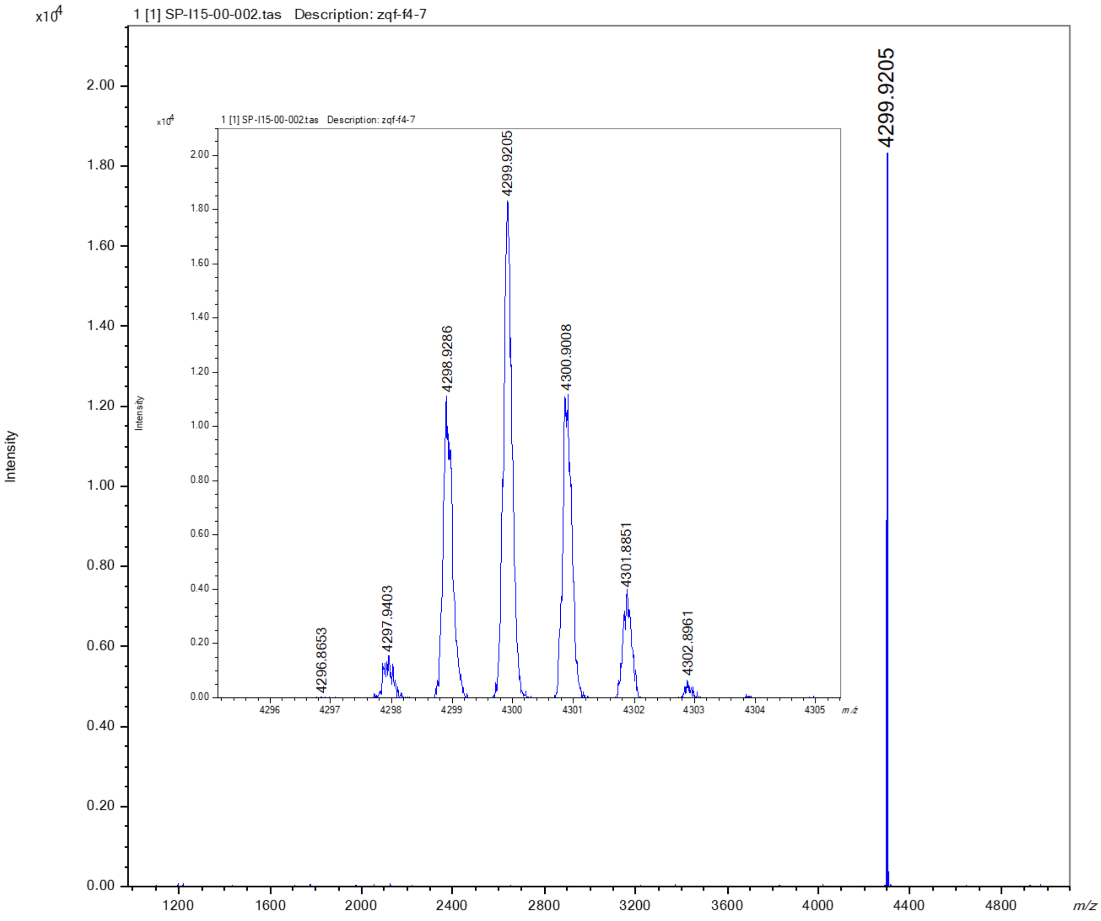

1/1

Fig. S86. HR mass spectrum (MALDI) of compound **1d**.

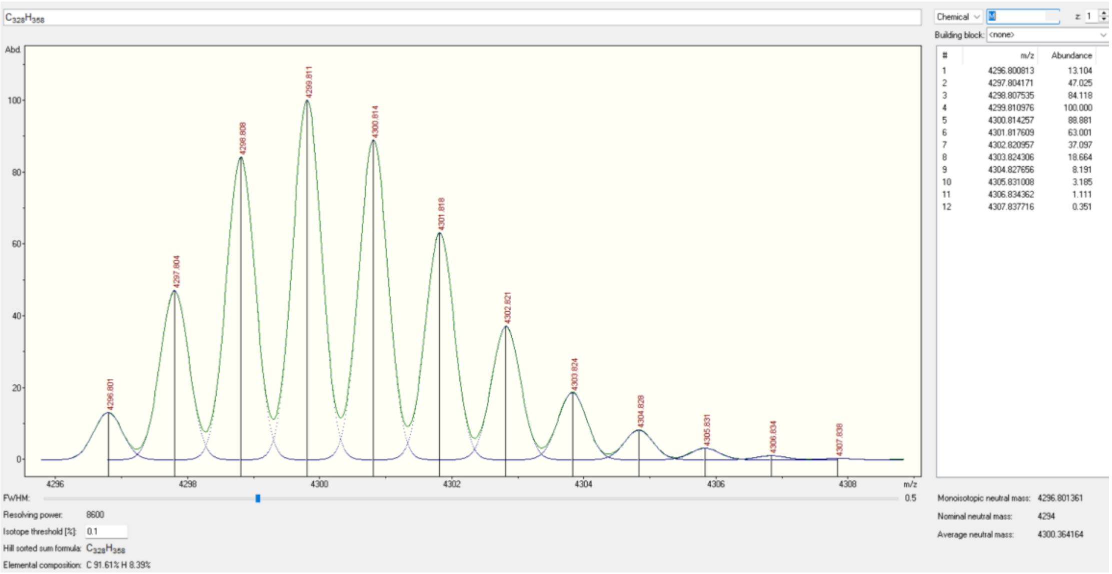

Fig. S87. Simulated mass spectrum of compound **1d**.

Multiple Mass Spectral Measurements of Compound **1d**:

1st:

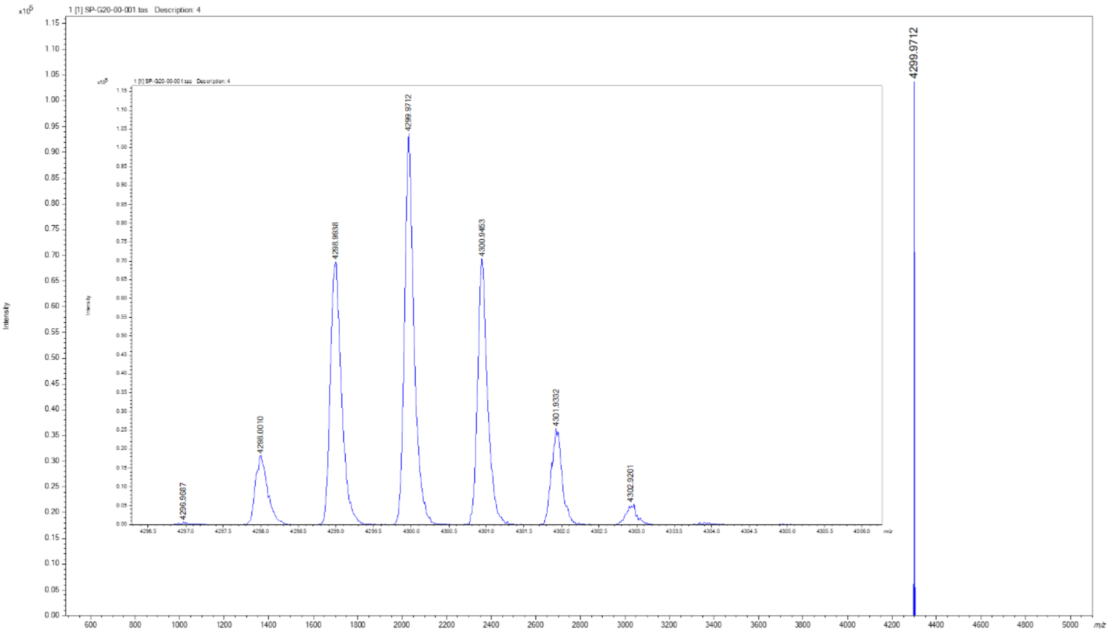

Fig. S88. Further measurements of the mass spectrum of compound **1d** (first measurement)

2nd:

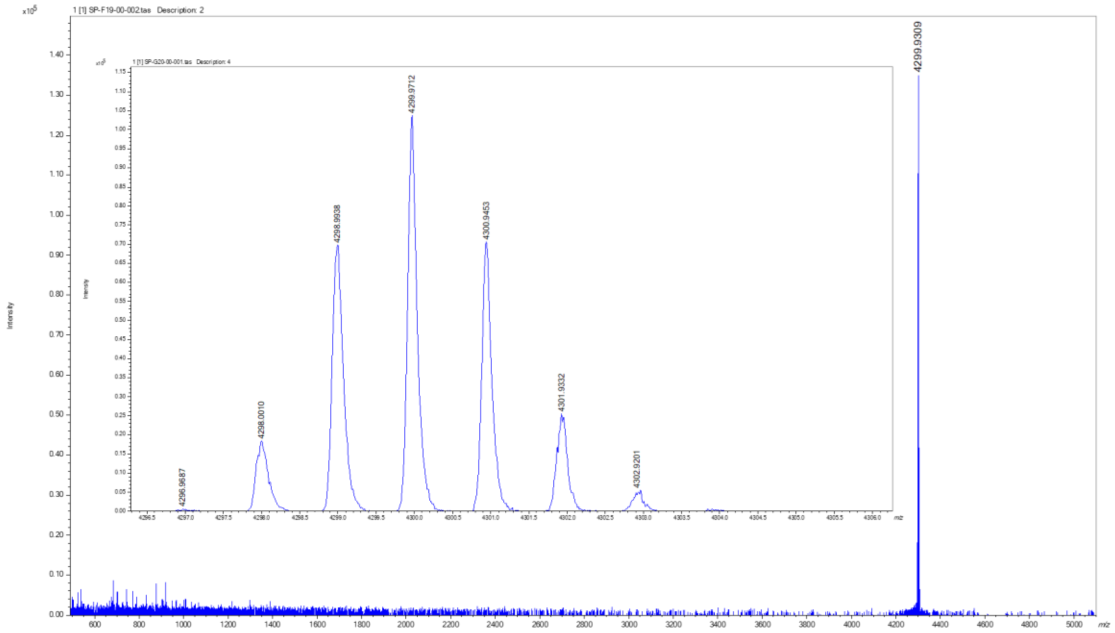

Fig. S89. Further measurements of the mass spectrum of compound **1d** (second measurement)

3rd:

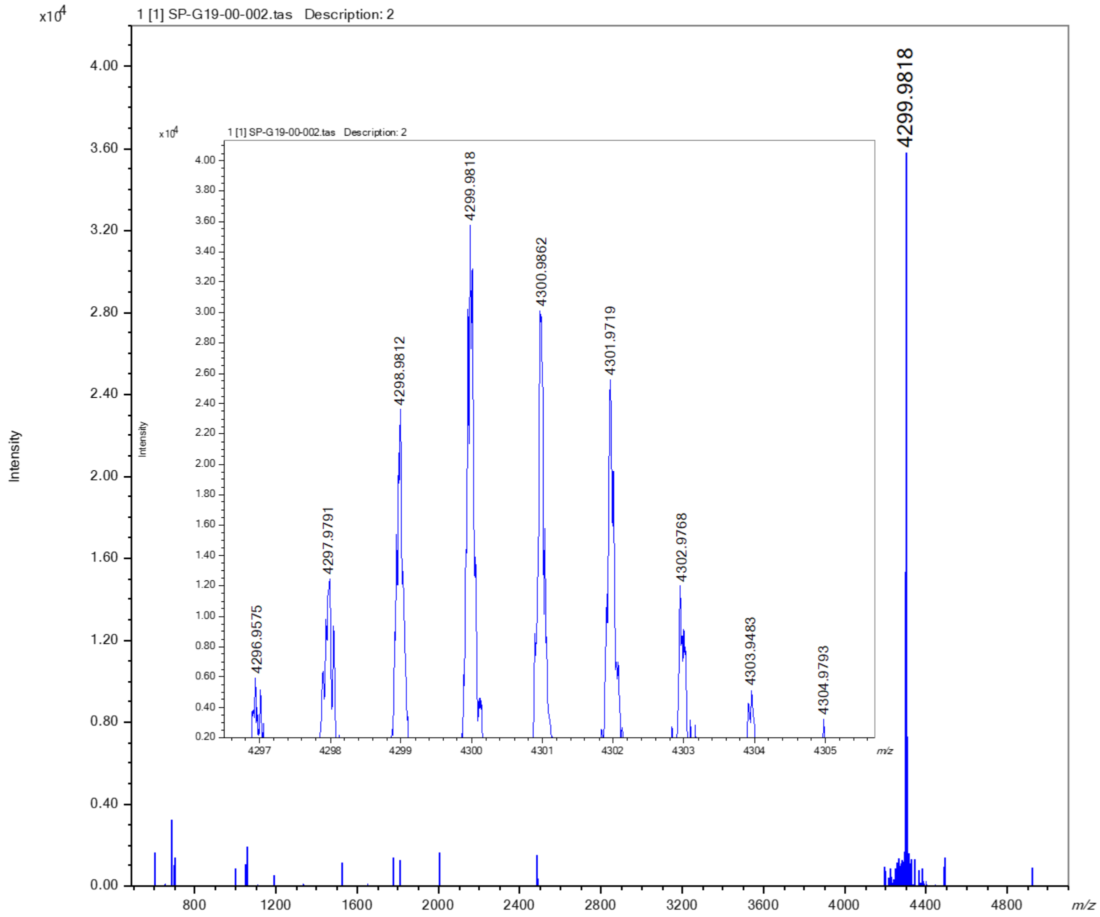

Fig. S90. Further measurements of the mass spectrum of compound **1d** (third measurement)

4th:

Mass Spectrum Report

**Sample Name**	Compound **1d**	**Instrument Name**	JEOL JMS-S3000 Sprial-TOF
**Meas. *m*/*z***	**Formula**	**Calc. Mass**	**Err [ppm]**
4296.8747	$C_{328}H_{358}$	4296.8008	17.20

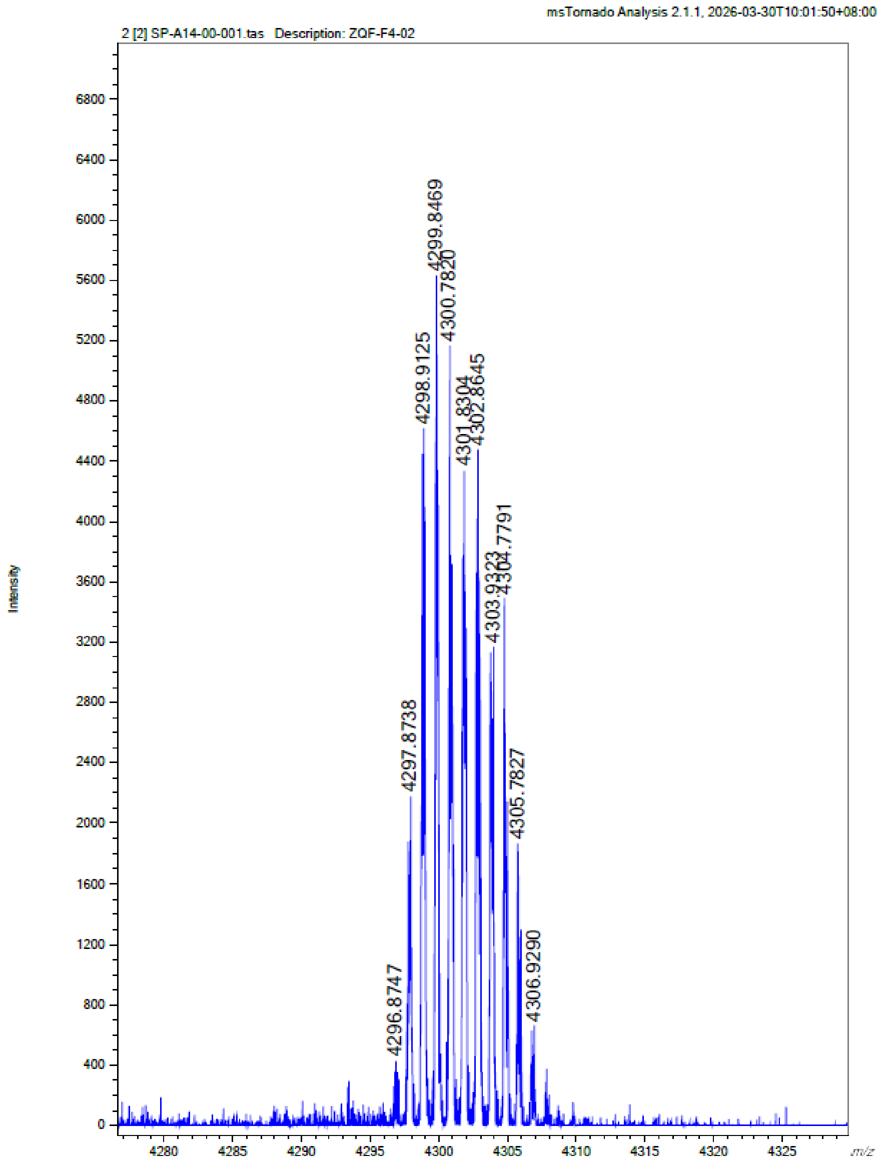

1/1

Fig. S91. Further measurements of the mass spectrum of compound **1d** (fouth measurement)

8. Appendix II: Cartesian coordinates of optimized structures

Calculated at the B3LYP-D3/6-31G(d,p) level. Coordinates (X, Y, Z) are in Angstroms.

1a-*rac*:

(*M*,*M*)-**1a-*rac*** in the ground state				(*M*,*M*)-**1a-*rac*** in the first excited state			
C	2.78	-2.451	0.012	C	-2.759	-2.457	0.063
C	1.373	-2.408	0.164	C	-1.372	-2.427	-0.09
C	0.647	-1.238	0.026	C	-0.63	-1.235	0.005
C	1.36	0.005	0.021	C	-1.341	0.006	-0.017
C	2.78	0.007	0.018	C	-2.765	0.008	-0.015
C	3.469	-1.232	-0.27	C	-3.452	-1.217	0.315
C	0.643	1.245	0.019	C	-0.626	1.246	-0.04
C	1.364	2.417	-0.122	C	-1.365	2.44	0.056
C	2.772	2.464	0.023	C	-2.752	2.474	-0.094
C	3.466	1.247	0.302	C	-3.449	1.235	-0.343
C	-0.819	-1.233	-0.071	C	0.814	-1.231	0.062
C	-1.546	-0.001	0.028	C	1.54	0.002	-0.02
C	-0.823	1.234	0.121	C	0.818	1.238	-0.099
C	-1.53	-2.392	-0.298	C	1.541	-2.414	0.252
C	-2.933	-2.461	-0.154	C	2.926	-2.47	0.107
C	-3.652	-1.278	0.21	C	3.649	-1.267	-0.236
C	-2.973	-0.004	0.033	C	2.972	0	-0.024
C	-3.659	1.267	-0.14	C	3.655	1.263	0.186
C	-2.943	2.455	0.209	C	2.934	2.471	-0.144
C	-1.539	2.391	0.347	C	1.549	2.418	-0.288
C	2.971	-4.944	0.564	C	-2.989	-4.955	-0.46
C	3.522	-3.696	0.114	C	-3.523	-3.702	-0.024
C	4.89	-3.683	-0.202	C	-4.887	-3.671	0.307
C	5.653	-4.879	-0.03	C	-5.659	-4.864	0.163
C	5.13	-6.098	0.467	C	-5.15	-6.097	-0.316
C	3.722	-6.085	0.739	C	-3.75	-6.099	-0.603

C	6.997	-4.842	-0.384	C	-7.001	-4.811	0.528
C	7.9	-5.88	-0.279	C	-7.912	-5.847	0.451
C	7.398	-7.089	0.222	C	-7.424	-7.069	-0.029
C	6.04	-7.206	0.598	C	-6.072	-7.204	-0.417
C	5.512	-2.494	-0.739	C	-5.498	-2.465	0.823
C	6.883	-2.518	-1.145	C	-6.866	-2.469	1.229
C	7.596	-3.691	-0.934	C	-7.588	-3.644	1.051
C	4.798	-1.29	-0.844	C	-4.773	-1.259	0.893
C	5.456	-0.204	-1.522	C	-5.431	-0.15	1.535
C	6.768	-0.246	-1.923	C	-6.743	-0.174	1.935
C	7.569	-1.419	-1.717	C	-7.55	-1.35	1.766
C	8.964	-1.591	-2.013	C	-8.945	-1.504	2.063
C	9.63	-2.81	-1.75	C	-9.62	-2.73	1.837
C	8.932	-3.9	-1.215	C	-8.928	-3.837	1.338
C	9.282	-5.37	-0.81	C	-9.285	-5.318	0.982
C	9.683	-6.26	-1.996	C	-9.664	-6.17	2.204
C	10.375	-5.318	0.269	C	-10.397	-5.306	-0.077
C	10.206	-7.537	-1.739	C	-10.197	-7.452	1.999
C	10.513	-8.416	-2.776	C	-10.483	-8.297	3.07
C	10.29	-8	-4.097	C	-10.227	-7.841	4.372
C	9.772	-6.737	-4.382	C	-9.699	-6.571	4.606
C	9.472	-5.873	-3.318	C	-9.421	-5.742	3.509
C	11.718	-5.186	-0.115	C	-11.731	-5.143	0.328
C	12.735	-5.078	0.835	C	-12.767	-5.078	-0.603
C	12.39	-5.102	2.193	C	-12.453	-5.178	-1.967
C	11.062	-5.225	2.603	C	-11.134	-5.332	-2.398
C	10.061	-5.328	1.628	C	-10.113	-5.39	-1.44
C	5.58	-8.516	1.129	C	-5.628	-8.524	-0.932
C	9.758	-0.461	-2.566	C	-9.738	-0.356	2.574
C	9.392	0.186	-3.753	C	-9.368	0.343	3.731

C	10.138	1.26	-4.251	C	-10.116	1.435	4.186
C	11.258	1.691	-3.532	C	-11.242	1.83	3.456
C	11.653	1.059	-2.346	C	-11.641	1.146	2.302
C	10.905	-0.027	-1.887	C	-10.891	0.045	1.885
C	4.898	-8.606	2.354	C	-4.948	-8.639	-2.155
C	4.492	-9.84	2.869	C	-4.558	-9.883	-2.655
C	4.776	-10.999	2.137	C	-4.856	-11.03	-1.909
C	5.457	-10.945	0.916	C	-5.535	-10.952	-0.689
C	5.855	-9.697	0.426	C	-5.918	-9.694	-0.214
C	5.464	0.225	1.541	C	-5.437	0.172	-1.552
C	4.799	1.31	0.868	C	-4.773	1.28	-0.914
C	5.509	2.514	0.756	C	-5.495	2.487	-0.839
C	6.884	2.54	1.149	C	-6.867	2.492	-1.234
C	7.577	1.442	1.713	C	-7.557	1.373	-1.762
C	6.779	0.269	1.93	C	-6.752	0.197	-1.939
C	7.594	3.713	0.929	C	-7.587	3.667	-1.048
C	8.933	3.922	1.192	C	-8.931	3.859	-1.317
C	9.639	2.832	1.717	C	-9.628	2.752	-1.806
C	8.976	1.613	1.99	C	-8.956	1.526	-2.039
C	4.881	3.702	0.222	C	-4.879	3.692	-0.326
C	5.642	4.897	0.039	C	-5.65	4.885	-0.175
C	6.989	4.862	0.382	C	-6.994	4.833	-0.529
C	3.511	3.711	-0.086	C	-3.514	3.72	-0.003
C	2.955	4.957	-0.535	C	-2.976	4.971	0.432
C	3.704	6.098	-0.72	C	-3.735	6.116	0.583
C	5.114	6.114	-0.459	C	-5.137	6.116	0.305
C	6.023	7.22	-0.605	C	-6.059	7.222	0.419
C	7.385	7.105	-0.241	C	-7.414	7.088	0.044
C	7.891	5.9	0.261	C	-7.906	5.868	-0.437
C	9.279	5.39	0.778	C	-9.286	5.338	-0.952

C	10.359	5.335	-0.314	C	-10.382	5.321	0.124
C	9.695	6.285	1.956	C	-9.685	6.193	-2.165
C	11.706	5.204	0.055	C	-11.721	5.163	-0.263
C	12.712	5.093	-0.906	C	-12.744	5.094	0.684
C	12.352	5.113	-2.261	C	-12.409	5.185	2.042
C	11.019	5.234	-2.655	C	-11.085	5.334	2.455
C	10.029	5.34	-1.669	C	-10.077	5.396	1.482
C	9.505	5.9	3.282	C	-9.471	5.767	-3.475
C	9.821	6.767	4.339	C	-9.77	6.598	-4.565
C	10.331	8.031	4.043	C	-10.288	7.87	-4.318
C	10.533	8.444	2.718	C	-10.514	8.325	-3.011
C	10.212	7.562	1.688	C	-10.208	7.478	-1.947
C	9.778	0.481	2.526	C	-9.755	0.374	-2.533
C	5.558	8.527	-1.141	C	-5.611	8.54	0.938
C	4.867	8.61	-2.36	C	-4.922	8.648	2.157
C	4.457	9.84	-2.88	C	-4.53	9.891	2.66
C	4.748	11.004	-2.158	C	-4.835	11.041	1.922
C	5.439	10.958	-0.943	C	-5.524	10.969	0.707
C	5.84	9.713	-0.448	C	-5.909	9.713	0.228
C	9.433	-0.169	3.718	C	-9.405	-0.329	-3.693
C	10.186	-1.246	4.198	C	-10.157	-1.426	-4.129
C	11.29	-1.678	3.456	C	-11.269	-1.82	-3.377
C	11.663	-1.044	2.265	C	-11.649	-1.132	-2.219
C	10.909	0.046	1.823	C	-10.894	-0.027	-1.821
C	-3.019	-4.914	-0.866	C	3.051	-4.926	0.809
C	-3.619	-3.729	-0.316	C	3.631	-3.74	0.257
C	-4.97	-3.806	0.043	C	4.974	-3.805	-0.131
C	-5.656	-5.05	-0.116	C	5.665	-5.05	-0.004
C	-5.065	-6.235	-0.621	C	5.087	-6.244	0.495
C	-3.696	-6.102	-1.025	C	3.733	-6.119	0.936

C	-6.991	-5.1	0.262	C	6.994	-5.09	-0.404
C	-7.818	-6.203	0.196	C	7.827	-6.191	-0.366
C	-7.251	-7.377	-0.312	C	7.272	-7.377	0.13
C	-5.893	-7.408	-0.705	C	5.922	-7.42	0.544
C	-5.644	-2.661	0.615	C	5.638	-2.646	-0.691
C	-7.011	-2.771	1.023	C	6.999	-2.744	-1.11
C	-7.658	-3.982	0.799	C	7.652	-3.959	-0.923
C	-4.986	-1.427	0.768	C	4.973	-1.405	-0.804
C	-5.699	-0.409	1.497	C	5.688	-0.366	-1.505
C	-7.01	-0.53	1.89	C	6.996	-0.476	-1.907
C	-7.754	-1.728	1.626	C	7.742	-1.682	-1.685
C	-9.138	-1.982	1.912	C	9.123	-1.925	-1.981
C	-9.734	-3.234	1.637	C	9.725	-3.186	-1.744
C	-8.979	-4.273	1.08	C	8.974	-4.24	-1.217
C	-9.219	-5.784	0.748	C	9.22	-5.758	-0.925
C	-9.589	-6.484	2.066	C	9.571	-6.424	-2.266
C	-10.255	-6.047	-0.352	C	10.269	-6.049	0.155
C	-8.642	-7.158	2.837	C	8.611	-7.073	-3.042
C	-8.987	-7.732	4.069	C	8.937	-7.612	-4.295
C	-10.304	-7.618	4.519	C	10.246	-7.489	-4.76
C	-11.275	-6.94	3.768	C	11.231	-6.836	-4.004
C	-10.904	-6.382	2.545	C	10.879	-6.311	-2.761
C	-10.726	-7.355	-0.543	C	10.75	-7.359	0.299
C	-11.59	-7.667	-1.592	C	11.627	-7.699	1.33
C	-11.985	-6.642	-2.462	C	12.022	-6.7	2.229
C	-11.536	-5.332	-2.293	C	11.561	-5.388	2.107
C	-10.666	-5.045	-1.23	C	10.681	-5.074	1.062
C	-5.346	-8.698	-1.202	C	5.386	-8.72	1.025
C	-10.006	-0.937	2.518	C	9.991	-0.863	-2.556
C	-9.718	-0.399	3.781	C	9.698	-0.279	-3.796

C	-10.583	0.513	4.391	C	10.566	0.648	-4.38
C	-11.746	0.892	3.709	C	11.736	0.994	-3.693
C	-12.041	0.403	2.433	C	12.037	0.457	-2.437
C	-11.165	-0.52	1.851	C	11.159	-0.479	-1.883
C	-6.019	-9.41	-2.206	C	6.09	-9.462	1.984
C	-5.541	-10.638	-2.672	C	5.622	-10.701	2.432
C	-4.369	-11.157	-2.112	C	4.43	-11.201	1.897
C	-3.676	-10.477	-1.103	C	3.707	-10.49	0.931
C	-4.175	-9.25	-0.658	C	4.195	-9.252	0.505
C	-3.022	4.932	0.844	C	3.048	4.952	-0.76
C	-3.634	3.723	0.366	C	3.644	3.74	-0.287
C	-4.996	3.783	0.042	C	5	3.788	0.061
C	-5.69	5.022	0.209	C	5.7	5.026	-0.077
C	-5.101	6.213	0.7	C	5.125	6.227	-0.562
C	-3.705	6.114	1.009	C	3.738	6.14	-0.895
C	-7.02	5.071	-0.187	C	7.026	5.064	0.336
C	-7.839	6.181	-0.16	C	7.851	6.171	0.336
C	-7.28	7.357	0.352	C	7.306	7.358	-0.167
C	-5.935	7.383	0.786	C	5.969	7.397	-0.62
C	-5.666	2.634	-0.528	C	5.66	2.625	0.618
C	-7.032	2.74	-0.944	C	7.021	2.717	1.042
C	-7.682	3.951	-0.729	C	7.677	3.932	0.861
C	-4.998	1.406	-0.689	C	4.985	1.391	0.744
C	-5.706	0.385	-1.419	C	5.693	0.35	1.447
C	-7.014	0.503	-1.821	C	7	0.456	1.856
C	-7.764	1.699	-1.567	C	7.752	1.658	1.639
C	-9.139	1.96	-1.89	C	9.126	1.905	1.967
C	-9.735	3.216	-1.63	C	9.729	3.167	1.742
C	-8.992	4.249	-1.049	C	8.99	4.217	1.191
C	-9.229	5.766	-0.742	C	9.234	5.74	0.921

C	-10.295	6.052	0.321	C	10.309	6.049	-0.127
C	-9.551	6.45	-2.082	C	9.543	6.391	2.279
C	-10.774	7.362	0.47	C	10.801	7.358	-0.232
C	-11.667	7.696	1.488	C	11.703	7.714	-1.236
C	-12.081	6.69	2.371	C	12.11	6.733	-2.149
C	-11.623	5.378	2.245	C	11.637	5.422	-2.066
C	-10.726	5.07	1.212	C	10.734	5.091	-1.047
C	-8.57	7.095	-2.836	C	8.557	7.025	3.036
C	-8.869	7.652	-4.088	C	8.844	7.554	4.302
C	-10.173	7.552	-4.574	C	10.143	7.437	4.801
C	-11.177	6.903	-3.841	C	11.152	6.8	4.066
C	-10.852	6.36	-2.598	C	10.838	6.285	2.808
C	-5.404	8.663	1.324	C	5.454	8.685	-1.153
C	-9.996	0.926	-2.529	C	9.982	0.852	2.574
C	-9.649	0.357	-3.765	C	9.638	0.249	3.795
C	-10.501	-0.541	-4.411	C	10.491	-0.668	4.412
C	-11.718	-0.87	-3.797	C	11.706	-0.98	3.786
C	-12.078	-0.345	-2.554	C	12.062	-0.422	2.556
C	-11.208	0.559	-1.932	C	11.191	0.499	1.962
C	-5.571	9.855	0.603	C	5.629	9.871	-0.424
C	-5.104	11.076	1.098	C	5.178	11.099	-0.915
C	-4.465	11.094	2.343	C	4.547	11.132	-2.163
C	-4.288	9.925	3.092	C	4.364	9.969	-2.921
C	-4.761	8.717	2.571	C	4.82	8.753	-2.404
C	5.721	-12.202	0.121	C	-5.814	-12.196	0.123
C	3.797	-9.928	4.207	C	-3.865	-9.998	-3.993
C	9.724	1.936	-5.538	C	-9.695	2.171	5.437
C	12.826	1.569	-1.545	C	-12.824	1.614	1.488
C	12.82	-1.555	1.439	C	-12.815	-1.6	-1.382
C	9.795	-1.926	5.49	C	-9.755	-2.169	-5.382

C	3.751	9.92	-4.213	C	-3.826	9.999	3.993
C	5.71	12.22	-0.158	C	-5.811	12.217	-0.095
C	-2.44	-11.081	-0.479	C	2.448	-11.073	0.333
C	-6.254	-11.366	-3.788	C	6.369	-11.463	3.502
C	-10.299	1.046	5.776	C	10.275	1.233	-5.742
C	-13.259	0.887	1.681	C	13.263	0.906	-1.678
C	-3.639	9.974	4.455	C	3.724	10.034	-4.288
C	-5.248	12.342	0.287	C	5.33	12.359	-0.095
C	-10.143	-1.137	-5.751	C	10.125	-1.319	5.725
C	-13.361	-0.764	-1.876	C	13.343	-0.823	1.862
C	-7.939	-8.44	4.894	C	7.873	-8.297	-5.121
C	-12.687	-6.804	4.286	C	12.636	-6.69	-4.539
C	-12.112	-9.073	-1.774	C	12.16	-9.107	1.461
C	-11.979	-4.227	-3.219	C	12.006	-4.312	3.065
C	-12.087	4.296	3.188	C	12.094	4.366	-3.042
C	-12.197	9.104	1.625	C	12.248	9.12	-1.324
C	-7.784	8.327	-4.894	C	7.753	8.215	5.111
C	-12.576	6.78	-4.399	C	12.543	6.659	4.637
C	9.594	6.325	5.766	C	-9.509	6.116	-5.973
C	11.108	9.81	2.423	C	-11.103	9.695	-2.768
C	14.157	4.927	-0.498	C	-14.181	4.9	0.26
C	10.631	5.276	-4.114	C	-10.724	5.454	3.916
C	11.099	-9.78	-2.492	C	-11.082	-9.665	2.842
C	9.517	-6.295	-5.804	C	-9.406	-6.088	6.007
C	14.174	-4.906	0.41	C	-14.197	-4.878	-0.16
C	10.691	-5.271	4.066	C	-10.796	-5.461	-3.863
H	0.846	-3.337	0.331	H	-0.845	-3.356	-0.266
H	0.833	3.345	-0.288	H	-0.836	3.367	0.231
H	-0.991	-3.302	-0.52	H	1.004	-3.323	0.481
H	-1	3.302	0.571	H	1.012	3.326	-0.521

H	1.914	-4.997	0.804	H	-1.936	-5.017	-0.714
H	3.234	-6.99	1.083	H	-3.269	-7.012	-0.935
H	8.041	-7.955	0.346	H	-8.075	-7.932	-0.131
H	4.892	0.701	-1.711	H	-4.864	0.758	1.693
H	7.202	0.627	-2.394	H	-7.174	0.717	2.374
H	10.687	-2.881	-1.991	H	-10.676	-2.787	2.081
H	10.387	-7.839	-0.711	H	-10.402	-7.785	0.986
H	10.527	-8.677	-4.915	H	-10.446	-8.492	5.216
H	9.066	-4.889	-3.532	H	-9.007	-4.754	3.682
H	11.971	-5.187	-1.171	H	-11.96	-5.088	1.388
H	13.176	-5.029	2.943	H	-13.254	-5.14	-2.702
H	9.026	-5.424	1.939	H	-9.085	-5.51	-1.768
H	8.523	-0.166	-4.303	H	-8.496	0.017	4.292
H	11.833	2.538	-3.896	H	-11.82	2.688	3.789
H	11.193	-0.537	-0.974	H	-11.182	-0.503	0.995
H	4.705	-7.699	2.919	H	-4.744	-7.741	-2.733
H	4.461	-11.965	2.528	H	-4.553	-12.004	-2.288
H	6.372	-9.634	-0.528	H	-6.433	-9.613	0.74
H	4.902	-0.68	1.737	H	-4.871	-0.737	-1.715
H	7.219	-0.604	2.398	H	-7.188	-0.695	-2.375
H	10.699	2.903	1.943	H	-10.689	2.807	-2.033
H	1.896	5.008	-0.767	H	-1.921	5.033	0.68
H	3.212	7.001	-1.064	H	-3.252	7.028	0.914
H	8.026	7.97	-0.378	H	-8.065	7.949	0.157
H	11.972	5.209	1.108	H	-11.967	5.114	-1.32
H	13.129	5.039	-3.019	H	-13.2	5.144	2.789
H	8.991	5.435	-1.968	H	-9.044	5.512	1.796
H	9.105	4.916	3.504	H	-9.065	4.777	-3.659
H	10.579	8.711	4.856	H	-10.524	8.522	-5.157
H	10.377	7.862	0.656	H	-10.392	7.81	-0.929

H	4.668	7.699	-2.918	H	-4.712	7.748	2.728
H	4.431	11.967	-2.553	H	-4.531	12.014	2.304
H	6.365	9.655	0.502	H	-6.431	9.636	-0.722
H	8.576	0.184	4.286	H	-8.544	-0.003	-4.271
H	11.87	-2.527	3.807	H	-11.85	-2.682	-3.695
H	11.18	0.556	0.905	H	-11.17	0.524	-0.927
H	-1.993	-4.873	-1.215	H	2.034	-4.889	1.186
H	-3.186	-6.943	-1.48	H	3.235	-6.966	1.395
H	-7.829	-8.292	-0.386	H	7.857	-8.29	0.178
H	-5.178	0.509	1.738	H	5.167	0.56	-1.711
H	-7.485	0.296	2.407	H	7.469	0.367	-2.398
H	-10.778	-3.373	1.902	H	10.768	-3.314	-2.015
H	-7.622	-7.248	2.478	H	7.595	-7.169	-2.672
H	-10.584	-8.068	5.469	H	10.512	-7.912	-5.727
H	-11.651	-5.874	1.941	H	11.635	-5.821	-2.154
H	-10.415	-8.136	0.144	H	10.439	-8.12	-0.411
H	-12.655	-6.874	-3.288	H	12.7	-6.954	3.042
H	-10.302	-4.031	-1.098	H	10.308	-4.059	0.966
H	-8.823	-0.726	4.303	H	8.797	-0.581	-4.323
H	-12.434	1.587	4.182	H	12.426	1.701	-4.147
H	-11.372	-0.915	0.859	H	11.37	-0.91	-0.908
H	-6.92	-8.983	-2.639	H	7.006	-9.051	2.4
H	-3.987	-12.112	-2.467	H	4.057	-12.164	2.237
H	-3.662	-8.721	0.14	H	3.658	-8.701	-0.262
H	-1.97	4.924	1.106	H	2.001	4.951	-1.042
H	-3.171	6.987	1.366	H	3.212	7.018	-1.248
H	-7.87	8.263	0.447	H	7.904	8.26	-0.244
H	-5.181	-0.531	-1.657	H	5.168	-0.573	1.654
H	-7.481	-0.326	-2.34	H	7.464	-0.389	2.352
H	-10.768	3.364	-1.93	H	10.763	3.302	2.045

H	-10.446	8.129	-0.226	H	10.478	8.106	0.486
H	-12.773	6.939	3.174	H	12.807	7	-2.941
H	-10.355	4.054	1.113	H	10.351	4.076	-0.983
H	-7.559	7.173	-2.45	H	7.551	7.115	2.64
H	-10.419	7.99	-5.54	H	10.379	7.853	5.778
H	-11.624	5.874	-2.009	H	11.615	5.807	2.217
H	-8.717	0.651	-4.239	H	8.708	0.531	4.28
H	-12.396	-1.553	-4.302	H	12.387	-1.675	4.271
H	-11.467	0.981	-0.964	H	11.448	0.949	1.006
H	-6.056	9.819	-0.369	H	6.107	9.824	0.551
H	-4.097	12.039	2.738	H	4.192	12.083	-2.555
H	-4.652	7.804	3.149	H	4.705	7.846	-2.99
H	4.901	-12.404	-0.58	H	-4.997	-12.399	0.826
H	6.638	-12.116	-0.47	H	-6.73	-12.092	0.711
H	5.816	-13.076	0.772	H	-5.919	-13.078	-0.518
H	4.509	-10.171	5.006	H	-4.577	-10.263	-4.784
H	3.031	-10.71	4.209	H	-3.094	-10.775	-3.978
H	3.317	-8.982	4.474	H	-3.391	-9.055	-4.282
H	9.517	1.201	-6.323	H	-9.43	1.474	6.239
H	8.808	2.523	-5.4	H	-8.811	2.795	5.251
H	10.5	2.614	-5.904	H	-10.489	2.826	5.805
H	13.531	2.125	-2.17	H	-13.527	2.192	2.094
H	12.484	2.254	-0.761	H	-12.492	2.266	0.673
H	13.367	0.749	-1.062	H	-13.363	0.77	1.046
H	13.542	-2.102	2.052	H	-13.533	-2.173	-1.976
H	13.344	-0.736	0.937	H	-13.342	-0.756	-0.925
H	12.462	-2.248	0.669	H	-12.468	-2.257	-0.577
H	10.577	-2.603	5.842	H	-10.559	-2.817	-5.741
H	8.878	-2.515	5.366	H	-8.876	-2.801	-5.202
H	9.599	-1.193	6.28	H	-9.49	-1.476	-6.188

H	4.456	10.156	-5.019	H	-4.532	10.262	4.791
H	2.985	10.703	-4.214	H	-3.055	10.776	3.975
H	3.267	8.973	-4.469	H	-3.351	9.055	4.274
H	4.893	12.431	0.543	H	-4.996	12.427	-0.8
H	5.807	13.089	-0.816	H	-5.917	13.094	0.551
H	6.628	12.135	0.431	H	-6.729	12.113	-0.682
H	-1.813	-10.318	-0.009	H	1.82	-10.298	-0.116
H	-1.832	-11.606	-1.224	H	1.852	-11.596	1.088
H	-2.705	-11.811	0.296	H	2.685	-11.8	-0.454
H	-7.332	-11.18	-3.762	H	7.445	-11.275	3.45
H	-6.093	-12.447	-3.728	H	6.207	-12.542	3.413
H	-5.89	-11.035	-4.769	H	6.034	-11.164	4.503
H	-9.23	1.013	6.006	H	9.205	1.209	-5.968
H	-10.817	0.454	6.541	H	10.789	0.67	-6.531
H	-10.637	2.082	5.882	H	10.613	2.272	-5.811
H	-14.033	1.253	2.362	H	14.035	1.293	-2.35
H	-12.999	1.714	1.008	H	13.014	1.707	-0.971
H	-13.693	0.094	1.067	H	13.697	0.086	-1.097
H	-4.376	10.204	5.234	H	4.466	10.277	-5.059
H	-3.178	9.016	4.714	H	3.269	9.078	-4.564
H	-2.866	10.747	4.502	H	2.948	10.805	-4.33
H	-5.369	13.219	0.931	H	5.455	13.24	-0.732
H	-6.111	12.292	-0.384	H	6.193	12.3	0.576
H	-4.361	12.515	-0.334	H	4.444	12.532	0.529
H	-10.171	-2.232	-5.716	H	9.931	-2.39	5.592
H	-9.141	-0.838	-6.071	H	9.228	-0.871	6.16
H	-10.85	-0.822	-6.527	H	10.938	-1.228	6.454
H	-14.058	-1.225	-2.581	H	14.054	-1.277	2.558
H	-13.164	-1.496	-1.082	H	13.144	-1.557	1.07
H	-13.865	0.089	-1.41	H	13.83	0.037	1.391

H	-8.391	-9.101	5.64	H	8.31	-8.88	-5.937
H	-7.275	-9.042	4.264	H	7.265	-8.97	-4.509
H	-7.308	-7.72	5.43	H	7.19	-7.563	-5.568
H	-13.386	-6.549	3.484	H	13.342	-6.427	-3.747
H	-12.753	-6.014	5.045	H	12.687	-5.902	-5.301
H	-13.033	-7.731	4.755	H	12.982	-7.616	-5.01
H	-11.484	-9.804	-1.257	H	11.527	-9.826	0.933
H	-13.128	-9.172	-1.374	H	13.17	-9.187	1.04
H	-12.154	-9.35	-2.833	H	12.222	-9.414	2.51
H	-12.754	-3.608	-2.752	H	12.803	-3.699	2.627
H	-11.15	-3.557	-3.464	H	11.185	-3.632	3.307
H	-12.39	-4.622	-4.153	H	12.39	-4.734	3.999
H	-12.462	4.713	4.128	H	12.473	4.808	-3.968
H	-12.898	3.706	2.743	H	12.898	3.755	-2.614
H	-11.279	3.597	3.418	H	11.279	3.682	-3.294
H	-11.564	9.824	1.098	H	11.609	9.832	-0.794
H	-12.256	9.408	2.675	H	12.334	9.451	-2.364
H	-13.208	9.188	1.207	H	13.25	9.184	-0.881
H	-7.114	8.909	-4.253	H	7.095	8.817	4.476
H	-7.167	7.587	-5.418	H	7.125	7.466	5.609
H	-8.202	9	-5.649	H	8.165	8.866	5.888
H	-13.301	6.545	-3.615	H	13.275	6.423	3.859
H	-12.893	7.706	-4.89	H	12.865	7.579	5.137
H	-12.631	5.981	-5.149	H	12.583	5.855	5.382
H	10.102	5.375	5.972	H	-9.924	5.115	-6.133
H	9.962	7.065	6.482	H	-9.947	6.788	-6.717
H	8.528	6.166	5.965	H	-8.433	6.053	-6.174
H	12.176	9.852	2.672	H	-12.182	9.702	-2.968
H	10.61	10.587	3.011	H	-10.649	10.446	-3.422
H	11.006	10.068	1.365	H	-10.959	10.015	-1.732

H	14.442	3.867	-0.491	H	-14.487	3.854	0.378
H	14.338	5.321	0.506	H	-14.33	5.17	-0.789
H	14.831	5.439	-1.192	H	-14.862	5.506	0.867
H	9.812	4.58	-4.322	H	-9.899	4.783	4.173
H	11.47	5.012	-4.763	H	-11.572	5.21	4.563
H	10.288	6.276	-4.402	H	-10.402	6.474	4.16
H	10.615	-10.555	-3.097	H	-10.626	-10.415	3.496
H	12.169	-9.808	-2.73	H	-12.159	-9.665	3.052
H	10.987	-10.053	-1.439	H	-10.95	-9.993	1.807
H	9.934	-7	-6.528	H	-9.823	-6.761	6.761
H	8.441	-6.211	-6.003	H	-8.326	-6.02	6.182
H	9.956	-5.31	-5.999	H	-9.822	-5.089	6.177
H	14.451	-3.845	0.384	H	-14.5	-3.83	-0.277
H	14.348	-5.314	-0.59	H	-14.331	-5.143	0.893
H	14.859	-5.403	1.104	H	-14.889	-5.483	-0.754
H	11.536	-5.004	4.706	H	-11.653	-5.221	-4.499
H	9.87	-4.581	4.284	H	-9.976	-4.79	-4.137
H	10.358	-6.275	4.357	H	-10.476	-6.481	-4.107

1a-*meso* and **perylene** in ground states:

**1a-*meso*** in the ground state				**Perylene** in the ground state			
C	-2.871	-2.413	-0.076	C	-2.886	2.422	0.001
C	-1.471	-2.36	-0.264	C	-1.479	2.427	0
C	-0.737	-1.199	-0.103	C	-0.738	1.25	0
C	-1.45	0.042	0.013	C	-1.439	0	0
C	-2.874	0.038	0.048	C	-2.874	0	0
C	-3.55	-1.217	0.3	C	-3.575	1.232	0
C	-0.74	1.287	0.079	C	-0.738	-1.25	0
C	-1.474	2.453	0.184	C	-1.479	-2.427	0
C	-2.878	2.5	0.021	C	2.886	-2.422	0.001

C	-3.579	1.278	-0.208
C	0.735	-1.199	-0.091
C	1.445	0.045	0.006
C	0.734	1.289	0.061
C	1.469	-2.364	-0.213
C	2.87	-2.412	-0.024
C	3.549	-1.204	0.311
C	2.869	0.044	0.032
C	3.571	1.278	-0.256
C	2.868	2.505	-0.054
C	1.466	2.46	0.123
C	-3.08	-4.846	-0.836
C	-3.613	-3.65	-0.247
C	-4.971	-3.663	0.105
C	-5.734	-4.849	-0.125
C	-5.212	-6.042	-0.685
C	-3.829	-5.98	-1.057
C	-7.079	-4.826	0.227
C	-7.98	-5.861	0.077
C	-7.475	-7.05	-0.465
C	-6.115	-7.152	-0.841
C	-5.581	-2.512	0.732
C	-6.947	-2.562	1.152
C	-7.671	-3.711	0.853
C	-4.863	-1.318	0.907
C	-5.507	-0.28	1.668
C	-6.805	-0.361	2.111
C	-7.611	-1.518	1.84
C	-8.99	-1.729	2.186
C	-9.669	-2.916	1.822

C	-3.575	-1.232	0
C	0.738	1.25	0
C	1.439	0	0
C	0.738	-1.25	0
C	1.479	2.427	0
C	2.886	2.422	0
C	3.575	1.232	0
C	2.874	0	0
C	3.575	-1.232	0
C	-2.886	-2.422	-0.001
C	1.479	-2.427	0
H	-3.421	3.367	0.001
H	-0.978	3.388	0.001
H	-4.661	1.218	0
H	-0.978	-3.388	-0.001
H	3.421	-3.367	0.001
H	-4.662	-1.218	0
H	0.978	3.388	-0.001
H	3.421	3.367	-0.001
H	4.661	1.218	0
H	4.662	-1.218	0
H	-3.421	-3.367	-0.001
H	0.978	-3.388	0.001

C	-8.999	-3.944	1.149
C	-9.348	-5.396	0.679
C	-9.677	-6.341	1.85
C	-10.499	-5.361	-0.338
C	-10.265	-7.586	1.578
C	-10.509	-8.509	2.595
C	-10.161	-8.168	3.91
C	-9.575	-6.937	4.209
C	-9.333	-6.032	3.166
C	-11.796	-5.089	0.119
C	-12.884	-5.064	-0.755
C	-12.66	-5.327	-2.112
C	-11.379	-5.6	-2.598
C	-10.303	-5.601	-1.7
C	-5.646	-8.45	-1.393
C	-9.749	-0.728	2.981
C	-9.261	-0.258	4.212
C	-10.011	0.617	5.001
C	-11.273	1.019	4.545
C	-11.78	0.583	3.317
C	-11.008	-0.295	2.549
C	-4.522	-9.103	-0.862
C	-4.097	-10.337	-1.36
C	-4.815	-10.92	-2.411
C	-5.941	-10.299	-2.96
C	-6.347	-9.066	-2.439
C	-5.63	0.23	-1.339
C	-4.931	1.332	-0.731
C	-5.63	2.547	-0.655
C	-7.02	2.565	-0.995

C	-7.753	1.436	-1.433
C	-6.969	0.252	-1.635
C	-7.702	3.768	-0.858
C	-9.048	3.979	-1.084
C	-9.795	2.856	-1.465
C	-9.167	1.601	-1.638
C	-4.968	3.76	-0.23
C	-5.698	4.987	-0.148
C	-7.05	4.955	-0.47
C	-3.594	3.765	0.053
C	-3.004	5.034	0.382
C	-3.721	6.206	0.464
C	-5.133	6.234	0.218
C	-6.01	7.375	0.263
C	-7.377	7.262	-0.079
C	-7.916	6.029	-0.466
C	-9.33	5.508	-0.885
C	-10.303	5.837	0.254
C	-9.808	6.063	-2.237
C	-10.886	7.108	0.314
C	-11.691	7.488	1.393
C	-11.9	6.57	2.426
C	-11.327	5.293	2.394
C	-10.533	4.937	1.299
C	-8.953	6.737	-3.106
C	-9.394	7.17	-4.366
C	-10.712	6.913	-4.741
C	-11.596	6.237	-3.886
C	-11.131	5.819	-2.641
C	-10.012	0.423	-1.972

C	-5.506	8.712	0.674
C	-5.77	9.839	-0.117
C	-5.334	11.113	0.263
C	-4.626	11.248	1.462
C	-4.352	10.145	2.278
C	-4.796	8.884	1.873
C	-9.724	-0.404	-3.067
C	-10.48	-1.55	-3.329
C	-11.559	-1.85	-2.491
C	-11.893	-1.028	-1.41
C	-11.113	0.107	-1.162
C	3.067	-4.886	-0.654
C	3.614	-3.652	-0.163
C	4.982	-3.644	0.15
C	5.751	-4.827	-0.071
C	5.229	-6.036	-0.594
C	3.821	-6.019	-0.863
C	7.096	-4.795	0.282
C	7.993	-5.838	0.173
C	7.49	-7.038	-0.346
C	6.137	-7.143	-0.74
C	5.592	-2.477	0.747
C	6.96	-2.513	1.161
C	7.686	-3.666	0.885
C	4.868	-1.285	0.909
C	5.51	-0.23	1.648
C	6.809	-0.299	2.089
C	7.619	-1.457	1.837
C	8.996	-1.662	2.196
C	9.676	-2.856	1.86

C	9.011	-3.896	1.2
C	9.359	-5.36	0.769
C	10.517	-5.353	-0.24
C	9.675	-6.278	1.965
C	11.811	-5.072	0.219
C	12.905	-5.067	-0.647
C	12.69	-5.361	-2
C	11.412	-5.645	-2.488
C	10.33	-5.625	-1.597
C	9.322	-5.936	3.27
C	9.541	-6.823	4.335
C	10.117	-8.065	4.069
C	10.479	-8.436	2.765
C	10.252	-7.535	1.726
C	5.676	-8.441	-1.299
C	9.749	-0.649	2.981
C	9.253	-0.159	4.2
C	9.998	0.727	4.981
C	11.264	1.121	4.527
C	11.779	0.665	3.31
C	11.012	-0.225	2.55
C	5.005	-8.505	-2.531
C	4.598	-9.727	-3.073
C	4.872	-10.901	-2.361
C	5.542	-10.873	-1.133
C	5.941	-9.637	-0.616
C	2.991	5.047	0.24
C	3.582	3.772	-0.057
C	4.957	3.761	-0.34
C	5.686	4.99	-0.285

C	5.12	6.244	0.054
C	3.708	6.221	0.297
C	7.039	4.951	-0.603
C	7.906	6.024	-0.619
C	7.366	7.264	-0.258
C	5.998	7.385	0.077
C	5.619	2.539	-0.737
C	7.009	2.55	-1.077
C	7.691	3.756	-0.965
C	4.921	1.322	-0.785
C	5.62	0.208	-1.37
C	6.959	0.223	-1.665
C	7.744	1.411	-1.487
C	9.159	1.57	-1.69
C	9.786	2.83	-1.543
C	9.039	3.961	-1.19
C	9.321	5.493	-1.021
C	9.807	6.021	-2.379
C	10.288	5.843	0.118
C	11.132	5.77	-2.771
C	11.605	6.167	-4.021
C	10.727	6.828	-4.892
C	9.406	7.091	-4.531
C	8.957	6.681	-3.266
C	10.873	7.113	0.157
C	11.672	7.513	1.233
C	11.871	6.616	2.286
C	11.295	5.34	2.276
C	10.508	4.963	1.183
C	5.494	8.73	0.461

C	10.005	0.385	-1.992
C	9.721	-0.471	-3.066
C	10.477	-1.623	-3.295
C	11.555	-1.9	-2.448
C	11.886	-1.048	-1.389
C	11.105	0.091	-1.172
C	4.779	8.926	1.653
C	4.336	10.196	2.033
C	4.616	11.283	1.197
C	5.329	11.123	0.004
C	5.764	9.843	-0.35
C	-6.683	-10.923	-4.12
C	-2.912	-11.05	-0.751
C	-9.472	1.135	6.314
C	-13.114	1.072	2.806
C	-13.046	-1.394	-0.507
C	-10.117	-2.471	-4.469
C	-5.587	12.308	-0.625
C	-3.629	10.323	3.593
C	5.793	-12.147	-0.36
C	3.915	-9.787	-4.419
C	9.45	1.267	6.281
C	13.117	1.145	2.8
C	5.589	12.302	-0.905
C	3.608	10.399	3.341
C	10.117	-2.575	-4.411
C	13.041	-1.389	-0.476
C	-13.025	5.989	-4.307
C	-8.441	7.894	-5.288
C	-12.337	8.853	1.421

C	-11.563	4.322	3.525
C	8.455	7.78	-5.481
C	13.039	5.919	-4.424
C	12.321	8.877	1.239
C	11.519	4.392	3.429
C	9.138	-6.427	5.736
C	11.128	-9.775	2.503
C	14.299	-4.794	-0.132
C	11.207	-6.009	-3.939
C	-9.225	-6.56	5.63
C	-11.112	-9.86	2.288
C	-14.281	-4.805	-0.242
C	-11.165	-5.93	-4.056
H	-0.958	-3.291	-0.458
H	-0.955	3.391	0.317
H	0.955	-3.299	-0.382
H	0.946	3.4	0.228
H	-2.047	-4.854	-1.167
H	-3.366	-6.832	-1.541
H	-8.104	-7.928	-0.572
H	-4.943	0.618	1.887
H	-7.228	0.474	2.657
H	-10.705	-3.023	2.127
H	-10.544	-7.83	0.558
H	-10.355	-8.876	4.712
H	-8.873	-5.076	3.392
H	-11.961	-4.923	1.18
H	-13.502	-5.32	-2.802
H	-9.305	-5.807	-2.07
H	-8.296	-0.608	4.568

H	-11.869	1.691	5.158
H	-11.381	-0.647	1.593
H	-3.99	-8.649	-0.031
H	-4.49	-11.88	-2.809
H	-7.212	-8.562	-2.862
H	-5.079	-0.675	-1.553
H	-7.429	-0.644	-2.031
H	-10.861	2.925	-1.656
H	-1.941	5.084	0.589
H	-3.202	7.125	0.711
H	-8.001	8.147	-0.007
H	-10.713	7.811	-0.496
H	-12.517	6.855	3.276
H	-10.078	3.951	1.268
H	-7.927	6.929	-2.81
H	-11.065	7.245	-5.715
H	-11.806	5.307	-1.962
H	-6.309	9.712	-1.052
H	-4.282	12.235	1.767
H	-4.612	8.021	2.507
H	-8.901	-0.144	-3.727
H	-12.144	-2.746	-2.675
H	-11.338	0.745	-0.312
H	2.01	-4.936	-0.895
H	3.335	-6.915	-1.232
H	8.129	-7.907	-0.467
H	4.943	0.669	1.854
H	7.229	0.546	2.622
H	10.708	-2.96	2.177
H	11.968	-4.88	1.277

H	13.537	-5.371	-2.684
H	9.334	-5.839	-1.97
H	8.869	-4.971	3.47
H	10.291	-8.76	4.888
H	10.537	-7.805	0.713
H	8.285	-0.503	4.555
H	11.856	1.803	5.133
H	11.391	-0.592	1.603
H	4.819	-7.586	-3.08
H	4.557	-11.858	-2.773
H	6.449	-9.593	0.343
H	1.928	5.102	0.445
H	3.188	7.146	0.522
H	7.991	8.151	-0.201
H	5.068	-0.701	-1.566
H	7.419	-0.682	-2.041
H	10.853	2.894	-1.731
H	11.803	5.27	-2.079
H	11.087	7.144	-5.869
H	7.93	6.881	-2.978
H	10.707	7.801	-0.668
H	12.483	6.916	3.134
H	10.051	3.979	1.168
H	8.899	-0.228	-3.734
H	12.14	-2.801	-2.607
H	11.328	0.751	-0.339
H	4.591	8.076	2.302
H	4.273	12.276	1.482
H	6.308	9.697	-1.28
H	-6.561	-12.01	-4.135

H	-7.753	-10.701	-4.076
H	-6.311	-10.538	-5.077
H	-2.251	-10.354	-0.226
H	-2.323	-11.57	-1.513
H	-3.238	-11.804	-0.023
H	-8.668	0.5	6.698
H	-9.064	2.147	6.197
H	-10.256	1.189	7.076
H	-13.723	1.497	3.61
H	-12.979	1.854	2.049
H	-13.686	0.262	2.341
H	-13.875	-1.826	-1.075
H	-12.738	-2.148	0.228
H	-13.424	-0.525	0.039
H	-9.5	-1.965	-5.217
H	-9.551	-3.335	-4.099
H	-11.008	-2.863	-4.969
H	-6.51	12.19	-1.202
H	-5.666	13.231	-0.043
H	-4.77	12.445	-1.344
H	-2.87	11.109	3.53
H	-3.135	9.398	3.907
H	-4.326	10.608	4.391
H	5.901	-13.007	-1.027
H	6.7	-12.073	0.249
H	4.961	-12.364	0.321
H	3.429	-8.838	-4.665
H	3.155	-10.574	-4.447
H	4.635	-10.002	-5.218
H	10.229	1.334	7.047

H	8.644	0.639	6.67
H	9.043	2.277	6.144
H	13.723	1.576	3.602
H	12.987	1.921	2.035
H	13.688	0.33	2.344
H	5.67	13.235	-0.339
H	6.512	12.17	-1.478
H	4.773	12.429	-1.628
H	3.1	9.485	3.664
H	2.86	11.194	3.264
H	4.305	10.685	4.139
H	9.554	-3.43	-4.018
H	11.009	-2.976	-4.901
H	9.498	-2.09	-5.171
H	13.874	-1.827	-1.035
H	13.412	-0.506	0.053
H	12.737	-2.13	0.273
H	-13.6	6.923	-4.325
H	-13.074	5.562	-5.315
H	-13.532	5.301	-3.625
H	-8.932	8.203	-6.215
H	-7.589	7.258	-5.555
H	-8.031	8.791	-4.808
H	-12.568	9.167	2.444
H	-13.278	8.855	0.857
H	-11.689	9.611	0.971
H	-10.945	3.428	3.423
H	-12.609	3.996	3.559
H	-11.338	4.78	4.495
H	8.99	8.276	-6.297

H	7.759	7.062	-5.931
H	7.85	8.532	-4.964
H	13.521	5.19	-3.766
H	13.105	5.543	-5.45
H	13.628	6.843	-4.38
H	13.27	8.863	0.688
H	12.538	9.214	2.257
H	11.681	9.626	0.762
H	12.567	4.075	3.488
H	10.91	3.491	3.334
H	11.273	4.866	4.386
H	9.475	-7.159	6.475
H	8.049	-6.34	5.824
H	9.56	-5.453	6.009
H	11.074	-10.046	1.445
H	10.649	-10.571	3.083
H	12.188	-9.762	2.786
H	14.277	-4.217	0.797
H	14.893	-4.236	-0.862
H	14.832	-5.73	0.075
H	11.849	-5.414	-4.597
H	10.17	-5.856	-4.251
H	11.451	-7.064	-4.116
H	-9.937	-5.828	6.03
H	-8.231	-6.106	5.686
H	-9.239	-7.43	6.293
H	-11.845	-10.152	3.047
H	-10.341	-10.639	2.266
H	-11.611	-9.864	1.315
H	-14.875	-4.24	-0.967

H	-14.267	-4.24	0.695
H	-14.811	-5.746	-0.05
H	-11.794	-5.313	-4.705
H	-10.124	-5.779	-4.355
H	-11.417	-6.978	-4.261

1b:

(*P*[5], *M*[6])-**1b** in the ground state				(*P*[5], *M*[6])-**1b** in the first excited state			
C	1.59	1.147	0.688	C	1.597	1.158	0.7
C	0.245	1.406	0.362	C	0.263	1.415	0.407
C	-0.622	0.434	-0.081	C	-0.636	0.426	-0.003
C	-0.166	-0.917	-0.128	C	-0.189	-0.931	-0.031
C	1.225	-1.194	-0.022	C	1.21	-1.208	0.051
C	2.097	-0.187	0.57	C	2.097	-0.196	0.6
C	-1.125	-1.979	-0.229	C	-1.149	-1.987	-0.132
C	-0.658	-3.277	-0.29	C	-0.677	-3.304	-0.161
C	-4.925	-2.278	0.154	C	-4.934	-2.302	0.14
C	1.655	-2.494	-0.502	C	1.628	-2.513	-0.418
C	-2	0.752	-0.468	C	-1.989	0.743	-0.373
C	-2.966	-0.268	-0.337	C	-2.965	-0.285	-0.265
C	-2.557	-1.636	-0.136	C	-2.564	-1.647	-0.09
C	-2.399	2.071	-0.826	C	-2.391	2.084	-0.713
C	-3.756	2.443	-0.528	C	-3.734	2.458	-0.366
C	-4.687	1.43	-0.279	C	-4.664	1.44	-0.126
C	-4.361	0.066	-0.341	C	-4.355	0.066	-0.247
C	-5.362	-0.983	-0.249	C	-5.36	-0.967	-0.211
C	0.719	-3.574	-0.445	C	0.686	-3.598	-0.312
C	-3.528	-2.564	0.165	C	-3.563	-2.598	0.163
C	4.96	6.856	1.935	C	5.043	6.861	1.81
C	8.254	2.87	2.708	C	8.312	2.861	2.585

C	7.796	1.394	2.369	C	7.838	1.384	2.272
C	8.382	0.116	2.364	C	8.416	0.104	2.27
C	7.595	-1.037	2.142	C	7.615	-1.048	2.078
C	6.182	-0.943	1.893	C	6.2	-0.948	1.857
C	5.248	-2.019	1.729	C	5.256	-2.021	1.727
C	3.936	-1.777	1.412	C	3.94	-1.776	1.435
C	3.411	-0.467	1.129	C	3.413	-0.468	1.143
C	6.443	1.437	2.086	C	6.478	1.433	2.014
C	5.615	0.351	1.816	C	5.637	0.349	1.774
C	4.253	0.614	1.455	C	4.272	0.616	1.438
C	5.265	5.404	2.034	C	5.335	5.409	1.928
C	6.578	5.02	2.388	C	6.649	5.018	2.266
C	6.935	3.664	2.436	C	6.995	3.66	2.327
C	5.952	2.759	2.096	C	5.999	2.758	2.016
C	2.88	4.668	1.423	C	2.936	4.682	1.363
C	4.256	4.425	1.738	C	4.31	4.432	1.661
C	4.64	3.062	1.756	C	4.683	3.066	1.693
C	3.773	1.977	1.422	C	3.802	1.983	1.39
C	2.44	2.252	1.093	C	2.469	2.267	1.073
C	2.026	3.63	1.125	C	2.069	3.644	1.088
C	-6.092	8.711	0.496	C	-5.98	8.745	0.723
C	-1.188	9.105	-1.336	C	-1.148	9.101	-1.292
C	-2.544	8.492	-0.817	C	-2.494	8.503	-0.733
C	-3.753	8.989	-0.305	C	-3.67	9.008	-0.171
C	-4.816	8.117	0.017	C	-4.73	8.14	0.196
C	-4.68	6.689	-0.121	C	-4.611	6.712	0.057
C	-5.634	5.684	0.238	C	-5.547	5.713	0.479
C	-5.36	4.344	0.068	C	-5.287	4.369	0.31
C	-4.111	3.856	-0.439	C	-4.077	3.869	-0.265
C	-2.474	7.123	-0.98	C	-2.444	7.127	-0.905

C	-3.455	6.198	-0.634	C	-3.417	6.211	-0.516
C	-3.148	4.812	-0.793	C	-3.125	4.823	-0.684
C	1.3	6.2	-2.418	C	1.277	6.16	-2.428
C	0.874	7.535	-2.221	C	0.869	7.508	-2.226
C	-0.427	7.816	-1.789	C	-0.414	7.804	-1.766
C	-1.24	6.73	-1.538	C	-1.236	6.724	-1.497
C	0.63	3.709	-2.478	C	0.592	3.675	-2.457
C	0.395	5.099	-2.212	C	0.371	5.073	-2.199
C	-0.899	5.395	-1.716	C	-0.912	5.382	-1.677
C	-1.857	4.409	-1.319	C	-1.868	4.413	-1.254
C	-1.504	3.059	-1.407	C	-1.523	3.042	-1.332
C	-0.282	2.752	-2.105	C	-0.311	2.726	-2.051
C	-8.721	-7.499	2.203	C	-8.845	-7.533	1.97
C	-11.671	1.133	-1.366	C	-11.626	1.275	-1.294
C	-11.947	-3.678	0.682	C	-11.991	-3.604	0.564
C	-10.635	-4.454	1.036	C	-10.695	-4.415	0.896
C	-10.324	-5.722	1.539	C	-10.411	-5.707	1.356
C	-8.977	-6.13	1.685	C	-9.074	-6.142	1.497
C	-7.89	-5.254	1.336	C	-7.967	-5.271	1.189
C	-6.485	-5.506	1.476	C	-6.572	-5.551	1.334
C	-5.55	-4.577	1.086	C	-5.617	-4.619	0.987
C	-5.889	-3.293	0.532	C	-5.929	-3.315	0.479
C	-9.563	-3.653	0.692	C	-9.608	-3.621	0.589
C	-8.218	-3.977	0.819	C	-8.268	-3.97	0.715
C	-7.258	-2.994	0.417	C	-7.289	-2.99	0.36
C	-11.052	-0.123	-0.866	C	-11.029	-0.008	-0.839
C	-11.904	-1.158	-0.41	C	-11.901	-1.046	-0.429
C	-11.368	-2.349	0.091	C	-11.386	-2.264	0.028
C	-9.99	-2.428	0.147	C	-10.01	-2.368	0.088
C	-8.644	0.622	-1.386	C	-8.607	0.722	-1.311

C	-9.623	-0.269	-0.835	C	-9.603	-0.176	-0.805
C	-9.091	-1.456	-0.276	C	-9.092	-1.395	-0.291
C	-7.695	-1.731	-0.127	C	-7.704	-1.695	-0.14
C	-6.762	-0.75	-0.521	C	-6.753	-0.712	-0.484
C	-7.3	0.382	-1.239	C	-7.268	0.455	-1.161
C	2.154	-10.198	-0.499	C	2.148	-10.22	-0.205
C	6.54	-7.592	-1.652	C	6.482	-7.641	-1.584
C	5.06	-7.889	-1.232	C	5.018	-7.928	-1.111
C	4.292	-9.037	-1	C	4.26	-9.071	-0.821
C	2.911	-8.936	-0.71	C	2.892	-8.964	-0.483
C	2.256	-7.656	-0.632	C	2.236	-7.683	-0.417
C	0.884	-7.392	-0.307	C	0.881	-7.412	-0.041
C	0.39	-6.107	-0.28	C	0.385	-6.124	-0.032
C	1.192	-4.945	-0.547	C	1.17	-4.974	-0.372
C	4.39	-6.683	-1.168	C	4.347	-6.722	-1.059
C	3.043	-6.505	-0.877	C	3.01	-6.536	-0.725
C	2.546	-5.165	-0.844	C	2.511	-5.197	-0.713
C	7.043	-3.679	-2.193	C	6.969	-3.739	-2.217
C	7.437	-5.037	-2.123	C	7.365	-5.097	-2.133
C	6.515	-6.034	-1.785	C	6.452	-6.085	-1.751
C	5.236	-5.604	-1.493	C	5.18	-5.65	-1.435
C	5.121	-1.963	-2.005	C	5.052	-2.018	-2.015
C	5.692	-3.277	-1.905	C	5.626	-3.332	-1.906
C	4.78	-4.291	-1.523	C	4.722	-4.338	-1.481
C	3.419	-4.058	-1.157	C	3.372	-4.096	-1.086
C	2.962	-2.734	-1.075	C	2.913	-2.768	-1.02
C	3.834	-1.714	-1.593	C	3.777	-1.761	-1.577
C	-11.227	2.385	-0.914	C	-11.167	2.503	-0.793
C	-11.811	3.572	-1.368	C	-11.731	3.715	-1.204
C	-12.861	3.492	-2.289	C	-12.777	3.686	-2.133

C	-13.333	2.259	-2.755	C	-13.264	2.478	-2.648
C	-12.731	1.09	-2.285	C	-12.682	1.283	-2.219
C	-14.441	2.197	-3.781	C	-14.367	2.472	-3.681
C	-11.338	4.909	-0.843	C	-11.241	5.023	-0.627
C	-9.351	-7.939	3.376	C	-9.497	-8.004	3.118
C	-9.149	-9.232	3.869	C	-9.321	-9.316	3.569
C	-8.305	-10.094	3.162	C	-8.478	-10.165	2.844
C	-7.667	-9.69	1.983	C	-7.818	-9.73	1.689
C	-7.882	-8.392	1.516	C	-8.008	-8.413	1.264
C	-9.798	-9.672	5.161	C	-9.993	-9.79	4.836
C	-6.797	-10.655	1.211	C	-6.95	-10.681	0.897
C	-12.729	-4.515	-0.341	C	-12.781	-4.388	-0.495
C	-13.495	-5.601	0.109	C	-13.573	-5.473	-0.091
C	-14.168	-6.433	-0.786	C	-14.254	-6.258	-1.022
C	-14.072	-6.16	-2.158	C	-14.138	-5.939	-2.382
C	-13.316	-5.089	-2.635	C	-13.354	-4.867	-2.813
C	-12.647	-4.271	-1.713	C	-12.678	-4.097	-1.855
C	-14.968	-7.616	-0.292	C	-15.082	-7.441	-0.578
C	-13.194	-4.816	-4.116	C	-13.213	-4.543	-4.281
C	-12.798	-3.318	1.908	C	-12.844	-3.277	1.799
C	-12.282	-3.37	3.203	C	-12.34	-3.395	3.095
C	-13.051	-2.971	4.306	C	-13.11	-3.028	4.207
C	-14.353	-2.518	4.088	C	-14.401	-2.538	3.999
C	-14.896	-2.449	2.797	C	-14.931	-2.404	2.709
C	-14.109	-2.855	1.719	C	-14.143	-2.78	1.62
C	-12.462	-3.018	5.696	C	-12.535	-3.148	5.599
C	-16.297	-1.927	2.581	C	-16.319	-1.844	2.502
C	0.976	-10.468	-1.213	C	0.937	-10.508	-0.855
C	0.28	-11.668	-1.038	C	0.251	-11.703	-0.616
C	0.78	-12.607	-0.13	C	0.795	-12.619	0.292

C	1.954	-12.372	0.596	C	2.002	-12.366	0.952
C	2.631	-11.165	0.399	C	2.668	-11.165	0.692
C	-0.96	-11.961	-1.849	C	-1.027	-12.017	-1.358
C	2.454	-13.383	1.6	C	2.554	-13.35	1.957
C	-6.094	9.663	1.526	C	-5.932	9.719	1.733
C	-7.283	10.248	1.973	C	-7.098	10.314	2.222
C	-8.487	9.866	1.37	C	-8.33	9.923	1.683
C	-8.521	8.92	0.339	C	-8.413	8.958	0.672
C	-7.317	8.358	-0.093	C	-7.231	8.384	0.197
C	-7.271	11.244	3.109	C	-7.035	11.331	3.338
C	-9.833	8.497	-0.281	C	-9.752	8.53	0.119
C	4.473	7.399	0.735	C	4.541	7.392	0.61
C	4.252	8.773	0.604	C	4.336	8.766	0.461
C	4.517	9.602	1.7	C	4.629	9.609	1.54
C	4.983	9.089	2.915	C	5.107	9.108	2.755
C	5.21	7.712	3.016	C	5.32	7.731	2.874
C	5.195	9.998	4.102	C	5.348	10.033	3.925
C	3.716	9.35	-0.682	C	3.79	9.332	-0.826
C	7.585	-8.003	-0.604	C	7.561	-8.033	-0.564
C	7.233	-8.453	0.668	C	7.25	-8.468	0.724
C	8.212	-8.832	1.6	C	8.258	-8.84	1.627
C	9.557	-8.728	1.242	C	9.591	-8.743	1.224
C	9.941	-8.263	-0.025	C	9.934	-8.292	-0.059
C	8.945	-7.913	-0.937	C	8.91	-7.951	-0.942
C	7.807	-9.387	2.945	C	7.895	-9.383	2.989
C	11.403	-8.126	-0.378	C	11.384	-8.159	-0.461
C	6.794	-8.304	-2.993	C	6.693	-8.38	-2.919
C	7.085	-9.676	-2.992	C	6.979	-9.754	-2.899
C	7.258	-10.381	-4.183	C	7.113	-10.483	-4.08
C	7.141	-9.687	-5.396	C	6.963	-9.813	-5.303

C	6.85	-8.323	-5.428	C	6.677	-8.448	-5.353
C	6.678	-7.64	-4.214	C	6.543	-7.741	-4.149
C	7.554	-11.862	-4.176	C	7.403	-11.965	-4.052
C	6.701	-7.583	-6.737	C	6.492	-7.734	-6.672
C	8.081	-2.682	-2.553	C	8	-2.75	-2.612
C	8.99	-2.943	-3.594	C	8.888	-3.025	-3.666
C	10.022	-2.055	-3.899	C	9.917	-2.143	-4.002
C	10.14	-0.879	-3.147	C	10.053	-0.959	-3.266
C	9.244	-0.582	-2.115	C	9.178	-0.648	-2.221
C	8.222	-1.492	-1.827	C	8.159	-1.551	-1.904
C	10.976	-2.337	-5.037	C	10.848	-2.442	-5.154
C	9.352	0.695	-1.325	C	9.305	0.638	-1.447
C	8.24	-2.379	2.075	C	8.252	-2.393	2.016
C	9.203	-2.641	1.093	C	9.192	-2.671	1.016
C	9.682	-3.937	0.873	C	9.663	-3.972	0.804
C	9.214	-4.972	1.686	C	9.209	-4.994	1.643
C	8.297	-4.731	2.717	C	8.315	-4.736	2.689
C	7.821	-3.431	2.904	C	7.846	-3.432	2.867
C	10.665	-4.212	-0.242	C	10.618	-4.267	-0.328
C	7.782	-5.871	3.564	C	7.814	-5.862	3.561
C	9.336	3.435	1.766	C	9.381	3.407	1.618
C	9.064	3.47	0.389	C	9.084	3.43	0.245
C	9.94	4.074	-0.512	C	9.947	4.019	-0.678
C	11.126	4.638	-0.019	C	11.146	4.579	-0.212
C	11.426	4.617	1.342	C	11.471	4.569	1.143
C	10.513	4.023	2.228	C	10.57	3.991	2.052
C	9.63	4.135	-1.99	C	9.61	4.067	-2.15
C	12.706	5.219	1.872	C	12.765	5.166	1.644
C	8.656	2.888	4.193	C	8.74	2.893	4.062
C	9.754	2.12	4.618	C	9.84	2.123	4.477

C	10.141	2.076	5.955	C	10.249	2.092	5.809
C	9.407	2.82	6.891	C	9.536	2.852	6.748
C	8.312	3.59	6.504	C	8.439	3.624	6.37
C	7.948	3.616	5.148	C	8.053	3.637	5.02
C	11.312	1.231	6.4	C	11.422	1.245	6.243
C	7.506	4.373	7.513	C	7.656	4.425	7.383
C	3.407	6.777	-3.669	C	3.356	6.723	-3.729
C	2.732	5.968	-2.741	C	2.699	5.919	-2.784
C	3.467	4.983	-2.058	C	3.445	4.932	-2.115
C	4.835	4.803	-2.284	C	4.806	4.746	-2.371
C	5.476	5.634	-3.211	C	5.429	5.57	-3.315
C	4.777	6.626	-3.907	C	4.719	6.563	-3.999
C	5.494	7.536	-4.877	C	5.418	7.466	-4.988
C	5.61	3.761	-1.513	C	5.593	3.705	-1.611
C	-1.498	10.658	-4.852	C	-1.537	10.66	-4.797
C	-1.155	9.787	-3.806	C	-1.174	9.785	-3.761
C	-1.503	10.069	-2.486	C	-1.486	10.069	-2.433
C	-2.21	11.254	-2.217	C	-2.176	11.259	-2.145
C	-2.564	12.138	-3.235	C	-2.548	12.147	-3.152
C	-2.199	11.825	-4.553	C	-2.219	11.833	-4.479
C	1.11	11.461	0.704	C	1.224	11.434	0.688
C	0.271	10.973	-0.312	C	0.355	10.954	-0.307
C	-0.347	9.728	-0.199	C	-0.272	9.716	-0.177
C	-0.093	8.951	0.944	C	0.003	8.936	0.959
C	0.76	9.396	1.952	C	0.884	9.373	1.945
C	1.337	10.669	1.827	C	1.471	10.64	1.805
C	-1.123	10.31	-6.273	C	-1.2	10.312	-6.228
C	-3.342	13.398	-2.935	C	-3.307	13.413	-2.831
C	1.8	12.794	0.538	C	1.923	12.759	0.504
C	1.118	8.515	3.124	C	1.263	8.49	3.109

H	-0.13	2.408	0.482	H	-0.116	2.416	0.536
H	-1.366	-4.096	-0.307	H	-1.386	-4.121	-0.159
H	-5.696	1.704	-0.015	H	-5.671	1.712	0.154
H	-3.222	-3.575	0.395	H	-3.26	-3.608	0.4
H	9.443	-0.028	2.541	H	9.479	-0.046	2.427
H	5.572	-3.043	1.863	H	5.577	-3.045	1.867
H	3.268	-2.623	1.355	H	3.266	-2.62	1.4
H	7.308	5.799	2.583	H	7.389	5.794	2.437
H	2.504	5.685	1.401	H	2.566	5.701	1.331
H	1.005	3.888	0.873	H	1.048	3.906	0.838
H	-3.92	10.054	-0.183	H	-3.823	10.073	-0.039
H	-6.583	5.975	0.674	H	-6.467	6.013	0.967
H	-6.118	3.633	0.376	H	-6.025	3.663	0.675
H	1.595	8.333	-2.355	H	1.597	8.296	-2.378
H	1.535	3.406	-2.992	H	1.482	3.364	-2.991
H	-0.066	1.715	-2.331	H	-0.106	1.685	-2.272
H	-11.106	-6.431	1.793	H	-11.208	-6.41	1.576
H	-6.15	-6.44	1.914	H	-6.253	-6.504	1.741
H	-4.506	-4.824	1.241	H	-4.578	-4.889	1.141
H	-12.976	-0.992	-0.432	H	-12.97	-0.862	-0.451
H	-8.965	1.482	-1.963	H	-8.911	1.609	-1.855
H	-6.61	1.053	-1.734	H	-6.563	1.136	-1.621
H	4.729	-10.028	-1.075	H	4.697	-10.062	-0.886
H	0.224	-8.215	-0.057	H	0.234	-8.227	0.265
H	-0.65	-5.975	-0.002	H	-0.64	-5.982	0.292
H	8.476	-5.278	-2.318	H	8.399	-5.341	-2.35
H	5.71	-1.149	-2.41	H	5.632	-1.213	-2.45
H	3.452	-0.703	-1.663	H	3.394	-0.75	-1.656
H	-10.426	2.429	-0.182	H	-10.369	2.508	-0.056
H	-13.321	4.409	-2.651	H	-13.221	4.622	-2.464

H	-13.072	0.125	-2.652	H	-13.035	0.338	-2.624
H	-14.04	2.281	-4.798	H	-13.961	2.597	-4.692
H	-14.989	1.252	-3.722	H	-14.924	1.531	-3.667
H	-15.158	3.014	-3.646	H	-15.077	3.289	-3.514
H	-10.262	4.903	-0.642	H	-10.166	4.994	-0.422
H	-11.545	5.715	-1.552	H	-11.432	5.859	-1.306
H	-11.842	5.164	0.098	H	-11.746	5.251	0.321
H	-9.993	-7.251	3.919	H	-10.137	-7.325	3.676
H	-8.14	-11.103	3.536	H	-8.333	-11.188	3.186
H	-7.413	-8.07	0.59	H	-7.522	-8.067	0.356
H	-10.765	-9.183	5.31	H	-10.957	-9.294	4.987
H	-9.957	-10.754	5.182	H	-10.166	-10.87	4.819
H	-9.169	-9.418	6.023	H	-9.374	-9.572	5.715
H	-6.222	-11.3	1.883	H	-6.373	-11.338	1.556
H	-7.405	-11.311	0.575	H	-7.559	-11.324	0.25
H	-6.094	-10.129	0.56	H	-6.247	-10.142	0.255
H	-13.579	-5.784	1.176	H	-13.672	-5.693	0.968
H	-14.604	-6.795	-2.864	H	-14.675	-6.536	-3.116
H	-12.06	-3.433	-2.075	H	-12.071	-3.259	-2.181
H	-15.242	-7.504	0.761	H	-15.395	-7.343	0.466
H	-15.888	-7.746	-0.871	H	-15.981	-7.554	-1.193
H	-14.392	-8.545	-0.385	H	-14.512	-8.374	-0.664
H	-13.217	-3.742	-4.327	H	-13.228	-3.462	-4.455
H	-12.246	-5.201	-4.509	H	-12.26	-4.918	-4.676
H	-14.002	-5.291	-4.68	H	-14.015	-4.994	-4.873
H	-11.27	-3.728	3.364	H	-11.337	-3.781	3.248
H	-14.962	-2.216	4.938	H	-15.011	-2.26	4.856
H	-14.523	-2.83	0.715	H	-14.549	-2.704	0.615
H	-11.782	-2.174	5.863	H	-11.825	-2.337	5.801
H	-13.239	-2.971	6.465	H	-13.315	-3.101	6.364

H	-11.882	-3.934	5.853	H	-11.99	-4.089	5.725
H	-16.315	-0.83	2.598	H	-16.308	-0.747	2.531
H	-16.699	-2.247	1.615	H	-16.732	-2.142	1.534
H	-16.979	-2.274	3.364	H	-17.007	-2.18	3.284
H	0.615	-9.738	-1.932	H	0.54	-9.798	-1.574
H	0.244	-13.543	0.015	H	0.267	-13.551	0.486
H	3.536	-10.958	0.964	H	3.599	-10.944	1.207
H	-1.496	-11.043	-2.109	H	-1.566	-11.105	-1.631
H	-0.706	-12.466	-2.789	H	-0.819	-12.563	-2.287
H	-1.649	-12.614	-1.305	H	-1.696	-12.641	-0.757
H	2.237	-14.407	1.28	H	2.258	-14.376	1.718
H	3.534	-13.297	1.752	H	3.647	-13.312	1.996
H	1.974	-13.239	2.576	H	2.185	-13.131	2.967
H	-5.152	9.936	1.994	H	-4.97	9.999	2.151
H	-9.418	10.317	1.71	H	-9.243	10.383	2.055
H	-7.322	7.649	-0.916	H	-7.276	7.661	-0.612
H	-6.325	11.792	3.151	H	-6.069	11.843	3.36
H	-8.082	11.972	3.013	H	-7.819	12.088	3.234
H	-7.399	10.74	4.075	H	-7.174	10.852	4.315
H	-10.561	9.314	-0.279	H	-10.474	9.353	0.128
H	-9.699	8.163	-1.314	H	-9.664	8.167	-0.909
H	-10.279	7.662	0.275	H	-10.181	7.714	0.715
H	4.296	6.745	-0.113	H	4.343	6.726	-0.225
H	4.352	10.673	1.604	H	4.475	10.679	1.429
H	5.579	7.292	3.949	H	5.7	7.32	3.806
H	5.537	10.991	3.791	H	5.685	11.02	3.594
H	4.261	10.136	4.661	H	4.426	10.18	4.502
H	5.933	9.587	4.798	H	6.099	9.629	4.611
H	2.632	9.487	-0.611	H	2.714	9.514	-0.731
H	4.153	10.332	-0.892	H	4.259	10.29	-1.07

H	3.922	8.692	-1.532	H	3.947	8.647	-1.663
H	6.185	-8.528	0.941	H	6.212	-8.537	1.032
H	10.324	-9.018	1.957	H	10.38	-9.028	1.917
H	9.225	-7.588	-1.935	H	9.158	-7.64	-1.952
H	7.61	-10.464	2.881	H	7.688	-10.459	2.939
H	8.591	-9.241	3.693	H	8.706	-9.239	3.709
H	6.894	-8.911	3.316	H	7	-8.897	3.388
H	11.981	-8.988	-0.03	H	11.981	-8.999	-0.091
H	11.548	-8.033	-1.458	H	11.496	-8.115	-1.548
H	11.837	-7.234	0.091	H	11.822	-7.242	-0.049
H	7.195	-10.195	-2.044	H	7.115	-10.254	-1.944
H	7.286	-10.224	-6.332	H	7.077	-10.369	-6.232
H	6.453	-6.579	-4.231	H	6.323	-6.679	-4.18
H	6.677	-12.44	-4.493	H	6.517	-12.545	-4.339
H	8.369	-12.11	-4.864	H	8.201	-12.229	-4.754
H	7.836	-12.21	-3.178	H	7.707	-12.296	-3.055
H	5.645	-7.387	-6.959	H	5.43	-7.542	-6.869
H	7.208	-6.613	-6.706	H	6.998	-6.763	-6.673
H	7.114	-8.156	-7.572	H	6.883	-8.322	-7.507
H	8.879	-3.854	-4.177	H	8.765	-3.943	-4.235
H	10.946	-0.182	-3.369	H	10.856	-0.267	-3.511
H	7.561	-1.297	-0.99	H	7.512	-1.344	-1.057
H	10.669	-1.81	-5.949	H	10.536	-1.911	-6.062
H	11.991	-2.005	-4.798	H	11.873	-2.126	-4.932
H	11.014	-3.404	-5.273	H	10.866	-3.51	-5.39
H	10.359	1.12	-1.357	H	10.315	1.055	-1.499
H	8.669	1.452	-1.725	H	8.622	1.395	-1.846
H	9.08	0.549	-0.276	H	9.048	0.506	-0.393
H	9.535	-1.834	0.449	H	9.512	-1.875	0.353
H	9.551	-5.989	1.507	H	9.541	-6.014	1.471

H	7.096	-3.232	3.688	H	7.138	-3.219	3.663
H	10.52	-5.219	-0.645	H	10.438	-5.267	-0.734
H	10.547	-3.497	-1.061	H	10.508	-3.545	-1.142
H	11.701	-4.144	0.112	H	11.662	-4.233	0.009
H	7.016	-6.435	3.02	H	7.031	-6.428	3.042
H	8.578	-6.579	3.812	H	8.611	-6.572	3.799
H	7.337	-5.512	4.496	H	7.392	-5.489	4.499
H	8.136	3.041	0.027	H	8.146	3.004	-0.096
H	11.816	5.115	-0.712	H	11.827	5.044	-0.922
H	10.73	4.04	3.29	H	10.807	4.017	3.109
H	8.568	3.962	-2.184	H	8.54	3.923	-2.32
H	9.895	5.112	-2.408	H	9.895	5.029	-2.589
H	10.193	3.378	-2.548	H	10.139	3.284	-2.707
H	12.52	5.833	2.76	H	12.6	5.786	2.531
H	13.417	4.437	2.167	H	13.477	4.382	1.928
H	13.197	5.846	1.123	H	13.245	5.787	0.881
H	10.328	1.563	3.884	H	10.398	1.554	3.741
H	9.701	2.796	7.939	H	9.848	2.838	7.79
H	7.095	4.214	4.847	H	7.198	4.236	4.728
H	10.972	0.344	6.947	H	11.084	0.361	6.798
H	11.905	0.886	5.548	H	12.003	0.893	5.386
H	11.974	1.789	7.071	H	12.094	1.803	6.903
H	8.019	4.437	8.477	H	8.181	4.489	8.34
H	7.314	5.393	7.163	H	7.473	5.445	7.029
H	6.53	3.905	7.686	H	6.676	3.971	7.571
H	2.849	7.528	-4.222	H	2.79	7.476	-4.271
H	2.974	4.373	-1.312	H	2.965	4.329	-1.354
H	6.541	5.505	-3.393	H	6.489	5.436	-3.521
H	4.814	7.927	-5.639	H	4.723	7.856	-5.738
H	5.93	8.397	-4.355	H	5.868	8.327	-4.48

H	6.312	7.017	-5.385	H	6.223	6.941	-5.512
H	4.949	3.004	-1.083	H	4.94	2.953	-1.162
H	6.338	3.253	-2.153	H	6.306	3.191	-2.264
H	6.159	4.217	-0.681	H	6.163	4.165	-0.794
H	-0.612	8.877	-4.035	H	-0.644	8.871	-4.006
H	-2.464	11.5	-1.191	H	-2.402	11.505	-1.112
H	-2.465	12.51	-5.355	H	-2.5	12.522	-5.273
H	0.117	11.577	-1.199	H	0.183	11.56	-1.189
H	-0.55	7.97	1.03	H	-0.461	7.958	1.056
H	1.996	11.03	2.613	H	2.153	10.995	2.574
H	-0.081	9.976	-6.339	H	-0.162	9.976	-6.322
H	-1.746	9.493	-6.656	H	-1.835	9.497	-6.595
H	-1.248	11.164	-6.944	H	-1.341	11.167	-6.895
H	-2.927	14.26	-3.467	H	-2.888	14.275	-3.361
H	-4.388	13.298	-3.249	H	-4.358	13.329	-3.132
H	-3.34	13.626	-1.866	H	-3.288	13.633	-1.759
H	2.664	12.703	-0.132	H	2.777	12.656	-0.178
H	1.131	13.542	0.1	H	1.256	13.511	0.071
H	2.164	13.181	1.494	H	2.305	13.148	1.452
H	1.065	9.064	4.07	H	1.223	9.036	4.058
H	0.455	7.648	3.197	H	0.604	7.621	3.19
H	2.146	8.147	3.021	H	2.291	8.126	2.99

1c:

(*P*[4], *M*[6])-**1c** in the ground state				(*P*[4], *M*[6])-**1b** in the first excited state			
C	-1.966	1.095	-0.852	C	-1.989	1.096	-0.907
C	-0.631	1.541	-0.592	C	-0.659	1.542	-0.66
C	0.42	0.613	-0.593	C	0.408	0.607	-0.697
C	0.115	-0.785	-0.59	C	0.107	-0.793	-0.703
C	-1.237	-1.226	-0.532	C	-1.246	-1.234	-0.623

C	-2.264	-0.29	-0.959	C	-2.284	-0.294	-1.027
C	1.179	-1.744	-0.573	C	1.175	-1.746	-0.704
C	0.864	-3.079	-0.4	C	0.856	-3.094	-0.554
C	-0.444	-3.519	-0.086	C	-0.439	-3.532	-0.224
C	-1.499	-2.553	-0.026	C	-1.497	-2.561	-0.126
C	1.792	1.053	-0.47	C	1.761	1.051	-0.608
C	2.852	0.117	-0.535	C	2.831	0.111	-0.681
C	2.564	-1.275	-0.725	C	2.55	-1.278	-0.839
C	2.065	2.427	-0.276	C	2.033	2.436	-0.425
C	3.423	2.895	-0.232	C	3.391	2.908	-0.419
C	4.442	1.955	-0.388	C	4.41	1.966	-0.584
C	4.209	0.567	-0.46	C	4.186	0.572	-0.617
C	5.291	-0.403	-0.533	C	5.27	-0.389	-0.636
C	4.97	-1.704	-1.026	C	4.961	-1.721	-1.083
C	3.607	-2.109	-1.064	C	3.617	-2.13	-1.131
C	-2.712	3.495	-0.849	C	-2.741	3.495	-0.868
C	-2.964	2.093	-1.07	C	-2.991	2.094	-1.103
C	-4.231	1.703	-1.511	C	-4.268	1.704	-1.522
C	-5.188	2.713	-1.812	C	-5.24	2.711	-1.775
C	-4.914	4.107	-1.772	C	-4.978	4.11	-1.711
C	-3.631	4.459	-1.267	C	-3.691	4.464	-1.247
C	-6.439	2.294	-2.239	C	-6.498	2.291	-2.177
C	-7.482	3.107	-2.627	C	-7.561	3.103	-2.519
C	-7.213	4.483	-2.66	C	-7.306	4.482	-2.534
C	-5.953	4.988	-2.264	C	-6.043	4.993	-2.162
C	-4.522	0.308	-1.725	C	-4.556	0.311	-1.747
C	-5.825	-0.077	-2.173	C	-5.866	-0.076	-2.164
C	-6.759	0.93	-2.389	C	-6.812	0.929	-2.34
C	-3.542	-0.682	-1.523	C	-3.564	-0.68	-1.575
C	-3.888	-2.012	-1.958	C	-3.918	-2.007	-2.015

C	-5.143	-2.367	-2.384	C	-5.181	-2.362	-2.416
C	-6.211	-1.408	-2.451	C	-6.255	-1.406	-2.446
C	-7.575	-1.635	-2.847	C	-7.627	-1.635	-2.805
C	-8.471	-0.557	-3.046	C	-8.536	-0.558	-2.968
C	-8.053	0.764	-2.846	C	-8.119	0.76	-2.762
C	-8.701	2.188	-2.959	C	-8.781	2.181	-2.834
C	-9.233	2.535	-4.352	C	-9.348	2.55	-4.208
C	-9.787	2.276	-1.869	C	-9.84	2.243	-1.716
C	-10.064	3.654	-4.508	C	-10.183	3.671	-4.325
C	-10.506	4.056	-5.77	C	-10.659	4.092	-5.568
C	-10.111	3.311	-6.889	C	-10.294	3.365	-6.708
C	-9.283	2.193	-6.763	C	-9.462	2.246	-6.621
C	-8.849	1.816	-5.485	C	-8.994	1.849	-5.362
C	-11.042	1.697	-2.098	C	-11.103	1.676	-1.928
C	-12.03	1.696	-1.111	C	-12.066	1.652	-0.916
C	-11.746	2.291	0.125	C	-11.748	2.213	0.328
C	-10.499	2.864	0.386	C	-10.491	2.773	0.571
C	-9.528	2.853	-0.623	C	-9.547	2.785	-0.462
C	-5.712	6.445	-2.447	C	-5.822	6.454	-2.319
C	-8.081	-3.02	-3.015	C	-8.133	-3.02	-2.968
C	-7.792	-3.996	-2.048	C	-7.809	-4.003	-2.019
C	-8.278	-5.298	-2.159	C	-8.293	-5.306	-2.127
C	-9.073	-5.625	-3.264	C	-9.12	-5.628	-3.21
C	-9.384	-4.677	-4.245	C	-9.466	-4.672	-4.173
C	-8.883	-3.378	-4.108	C	-8.967	-3.372	-4.039
C	-5.216	7.247	-1.415	C	-5.285	7.236	-1.29
C	-4.987	8.618	-1.593	C	-5.068	8.61	-1.447
C	-5.269	9.184	-2.838	C	-5.401	9.203	-2.668
C	-5.776	8.411	-3.894	C	-5.95	8.452	-3.719
C	-5.995	7.049	-3.686	C	-6.158	7.085	-3.531

C	-3.721	-1.968	1.109	C	-3.694	-1.993	1.065
C	-2.744	-2.912	0.631	C	-2.728	-2.93	0.554
C	-3.001	-4.267	0.896	C	-2.976	-4.288	0.816
C	-4.272	-4.635	1.443	C	-4.228	-4.661	1.4
C	-5.301	-3.716	1.757	C	-5.249	-3.746	1.753
C	-4.939	-2.335	1.622	C	-4.895	-2.365	1.613
C	-4.491	-5.976	1.74	C	-4.438	-6.005	1.693
C	-5.617	-6.509	2.342	C	-5.544	-6.543	2.326
C	-6.666	-5.613	2.595	C	-6.586	-5.651	2.619
C	-6.535	-4.241	2.274	C	-6.467	-4.278	2.302
C	-1.988	-5.275	0.675	C	-1.971	-5.297	0.55
C	-2.248	-6.642	1.009	C	-2.223	-6.667	0.877
C	-3.495	-6.954	1.545	C	-3.451	-6.982	1.454
C	-0.705	-4.915	0.23	C	-0.703	-4.935	0.068
C	0.268	-5.966	0.13	C	0.26	-5.988	-0.084
C	0.003	-7.278	0.449	C	0.002	-7.304	0.227
C	-1.29	-7.682	0.915	C	-1.273	-7.709	0.736
C	-1.686	-8.994	1.354	C	-1.656	-9.026	1.176
C	-2.959	-9.223	1.92	C	-2.908	-9.258	1.786
C	-3.89	-8.182	2.039	C	-3.83	-8.214	1.948
C	-5.301	-8.006	2.691	C	-5.218	-8.043	2.65
C	-6.285	-9.06	2.178	C	-6.218	-9.09	2.155
C	-5.225	-8.06	4.236	C	-5.09	-8.112	4.191
C	-6.064	-10.402	2.528	C	-5.996	-10.435	2.495
C	-6.916	-11.416	2.095	C	-6.858	-11.443	2.07
C	-8.028	-11.067	1.314	C	-7.981	-11.087	1.308
C	-8.287	-9.742	0.968	C	-8.239	-9.759	0.971
C	-7.399	-8.743	1.404	C	-7.341	-8.766	1.399
C	-4.02	-7.938	4.926	C	-3.863	-7.991	4.84
C	-3.988	-7.917	6.329	C	-3.783	-7.986	6.241

C	-5.189	-8.019	7.031	C	-4.958	-8.104	6.983
C	-6.416	-8.138	6.362	C	-6.207	-8.221	6.355
C	-6.418	-8.16	4.968	C	-6.257	-8.226	4.962
C	-7.708	-3.345	2.442	C	-7.639	-3.387	2.501
C	-0.753	-10.147	1.25	C	-0.734	-10.181	1.022
C	-0.51	-10.964	2.364	C	-0.453	-11.013	2.115
C	0.345	-12.067	2.284	C	0.39	-12.121	1.989
C	0.957	-12.353	1.059	C	0.954	-12.395	0.739
C	0.73	-11.564	-0.075	C	0.69	-11.589	-0.375
C	-0.125	-10.464	0.035	C	-0.153	-10.486	-0.22
C	-8.514	-3.4	3.592	C	-8.414	-3.441	3.671
C	-9.64	-2.586	3.724	C	-9.542	-2.632	3.829
C	-9.968	-1.71	2.678	C	-9.901	-1.763	2.788
C	-9.19	-1.631	1.52	C	-9.155	-1.686	1.609
C	-8.061	-2.452	1.42	C	-8.025	-2.501	1.484
C	4.996	4.897	0.091	C	4.959	4.912	-0.137
C	3.69	4.31	0.013	C	3.658	4.321	-0.187
C	2.601	5.161	0.264	C	2.564	5.176	0.089
C	2.845	6.511	0.664	C	2.818	6.525	0.492
C	4.134	7.078	0.81	C	4.112	7.087	0.626
C	5.218	6.21	0.453	C	5.186	6.224	0.232
C	1.744	7.293	1.002	C	1.725	7.303	0.861
C	1.775	8.583	1.492	C	1.769	8.589	1.381
C	3.044	9.168	1.611	C	3.037	9.165	1.492
C	4.21	8.435	1.29	C	4.2	8.432	1.135
C	1.241	4.677	0.182	C	1.217	4.693	0.036
C	0.138	5.507	0.519	C	0.119	5.523	0.409
C	0.425	6.795	0.956	C	0.415	6.803	0.845
C	0.978	3.339	-0.123	C	0.953	3.338	-0.248
C	-0.383	2.929	-0.315	C	-0.407	2.918	-0.383

C	-1.456	3.85	-0.205	C	-1.487	3.849	-0.256
C	-1.221	5.096	0.488	C	-1.236	5.1	0.438
C	-2.164	5.933	1.213	C	-2.147	5.911	1.209
C	-1.784	7.223	1.654	C	-1.763	7.219	1.646
C	-0.48	7.701	1.459	C	-0.483	7.704	1.406
C	0.299	9.013	1.799	C	0.301	9.017	1.729
C	-0.065	10.161	0.839	C	-0.089	10.164	0.778
C	0.037	9.373	3.265	C	0.075	9.378	3.201
C	-0.727	9.921	-0.368	C	-0.788	9.924	-0.408
C	-1.005	10.963	-1.262	C	-1.091	10.964	-1.295
C	-0.611	12.26	-0.923	C	-0.679	12.26	-0.973
C	0.06	12.529	0.277	C	0.03	12.529	0.205
C	0.33	11.469	1.146	C	0.321	11.471	1.069
C	0.917	9.007	4.283	C	0.974	9.001	4.199
C	0.616	9.266	5.628	C	0.706	9.263	5.551
C	-0.588	9.9	5.938	C	-0.485	9.907	5.887
C	-1.493	10.277	4.935	C	-1.409	10.293	4.906
C	-1.168	10.008	3.606	C	-1.116	10.023	3.569
C	5.524	9.102	1.491	C	5.518	9.083	1.348
C	-3.493	5.404	1.633	C	-3.45	5.368	1.68
C	-4.614	6.24	1.733	C	-4.576	6.191	1.827
C	-5.892	5.733	1.995	C	-5.838	5.666	2.134
C	-6.042	4.358	2.192	C	-5.963	4.288	2.329
C	-4.935	3.499	2.171	C	-4.846	3.443	2.264
C	-3.673	4.04	1.924	C	-3.602	4	1.972
C	6.539	8.499	2.248	C	6.539	8.44	2.065
C	7.766	9.136	2.458	C	7.771	9.061	2.29
C	7.969	10.401	1.896	C	7.973	10.35	1.786
C	6.974	11.035	1.141	C	6.973	11.024	1.074
C	5.757	10.376	0.949	C	5.751	10.381	0.865

C	5.795	-3.877	-2.09	C	5.836	-3.918	-2.06
C	6.02	-2.612	-1.444	C	6.035	-2.634	-1.452
C	7.356	-2.233	-1.235	C	7.361	-2.236	-1.224
C	8.398	-3.109	-1.672	C	8.421	-3.117	-1.607
C	8.186	-4.342	-2.337	C	8.234	-4.373	-2.235
C	6.81	-4.701	-2.52	C	6.868	-4.75	-2.436
C	9.708	-2.742	-1.389	C	9.721	-2.728	-1.309
C	10.845	-3.462	-1.699	C	10.871	-3.446	-1.571
C	10.65	-4.674	-2.37	C	10.702	-4.682	-2.206
C	9.346	-5.112	-2.7	C	9.411	-5.144	-2.548
C	7.672	-1.01	-0.534	C	7.654	-0.983	-0.56
C	9.033	-0.703	-0.211	C	9.003	-0.652	-0.224
C	10.017	-1.574	-0.665	C	10.005	-1.531	-0.624
C	6.656	-0.115	-0.14	C	6.621	-0.08	-0.225
C	7.072	0.982	0.701	C	7.013	1.053	0.58
C	8.381	1.25	1.02	C	8.314	1.347	0.912
C	9.448	0.417	0.551	C	9.397	0.504	0.498
C	10.857	0.582	0.785	C	10.799	0.694	0.747
C	11.799	-0.339	0.273	C	11.76	-0.238	0.292
C	11.377	-1.457	-0.455	C	11.36	-1.39	-0.395
C	12.076	-2.685	-1.126	C	12.083	-2.635	-1.007
C	13.034	-2.155	-2.203	C	13.052	-2.134	-2.087
C	12.785	-3.613	-0.128	C	12.787	-3.516	0.037
C	14.277	-1.636	-1.811	C	14.288	-1.596	-1.697
C	15.153	-1.077	-2.741	C	15.171	-1.059	-2.633
C	14.768	-1.041	-4.089	C	14.802	-1.066	-3.986
C	13.537	-1.549	-4.507	C	13.58	-1.594	-4.403
C	12.676	-2.106	-3.549	C	12.711	-2.128	-3.439
C	13.652	-4.603	-0.618	C	13.67	-4.515	-0.4
C	14.252	-5.526	0.237	C	14.269	-5.396	0.5

C	13.975	-5.449	1.611	C	13.973	-5.265	1.865
C	13.119	-4.475	2.125	C	13.101	-4.28	2.328
C	12.527	-3.56	1.241	C	12.51	-3.41	1.399
C	9.212	-6.404	-3.424	C	9.304	-6.456	-3.238
C	11.372	1.734	1.574	C	11.289	1.888	1.489
C	10.908	1.995	2.873	C	10.794	2.214	2.762
C	11.407	3.066	3.619	C	11.264	3.33	3.459
C	12.388	3.884	3.047	C	12.25	4.127	2.864
C	12.878	3.646	1.759	C	12.772	3.824	1.603
C	12.364	2.565	1.035	C	12.285	2.7	0.928
C	8.463	-6.501	-4.607	C	8.58	-6.593	-4.433
C	8.361	-7.708	-5.304	C	8.507	-7.818	-5.102
C	9.022	-8.833	-4.798	C	9.174	-8.921	-4.556
C	9.781	-8.77	-3.624	C	9.908	-8.819	-3.369
C	9.869	-7.549	-2.949	C	9.967	-7.58	-2.723
C	-6.049	9.047	-5.237	C	-6.279	9.116	-5.035
C	-4.462	9.45	-0.446	C	-4.499	9.421	-0.305
C	-7.976	-6.313	-1.086	C	-7.954	-6.328	-1.072
C	-10.208	-5.058	-5.453	C	-10.324	-5.047	-5.358
C	-9.55	-0.713	0.379	C	-9.548	-0.776	0.472
C	-10.476	-2.619	4.982	C	-10.344	-2.665	5.109
C	0.634	-12.906	3.507	C	0.719	-12.978	3.19
C	1.364	-11.919	-1.399	C	1.272	-11.931	-1.727
C	7.23	12.384	0.512	C	7.229	12.401	0.508
C	8.832	8.486	3.31	C	8.843	8.366	3.098
C	-7.085	6.658	1.97	C	-7.041	6.577	2.16
C	-5.105	2.006	2.333	C	-4.993	1.947	2.424
C	10.456	-10.003	-3.071	C	10.588	-10.029	-2.773
C	7.589	-7.787	-6.6	C	7.762	-7.939	-6.411
C	10.927	3.317	5.03	C	10.747	3.653	4.842

C	13.911	4.561	1.144	C	13.81	4.716	0.962
C	-7.705	-8.237	7.144	C	-7.467	-8.337	7.181
C	-2.668	-7.77	7.049	C	-2.439	-7.839	6.917
C	-6.65	-12.86	2.449	C	-6.594	-12.89	2.415
C	-9.497	-9.38	0.138	C	-9.458	-9.389	0.158
C	13.116	-1.488	-5.956	C	13.174	-1.578	-5.858
C	16.503	-0.547	-2.316	C	16.512	-0.508	-2.209
C	15.199	-6.577	-0.293	C	15.233	-6.458	0.026
C	12.825	-4.388	3.604	C	12.786	-4.136	3.799
C	1.578	8.839	6.712	C	1.688	8.826	6.612
C	-2.779	10.985	5.293	C	-2.682	11.009	5.293
C	-1.69	10.676	-2.578	C	-1.821	10.675	-2.586
C	0.45	13.941	0.644	C	0.437	13.941	0.554
C	-8.837	1.414	-7.977	C	-9.048	1.486	-7.86
C	-11.375	5.282	-5.928	C	-11.534	5.318	-5.684
C	-13.365	1.034	-1.361	C	-13.409	1.002	-1.148
C	-10.176	3.458	1.736	C	-10.129	3.323	1.931
H	1.668	-3.802	-0.389	H	1.658	-3.819	-0.57
H	5.457	2.309	-0.478	H	5.423	2.32	-0.697
H	3.387	-3.134	-1.334	H	3.4	-3.157	-1.392
H	-3.356	5.501	-1.177	H	-3.411	5.505	-1.164
H	-7.963	5.188	-3.004	H	-8.074	5.185	-2.842
H	-3.116	-2.77	-1.958	H	-3.144	-2.763	-2.037
H	-5.319	-3.388	-2.703	H	-5.361	-3.38	-2.742
H	-9.493	-0.784	-3.334	H	-9.564	-0.788	-3.228
H	-10.382	4.207	-3.629	H	-10.478	4.209	-3.429
H	-10.462	3.606	-7.875	H	-10.671	3.675	-7.68
H	-8.206	0.949	-5.377	H	-8.349	0.979	-5.285
H	-11.254	1.255	-3.068	H	-11.343	1.262	-2.904
H	-12.513	2.305	0.898	H	-12.493	2.208	1.12

H	-8.561	3.304	-0.428	H	-8.572	3.226	-0.28
H	-7.191	-3.731	-1.185	H	-7.184	-3.743	-1.172
H	-9.458	-6.639	-3.362	H	-9.502	-6.642	-3.305
H	-9.1	-2.636	-4.872	H	-9.212	-2.625	-4.789
H	-5.025	6.795	-0.452	H	-5.057	6.764	-0.345
H	-5.093	10.247	-2.993	H	-5.235	10.269	-2.806
H	-6.367	6.434	-4.5	H	-6.56	6.487	-4.344
H	-3.481	-0.914	1.076	H	-3.459	-0.937	1.026
H	-5.62	-1.566	1.968	H	-5.568	-1.598	1.981
H	-7.606	-5.954	3.018	H	-7.513	-5.999	3.065
H	1.269	-5.729	-0.213	H	1.247	-5.75	-0.465
H	0.797	-8.012	0.367	H	0.789	-8.04	0.103
H	-3.195	-10.231	2.243	H	-3.138	-10.268	2.106
H	-5.23	-10.65	3.177	H	-5.152	-10.689	3.129
H	-8.712	-11.848	0.985	H	-8.672	-11.863	0.985
H	-7.595	-7.709	1.15	H	-7.538	-7.73	1.153
H	-3.088	-7.858	4.377	H	-2.951	-7.9	4.26
H	-5.175	-8.01	8.119	H	-4.906	-8.108	8.07
H	-7.359	-8.271	4.436	H	-7.216	-8.333	4.462
H	-0.981	-10.716	3.311	H	-0.887	-10.775	3.083
H	1.625	-13.209	0.985	H	1.612	-13.254	0.629
H	-0.328	-9.853	-0.84	H	-0.385	-9.862	-1.078
H	-8.24	-4.074	4.4	H	-8.116	-4.111	4.474
H	-10.85	-1.08	2.769	H	-10.784	-1.136	2.899
H	-7.459	-2.405	0.519	H	-7.447	-2.456	0.567
H	5.863	4.287	-0.141	H	5.823	4.307	-0.392
H	6.235	6.584	0.474	H	6.203	6.599	0.234
H	3.162	10.176	1.995	H	3.167	10.161	1.903
H	-2.494	7.806	2.23	H	-2.463	7.793	2.243
H	-1.03	8.912	-0.624	H	-1.103	8.915	-0.652

H	-0.828	13.079	-1.605	H	-0.913	13.078	-1.652
H	0.836	11.665	2.087	H	0.856	11.668	1.994
H	1.849	8.51	4.035	H	1.895	8.494	3.93
H	-0.83	10.107	6.978	H	-0.703	10.115	6.933
H	-1.852	10.308	2.817	H	-1.814	10.33	2.795
H	-4.506	7.305	1.551	H	-4.488	7.258	1.65
H	-7.034	3.947	2.366	H	-6.943	3.864	2.539
H	-2.815	3.377	1.915	H	-2.736	3.348	1.929
H	6.358	7.526	2.698	H	6.358	7.449	2.472
H	8.921	10.905	2.05	H	8.929	10.842	1.953
H	4.979	10.845	0.353	H	4.969	10.881	0.3
H	4.777	-4.201	-2.273	H	4.825	-4.256	-2.255
H	6.559	-5.643	-2.994	H	6.635	-5.71	-2.884
H	11.491	-5.297	-2.658	H	11.555	-5.304	-2.459
H	6.312	1.607	1.151	H	6.24	1.688	0.991
H	8.6	2.099	1.658	H	8.513	2.223	1.517
H	12.85	-0.17	0.486	H	12.805	-0.049	0.514
H	14.568	-1.687	-0.765	H	14.566	-1.613	-0.647
H	15.444	-0.609	-4.824	H	15.485	-0.652	-4.725
H	11.717	-2.503	-3.865	H	11.758	-2.54	-3.754
H	13.866	-4.641	-1.682	H	13.899	-4.595	-1.459
H	14.439	-6.163	2.287	H	14.436	-5.946	2.577
H	11.854	-2.804	1.633	H	11.825	-2.645	1.751
H	10.163	1.337	3.311	H	10.047	1.572	3.219
H	12.78	4.723	3.619	H	12.62	5.001	3.398
H	12.722	2.372	0.028	H	12.667	2.458	-0.06
H	7.974	-5.613	-4.998	H	8.086	-5.722	-4.855
H	8.946	-9.779	-5.331	H	9.122	-9.88	-5.068
H	10.442	-7.483	-2.027	H	10.52	-7.485	-1.792
H	-5.114	9.313	-5.746	H	-5.366	9.391	-5.577

H	-6.63	9.97	-5.131	H	-6.852	10.037	-4.884
H	-6.603	8.373	-5.896	H	-6.863	8.457	-5.683
H	-5.184	9.482	0.379	H	-5.195	9.448	0.543
H	-3.53	9.036	-0.046	H	-3.56	8.991	0.06
H	-4.259	10.48	-0.751	H	-4.293	10.452	-0.601
H	-7.667	-7.274	-1.511	H	-7.673	-7.29	-1.513
H	-8.863	-6.502	-0.471	H	-8.816	-6.512	-0.421
H	-7.182	-5.969	-0.418	H	-7.13	-5.993	-0.437
H	-10.906	-5.868	-5.223	H	-11.02	-5.854	-5.11
H	-10.785	-4.207	-5.828	H	-10.908	-4.193	-5.716
H	-9.567	-5.403	-6.274	H	-9.708	-5.394	-6.197
H	-10.448	-0.128	0.587	H	-10.449	-0.202	0.695
H	-8.742	-0.007	0.158	H	-8.753	-0.061	0.236
H	-9.719	-1.282	-0.542	H	-9.726	-1.35	-0.443
H	-10.209	-1.794	5.655	H	-10.067	-1.835	5.77
H	-11.543	-2.518	4.756	H	-11.417	-2.575	4.91
H	-10.333	-3.551	5.536	H	-10.179	-3.593	5.663
H	-0.22	-12.923	4.192	H	-0.106	-12.993	3.909
H	0.873	-13.939	3.238	H	0.935	-14.01	2.898
H	1.49	-12.507	4.064	H	1.603	-12.595	3.716
H	2.363	-12.344	-1.263	H	2.271	-12.368	-1.634
H	1.453	-11.043	-2.049	H	1.346	-11.046	-2.367
H	0.764	-12.664	-1.936	H	0.645	-12.662	-2.252
H	6.295	12.909	0.295	H	6.295	12.94	0.327
H	7.834	13.022	1.166	H	7.844	13.003	1.184
H	7.776	12.281	-0.434	H	7.762	12.34	-0.449
H	9.832	8.833	3.034	H	9.842	8.724	2.829
H	8.812	7.396	3.213	H	8.82	7.283	2.946
H	8.685	8.721	4.372	H	8.708	8.548	4.171
H	-7.326	6.943	0.938	H	-7.316	6.877	1.141

H	-7.972	6.187	2.402	H	-7.91	6.088	2.61
H	-6.889	7.583	2.523	H	-6.839	7.494	2.724
H	-5.195	1.525	1.35	H	-5.14	1.474	1.445
H	-6.004	1.758	2.905	H	-5.853	1.687	3.049
H	-4.244	1.555	2.836	H	-4.099	1.5	2.869
H	11.374	-9.749	-2.532	H	11.491	-9.749	-2.221
H	10.712	-10.711	-3.865	H	10.871	-10.751	-3.545
H	9.8	-10.528	-2.365	H	9.924	-10.546	-2.069
H	7.123	-8.768	-6.73	H	7.309	-8.929	-6.523
H	6.801	-7.028	-6.645	H	6.968	-7.19	-6.491
H	8.248	-7.623	-7.462	H	8.437	-7.791	-7.263
H	9.958	2.843	5.215	H	9.776	3.187	5.027
H	10.825	4.388	5.233	H	10.638	4.733	4.984
H	11.635	2.915	5.765	H	11.437	3.293	5.615
H	14.575	4.985	1.904	H	14.442	5.2	1.713
H	14.528	4.033	0.41	H	14.458	4.152	0.284
H	13.433	5.4	0.624	H	13.336	5.51	0.373
H	-8.561	-8.417	6.488	H	-8.344	-8.515	6.552
H	-7.9	-7.314	7.703	H	-7.647	-7.422	7.757
H	-7.664	-9.052	7.876	H	-7.396	-9.161	7.9
H	-2.768	-7.964	8.12	H	-2.502	-8.042	7.989
H	-2.266	-6.757	6.93	H	-2.046	-6.822	6.793
H	-1.918	-8.461	6.648	H	-1.7	-8.522	6.485
H	-7.572	-13.377	2.734	H	-7.513	-13.405	2.712
H	-6.226	-13.402	1.595	H	-6.185	-13.431	1.553
H	-5.943	-12.946	3.278	H	-5.874	-12.982	3.233
H	-10.216	-10.204	0.101	H	-10.181	-10.209	0.128
H	-9.215	-9.138	-0.893	H	-9.188	-9.146	-0.876
H	-10.01	-8.502	0.543	H	-9.961	-8.509	0.572
H	13.961	-1.259	-6.612	H	14.024	-1.362	-6.511

H	12.679	-2.437	-6.283	H	12.748	-2.54	-6.162
H	12.355	-0.713	-6.112	H	12.41	-0.814	-6.045
H	16.722	0.414	-2.794	H	16.732	0.439	-2.714
H	17.306	-1.239	-2.6	H	17.323	-1.203	-2.461
H	16.556	-0.407	-1.233	H	16.552	-0.334	-1.13
H	14.959	-7.568	0.108	H	14.996	-7.434	0.464
H	16.235	-6.357	-0.006	H	16.262	-6.216	0.317
H	15.163	-6.636	-1.384	H	15.213	-6.562	-1.062
H	13.317	-5.19	4.161	H	13.281	-4.908	4.395
H	13.167	-3.433	4.02	H	13.111	-3.16	4.178
H	11.749	-4.454	3.798	H	11.708	-4.207	3.981
H	1.325	9.288	7.676	H	1.462	9.278	7.582
H	2.606	9.123	6.464	H	2.714	9.099	6.341
H	1.567	7.75	6.84	H	1.669	7.737	6.741
H	-3.281	10.498	6.136	H	-3.166	10.527	6.148
H	-2.59	12.024	5.588	H	-2.478	12.048	5.581
H	-3.475	11.003	4.45	H	-3.397	11.032	4.466
H	-2.403	9.85	-2.493	H	-2.548	9.866	-2.468
H	-2.23	11.552	-2.95	H	-2.353	11.558	-2.953
H	-0.959	10.394	-3.346	H	-1.119	10.368	-3.371
H	-0.354	14.44	1.199	H	-0.355	14.452	1.116
H	0.655	14.545	-0.245	H	0.634	14.536	-0.343
H	1.341	13.958	1.279	H	1.338	13.956	1.176
H	-9.53	1.542	-8.815	H	-9.762	1.628	-8.677
H	-7.848	1.746	-8.316	H	-8.068	1.823	-8.218
H	-8.76	0.344	-7.76	H	-8.967	0.412	-7.662
H	-12.097	5.16	-6.742	H	-12.284	5.202	-6.473
H	-10.769	6.166	-6.164	H	-10.937	6.205	-5.934
H	-11.93	5.502	-5.011	H	-12.056	5.529	-4.746
H	-14.165	1.513	-0.788	H	-14.193	1.472	-0.546

H	-13.338	-0.022	-1.063	H	-13.381	-0.06	-0.875
H	-13.637	1.067	-2.42	H	-13.707	1.06	-2.2
H	-9.631	2.736	2.358	H	-9.578	2.576	2.516
H	-9.542	4.345	1.639	H	-9.487	4.206	1.845
H	-11.081	3.742	2.282	H	-11.017	3.6	2.506

1d:

(*M*)-**1d** in the ground state				(*M*)-**1d** in the first excited state			
C	2.172	-1.008	0.028	C	2.174	-1.003	0.058
C	0.821	-1.415	0.288	C	0.826	-1.405	0.295
C	-0.21	-0.481	0.122	C	-0.213	-0.456	0.119
C	0.118	0.892	-0.16	C	0.121	0.915	-0.166
C	1.475	1.376	-0.264	C	1.485	1.392	-0.26
C	2.52	0.34	-0.344	C	2.529	0.347	-0.327
C	-0.984	1.803	-0.27	C	-0.976	1.828	-0.301
C	-0.735	3.144	-0.155	C	-0.717	3.178	-0.217
C	0.548	3.67	0.034	C	0.559	3.694	-0.011
C	1.7	2.83	-0.17	C	1.714	2.838	-0.182
C	-1.598	-0.894	0.289	C	-1.582	-0.862	0.28
C	-2.664	-0.007	0.005	C	-2.652	0.036	-0.008
C	-2.375	1.341	-0.351	C	-2.36	1.373	-0.367
C	-1.899	-2.215	0.696	C	-1.891	-2.19	0.691
C	-3.259	-2.596	0.98	C	-3.252	-2.56	0.978
C	-4.27	-1.679	0.712	C	-4.257	-1.631	0.721
C	-4.026	-0.438	0.099	C	-4.013	-0.394	0.096
C	-5.099	0.425	-0.364	C	-5.084	0.463	-0.364
C	-4.782	1.787	-0.641	C	-4.768	1.837	-0.639
C	-3.421	2.187	-0.654	C	-3.421	2.235	-0.657
C	2.899	-3.359	0.619	C	2.887	-3.353	0.666
C	3.174	-2.021	0.163	C	3.173	-2.014	0.218

C	4.477	-1.773	-0.259	C	4.479	-1.777	-0.208
C	5.479	-2.764	-0.061	C	5.472	-2.779	-0.019
C	5.284	-3.955	0.679	C	5.268	-3.977	0.717
C	3.925	-4.224	0.994	C	3.921	-4.234	1.039
C	6.697	-2.569	-0.684	C	6.685	-2.598	-0.653
C	7.786	-3.411	-0.629	C	7.77	-3.454	-0.606
C	7.65	-4.541	0.191	C	7.631	-4.584	0.212
C	6.43	-4.823	0.855	C	6.415	-4.858	0.886
C	4.752	-0.557	-0.957	C	4.757	-0.571	-0.92
C	5.983	-0.452	-1.677	C	5.985	-0.477	-1.644
C	6.916	-1.469	-1.532	C	6.911	-1.502	-1.499
C	3.849	0.526	-0.897	C	3.853	0.519	-0.873
C	4.407	1.762	-1.412	C	4.423	1.75	-1.392
C	5.455	1.77	-2.326	C	5.471	1.747	-2.303
C	6.323	0.646	-2.494	C	6.332	0.616	-2.467
C	7.602	0.62	-3.152	C	7.608	0.579	-3.125
C	8.503	-0.46	-2.958	C	8.503	-0.508	-2.931
C	8.162	-1.532	-2.127	C	8.156	-1.576	-2.1
C	8.858	-2.847	-1.629	C	8.843	-2.896	-1.606
C	4.241	2.999	-0.629	C	4.259	2.991	-0.615
C	2.994	3.512	-0.117	C	3.011	3.515	-0.12
C	3.071	4.822	0.433	C	3.091	4.828	0.419
C	4.346	5.434	0.667	C	4.367	5.434	0.662
C	5.582	4.839	0.342	C	5.603	4.828	0.354
C	5.46	3.616	-0.354	C	5.48	3.6	-0.333
C	4.396	6.703	1.234	C	4.418	6.707	1.217
C	5.535	7.425	1.532	C	5.559	7.426	1.519
C	6.764	6.822	1.234	C	6.788	6.813	1.238
C	6.805	5.539	0.648	C	6.827	5.525	0.664
C	1.886	5.587	0.751	C	1.905	5.606	0.712

C	1.971	6.914	1.285	C	1.994	6.937	1.235
C	3.233	7.434	1.529	C	3.257	7.45	1.491
C	0.628	5.044	0.498	C	0.647	5.073	0.444
C	-0.512	5.861	0.824	C	-0.49	5.903	0.745
C	-0.417	7.133	1.334	C	-0.392	7.179	1.246
C	0.858	7.742	1.569	C	0.884	7.778	1.495
C	1.116	9.061	2.084	C	1.146	9.1	2.002
C	2.436	9.512	2.315	C	2.466	9.544	2.245
C	3.529	8.689	2.022	C	3.557	8.708	1.973
C	5.087	8.816	2.093	C	5.114	8.826	2.059
C	-4.866	-4.425	1.78	C	-4.868	-4.383	1.754
C	-3.558	-3.951	1.429	C	-3.559	-3.911	1.42
C	-2.519	-4.892	1.427	C	-2.516	-4.864	1.429
C	-2.83	-6.269	1.663	C	-2.84	-6.24	1.652
C	-4.134	-6.759	1.918	C	-4.153	-6.724	1.874
C	-5.148	-5.751	2.027	C	-5.161	-5.712	1.983
C	-1.804	-7.196	1.516	C	-1.817	-7.173	1.517
C	-1.925	-8.571	1.583	C	-1.953	-8.555	1.56
C	-3.208	-9.067	1.854	C	-3.24	-9.042	1.79
C	-4.302	-8.185	2.013	C	-4.335	-8.15	1.936
C	-1.165	-4.504	1.096	C	-1.169	-4.484	1.118
C	-0.152	-5.484	0.924	C	-0.156	-5.472	0.959
C	-0.511	-6.815	1.102	C	-0.523	-6.797	1.127
C	-0.852	-3.172	0.792	C	-0.852	-3.143	0.797
C	0.516	-2.806	0.56	C	0.513	-2.783	0.563
C	1.527	-3.798	0.563	C	1.53	-3.786	0.593
C	1.162	-5.195	0.48	C	1.158	-5.19	0.512
C	1.922	-6.315	-0.057	C	1.904	-6.3	-0.035
C	1.48	-7.64	0.146	C	1.457	-7.641	0.166
C	0.262	-7.909	0.786	C	0.248	-7.906	0.797

C	-0.542	-9.19	1.184	C	-0.567	-9.183	1.184
C	-5.616	4.165	-1.092	C	-5.632	4.209	-1.075
C	-5.833	2.761	-0.871	C	-5.835	2.805	-0.862
C	-7.168	2.326	-0.834	C	-7.164	2.355	-0.829
C	-8.216	3.289	-0.982	C	-8.222	3.308	-0.97
C	-8.009	4.679	-1.155	C	-8.029	4.702	-1.136
C	-6.634	5.08	-1.228	C	-6.66	5.116	-1.208
C	-9.525	2.822	-0.992	C	-9.527	2.829	-0.978
C	-10.665	3.584	-1.15	C	-10.676	3.581	-1.124
C	-10.475	4.962	-1.303	C	-10.499	4.963	-1.272
C	-9.172	5.515	-1.294	C	-9.203	5.528	-1.267
C	-7.479	0.921	-0.707	C	-7.465	0.946	-0.707
C	-8.84	0.483	-0.764	C	-8.82	0.496	-0.756
C	-9.829	1.451	-0.896	C	-9.82	1.455	-0.881
C	-6.458	-0.039	-0.555	C	-6.435	-0.007	-0.559
C	-6.868	-1.417	-0.69	C	-6.834	-1.389	-0.696
C	-8.179	-1.821	-0.756	C	-8.141	-1.804	-0.752
C	-9.25	-0.871	-0.722	C	-9.22	-0.862	-0.708
C	-10.66	-1.148	-0.751	C	-10.627	-1.151	-0.719
C	-11.608	-0.108	-0.893	C	-11.586	-0.119	-0.856
C	-11.189	1.225	-0.99	C	-11.178	1.217	-0.962
C	-11.893	2.613	-1.158	C	-11.895	2.6	-1.123
C	-12.082	-5.164	-0.295	C	-11.977	-5.186	-0.215
C	-12.582	-4.376	-1.341	C	-12.505	-4.415	-1.259
C	-12.108	-3.07	-1.483	C	-12.058	-3.101	-1.416
C	-11.145	-2.545	-0.606	C	-11.093	-2.552	-0.556
C	-10.671	-3.359	0.433	C	-10.592	-3.35	0.484
C	-11.128	-4.671	0.6	C	-11.022	-4.669	0.666
C	-10.594	-5.521	1.729	C	-10.458	-5.501	1.795
C	-13.589	-4.945	-2.313	C	-13.514	-5.009	-2.214

C	-8.163	-9.961	2.56	C	-8.229	-9.895	2.308
C	-7.978	-9.038	1.521	C	-8.004	-8.936	1.311
C	-6.72	-8.453	1.363	C	-6.735	-8.362	1.211
C	-5.65	-8.781	2.215	C	-5.693	-8.737	2.078
C	-5.867	-9.717	3.233	C	-5.948	-9.711	3.052
C	-7.119	-10.315	3.418	C	-7.212	-10.297	3.18
C	-7.324	-11.335	4.514	C	-7.462	-11.355	4.229
C	-9.108	-8.72	0.57	C	-9.1	-8.569	0.338
C	-1.302	-12.037	-1.976	C	-1.294	-12.035	-1.979
C	-0.937	-10.734	-2.315	C	-0.931	-10.73	-2.317
C	-0.667	-9.82	-1.286	C	-0.672	-9.815	-1.286
C	-0.754	-10.198	0.053	C	-0.766	-10.194	0.053
C	-1.125	-11.515	0.365	C	-1.136	-11.511	0.363
C	-1.405	-12.443	-0.638	C	-1.406	-12.44	-0.642
C	-1.822	-13.855	-0.299	C	-1.821	-13.853	-0.305
C	-0.846	-10.294	-3.758	C	-0.832	-10.291	-3.759
C	1.894	-10.61	4.465	C	1.8	-10.607	4.514
C	2.306	-10.826	3.144	C	2.237	-10.825	3.201
C	1.464	-10.417	2.108	C	1.417	-10.414	2.149
C	0.243	-9.783	2.369	C	0.193	-9.776	2.385
C	-0.149	-9.59	3.695	C	-0.224	-9.58	3.703
C	0.667	-10.004	4.756	C	0.569	-9.997	4.78
C	0.217	-9.817	6.185	C	0.091	-9.806	6.2
C	3.646	-11.449	2.828	C	3.581	-11.453	2.913
C	5.216	-5.673	-2.743	C	5.199	-5.664	-2.716
C	5.229	-6.794	-1.908	C	5.208	-6.787	-1.884
C	4.169	-6.985	-1.015	C	4.145	-6.979	-0.994
C	3.068	-6.112	-0.994	C	3.048	-6.101	-0.971
C	3.053	-5.045	-1.909	C	3.036	-5.031	-1.883
C	4.135	-4.786	-2.751	C	4.12	-4.774	-2.723

C	4.166	-3.548	-3.615	C	4.156	-3.534	-3.585
C	6.377	-7.771	-1.999	C	6.354	-7.766	-1.974
C	6.014	-8.449	3.115	C	6.005	-8.481	3.152
C	6.852	-8.426	1.995	C	6.843	-8.457	2.032
C	6.99	-7.231	1.285	C	6.979	-7.263	1.32
C	6.32	-6.058	1.671	C	6.305	-6.09	1.702
C	5.525	-6.11	2.832	C	5.507	-6.144	2.862
C	5.344	-7.298	3.545	C	5.331	-7.33	3.578
C	4.426	-7.36	4.744	C	4.413	-7.392	4.777
C	7.596	-9.669	1.565	C	7.589	-9.699	1.603
C	9.008	-6.032	-4.553	C	8.974	-6.073	-4.54
C	9.731	-6.15	-3.358	C	9.694	-6.2	-3.343
C	9.717	-5.082	-2.459	C	9.685	-5.136	-2.441
C	8.985	-3.916	-2.726	C	8.962	-3.963	-2.705
C	8.28	-3.822	-3.927	C	8.262	-3.861	-3.908
C	8.281	-4.877	-4.851	C	8.257	-4.911	-4.836
C	7.463	-4.777	-6.117	C	7.443	-4.8	-6.103
C	10.473	-7.425	-3.033	C	10.426	-7.482	-3.021
C	12.564	-1.465	0.102	C	12.558	-1.539	0.125
C	12.515	-1.774	-1.262	C	12.51	-1.854	-1.238
C	11.32	-2.266	-1.796	C	11.312	-2.338	-1.772
C	10.191	-2.451	-0.99	C	10.179	-2.51	-0.967
C	10.269	-2.142	0.37	C	10.257	-2.194	0.392
C	11.451	-1.643	0.931	C	11.442	-1.703	0.952
C	11.504	-1.263	2.391	C	11.495	-1.316	2.411
C	13.737	-1.611	-2.135	C	13.735	-1.707	-2.109
C	8.888	3.938	-5.53	C	8.921	3.889	-5.498
C	9.76	3.393	-4.581	C	9.787	3.339	-4.547
C	9.321	2.315	-3.808	C	9.341	2.262	-3.777
C	8.041	1.768	-3.982	C	8.057	1.723	-3.954

C	7.193	2.333	-4.948	C	7.215	2.294	-4.922
C	7.601	3.422	-5.723	C	7.632	3.382	-5.695
C	6.66	4.048	-6.726	C	6.698	4.014	-6.7
C	11.156	3.94	-4.403	C	11.186	3.877	-4.364
C	10.557	3.83	-0.535	C	10.576	3.791	-0.496
C	9.612	3.014	0.1	C	9.625	2.983	0.14
C	8.416	3.593	0.53	C	8.43	3.571	0.565
C	8.118	4.945	0.28	C	8.14	4.923	0.307
C	9.087	5.728	-0.365	C	9.115	5.698	-0.339
C	10.314	5.188	-0.763	C	10.341	5.15	-0.731
C	11.334	6.063	-1.456	C	11.366	6.015	-1.425
C	9.853	1.533	0.277	C	9.857	1.501	0.323
C	6.013	9.521	6.261	C	6.003	9.577	6.227
C	5.58	10.584	5.453	C	5.589	10.634	5.4
C	5.318	10.335	4.107	C	5.338	10.369	4.055
C	5.474	9.051	3.56	C	5.488	9.077	3.526
C	5.904	8.014	4.385	C	5.9	8.047	4.37
C	6.18	8.237	5.744	C	6.164	8.285	5.728
C	6.661	7.097	6.61	C	6.621	7.151	6.615
C	5.409	11.965	6.04	C	5.425	12.024	5.967
C	6.827	11.715	-0.61	C	6.898	11.68	-0.662
C	7.596	11.193	0.439	C	7.654	11.166	0.4
C	6.999	10.288	1.319	C	7.044	10.276	1.285
C	5.663	9.894	1.161	C	5.706	9.888	1.12
C	4.923	10.425	0.105	C	4.979	10.412	0.05
C	5.493	11.343	-0.788	C	5.563	11.315	-0.848
C	4.66	11.936	-1.9	C	4.744	11.899	-1.976
C	9.047	11.581	0.596	C	9.106	11.547	0.565
C	-2.085	11.769	2.972	C	-2.046	11.839	2.829
C	-1.092	11.54	3.93	C	-1.065	11.612	3.8

C	-0.061	10.646	3.626	C	-0.036	10.708	3.515
C	-0.003	9.992	2.387	C	0.03	10.043	2.283
C	-1.007	10.259	1.441	C	-0.962	10.307	1.324
C	-2.056	11.136	1.723	C	-2.009	11.194	1.587
C	-3.155	11.378	0.714	C	-3.096	11.433	0.565
C	-1.119	12.258	5.26	C	-1.1	12.343	5.122
C	-8.827	9.766	-1.713	C	-8.898	9.784	-1.665
C	-8.137	9.126	-0.676	C	-8.194	9.145	-0.637
C	-8.256	7.74	-0.549	C	-8.3	7.757	-0.516
C	-9.035	6.988	-1.444	C	-9.08	7.003	-1.409
C	-9.714	7.661	-2.47	C	-9.773	7.676	-2.425
C	-9.619	9.049	-2.616	C	-9.692	9.065	-2.565
C	-10.329	9.75	-3.751	C	-10.417	9.766	-3.689
C	-7.267	9.918	0.272	C	-7.322	9.938	0.308
C	-13.795	3.237	-4.996	C	-13.834	3.209	-4.945
C	-12.935	2.149	-4.84	C	-12.967	2.126	-4.795
C	-12.342	1.932	-3.588	C	-12.361	1.914	-3.548
C	-12.604	2.778	-2.51	C	-12.619	2.759	-2.469
C	-13.475	3.862	-2.696	C	-13.498	3.838	-2.648
C	-14.077	4.104	-3.931	C	-14.112	4.075	-3.877
C	-15.034	5.258	-4.115	C	-15.078	5.223	-4.054
C	-12.654	1.202	-5.983	C	-12.69	1.181	-5.94
C	-14.58	3.01	2.211	C	-14.558	2.974	2.268
C	-14.974	2.265	1.089	C	-14.955	2.226	1.148
C	-14.1	2.176	0.007	C	-14.09	2.145	0.059
C	-12.849	2.812	0.027	C	-12.843	2.79	0.069
C	-12.482	3.543	1.155	C	-12.473	3.524	1.195
C	-13.343	3.652	2.258	C	-13.326	3.625	2.306
C	-12.92	4.457	3.465	C	-12.899	4.431	3.51
C	-16.324	1.588	1.06	C	-16.301	1.54	1.129

H	-1.573	3.818	-0.112	H	-1.553	3.857	-0.194
H	-5.284	-1.935	0.971	H	-5.271	-1.873	0.995
H	-3.199	3.204	-0.943	H	-3.198	3.253	-0.941
H	3.655	-5.178	1.42	H	3.64	-5.182	1.473
H	8.483	-5.221	0.333	H	8.461	-5.268	0.348
H	5.766	2.726	-2.731	H	5.791	2.7	-2.708
H	9.462	-0.426	-3.464	H	9.462	-0.482	-3.44
H	6.358	3.127	-0.695	H	6.377	3.103	-0.664
H	7.701	7.328	1.447	H	7.725	7.317	1.453
H	-1.512	5.463	0.702	H	-1.491	5.511	0.612
H	-1.325	7.671	1.58	H	-1.299	7.725	1.475
H	2.578	10.52	2.693	H	2.611	10.554	2.613
H	-5.681	-3.714	1.858	H	-5.683	-3.67	1.824
H	-6.16	-6.035	2.294	H	-6.179	-5.992	2.232
H	-3.401	-10.135	1.878	H	-3.445	-10.109	1.785
H	2.056	-8.445	-0.297	H	2.041	-8.443	-0.272
H	-4.601	4.54	-1.155	H	-4.62	4.593	-1.132
H	-6.387	6.12	-1.408	H	-6.422	6.158	-1.382
H	-11.319	5.636	-1.416	H	-11.35	5.628	-1.376
H	-6.105	-2.176	-0.806	H	-6.063	-2.139	-0.817
H	-8.401	-2.875	-0.881	H	-8.355	-2.859	-0.878
H	-12.662	-0.367	-0.897	H	-12.638	-0.388	-0.846
H	-12.449	-6.182	-0.174	H	-12.323	-6.209	-0.083
H	-12.476	-2.45	-2.295	H	-12.447	-2.493	-2.228
H	-9.949	-2.952	1.135	H	-9.868	-2.924	1.173
H	-9.555	-5.82	1.548	H	-9.421	-5.797	1.595
H	-10.609	-4.974	2.678	H	-10.457	-4.942	2.736
H	-11.18	-6.435	1.858	H	-11.034	-6.417	1.947
H	-14.132	-4.154	-2.838	H	-14.077	-4.232	-2.739
H	-13.098	-5.566	-3.072	H	-13.02	-5.628	-2.974

H	-14.323	-5.578	-1.803	H	-14.229	-5.651	-1.69
H	-9.142	-10.415	2.698	H	-9.217	-10.341	2.403
H	-6.554	-7.749	0.554	H	-6.538	-7.63	0.434
H	-5.047	-9.967	3.902	H	-5.15	-9.998	3.732
H	-6.797	-11.049	5.43	H	-6.934	-11.127	5.161
H	-8.384	-11.456	4.758	H	-8.527	-11.452	4.458
H	-6.941	-12.318	4.214	H	-7.109	-12.337	3.889
H	-8.933	-7.785	0.031	H	-8.936	-7.585	-0.109
H	-9.225	-9.513	-0.178	H	-9.154	-9.297	-0.482
H	-10.062	-8.63	1.099	H	-10.08	-8.557	0.825
H	-1.506	-12.757	-2.767	H	-1.491	-12.755	-2.771
H	-0.384	-8.803	-1.54	H	-0.391	-8.798	-1.539
H	-1.18	-11.818	1.407	H	-1.197	-11.814	1.405
H	-2.878	-14.023	-0.541	H	-2.874	-14.026	-0.557
H	-1.241	-14.59	-0.867	H	-1.232	-14.586	-0.867
H	-1.688	-14.069	0.765	H	-1.696	-14.066	0.761
H	-0.851	-11.148	-4.441	H	-0.826	-11.145	-4.441
H	-1.693	-9.65	-4.025	H	-1.68	-9.652	-4.033
H	0.066	-9.716	-3.941	H	0.079	-9.708	-3.935
H	2.541	-10.923	5.282	H	2.429	-10.921	5.344
H	1.769	-10.577	1.078	H	1.743	-10.575	1.124
H	-1.093	-9.097	3.908	H	-1.17	-9.083	3.897
H	-0.4	-8.92	6.296	H	-0.506	-8.894	6.301
H	-0.385	-10.67	6.524	H	-0.54	-10.645	6.519
H	1.069	-9.729	6.866	H	0.929	-9.742	6.9
H	4.132	-11.837	3.728	H	4.052	-11.834	3.823
H	3.544	-12.275	2.116	H	3.488	-12.285	2.206
H	4.318	-10.71	2.377	H	4.262	-10.72	2.466
H	6.069	-5.486	-3.39	H	6.052	-5.479	-3.363
H	4.22	-7.803	-0.302	H	4.194	-7.799	-0.283

H	2.199	-4.375	-1.929	H	2.184	-4.36	-1.902
H	4.648	-3.74	-4.579	H	4.646	-3.723	-4.545
H	3.16	-3.162	-3.805	H	3.151	-3.149	-3.781
H	4.734	-2.751	-3.121	H	4.719	-2.738	-3.083
H	7.339	-7.249	-1.959	H	7.317	-7.246	-1.93
H	6.343	-8.511	-1.196	H	6.318	-8.507	-1.173
H	6.349	-8.31	-2.954	H	6.328	-8.303	-2.931
H	5.883	-9.378	3.666	H	5.876	-9.408	3.705
H	7.602	-7.222	0.391	H	7.592	-7.255	0.427
H	5.039	-5.207	3.185	H	5.019	-5.242	3.214
H	3.57	-8.016	4.543	H	3.554	-8.044	4.575
H	4.943	-7.765	5.621	H	4.929	-7.801	5.653
H	4.038	-6.373	5.007	H	4.028	-6.404	5.042
H	6.994	-10.57	1.723	H	6.988	-10.601	1.76
H	7.871	-9.627	0.507	H	7.867	-9.657	0.546
H	8.522	-9.793	2.139	H	8.515	-9.822	2.18
H	9.013	-6.857	-5.262	H	8.975	-6.896	-5.252
H	10.28	-5.153	-1.532	H	10.246	-5.214	-1.513
H	7.71	-2.926	-4.147	H	7.698	-2.959	-4.126
H	7.424	-3.748	-6.488	H	7.422	-3.772	-6.477
H	6.429	-5.097	-5.936	H	6.404	-5.103	-5.924
H	7.867	-5.412	-6.911	H	7.838	-5.444	-6.895
H	10.92	-7.869	-3.927	H	10.871	-7.927	-3.916
H	11.271	-7.251	-2.304	H	11.223	-7.317	-2.29
H	9.793	-8.172	-2.604	H	9.739	-8.225	-2.596
H	13.488	-1.08	0.528	H	13.485	-1.16	0.552
H	11.267	-2.513	-2.853	H	11.258	-2.59	-2.827
H	9.395	-2.278	0.999	H	9.38	-2.318	1.019
H	11.213	-0.214	2.529	H	11.213	-0.265	2.544
H	10.819	-1.872	2.989	H	10.804	-1.917	3.01

H	12.512	-1.379	2.802	H	12.501	-1.439	2.824
H	14.377	-0.797	-1.783	H	14.382	-0.897	-1.758
H	14.345	-2.525	-2.137	H	14.334	-2.626	-2.106
H	13.462	-1.403	-3.174	H	13.464	-1.499	-3.149
H	9.216	4.784	-6.13	H	9.257	4.734	-6.096
H	9.97	1.91	-3.037	H	9.985	1.852	-3.005
H	6.205	1.906	-5.098	H	6.225	1.875	-5.074
H	6.082	4.859	-6.267	H	6.123	4.828	-6.242
H	7.204	4.475	-7.574	H	7.247	4.439	-7.546
H	5.945	3.317	-7.115	H	5.979	3.289	-7.091
H	11.878	3.381	-5.012	H	11.908	3.31	-4.965
H	11.219	4.99	-4.704	H	11.258	4.925	-4.67
H	11.477	3.865	-3.361	H	11.5	3.806	-3.319
H	11.503	3.396	-0.854	H	11.521	3.351	-0.81
H	7.698	2.981	1.068	H	7.708	2.965	1.104
H	8.867	6.77	-0.582	H	8.901	6.74	-0.562
H	12.304	5.565	-1.536	H	12.335	5.513	-1.498
H	11.009	6.317	-2.472	H	11.047	6.265	-2.443
H	11.48	7.005	-0.918	H	11.515	6.96	-0.892
H	9.434	1.166	1.219	H	9.428	1.138	1.263
H	9.374	0.962	-0.528	H	9.382	0.93	-0.485
H	10.917	1.28	0.257	H	10.92	1.244	0.313
H	6.225	9.704	7.312	H	6.206	9.773	7.278
H	4.998	11.149	3.464	H	5.031	11.178	3.398
H	6.026	7.016	3.976	H	6.017	7.043	3.975
H	6	6.227	6.523	H	5.927	6.305	6.564
H	7.663	6.768	6.309	H	7.603	6.778	6.303
H	6.707	7.383	7.664	H	6.698	7.462	7.66
H	6.353	12.343	6.448	H	6.372	12.404	6.368
H	5.059	12.68	5.29	H	5.078	12.729	5.207

H	4.684	11.959	6.863	H	4.701	12.034	6.79
H	7.279	12.427	-1.297	H	7.361	12.382	-1.354
H	7.577	9.888	2.148	H	7.611	9.882	2.123
H	3.888	10.125	-0.031	H	3.944	10.117	-0.092
H	4.001	11.186	-2.348	H	4.098	11.142	-2.431
H	4.019	12.744	-1.524	H	4.093	12.704	-1.614
H	5.286	12.356	-2.693	H	5.38	12.319	-2.761
H	9.208	12.636	0.352	H	9.276	12.598	0.312
H	9.4	11.411	1.617	H	9.449	11.385	1.591
H	9.686	10.993	-0.075	H	9.748	10.948	-0.094
H	-2.897	12.456	3.201	H	-2.856	12.532	3.044
H	0.704	10.435	4.369	H	0.72	10.499	4.268
H	-0.955	9.782	0.466	H	-0.903	9.82	0.354
H	-2.827	11.14	-0.302	H	-2.762	11.173	-0.444
H	-4.032	10.754	0.931	H	-3.982	10.824	0.784
H	-3.491	12.42	0.727	H	-3.418	12.479	0.558
H	-0.54	13.188	5.216	H	-0.519	13.272	5.074
H	-2.14	12.522	5.553	H	-2.122	12.612	5.405
H	-0.686	11.643	6.055	H	-0.675	11.735	5.926
H	-8.748	10.846	-1.817	H	-8.829	10.866	-1.763
H	-7.755	7.233	0.271	H	-7.788	7.25	0.297
H	-10.313	7.087	-3.172	H	-10.373	7.1	-3.126
H	-11.275	9.257	-3.996	H	-11.357	9.263	-3.935
H	-10.543	10.795	-3.506	H	-10.645	10.806	-3.433
H	-9.716	9.746	-4.66	H	-9.808	9.78	-4.602
H	-7.696	10.903	0.484	H	-7.75	10.924	0.518
H	-7.131	9.396	1.224	H	-7.185	9.418	1.261
H	-6.27	10.085	-0.156	H	-6.325	10.102	-0.121
H	-14.257	3.417	-5.965	H	-14.306	3.386	-5.91
H	-11.664	1.094	-3.46	H	-11.677	1.08	-3.426

H	-13.69	4.52	-1.858	H	-13.71	4.495	-1.808
H	-14.915	6.008	-3.328	H	-14.962	5.971	-3.264
H	-14.883	5.752	-5.08	H	-14.932	5.723	-5.018
H	-16.075	4.915	-4.087	H	-16.117	4.873	-4.026
H	-12.952	1.631	-6.944	H	-12.993	1.61	-6.9
H	-11.59	0.951	-6.039	H	-11.626	0.93	-6.001
H	-13.201	0.26	-5.858	H	-13.236	0.238	-5.814
H	-15.255	3.089	3.06	H	-15.227	3.047	3.123
H	-14.397	1.617	-0.876	H	-14.39	1.584	-0.822
H	-11.516	4.037	1.184	H	-11.511	4.025	1.217
H	-12.732	5.503	3.197	H	-12.71	5.476	3.24
H	-13.684	4.443	4.247	H	-13.66	4.418	4.294
H	-11.991	4.065	3.895	H	-11.969	4.039	3.938
H	-17.137	2.325	1.072	H	-17.119	2.271	1.141
H	-16.446	0.972	0.165	H	-16.423	0.92	0.237
H	-16.464	0.943	1.935	H	-16.432	0.899	2.007

9 and **10** in the first excited state:

(*M*, *M*)-**9** in the first excited state				(*M*, *M*)-**10** in the first excited state			
C	-2.851	2.459	-0.007	C	2.851	2.463	-0.036
C	-1.465	2.433	0.129	C	1.462	2.43	-0.169
C	-0.722	1.241	0.016	C	0.722	1.243	-0.027
C	-1.434	0	0	C	1.435	0	0
C	-2.863	0	0	C	2.858	0	0
C	-3.542	1.239	-0.282	C	3.542	1.244	0.266
C	-0.722	-1.241	-0.016	C	0.722	-1.243	0.027
C	-1.465	-2.433	-0.129	C	1.462	-2.43	0.169
C	-2.851	-2.459	0.007	C	2.851	-2.463	0.036
C	-3.542	-1.239	0.282	C	3.542	-1.244	-0.266
C	0.722	1.241	-0.016	C	-0.722	1.243	0.027

C	1.434	0	0	C	-1.435	0	0
C	0.722	-1.241	0.016	C	-0.722	-1.243	-0.027
C	1.465	2.433	-0.129	C	-1.462	2.43	0.169
C	2.851	2.459	0.007	C	-2.851	2.463	0.036
C	3.542	1.239	0.282	C	-3.542	1.244	-0.266
C	2.863	0	0	C	-2.858	0	0
C	3.542	-1.239	-0.282	C	-3.542	-1.244	0.266
C	2.851	-2.459	-0.007	C	-2.851	-2.463	-0.036
C	1.465	-2.433	0.129	C	-1.462	-2.43	-0.169
C	-3.071	4.848	0.732	C	3.04	4.875	-0.722
C	-3.626	3.696	0.137	C	3.618	3.695	-0.173
C	-4.988	3.722	-0.264	C	4.978	3.715	0.202
C	-5.74	4.891	-0.025	C	5.752	4.912	0.052
C	-5.172	6.013	0.559	C	5.143	6.076	-0.512
C	-3.824	5.994	0.935	C	3.773	6.017	-0.893
C	-5.557	2.552	-0.938	C	7.12	4.962	0.463
C	-6.788	2.619	-1.62	C	7.828	6.152	0.269
C	-4.838	1.321	-0.938	C	7.23	7.283	-0.302
C	-5.371	0.234	-1.674	C	5.904	7.253	-0.684
C	-6.582	0.326	-2.331	C	5.581	2.541	0.781
C	-7.308	1.526	-2.294	C	6.931	2.599	1.254
C	-5.371	-0.234	1.674	C	7.722	3.779	1.089
C	-4.838	-1.321	0.938	C	4.846	1.331	0.865
C	-5.557	-2.552	0.938	C	5.419	0.24	1.597
C	-6.788	-2.619	1.62	C	6.687	0.297	2.085
C	-7.308	-1.526	2.294	C	7.505	1.453	1.889
C	-6.582	-0.326	2.331	C	8.838	1.491	2.33
C	-4.988	-3.722	0.264	C	9.602	2.633	2.149
C	-5.74	-4.891	0.025	C	9.048	3.761	1.538
C	-3.626	-3.696	-0.137	C	5.419	-0.24	-1.597

C	-3.071	-4.848	-0.732	C	4.846	-1.331	-0.865
C	-3.824	-5.994	-0.935	C	5.581	-2.541	-0.781
C	-5.172	-6.013	-0.559	C	6.931	-2.599	-1.254
C	3.071	4.848	-0.732	C	7.505	-1.453	-1.889
C	3.626	3.696	-0.137	C	6.687	-0.297	-2.085
C	4.988	3.722	0.264	C	7.722	-3.779	-1.089
C	5.74	4.891	0.025	C	9.048	-3.761	-1.538
C	5.172	6.013	-0.559	C	9.602	-2.633	-2.149
C	3.824	5.993	-0.935	C	8.838	-1.491	-2.33
C	5.557	2.552	0.938	C	4.979	-3.715	-0.202
C	6.788	2.619	1.62	C	5.752	-4.912	-0.052
C	4.838	1.321	0.938	C	7.12	-4.962	-0.463
C	5.371	0.234	1.674	C	3.618	-3.695	0.173
C	6.582	0.326	2.331	C	3.04	-4.875	0.722
C	7.308	1.526	2.294	C	3.773	-6.017	0.893
C	3.071	-4.848	0.732	C	5.143	-6.076	0.512
C	3.626	-3.696	0.137	C	5.904	-7.253	0.684
C	4.988	-3.722	-0.264	C	7.23	-7.283	0.302
C	5.74	-4.891	-0.025	C	7.829	-6.152	-0.269
C	5.172	-6.013	0.558	C	-3.04	4.875	0.722
C	3.824	-5.994	0.935	C	-3.618	3.695	0.173
C	5.557	-2.552	-0.938	C	-4.979	3.715	-0.202
C	6.788	-2.619	-1.62	C	-5.752	4.912	-0.052
C	4.838	-1.321	-0.938	C	-5.143	6.076	0.513
C	5.371	-0.234	-1.674	C	-3.773	6.017	0.893
C	6.582	-0.326	-2.331	C	-7.12	4.962	-0.463
C	7.308	-1.526	-2.294	C	-7.829	6.152	-0.269
H	-0.938	3.362	0.293	C	-7.23	7.283	0.302
H	-0.938	-3.362	-0.293	C	-5.904	7.253	0.684
H	0.938	3.362	-0.293	C	-5.581	2.541	-0.781

H	0.938	-3.362	0.293	C	-6.931	2.599	-1.254
H	-2.043	4.833	1.079	C	-7.722	3.779	-1.089
H	-6.792	4.915	-0.284	C	-4.846	1.331	-0.865
H	-5.778	6.897	0.734	C	-5.419	0.24	-1.597
H	-3.373	6.862	1.406	C	-6.687	0.297	-2.085
H	-7.334	3.555	-1.65	C	-7.505	1.453	-1.889
H	-4.803	-0.685	-1.741	C	-8.838	1.491	-2.33
H	-6.958	-0.525	-2.89	C	-9.602	2.633	-2.149
H	-8.257	1.61	-2.814	C	-9.048	3.761	-1.538
H	-4.803	0.685	1.741	C	-3.04	-4.875	-0.722
H	-7.334	-3.555	1.65	C	-3.618	-3.695	-0.173
H	-8.257	-1.61	2.814	C	-4.978	-3.715	0.202
H	-6.958	0.526	2.89	C	-5.752	-4.912	0.052
H	-6.792	-4.915	0.284	C	-5.143	-6.076	-0.512
H	-2.043	-4.833	-1.079	C	-3.773	-6.017	-0.893
H	-3.373	-6.862	-1.406	C	-7.12	-4.962	0.463
H	-5.778	-6.897	-0.734	C	-7.828	-6.152	0.269
H	2.043	4.832	-1.079	C	-7.23	-7.283	-0.301
H	6.792	4.915	0.284	C	-5.904	-7.253	-0.684
H	5.778	6.897	-0.734	C	-5.581	-2.541	0.781
H	3.373	6.862	-1.406	C	-6.931	-2.599	1.254
H	7.334	3.555	1.65	C	-7.722	-3.779	1.089
H	4.803	-0.685	1.741	C	-4.846	-1.331	0.865
H	6.958	-0.525	2.89	C	-5.419	-0.24	1.597
H	8.257	1.61	2.814	C	-6.687	-0.297	2.085
H	2.043	-4.833	1.078	C	-7.505	-1.453	1.889
H	6.792	-4.915	-0.284	C	-8.838	-1.491	2.33
H	5.778	-6.897	0.733	C	-9.602	-2.633	2.149
H	3.373	-6.862	1.406	C	-9.048	-3.761	1.538
H	7.334	-3.555	-1.65	H	0.93	3.352	-0.355

H	4.803	0.685	-1.741
H	6.958	0.526	-2.89
H	8.257	-1.61	-2.814

H	0.93	-3.352	0.355
H	-0.93	3.352	0.355
H	-0.93	-3.353	-0.355
H	2.008	4.864	-1.051
H	3.316	6.899	-1.334
H	8.868	6.221	0.566
H	7.817	8.187	-0.434
H	5.43	8.129	-1.118
H	4.819	-0.644	1.77
H	7.097	-0.543	2.639
H	9.26	0.613	2.812
H	10.634	2.659	2.484
H	9.674	4.637	1.419
H	4.819	0.644	-1.77
H	7.097	0.543	-2.639
H	9.674	-4.637	-1.418
H	10.634	-2.659	-2.484
H	9.26	-0.613	-2.812
H	2.008	-4.864	1.051
H	3.316	-6.899	1.334
H	5.43	-8.129	1.118
H	7.817	-8.187	0.434
H	8.868	-6.221	-0.566
H	-2.008	4.864	1.051
H	-3.316	6.899	1.334
H	-8.868	6.221	-0.566
H	-7.817	8.187	0.434
H	-5.43	8.129	1.118
H	-4.819	-0.644	-1.77
H	-7.097	-0.543	-2.639

H	-9.26	0.613	-2.812
H	-10.634	2.659	-2.484
H	-9.674	4.637	-1.418
H	-2.008	-4.864	-1.051
H	-3.316	-6.899	-1.334
H	-8.868	-6.221	0.566
H	-7.817	-8.187	-0.434
H	-5.43	-8.129	-1.118
H	-4.819	0.644	1.77
H	-7.097	0.543	2.639
H	-9.26	-0.613	2.812
H	-10.634	-2.659	2.484
H	-9.674	-4.637	1.419
